# A Novel Analysis of the Peptide Terminome Characterizes Dynamics of Proteolytic Regulation in Vertebrate Skeletal Muscle Under Severe Stress

**DOI:** 10.3390/proteomes7010006

**Published:** 2019-02-13

**Authors:** Yuri Kominami, Tatsuya Hayashi, Tetsuji Tokihiro, Hideki Ushio

**Affiliations:** 1 Department of Aquatic Bioscience, Graduate School of Agricultural and Life Sciences, The University of Tokyo, 1-1-1 Yayoi, Bunkyo-ku, Tokyo 113-8657, Japan; komi.yuri@mail.u-tokyo.ac.jp; 2 Department of Mathematical Sciences, Graduate School of Mathematical Sciences, The University of Tokyo, 3-8-1 Komaba, Meguro-ku, Tokyo 153-8914, Japan; thayashi@ms.u-tokyo.ac.jp (T.H.); toki@ms.u-tokyo.ac.jp (T.T.)

**Keywords:** peptide terminome, proteolysis, peptidomic analysis, multiple linear regression model, non-model vertebrates

## Abstract

In healthy cells, proteolysis is orderly executed to maintain basal homeostasis and normal physiology. Dyscontrol in proteolysis under severe stress condition induces cell death, but the dynamics of proteolytic regulation towards the critical phase remain unclear. Teleosts have been suggested an alternative model for the study of proteolysis under severe stress. In this study, horse mackerel (*Trachurus japonicus*) was used and exacerbated under severe stress conditions due to air exposure. Although the complete genome for *T. japonicus* is not available, a transcriptomic analysis was performed to construct a reference protein database, and the expression of 72 proteases were confirmed. Quantitative peptidomic analysis revealed that proteins related to glycolysis and muscle contraction systems were highly cleaved into peptides immediately under the severe stress. Novel analysis of the peptide terminome using a multiple linear regression model demonstrated profiles of proteolysis under severe stress. The results indicated a phase transition towards dyscontrol in proteolysis in *T. japonicus* skeletal muscle during air exposure. Our novel approach will aid in investigating the dynamics of proteolytic regulation in skeletal muscle of non-model vertebrates.

## 1. Introduction

Proteolysis is a necessary biological process in all living organisms to sustain life. In healthy cells, proteolytic systems such as autophagy and ubiquitin-proteasome systems are methodically executed to maintain basal homeostasis and normal physiological status [1,2]. Many studies have shown that proteolysis can be induced by a wide variety of stresses, including starvation, hypoxia, heat shock, growth factor withdrawal, oxidative stress, and infections [1,2,3,4,5,6]. Apoptosis has also been shown to follow proteolytic events via activated caspases [7]. Appropriate regulations of cellular proteolytic processes are necessary for cell survival. For example, proteolytic dynamics should be drastically altered by cell death, and several studies have shown that impairment of proteolysis is associated with cell mortality [8,9], but comprehensive profiles of dyscontrolled proteolysis, including the dynamics of substrates and proteases, remain unknown.

A specific pattern of sequence at peptide termini has been mainly focused in previous studies related to cellular proteolysis because principal proteases in important cellular events are already identified. Additionally, proteolysis has been pointed out as one of the major post-translational modification processes and this find accelerates study on peptide termini analysis. Degradomic analysis using protein N-termini labeling has already been applied to characterize the proteolytic cleavage sites in apoptotic cells [10]. Degradomic analysis is indeed suitable for characterizing dominant proteolytic events such as caspase activity during apoptosis, but is limited to the identification of protein fragments that can be labelled. Peptidomic analysis, a more comprehensive analysis of peptides, is more suitable with the aim of exploring random peptide termini and a diversity of protein fragments. It has been recently developed in combination with some separation methods [11,12,13,14,15,16]. Size exclusion chromatography or molecular weight cutoff (MWCO) membranes are generally used to separate peptides from the proteinaceous fractions in sample preparation for peptidomic analyses [16]. The advantage of the peptidomic analyses is to discover peptides derived through proteolysis without modifications. The peptidomic analysis must contribute to the identification of dyscontrolled proteolysis under stress conditions, but includes two points of concern. First, the difficulty in identifying not only many substrates but also many proteases the cleave to them. Second, the application of peptidomic analysis to non-model and/or unsequenced organisms.

Peptidomic analysis facilitates profiling of small proteolytic fragments (<~10 kDa) and enables us to estimate major protein substrates in proteolysis (cleaved proteins). The sequence of all the peptide termini within the peptidome (referred to as the peptide terminome) changes according to proteolytic regulation. We have developed a multiple linear regression model for analysis of the peptide terminome to characterize dynamics of proteolytic regulation. In our model, sequence characteristics of the peptide terminome is expressed as the linear combination of cleavage site specificity of each protease with each contributing parameter. With regard to the difficulty associated with peptidomic analysis of unsequenced organisms, *de novo* transcriptome sequencing enables the construction of a protein database [17,18,19]. Many studies have already used large-scale RNA-sequencing to construct or refine databases for proteomic experiments, thereby improving the quality of protein identification and quantification [18,19]. 

There are many studies on proteolysis in teleost skeletal muscle under severe stress conditions, although whole genome sequences of teleosts remain largely unavailable [20,21,22]. Asphyxia in air is the commonly used method to slaughter fish in fish farms or fish vessels; however, it forces teleosts to struggle [23,24], and resulting in the deterioration of flesh quality by accelerating proteolysis [22,23,24,25,26]. Most previous studies have focused on the effects of asphyxia on *postmortem* protein degradation in teleost skeletal muscle [25,26], while *premortem* changes in proteolysis under severe stress conditions have not yet been explored. The aim of this study was to investigate the dynamics of proteolytic regulation under severe stress condition in non-model and/or unsequenced animals. Transcriptomic analysis was performed to construct a reference protein database for peptidomic analysis and to reveal the expression of genes encoding proteases in muscle tissue. Next, a quantitative peptidomic analysis was performed to profile cleaved proteins and characterize the dynamics of proteolytic regulation through a novel analysis of the peptide terminome.

## 2. Material and Methods

### 2.1. Reagents

Acetic acid (LC-MS grade) and trifluoroacetic acid (LC-MS grade) were purchased from Wako Pure Chemical (Osaka, Japan). Acetonitrile and water with 0.1% formic acid (LC-MS grade) were purchased from Thermo Fisher Scientific K.K. (Yokohama, Japan). Formic acid in water (0.1%, LC-MS grade) and formic acid in acetonitrile (0.1%, LC-MS grade) used as mobile phases in the liquid chromatography were purchased from Sigma-Aldrich Japan (Tokyo, Japan).

### 2.2. Fish Samples

All animal care and use were performed following the institutional protocol #AIMCB-404 that was approved by the University of Tokyo. Specimens of horse mackerel (*Trachurus japonicus*) were reared in a 1 m^3^ tank. The tank was connected to a recirculation system and the seawater temperature was kept at around 20 °C. After 2 days of starvation, 12 fish (standard length 16.0–19.3 cm, weight 63–112 g) were collected from the water, divided into 4 groups, and slaughtered under the following conditions: three individuals were decapitated immediately after being taken out of the water (Decap)., and three individuals were exposed to air for 1, 5, or 10 min (referred to as AirEx1, AirEx5, and AirEx10, respectively). The tissue specimens were dissected from dorsal skeletal muscle and flash frozen in liquid nitrogen. Frozen samples were stored at −80 °C until use.

### 2.3. Transcriptomic Analysis

#### 2.3.1. RNA Extraction

Total RNA was extracted using an RNeasy Plus Universal Kit (Qiagen, Tokyo, Japan) following the manufacturer’s instruction. The quality of RNA was assessed by electrophoresis using Agilent 4200 Tapestation (Agilent Technology, Tokyo, Japan). The concentration of total RNA was measured with the Qubit RNA BR Assay Kits (Thermo Fisher Scientific K.K., Yokohama, Japan).

#### 2.3.2. cDNA Library Construction and Sequencing

Twelve cDNA libraries representing 12 muscle tissue samples were constructed using a Truseq^TM^ Stranded mRNA sample prep Kit (Illumina K.K., Tokyo, Japan) according to the manufacturer’s instructions. Briefly, poly(A) mRNA was purified and fragmented using divalent cations at 80 °C for 2 min. Stranded cDNA libraries with index adapters were synthesized from the RNA fragments. The cDNA libraries were sequenced in an Illumina MiSeq sequencer (Illumina K.K., Tokyo, Japan) according to the manufacturer’s protocol, and pair-end reads (300 bp × 2) were obtained. The raw fastq files were deposited in the Sequence Read Archives (SRA) of the National Center for Biotechnology Information (NCBI) under accession number SRR8543815, SRR8543816, SRR8543817 and SRR8543818 of Bioproject PRJNA521293 and Biosample SAMN04485566.

#### 2.3.3. Sequence Data Processing and De Novo Assembly

The raw sequencing reads were processed using Trimmomatic Ver. 0.36 [27] to remove adapters and reads below 70 bp. The *de novo* assembly was performed using the Trinity Ver. 2.1.1 [28] platform in the DDBJ Read Annotation Pipeline with default settings. Contigs shorter than 200 bp were eliminated. Next, TransDecoder (http://transdecoder.sourceforge.net/) was used to identify candidate coding regions from the assembled contigs. The output file “longest.orf.pep” contains all the open reading frames (ORFs) that met the minimum length criteria (100 amino acids).

#### 2.3.4. Gene Expression Analysis of Protease

To detect proteases expressed in horse mackerel skeletal muscle, the expression level of proteases in each fish sample was explored. The protein dataset of Percomorphaceae from the NCBI Protein database was used to remove redundant contigs [29]. A non-duplicative database named Per40 DB was created by clustering the Percomorphaceae protein dataset with the CD-HIT program Ver. 4.6.4 [30] with an identity setting of 0.4. Redundant contigs in raw contigs were removed via a homology search with the Per40 DB [31]. The homology search with contigs as query sequences and the protein datasets of Per40 DB as the reference dataset was performed using the BLASTX algorithm with an e-value cut-off of 1 × 10^−5^. Each contig with the highest BitScore for each respective protein was selected as the annotated contig. After removing redundant contigs, the resulting contig set was designated as the Per40 DB contig set. The raw sequencing reads were mapped with the Bowtie2 aligner [32] to the Per40 DB contig set. The number of fragments per kilobase of exon per million mapped reads (FPKM) of each contigs was calculated using eXpress [33]. The FPKM was processed with EBMultiTest (the R package EBseq) [34] to identify expression levels of genes with maxround = 5, Qtrm = 1.0, and QtrmCut = −1. In EBMultiTest statistical significance in the gene expression is printed Pattern1, …, Pattern*n* (*n* depends on the number of conditions) and the posterior probability of being in each pattern for every gene is output. Functional annotation of genes by BLAST or GHOST comparisons against the manually curated KEGG GENES databases [35] was conducted by KEGG Automatic Annotation Server (KAAS; http://www.genome.jp/tools/kaas/). A binary relation files between a K number and the specific enzyme commission (EC) number downloaded from KEGG BRITE Database (http://www.genome.jp/kegg/brite.html) was used to convert K numbers to specific EC numbers. A homology search of the sequences attributed to the class of EC 3.4 was conducted by BLASTP with an e-value of 1e-5 against the protein dataset of Percomorphaceae. Unnamed protein products, uncharacterized proteins, and hypothetical proteins from the results of the first BLASTP were used as queries for the BLASTP run against the NCBI non-redundant protein (NR) database. After combining the two BLASTP results, the expression levels of genes coding for proteases were collected from the EBseq processing results. The super-computing resource was provided by the Human Genome Center at the Institute of Medical Science, the University of Tokyo.

### 2.4. Peptidomic Analysis

#### 2.4.1. Free Peptide Extraction

Free peptides were extracted individually from the frozen muscle tissues (*n* = 3, for each group) using a tissue homogenizer Precellys 24 (Bertin Technologies, Ampère Montigny-le-Bretonneux, France). A small portion (~100 mg) of frozen tissue was placed in a 1.5 mL screw cap micro tube (Sarstedt K.K., Tokyo, Japan) containing 5 ceramic beads (f2 mm) and 1 mL of 0.25% acetic acid. Each sample was homogenized at four times for 30 s at 6000 rpm. All extracts were clarified by centrifugation at 16,000 ×g and 4 °C for 60 min. Each supernatant was passed through a 5000 MWCO filter (Vivaspin 20, Sarstedt K.K., Tokyo, Japan). All filtrates were freeze-dried, dissolved in 0.1% formic acid and desalted by GL-Tip SDB (GL Sciences, Tokyo, Japan). Desalted samples were extracted by 80% acetonitrile in 0.1% trifluoroacetic acid solution and concentrated in a vacuum evaporator VC-15 sp (TAITEC, Tokyo, Japan).

#### 2.4.2. Liquid Chromatography

Prior to the mass spectrometry (MS) analysis, the concentrated peptides were diluted to approximately 2-fold with 0.1% formic acid and quantified by measuring absorbance at A_280_ nm with a NanoDrop 2000 spectrophotometer (Thermo Fisher Scientific K.K., Tokyo, Japan). The peptide samples were separated on an Eksigent cHiPLC^®^ system (AB SCIEX, Tokyo, Japan) equipped with a Nano cHiPLC ChromXP C18 column (75 µm × 15 cm; particle size, 3 µm; pore size, 120 Å; AB SCIEX Japan, Tokyo, Japan) at 25 °C. Mobile phase A containing 0.1% formic acid in water, and mobile phase B containing 0.1% formic acid in acetonitrile were used for the separation. The following gradient was used: 2% B for 0.1 min, from 2% B to 32% B in 70 min, from 32% B to 80% B in 1 min, 80% B for 4 min, 80% B to 2% B in 1 min, 2% B for 9 min at a flow rate of 300 nL/min. The same chromatographic conditions were applied to both shotgun and sequential window acquisitions of all theoretical mass spectra (SWATH) analyses. The separated peptides were injected directly into a TripleTOF^®^ 5600 mass spectrometer (AB SCIEX Japan, Tokyo, Japan) via a NanoSpray^®^ III Ion Source (AB SCIEX Japan, Tokyo, Japan) in the positive ion mode.

#### 2.4.3. Shotgun Analysis

Approximately 0.15 µg of the peptide was analyzed by a data-dependent shotgun method. A cycle of one full-scan MS spectrum (100–1600 *m*/*z*) with accumulation time of 0.1 s followed by 20 data-dependent MS/MS spectra (100–1800 *m*/*z*) with an accumulation time of 0.25 s was repeated continuously throughout the whole gradient. The dynamic exclusion time was set to 12 s. The following parameters were set for all shotgun analysis: ion spray voltage floating 2300 V, DP 80 V, CE 10 V, GS1 20 psi, GS2 0 psi, CUR 20 psi, and temperature 150 °C. The MS peptidomics data have been deposited to the ProteomeXchange Consortium (http://proteomecentral.proteomexchange.org) via the jPOST partner repository with the dataset identifier PXD 011911.

#### 2.4.4. SWATH Analysis

Approximately 0.15 µg of the peptide was quadruply analyzed using the SWATH method. A consecutive data-independent acquisition with 25 *m*/*z* increment (100–1600 *m*/*z*) in the precursor isolation window resulted in MS/MS across the 400–1250 *m*/*z* range. The accumulation time was set to 0.050 s for the MS scan and 0.096 s for the MS/MS scan. The total cycle time was approximately 3.4 s. The following parameters were set for all swath analysis: ion spray voltage floating 2300 V, DP 80 V, CE 10 V, GS1 20 psi, GS2 0 psi, CUR 20 psi, and temperature 150 °C.

#### 2.4.5. Database Search

ProteinPilot^®^ software version 4.5 (AB SCIEX) was used to analyze the shotgun MS data. The MS data were collectively queried against the predicted ORFs. A false discovery rate analysis in the ProteinPilot^®^ software was performed and *p*-value threshold was set to 0.05. The sequences identified to be present in the sample tissue were annotated via a two-step homology search against Per40 DB and NR database using BLASTP as mentioned above (see Section 2.3.4).

#### 2.4.6. SWATH Data Processing

ProteinPilot results were exported to PeakView^®^ software (AB SCIEX) for identification, quantification, and alignment of the spectral peaks. Specific tables of precursor masses and fragment ions were created and used to generate MS/MS peak-extracted ion chromatograms (XICs) of the fragment ions of targeted proteins and peptides in the SWATH processing by integrating the peak areas from the SWATH data. Shared peptides, and peptides with a confidence level below 80% were excluded. Each peptide peak area was obtained by the sum of fragment ion XICs from MS/MS data output by SWATH analysis. Then, the areas for multiple peptides per protein were summed to obtain protein areas. The report was used to perform relative quantification of cleaved proteins or released peptides. The reported quantitative peptidome data were normalized; the sum of elements measured in each SWATH analysis run was scaled to 1. Fold changes were calculated using relative cleaved protein abundance and proteins showing greater than 2.0-fold difference were considered significantly changed. The sequence specificity of the cleavage sites was calculated using the relative peptide abundance and reconstruction to a 20 by 4 matrix.

#### 2.4.7. Multiple Linear Regression Model

The matrix representing the cleavage site specificity of each protease which was confirmed for their gene expression in the *T. japonicus* muscle tissue was downloaded from the MEROPS database (http://merops.sanger.ac.uk/index.shtml) based on the EC numbers. The cleavage site specificity of each protease was manually reconstituted to a 20 × 4 matrix.

Generally, amino acid residues in a substrate are designated P1, P2, … in the N-terminal direction from the cleaved bond [36]. Similarly, the residues are designated P1′, P2′, … in the C-terminal direction. The protease *k* cleavage site specificity is expressed as a twenty by four matrix *X_k_*.

$$X_{k}=\left(\begin{array}{cc} \begin{array}{cc} x_{1}^{\left(k\right)} & y_{1}^{\left(k\right)}\\ x_{2}^{\left(k\right)} & y_{2}^{\left(k\right)} \end{array} & \begin{array}{cc} z_{1}^{\left(k\right)} & w_{1}^{\left(k\right)}\\ z_{2}^{\left(k\right)} & w_{2}^{\left(k\right)} \end{array}\\ \begin{array}{cc} \vdots & \vdots \\ x_{20}^{\left(k\right)} & y_{20}^{\left(k\right)} \end{array} & \begin{array}{cc} \vdots & \vdots \\ z_{20}^{\left(k\right)} & w_{20}^{\left(k\right)} \end{array} \end{array}\right)$$

where $x_{i}^{\left(k\right)}$, $y_{i}^{\left(k\right)}$, $z_{i}^{\left(k\right)}$ and $w_{i}^{\left(k\right)}\left(1\leq i\leq 20\right)$ corresponded to the appearance frequency of the amino acid *i* in P2, P1, P1′, and P2′ of the protease *k* cleavage site, respectively. Then, the sequence specificity in the peptide terminome is expressed as follow,

$$X_{p}=\left(\begin{array}{cc} \begin{array}{cc} \alpha_{1} & \beta_{1}\\ \alpha_{2} & \beta_{2} \end{array} & \begin{array}{cc} \gamma_{1} & \delta_{1}\\ \gamma_{2} & \delta_{2} \end{array}\\ \begin{array}{cc} \vdots & \vdots \\ \alpha_{20} & \beta_{20} \end{array} & \begin{array}{cc} \vdots & \vdots \\ \gamma_{20} & \delta_{20} \end{array} \end{array}\right)$$

here $\alpha_{i}$, $\beta_{i}$, $\gamma_{i}$ and $\delta_{i}$ (1≤i≤20) represent the appearance frequency of the amino acid *i* in P2, P1, P1′, and P2′, respectively. The matrix *X_p_* can be expressed as a linear combination of *X_k_* by the following equation

(1) $$X_{p}=\overset{N}{\underset{k=1}{\sum }}f_{k}X_{k}$$

where *N* is the number of kinds of proteases (1≤k≤N), and $f_{k}$ is a parameter corresponding to each protease contribution. The parameter $f_{k}$ satisfies the following constraints,

{(2)∑k=1Nfk=1(3) fk≥0

Let us define an evaluation function Z, to derive an optimized combination of $\left(f_{1},f_{2},\ldots ,f_{N}\right)$.

(4) $$\overset{20}{\underset{i=1}{\sum }}{\left(\overset{N}{\underset{k=1}{\sum }}x_{i}^{\left(k\right)}f_{k}-\alpha_{i}\right)}^{2}+\overset{20}{\underset{i=1}{\sum }}{\left(\overset{N}{\underset{k=1}{\sum }}y_{i}^{\left(k\right)}f_{k}-\beta_{i}\right)}^{2}+\overset{20}{\underset{i=1}{\sum }}{\left(\overset{N}{\underset{k=1}{\sum }}z_{i}^{\left(k\right)}f_{k}-\gamma {\;}_{i}\right)}^{2}\;+\overset{20}{\underset{i=1}{\sum }}{\left(\overset{N}{\underset{k=1}{\sum }}w_{i}^{\left(k\right)}f_{k}-\delta_{i}\right)}^{2}\;$$

The combination of $\left(f_{1},f_{2},\ldots ,f_{N}\right)$ was used as an index characterizing the proteolytic regulation. The matrix *X_p_* was respectively calculated for each group (Deacap, AirEx1, 5, and 10) by combining the SWATH data of three individuals with *N* = 45. The mathematical analysis on the linear regression model was executed in Mathematica 10.0.1 (Wolfram Research Inc.).

## 3. Results

### 3.1. Expression of Genes Encoding Proteases

Sequenced reads were obtained with Illumina sequencing of 12 cDNA libraries (4 groups × 3 individuals). The trinity assembler generated a total of 279,308 contigs (Table 1). After removal of redundant contigs via a homology search against Per40 DB, a total of 17,214 contigs were obtained as the non-redundant reference sequence data set (Per40 DB contig set). There were 165 sequences classified to EC number 3.4 in the Per40 DB contig set, and 156 sequences were annotated as proteases (Table 1).

The expression patterns of genes encoding proteases in *T. japonicus* skeletal muscle during air exposure are listed in Table S1. Air exposure induced expression of many proteases, but more than half of the genes encoding proteases were expressed without any significant differences during the air exposure treatments. Compared to the Decap group, 13 and 3 genes encoding proteases were highly expressed in the AirEx1 and AirEx5 groups, respectively. No significant increase in protease expression was observed in the AirEx10 group. Among these differentially expressed genes, few components of the ubiquitin–proteasome system were detected. Meanwhile, the expression levels of 41 genes encoding isoforms of ubiquitin carboxyl-terminal hydrolase were kept constant in *T. japonicus* skeletal muscle during air exposure. No significant increase was observed in all identified caspase family proteases and lysosomal proteases under severe stress condition.

### 3.2. Protein cleavage levels

Peptide identification was performed by database search of the acquired MS data against the predicted ORFs. Sequenced peptides were attributed to 479 ORFs including 14 reverse sequences and sequences with low confidence (Table S5,S6). Of these ORFs, 155 ORFs were quantified by SWATH analysis (Table S7) and 135 ORFs were annotated through a homology search using BLASTP. The result of peptide mapping is shown in Supplementary Data (File S1).

**Figure 1:**
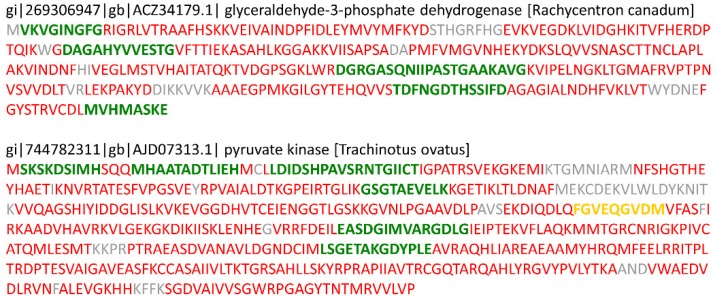
The results of peptide mapping for glyceraldehyde-3-phosphate dehydrogenase and pyruvate kinase. The color of character indicates the peptide confidence; green, yellow, and red means the peptide confidence is ≥95%, <95% ∩ ≥50% and <50%, respectively.

Cleaved proteins with a fold changes greater than two in the AirEx1, AiEx5, and AirEx10 groups compared with the Decap group included 19, 20, and 14 proteins, respectively (Table 2). Compared to the Decap group, fold changes of skeletal muscle fast troponin T isoform 2, ankyrin repeat domain-containing protein 1, and myosin heavy chain showed over a 2-fold changes for the other 3 groups (Table 3). In the AirEx1 group compared with the Decap group, glyceraldehyde-3-phosphate dehydrogenase and pyruvate kinase, which are related to glycolysis, were markedly targeted. Some of the proteins that make up muscular fibril or ribosomes were highly cleaved in *T. japonicus* skeletal muscle under severe stress conditions. Figure 1 shows the results of peptide mapping for glyceraldehyde-3-phosphate dehydrogenase and pyruvate kinase. Small peptides generated from N-or C-terminus were observed.

### 3.3. Dynamics of Proteolytic Regulation

In total, 156 sequences in the *de novo* assembled transcripts were classified to the class of EC 3.4 and annotated as proteases. The line number of the EC 3.4 classes to which contigs were classified was 72, and 45 protease cleavage site specificities were derived from the MEROPS database [37]. All obtained protease cleavage site specificities were manually reconstituted to a 20 × 4 matrix. The sequence specificity for cleavage events for each group was calculated from the result of quantitative peptidomic analysis (Table S3).

**Figure 2:**
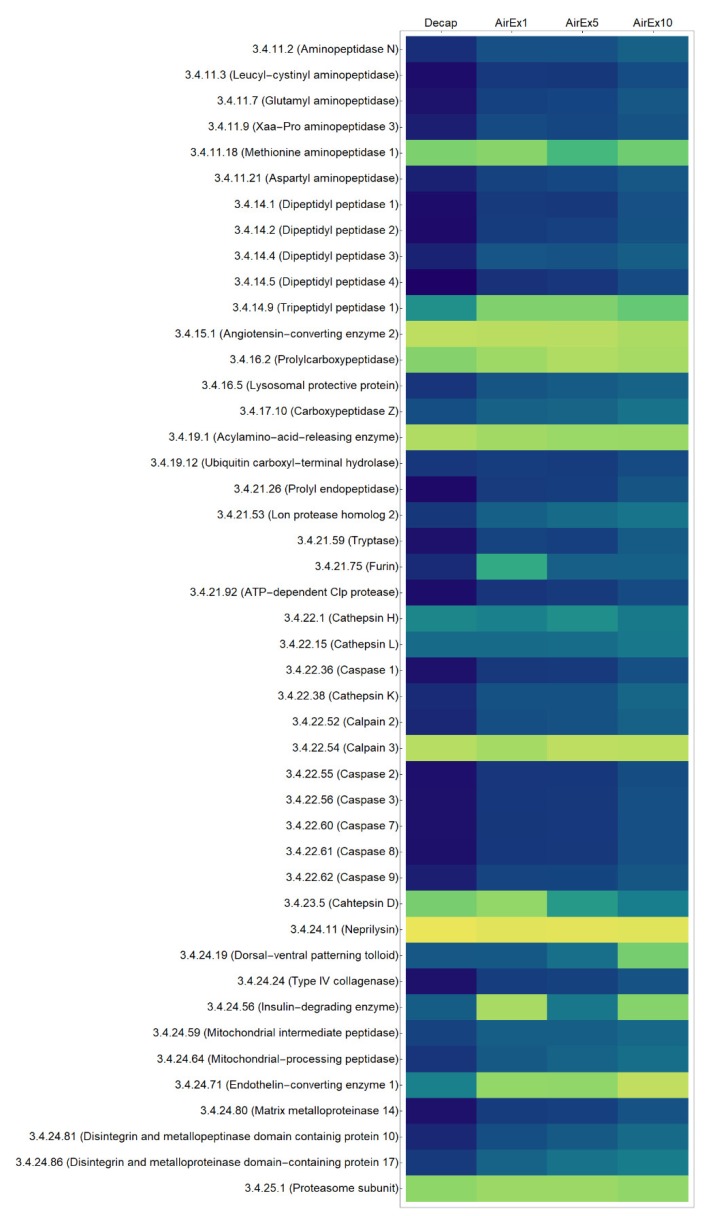
Peptide terminome analysis. Rows represent individual protease, columns represent each sample group. Each cell is colored according to the normalized contribution parameter of each protease calculated by equation (5). Each protease inserted with the E.C. number was confirmed for their gene expression in the *T. japonicus* muscle tissue.

The results of the analysis of the peptide terminome using the multiple linear regression model are shown as a heatmap in Figure 2. The heatmap (Figure 2) was generated from the calculation results (Table S4); cells are respectively colored according to the value of $f\prime_{k}^{s}$ calculated by the following equation:

(5) $$f\prime_{k}^{s}=\log_{10}\left(\frac{N\cdot f_{k}^{s}}{\sum_{s}^{}\sum_{k=1}^{N}f_{k}^{s}\;}\right)$$

where *s* is the sample group (Decap, AirEx1, AirEx5 or AirEx10) and $\sum_{s}$ is the sum of $f_{k}^{s}$ for all sample groups. However, each value (as shown in Table S4) does not indicate the actual protease activity, but rather is a product of all parameters related to protease hydrolysis such as activity, amount, and specificity. All $f_{k}$ (parameter corresponding to each protease contribution) will be equal to the in vitro proteolysis with a protease mixture, while some $f_{k}$ will be markedly higher in the orderly proteolysis of intracellular proteins such as during apoptosis.

A specific distribution pattern of $\left(f\prime_{1}^{s},f\prime_{2}^{s},\ldots ,f\prime_{N}^{s}\right)$ was observed in each group (Figure 2). In the Decap, AirEx1, and AirEx5 groups, most cells were >4 or <−1. Most cells in AirEx10, on the other hand, were around 0. The contributions of most EC groups tended to increase with increasing air exposure time. All $f\prime_{k}^{s}$ in the whole group were plotted (Figure S1). There was a clear trend in equalization of $f\prime_{k}^{s}$ in air exposure for 10 min.

## 4. Discussion

In this study, a novel approach for characterizing proteolytic dynamics in an unsequenced non-model animal was established and adopted to describe dyscontrolled proteolysis in fish skeletal muscle under severe stress conditions. *T. japonicus* was used as a non-model animal and exacerbated under severe stress conditions due to air exposure. Most carnivorous fish, including *T. japonicus* show insulin resistance, one of the common features of human type 2 diabetes [38]. They have a low capacity to use dietary carbohydrates and a high requirement of amino acids [39]. In *T. japonicus*, skeletal muscle is not only a locomotive organ, but also an amino acid pool as an energy resource. Amino acids are therefore released immediately upon protein degradation probably via proteolysis for energy metabolism in carnivorous fish skeletal muscle under stress conditions [21].

Proteases expressed in *T. japonicus* skeletal muscle were first identified, and the changes in their expression levels under severe stress conditions were revealed. The removal of redundant contigs from *de novo* RNA-seq assemblies via homology searches resulted in reference sequences for gene expression analysis with little redundancy. The method for removing redundancy from contigs suggested by Ono et al. (2015) [31] is thus useful for transcriptomic analysis in unsequenced animals. The results of protease expression analysis suggested an increase in the expression of a few genes related to the ubiquitin-proteasome system in the AirEx1 and AirEx5 groups compared with the Decap group, while no significant increase was observed in the AirEx10 group. The expression of few genes related to other biological processes except for proteolysis, translation, tricarboxylic acid cycle, and targeting of proteins to a membrane, were significantly increased (data was not shown) in the AirEx5 and AirEx10 groups. It is suggested that degradation rates of transcripts exceeded generation rates with nuclear destruction due to excessive contraction. The chromatin condensation in *T. japonicus* muscle after 10 min of air exposure has already been observed by transmission electron microscope (data was not shown). The changes in mRNA expression for the proteases did not appear to affect the protease protein levels during air exposure for 10 min.

Changes in protein cleavage levels were detected using the quantitative peptidomic analysis. The cytosolic proteins related to glycolysis were highly cleaved in the AirEx1 group. Additionally, high-level cleavage of the sarcomeric proteins were observed in the AirEx groups. A previous study [25] demonstrated that the deterioration in the integrity of sarcomeric and cytosolic proteins in sea bass (*Dicentrarchus labrax*) skeletal muscle due to struggle in air via proteomic analysis; thus, the result of peptidomic analyses of the present study are consistent with the results of the previous study. Most of proteins were cleaved randomly as shown in Figure 1. The results of peptide mapping indicate that the proteolysis under severe stress hardly contributes to the post-translational modification processes.

Interestingly, 26S proteasome non-ATPase regulatory subunit 14, which has a protease function as a component of the 26S proteasome, was cleaved frequently in the AirEx groups (Table S2). This indicates that the protease was also a proteolytic substrate under severe stress conditions. Yamashita (2010) observed hemolysis in the blood of *Thunnus orientalis* after 5 min of air exposure [22]. The hemolysis is known to be induced by apoptosis under oxidative stress. Therefore, we hypothesize that severe stress causes apoptosis first followed by dyscontrol of proteolysis, resulting in the degradation of proteases.

The results of the analysis of the peptide terminome showed that the distribution pattern of parameters $\left(f\prime_{1}^{s},f\prime_{2}^{s},\ldots ,f\prime_{N}^{s}\right)$ corresponding to each protease contribution was drastically changed in *T. japonicus* skeletal muscle under severe stress conditions. Our results suggest that broad spectra of the contribution of proteases to proteolysis should occur within 5–10 min after air exposure. Dyscontrol of proteolysis systems in skeletal muscle under the severe stress could be thus estimated by increasing contribution parameters with increasing time of air exposure.

Taken together, the results of this study indicate that proteolytic regulation in *T. japonicus* skeletal muscle changes drastically or breaks down in a few minutes under the severe stress conditions; the lineup of proteases and substrate proteins participate in proteolytic changes immediately in response to stress. These *premortem* changes due to stress at slaughter would have large impacts on the flesh quality, and therefore raise unfavorable *postmortem* changes as reported in the previous studies [22,24,25,26].

## 5. Conclusions

The original database generated from RNA-seq data was useful for peptidomic analysis of a non-model organism. We successfully identified both peptide sequences and substrate proteins of proteolysis in vivo by a peptidomic analysis. A multiple linear regression model was developed and applied for analysis of the peptide terminome. The results of analysis of the peptide terminome indicated serious *premortem* changes in *T. japonicus* skeletal muscle due to asphyxia in air; a phase transition occurred towards dyscontrol in proteolysis.

Our peptidomic analysis revealed a profile of the cleaved proteins by detecting cleaved proteins with a higher sensitivity than proteomic analysis. The present analysis of the peptide terminome using a multiple linear regression model provides new insights into proteolytic regulation. Our methodological framework can be applied for research on any animal tissues and thus may accelerate studies on proteolysis.

## Figures and Tables

**Table 1:** Summary of transcriptome assembly of the horse mackerel *Trachurus japonicus*.

Feature	Number
Number of total reads	30,554,904
Number of assembled contigs	279,308
Number of contigs after removal of redundancy	17,214
N50	1793 bp
Minimum contig length	201 bp
Maximum contig length	27,079 bp
Total length	20,694,310 bp
Number of contigs classified to EC 3.4	165
Number of contigs annotated as protease	156

Under dotted line, results after removal of redundancy are shown.

**Table 2:** The number of proteins with 2- or more fold change between the Decap and experimental groups calculated through the quantitative peptidome.

Feature	Number		
	AirEx1	AirEx5	AirEx10
Total proteins	19	20	14
Sarcomeric proteins	8	12	4
Cytosolic proteins	7	2	2
Other proteins	4	6	8

**Table 3:** Fold changes of cleaved proteins in *T. japonicus* skeletal muscle under the severe stress condition.

Cleaved Protein ^*^	Fold Change ^**^		
	AirEx1/Decap	AirEx5/Decap	AirEx10/Decap
PREDICTED: nuclear factor NF-kappa-B p105 subunit [Nothobranchius furzeri]	0.48	0.79	0.80
PREDICTED: phosphoglycerate kinase 1 [Nothobranchius furzeri]	**2.65**	1.57	1.93
PREDICTED: keratin, type II cytoskeletal cochleal-like [Nothobranchius furzeri]	1.70	**3.64**	1.90
PREDICTED: cofilin-2 [Nothobranchius furzeri]	1.01	1.49	0.87
PREDICTED: elongation factor 1-alpha [Nothobranchius furzeri]	0.79	0.49	0.75
Nebulin [Fundulus heteroclitus]	1.03	0.72	0.62
40S ribosomal protein S19 [Fundulus heteroclitus]	0.37	0.53	0.69
Nebulin [Fundulus heteroclitus]	1.53	1.76	1.69
Skeletal muscle fast troponin T isoform 2 [Hippoglossus hippoglossus]	**2.83**	**2.80**	**2.88**
Skeletal muscle fast troponin T isoform 2 [Hippoglossus hippoglossus]	1.62	1.09	0.71
Hemoglobin subunit beta [Decapterus maruadsi]	1.15	0.34	0.26
Muscle actin OlMA1 [Oryzias latipes]	0.51	0.96	1.89
Lactate dehydrogenase-A [Sphyraena idiastes]	**4.59**	1.00	1.24
Troponin I [Siniperca chuatsi]	0.94	0.89	0.40
PDZ and LIM domain 7 [Epinephelus coioides]	1.75	1.58	1.47
Glycerol-3-phosphate dehydrogenase [Rachycentron canadum]	1.15	0.28	0.25
Glyceraldehyde-3-phosphate dehydrogenase [Rachycentron canadum]	**11.96**	1.37	0.79
Parvalbumin 3 [Siniperca chuatsi]	0.49	0.33	0.85
Troponin I [Epinephelus coioides]	1.88	1.16	1.34
Ubiquitin [Siniperca chuatsi]	0.46	0.70	1.11
Keratin 8, partial [Oreochromis mossambicus]	**3.03**	**19.68**	0.93
Tropomyosin [Siniperca chuatsi]	**2.61**	0.77	0.60
PREDICTED: desmin-like [Oreochromis niloticus]	1.42	0.87	0.60
PREDICTED: nudC domain-containing protein 1 [Oreochromis niloticus]	1.37	**2.49**	1.84
PREDICTED: keratin, type II cytoskeletal 8-like [Oreochromis niloticus]	1.98	**7.34**	1.63
PREDICTED: 28S ribosomal protein S36, mitochondrial [Oreochromis niloticus]	1.35	0.91	0.89
PREDICTED: protein deglycase DJ-1 [Oreochromis niloticus]	0.99	0.78	0.94
PREDICTED: apoptosis-inducing factor 1, mitochondrial [Oreochromis niloticus]	0.75	0.31	0.34
Parvalbumin 1 [Siniperca chuatsi]	1.40	0.58	0.60
PREDICTED: early endosome antigen 1-like [Oryzias latipes]	**3.59**	**3.59**	0.38
PREDICTED: myozenin-2 [Maylandia zebra]	0.16	0.92	**2.10**
PREDICTED: troponin I, fast skeletal muscle-like [Maylandia zebra]	0.13	0.07	0.05
PREDICTED: myozenin-1-like [Oreochromis niloticus]	0.95	1.08	0.92
PREDICTED: ankyrin repeat domain-containing protein 1 [Pundamilia nyererei]	**5.85**	**2.22**	**2.84**
PREDICTED: myosin heavy chain, fast skeletal muscle-like [Pundamilia nyererei]	1.77	1.58	1.40
PREDICTED: keratin, type I cytoskeletal 18-like [Xiphophorus maculatus]	1.83	**2.84**	1.06
PREDICTED: aldose reductase-like [Haplochromis burtoni]	0.58	1.93	1.03
PREDICTED: creatine kinase M-type [Haplochromis burtoni]	1.09	1.13	1.22
Type 1 collagen alpha 2 [Paralichthys olivaceus]	0.86	0.21	0.33
SET and MYND domain containing protein 1a [Siniperca chuatsi]	1.46	**2.92**	1.95
PREDICTED: collagen alpha-2(I) chain-like isoform X1 [Neolamprologus brichardi]	0.98	0.94	0.46
PREDICTED: myc box-dependent-interacting protein 1-like isoform X1 [Neolamprologus brichardi]	1.12	1.75	**2.18**
PREDICTED: ATP synthase subunit beta, mitochondrial-like [Neolamprologus brichardi]	0.74	0.73	1.52
PREDICTED: putative peptidyl-tRNA hydrolase PTRHD1-like [Neolamprologus brichardi]	1.06	1.37	0.84
Cytochrome c oxidase subunit VIa precursor [Thunnus obesus]	0.59	0.26	0.54
PREDICTED: 60S ribosomal protein L3-like [Poecilia formosa]	0.70	1.98	**2.78**
Alpha hemoglobin A [Seriola quinqueradiata]	1.60	1.52	0.17
PREDICTED: myosin-binding protein H-like isoform X1 [Stegastes partitus]	0.31	0.92	0.72
PREDICTED: dnaJ homolog subfamily C member 8 [Stegastes partitus]	1.79	0.84	0.48
PREDICTED: proteasome subunit alpha type-1 [Stegastes partitus]	0.19	0.38	0.63
PREDICTED: 40S ribosomal protein S25 [Stegastes partitus]	0.66	1.04	0.78
PREDICTED: malate dehydrogenase, mitochondrial [Stegastes partitus]	0.61	0.32	0.34
PREDICTED: adenylate kinase isoenzyme 1 [Stegastes partitus]	0.26	0.07	0.16
PREDICTED: transcription factor BTF3 homolog 4 isoform X2 [Stegastes partitus]	0.75	0.92	1.17
PREDICTED: unconventional myosin-XVIIIb isoform X1 [Stegastes partitus]	0.38	0.67	0.20
PREDICTED: nascent polypeptide-associated complex subunit alpha isoform X1 [Stegastes partitus]	0.79	1.54	1.60
PREDICTED: poly(rC)-binding protein 2-like isoform X2 [Stegastes partitus]	1.17	1.28	0.92
PREDICTED: 26S proteasome non-ATPase regulatory subunit 14 [Stegastes partitus]	1.47	**4.82**	**3.08**
PREDICTED: voltage-dependent anion-selective channel protein 3 isoform X2 [Stegastes partitus]	1.44	0.41	0.84
PREDICTED: reticulon-3-like isoform X1 [Stegastes partitus]	0.60	0.43	0.51
PREDICTED: myomesin-1-like isoform X3 [Stegastes partitus]	1.78	**3.11**	1.48
PREDICTED: dehydrogenase/reductase SDR family member 7C-A-like [Stegastes partitus]	**2.23**	0.47	0.78
PREDICTED: ATP-binding cassette sub-family A member 1-like isoform X2 [Stegastes partitus]	1.86	**2.57**	0.46
PREDICTED: transcription factor E2-alpha-like isoform X1 [Stegastes partitus]	0.81	1.26	1.19
PREDICTED: creatine kinase M-type [Stegastes partitus]	1.10	0.93	1.03
PREDICTED: titin-like isoform X11 [Stegastes partitus]	**4.95**	1.22	1.72
PREDICTED: titin-like isoform X20 [Stegastes partitus]	1.69	**2.06**	1.51
PREDICTED: peptidyl-prolyl cis-trans isomerase A-like [Stegastes partitus]	1.03	1.16	0.69
PREDICTED: glutamate dehydrogenase, mitochondrial [Stegastes partitus]	0.65	1.00	0.99
PREDICTED: retinal dehydrogenase 2 [Stegastes partitus]	1.71	**2.74**	**8.28**
PREDICTED: creatine kinase S-type, mitochondrial [Stegastes partitus]	0.93	0.57	0.56
PREDICTED: CDK5 regulatory subunit-associated protein 2 isoform X3 [Stegastes partitus]	**2.13**	**2.19**	1.18
PREDICTED: sarcoplasmic/endoplasmic reticulum calcium ATPase 1 isoform X1 [Stegastes partitus]	0.94	0.91	0.89
PREDICTED: calpain-3-like isoform X1 [Stegastes partitus]	0.57	0.62	1.07
PREDICTED: creatine kinase M-type-like [Stegastes partitus]	0.88	1.20	1.59
PREDICTED: 40S ribosomal protein S11 [Stegastes partitus]	0.85	0.66	0.77
PREDICTED: mucin-5AC-like [Cynoglossus semilaevis]	1.29	1.27	0.77
PREDICTED: NADH dehydrogenase [ubiquinone] 1 alpha subcomplex subunit 8 [Poecilia reticulata]	1.23	1.33	1.23
PREDICTED: phosphate carrier protein, mitochondrial isoform X1 [Poecilia reticulata]	0.74	0.72	0.51
PREDICTED: lactoylglutathione lyase-like [Larimichthys crocea]	0.99	0.60	0.38
PREDICTED: myelin basic protein-like isoform X2 [Larimichthys crocea]	1.47	0.95	1.04
PREDICTED: 14 kDa phosphohistidine phosphatase-like [Larimichthys crocea]	0.40	0.63	0.24
PREDICTED: LOW QUALITY PROTEIN: myosin heavy chain, fast skeletal muscle-like [Larimichthys crocea]	1.13	1.13	0.82
PREDICTED: AMP deaminase 1 isoform X1 [Larimichthys crocea]	0.92	0.66	0.41
PREDICTED: myosin-binding protein C, fast-type-like isoform X5 [Larimichthys crocea]	0.40	0.95	0.40
PREDICTED: glucose-6-phosphate isomerase [Larimichthys crocea]	1.24	0.66	0.81
PREDICTED: SH3 domain-binding glutamic acid-rich protein isoform X2 [Larimichthys crocea]	0.60	1.60	1.79
PREDICTED: peroxisome proliferator-activated receptor gamma coactivator-related protein 1-like [Larimichthys crocea]	0.93	1.01	1.38
PREDICTED: 60S ribosomal protein L4-B isoform X1 [Larimichthys crocea]	0.39	0.89	1.19
PREDICTED: NADH dehydrogenase [ubiquinone] iron-sulfur protein 6, mitochondrial [Larimichthys crocea]	0.48	0.31	0.56
PREDICTED: alpha-2-macroglobulin-like isoform X1 [Larimichthys crocea]	**2.96**	**2.61**	1.81
PREDICTED: protocadherin-18-like isoform X2 [Larimichthys crocea]	0.48	0.17	0.44
PREDICTED: guanidinoacetate N-methyltransferase [Larimichthys crocea]	0.60	1.15	1.28
PREDICTED: calpastatin isoform X10 [Larimichthys crocea]	1.33	1.97	1.14
PREDICTED: fructose-bisphosphate aldolase A-like [Larimichthys crocea]	1.23	1.01	1.19
PREDICTED: proteoglycan 4-like [Larimichthys crocea]	1.32	0.80	0.57
PREDICTED: plasminogen activator inhibitor 1 RNA-binding protein-like isoform X1 [Larimichthys crocea]	0.82	0.71	1.83
PREDICTED: alpha-actinin-3 [Larimichthys crocea]	0.79	0.93	0.28
PREDICTED: protein S100-A14 [Larimichthys crocea]	1.74	0.59	0.49
PREDICTED: methionine aminopeptidase 2 [Larimichthys crocea]	0.81	1.03	0.82
PREDICTED: muscle-related coiled-coil protein-like [Larimichthys crocea]	0.96	1.10	0.71
PREDICTED: asparagine--tRNA ligase, cytoplasmic [Larimichthys crocea]	0.43	0.28	0.35
PREDICTED: patellin-2-like [Notothenia coriiceps]	**2.04**	**5.15**	**4.29**
PREDICTED: collagen alpha-1(I) chain-like [Notothenia coriiceps]	1.18	0.05	0.03
Pyruvate kinase [Trachinotus ovatus]	**9.68**	1.71	1.41
PREDICTED: 60S ribosomal protein L9 isoform X1 [Oryzias latipes]	0.36	1.58	**2.13**
PREDICTED: rho guanine nucleotide exchange factor 9 isoform X3 [Oryzias latipes]	0.46	1.71	**2.13**
Myosin light chain 1 [Trachurus trachurus]	1.93	0.49	0.32
Myosin light chain 3 [Trachurus trachurus]	1.48	0.79	0.74
Myosin light chain 2 [Trachurus trachurus]	1.20	0.70	0.51
White muscle parvalbumin [Trachurus japonicus]	1.41	0.63	0.52
Peroxiredoxin 5 [Oplegnathus fasciatus]	0.47	0.46	0.30
Nebulin [Larimichthys crocea]	1.14	1.05	1.04
Heat shock cognate protein [Larimichthys crocea]	0.75	1.25	0.93
Ral GTPase-activating protein subunit beta [Larimichthys crocea]	1.01	0.64	0.98
Myosin heavy chain, fast skeletal muscle [Larimichthys crocea]	**2.93**	**3.09**	**2.74**
PREDICTED: ATP-dependent 6-phosphofructokinase, muscle type-like [Fundulus heteroclitus]	0.63	0.25	0.18
PREDICTED: hematological and neurological expressed 1-like protein [Fundulus heteroclitus]	**2.07**	1.28	0.74
PREDICTED: LIM domain-binding protein 3-like isoform X3 [Oreochromis niloticus]	0.61	0.45	0.39
PREDICTED: PDZ and LIM domain protein 5 isoform X4 [Oreochromis niloticus]	**4.74**	0.69	0.51
PREDICTED: phosphorylase b kinase gamma catalytic chain, skeletal muscle/heart isoform [Oreochromis niloticus]	0.54	0.68	1.99
PREDICTED: neurofilament heavy polypeptide-like isoform X5 [Pundamilia nyererei]	1.63	0.79	0.53
PREDICTED: dysferlin [Pundamilia nyererei]	1.54	0.72	0.43
PREDICTED: fibrous sheath CABYR-binding protein-like [Haplochromis burtoni]	**3.52**	**4.93**	1.77
PREDICTED: glycogen phosphorylase, muscle form isoform X1 [Xiphophorus maculatus]	0.83	1.06	1.14
Aldolase A, partial [Poeciliopsis prolifica]	0.90	0.91	0.84
PREDICTED: 40S ribosomal protein S2 [Xiphophorus maculatus]	0.59	1.18	**2.10**
PREDICTED: triosephosphate isomerase [Poecilia latipinna]	0.76	0.74	0.76
PREDICTED: keratin, type I cytoskeletal 18 [Poecilia latipinna]	**2.05**	**4.94**	1.06
PREDICTED: guanine nucleotide-binding protein subunit beta-2-like 1 [Poecilia mexicana]	0.52	0.84	1.43
PREDICTED: phosphoglycerate mutase 2 [Cyprinodon variegatus]	0.68	1.21	1.80
PREDICTED: cold-inducible RNA-binding protein isoform X4 [Cyprinodon variegatus]	1.57	**2.13**	1.68
PREDICTED: glycogen [starch] synthase, muscle-like [Cyprinodon variegatus]	0.54	1.21	**2.55**
PREDICTED: ATP synthase subunit delta, mitochondrial [Cyprinodon variegatus]	0.95	1.79	1.88
PREDICTED: beta-enolase [Cyprinodon variegatus]	1.15	1.37	1.51

* The protein sequences predicted from open reading frames (ORFs) were used for MS sequence database search. Each protein name was identified using BLASTP. ** The result of quantitative peptidomic analysis was normalized; the sum of elements measured in each SWATH analysis run was scaled to 1. Fold change between the Decap and another group are calculated from the quantitative peptidome. Two-fold or more differences are indicated by boldface font.

## Supplementary Materials

The following are available online at https://www.mdpi.com/2227-7382/7/1/6/s1, Table S1: Expression of protease-coding genes in *T. japonicus* skeletal muscle. Table S2: Fold changes of free peptides in *T. japonicus* skeletal muscle under the severe stress condition. Table S3: The sequence specificity in both terminals of the whole peptidome, respectively calculated from the result of quantitative peptidomic analysis. Table S4: Raw contribution parameters of proteases. Rows represent individual proteases, and columns represent each sample group. Table S5: The raw protein identification data output from ProteinPilot^®^ software. Table S6: The raw peptide identification data output from ProteinPilot^®^ software. Table S7: The raw SWATH data output from PeakView Software. File S1. The result of peptide mapping. File S2. The reference proteome data originally constructed from RNA-seq data.
