# Supplementary material for: A Novel Analysis of the Peptide Terminome Characterizes Dynamics of Proteolytic Regulation in Vertebrate Skeletal Muscle Under Severe Stress
Source: Proteomes. 2019 Feb 13;7(1):6. doi: 10.3390/proteomes7010006 (PMC6473766; doi:10.3390/proteomes7010006)
Supplement: Supplementary file 1 [file proteomes-07-00006-s001.zip › supplementary/File S1.docx]

| ORF* | Top-hit gene ID in BLASTX** | Top-hit gene definition in BLASTX** | Coverage (%) | Peptide mapping*** |
| --- | --- | --- | --- | --- |
| TRINITY_DN45806_c0_g1::TRINITY_DN45806_c0_g1_i1::g.104285::m.104285 | gi\|958242933\|gb\|JAO38253.1\| | ALDOA, partial [Poeciliopsis prolifica] | 95 | GRINTRARGPEGCRRRRPDLLPPSLFSQLSSQTQRVRMPHAYPFLTPEQKKELS**DIAHKIVAPG**KG**ILAADESTGSVAKRFQSIN**AENTEENRRLYR**QLLFTADDRVAPCIGGVILFHETMYQ**KADSGKTFPAHLKERHMVVG**IKVDKGVVPLAGTNGETTTQGLDGLYER**CAQYKKDGADFAKWRCVL**KITPTTPSRLAIIENANVLARYAS**ICQM**HGIVPIVE**PEILPDGDHDLKRCQ**YVTEKVLA**AVYKALSDHHVY**LEGTLLKPNMVT**AGHSCSHKYS**NQEIAMATVTALRRTVPPAVPGITFLSGGQSEEEASVNLNAMNQCPLHRP**WALTF**SYGRALQASALK**AWAGKKENGKACQEEFVKR**ALANSQACQGKYAASGASAAGGESLFVANHAY** |
| TRINITY_DN58624_c13_g1::TRINITY_DN58624_c13_g1_i1::g.62013::m.62013 | gi\|548512008\|ref\|XP_005750706.1\| | PREDICTED: myosin heavy chain, fast skeletal muscle-like [Pundamilia nyererei] | 90.8 | M**STDAEMAVYGKAAIY**LRKPERERIEAQSKPFDAKAACYVTDAKELYLKGTIIKKDGGKVTVK**VLDTQEEKTVKEDDVSQMNPPKYDKIED**MAMMTYLNEASVLYNLKERYAAWMIYTYSGLFCATVNPYKWLPVY**DSEVVNAYR**GKKRMEAPPHIFSVSDNAYQF**MLTDRENQSVLITGESGAGKTVNT**KRVIQYFATISVGGGDKKKDAAPGKMQGSLE**DQIIAANPLLEA**YGNAK**TVRNDNSSRFG**KFIRIHFG**TTGKLASADIET**YLLEKSRVTFQLPDERGYHIFYQMMTNHKPELIEMSLITTNPYDFPMCSMGQITVASIDDKEELEATDNAIDILGFTNEEKMGIYKLT**GAVLHHGNMM**FKQKQREEQAEPDGTEVADKI**AYLLGLNSADMLKAL**CFPRVKVGNEFVTKGQTVPQVKNSVTALAKSIYERMFLWMVIRINQMLDTKQQRNYFIGVLDIAGFEIFDFNTLEQLCINFTNEKLQQFFNHHMFVLEQEEYKKEGIIWEFIDFGMDLAACIELIEKPMGIFSILEEECMFPKATDTSFK**NKLYDQHLGKTKAFE**KPKPAKGKAEAHFS**LVHYAGTVDYNIG**GWL**DKNKDPLNDSVVQLYQKSPVKI**L**AALYPPVVEEVG**KKGGKKKGGSMQ**TVSSQFRENLGKLMTN**LRSTHPHFVRCL**IPNESKTPGLMENF**LVIHQLRCNGVLEGIRICRKGFPSRILYGDFKQRYKVLNA**SVIPEGQFIDN**KKASEKL**LGSIDVNHDEY**RFGHTKVFFKAGLLGVLEEMRDEKLATLVTMTQALCRGYLMRREFVKMMERRESIFTIQYNIRSFMNVKHWPWMKVYYKIKPLLKSAETEKELANMKENYDKMKTDLATALAKKKDLEEKMVSLVQEKNDLQLQVASESENLSDAEERCEGLIKSKIQMEAKLKETSERLED**EEEINAELTAK**KRKLEDECSELKKDIDDLELTLAKVEKEKHATENKVKNLTEEM**ASQDESIAKLT**KEKKALQEAHQQTLDDLQAEEDKVNTLTKAKTKLEQQVDDLEGSLEQEKKLRMDLERAKRKLEGDLKLAQESIMDLENDKQQSDEKIKKKDFETSQLL**SKIEDEQSM**SAQLQKKIKELQARIEELEEEIEAERAARAKVEKQRADLSRELEEISERLEEAGGATAAQIEMNKKREAEFQKLRRDLEESTLQHEATAAGLRKKQADSVAELGEQIDNLQRVKQKLEKEKSEYKMEIDDLS**SNMEAVAKSKGNLE**KMCR**TLEDQFSELK**TKNDEHVRQINDISGQRARLL**TENGEFGRQIEE**KEALVSQLTRGKQAFTQQIEELKRQVEEEVKAKNALAHGVQSARHDCDLLREQF**EEEQEAKAELQ**RGMSKANSEVAQWRTKYETDAIQRTEELEEAKKKLAQRLQDAEEQIEAVNSKCASLEKTKQRLQSEVEDLMIDVERANGLAANLDKKQRNFDKVLAEWKQKYEEGQAELEGAQKEARSLSTELFKMKNSYEEALDHLETMKRENKNLQQEISDLTEQMGETGKSIHELEKSKKQVETEKTEIQTALEEAEGTLEHEESKILRVQLELNQIKGEVDRKLAEKDEEMEQIKRNSQRVIDSMQGTLDSEVRSRNDALRIKKKMEGDLNEMEIQLSHANRQASESQKQLR**NVQGQFKDAQLHLDD**AVRAQEDLKEQAAMVDRRNGLMVAEIEELRAALEQTERGRKVA**EQELVDASERVGLLHSQNTSLLN**TKKKLESDLVQVQSEVDDSIQEARNAEDKAKKAITDAAMMAEELKKEQDTSAHLERMKKNLEVAVKDLQHRL**DEAENLAMKGGKKQ**LQKLESRVRELETEVEAEQRRGADAVKGVRKYERRVKELTYQTEEDKKNVARLQDLVDKLQLKVKSYKRHAEE**AEEQANVHLS**KCRKIQHELEEAEERADIAESQVNKLRAKTRDSGKGKEAAE |
| TRINITY_DN54046_c11_g2::TRINITY_DN54046_c11_g2_i3::g.17254::m.17254 | gi\|657594936\|ref\|XP_008302105.1\| | PREDICTED: creatine kinase M-type-like [Stegastes partitus] | 95.3 | M**AKNCHNDYKMK**F**TTDDEYPDLSLHNNHMAKVLT**KDIYGKLR**GKSTPSGFTVDDVIQTGVDNPGHPFIMTVGCVAGDEESYEVFK**DLLDPIISDRHGGYKPTDKHKTDLN**FENLKGGDDLDPNYVLSSRVR**TGRSIKGFTLPPHNSRGERRAIQNLSIEAL**ASLEGEFK**GKYYPLDGMTDAEQEQLIADHF**LFDKPVSPLLT**CAGMARDWPDARGIWHNDNKTFLVWVNEEDHLRVISMQKGGNMKEVFRRFCVGLQKIEEIFKKHNHGFMW**NEHLGYILTCPSNLGTGLR**GGVHVKLPKLSTHAKFEEILTRLR**LQKRGTGGVDTASVGGVFDISNADRLGSSEVYQVQ**L**VVDGVKLMVE**MEKKLE**KGESIDGMIPAQK** |
| TRINITY_DN48833_c4_g9::TRINITY_DN48833_c4_g9_i1::g.72768::m.72768 | gi\|1552222\|dbj\|BAA13446.1\| | muscle actin OlMA1 [Oryzias latipes] | 98.7 | MC**DDEETTALVCDNGSGLVKAGFAGDDAPRAVFPSIVG**RPRHQGVM**VGMGQKDSYVGDEAQSKRGILT**LKYPIEHGIITNWDDMEKIWHHTFYNELR**VAPEEHPTLLTEAPLNPKANR**EKMTQIMFETFNVPAMYVAIQAVLSLYASGRTTGI**VLDAGDGVTHNVPVYE**GYALPHAIMRL**DLAGRDLTDY**LMKILTERGYSFVTTAEREIVRDIKEKLCYVALDFENE**MATAASSSSLEKS**YEL**PDGQVITIGNER**FRCPETLFQPSF**IGMESAGIHETAYN**SIMKCDIDIRKDLYANNVLSGGTTMYPGIADRMQKEITALAPSTMKIKIIAPPERKYSVWIGGSIL**ASLSTFQQM**WISKQEYD**EAGPSIVHR**KCF |
| TRINITY_DN54808_c0_g1::TRINITY_DN54808_c0_g1_i1::g.84158::m.84158 | gi\|657524690\|ref\|XP_008277999.1\| | PREDICTED: myosin-binding protein H-like isoform X1 [Stegastes partitus] | 98.2 | MPSKPAPIKKAAGKEPAKKEEKKA**PEPAPEPVPEPAPAEAAPAEAAPAEAAPAEGAPAEGAEGAPAPAEGEAAPAEDAAAAPPAE**EPKAPTPPPPDEPTSAPVELFVEDKNDTSVTIIWSQPETIGHSGLDGYTIEVCKDGTDDWKAVNEDLQKSCRYVIKNQTTGDRLKIRVVAVNAGGRSPPIALPEAVLVKEVADRPKVRLPRILKQRY**VAYVGDKINLT**IPFTGKPKPVVTWTKNGEPLDKKRVNIRSTDRDSILFIR**TSERDDSGVYEM**CVKVEDFEDKAPITLQIVELPGPPASVKIVDTWGFNVALEWTAPKDNGNTEITGYTVQKADKKTGDWFTVVEHYHRLNATISDLIMGNTYKFRVFSENKCGISESAAVSKEEAKILKTGIEYKPLEYKEHDFTEAPKFT**TSLTDRSTTVGYS**TKLLCSVRGFPKPKVIWMKNQMI**IGDDPKFR**QICVQGICSLEIRKPGNFDGGVYSCRAKNDHGEVTVSCK**LEVKQPAVADAEKK** |
| TRINITY_DN43137_c2_g2::TRINITY_DN43137_c2_g2_i1::g.71288::m.71288 | gi\|110808625\|gb\|ABG91151.1\| | skeletal muscle fast troponin T isoform 2 [Hippoglossus hippoglossus] | 91.6 | MSDTEEVD**AGAVEEVVVEEVEVAPEAAPEPEAEPEPEPEPEPEPEPEVEPEPEPEPEPEPEAEP**EPEAEEEKPKFKPSAPKI**PDGEKVDFDDIQK**KRQNKDLIE**LQALIDAHFE**CRKKEEEELIALKDRIEKRRAERAEQQRVRAEKDKERQARREEERRVR**EEADATKKAAEEAKKKSA**L**SSMGSSYSSHLQ**KADSKRGGKKETEREKKKKILAARRKQLN**IDHLNEDKLKDKINELH**DWMCQ**LESEKFDHMER**LKRQKYEVTTLRKRVEELSKFSKKGAAARRRK |
| TRINITY_DN40520_c0_g1::TRINITY_DN40520_c0_g1_i1::g.97405::m.97405 | gi\|974115284\|ref\|XP_015257043.1\| | PREDICTED: beta-enolase [Cyprinodon variegatus] | 96.1 | M**SITKIHAREILDSRGNPTVEVDL**WTAKGHFR**AAVPSGASTGVHEALE**LR**DGDKARYLG**KGVLKAVDHVNKDIAPKLLEKKFSVVDQEKIDKFM**LELDGTENKSKFGAN**AILGVSLAVCKAGAKEKGVPLYRHIADLAGHKDVILPVPAFNVINGGSHAGNKLAMQEF**MILPVGASNFHEA**MRIG**AEVYHNLKNVIKAKYG**KDATNVG**DEGGFAPNILENN**EALELLKTAIEKAGYPDKIIIGM**DVAASEFYRN**GKYDLDFKSPDDPARHISGEKLGDLYRSFIKGHPVQSIEDPFDQDDWEQWAKFTASTDIQIVGDDLTVTNPKRIQQAVDKKACNCLL**LKVNQIGSVTESIQACKLA**QSSGWG**VMVSHRSGETED**CFISD**LVVGLCTGQIKTG**APCRSERLAKYNQLMRIEEELGDKAKFAGKDFRHPKIH |
| TRINITY_DN13366_c0_g2::TRINITY_DN13366_c0_g2_i1::g.113635::m.113635 | gi\|77799798\|dbj\|BAE46762.1\| | white muscle parvalbumin [Trachurus japonicus] | 83.3 | GRSLQSTRDLSVSSVPYKAKTPKLTM**AFKGVLNDADVTAALDGCK**SAFDHKAFFKACGLAAKSADDIKKAF**AIIDQDKSGFIEED**ELKLFLQNFC**AGARALSDAETKAFLKAGDSDGDGKIGVDEFAAMVKH** |
| TRINITY_DN39861_c1_g1::TRINITY_DN39861_c1_g1_i1::g.104849::m.104849 | gi\|961866368\|ref\|XP_014893949.1\| | PREDICTED: triosephosphate isomerase [Poecilia latipinna] | 97.9 | MFINTPRYRFTSALLPQSRCTLVSCRYRFDSQQTHYPPKETATMSRKFFVGGNWKMNGDKKSLG**ELINTMNGAKVDPNVEVVCGAPTIYLDFVRSKLD**AK**FGVAAQNCYKVA**KGAFT**GEISPAMIH**DCGVKWVILGHSERRHVFGESDELIGQKTAHALENGLGVIACIGEKLDEREGGITEKVVFAQTKF**IADNVKDWSK**VVLAYEPVWAIGTGKTASPQQAQEVHEKLRGWMKTHVSEAVANSVR**IIYGGSVTGGTCK**ELA**SQKDVDGFLVGGASLKPEIIEIINAKA** |
| TRINITY_DN42486_c0_g1::TRINITY_DN42486_c0_g1_i1::g.80855::m.80855 | gi\|657590203\|ref\|XP_008299528.1\| | PREDICTED: sarcoplasmic/endoplasmic reticulum calcium ATPase 1 isoform X1 [Stegastes partitus] | 95.1 | **MDQAHTKNPAECLTY**F**GVNENTGLSPDQFK**KNLDKFGFNELPAEEGKSIWELIIEQFEDLLVRILLLAACISFVLAWFEEGEETVTAFVEPFVILLILIANAVVGVWQERNAEDAIEALKEYEPEMGKVYRSDRKSVQRIK**AREIVPGDIVE**VSVGDKVPADIRIVSIKSTTLRVDQSILTGESVSVIKHTEAVPDPRAVNQDKKNMLF**SGTNIAAGKAIGVA**VATGVSTEIGKIRDQMAATEQEKTPLQAKLDEFGEQLSKVITLICIAVWAINIGHFNDPVHGGSWIRGAVYYFKIAVALAVAAIPEGLPAVITTCLALGTRRMAKKNAIVRSLPSVETLGCT**SVICSDKTGTLT**TNQMCVTKMFIIKSVDGDHVDLDAFDISGSKYTPEGEVSQGGAKTNCSAYDGLVELSTICALCNDSSLDYNESKKIYEKVGEATETALCCLVEKMNVFNSNVKNLSKIERANACCAVIKQLMKKNFTLEFSRDRKSMSVYCTPGKGDGGAKMFVK**GAPEGVIER**CSYVRVGTSRVPLTGAIKEKIMAVIRDWGTGRDTLRCLALATCDSPLRPEEMNLEDSTKFANYETDLTFVGCVGMLDPPRKEVTGSIVLCREAGIRVIMITGDNKGTAIAICRRIGIFTEDEDVSGKAYTGREFD**DLPSHEQPEAVVR**ACCFARVEPSHKSKIVEFLQ**GHDDITAMTGDGVNDAPALK**KAEIGIAMGSGTAVAKSASEMVLADDNFSSIVAAVEEGRAIYNNMKQFIRYLISSNVGEVVCIFLTAALGLPEALIPVQLLWVNLVTDGLPATALGFNPPDLDIMGKPPRSPKEPLISGWLFFRYMAIGGYVGAATVGGAAWWFL**YDPTGPGVTYH**QLSHFMQCHDANEDFTGVECEIFEASPPMTMALSVLVTIEMCNALNSLSENQSLVRMPPWSNLWLAAAMTLSMSLHFMIIYVDPLPMIFKLTHLSVEQWMMVLKLSFPVILIDEVLKFFARNYVEK |
| TRINITY_DN41606_c6_g1::TRINITY_DN41606_c6_g1_i1::g.74538::m.74538 | gi\|281484920\|gb\|ADA70321.1\| | parvalbumin 3 [Siniperca chuatsi] | 100 | MAFAAVLKEAEMKAALDGCAAADSFDYKKFFKACGLAGKTAEDVKKAFLIIDQDNSGFIEEEELKLFLQNFSAGARALTDKETKA**FLAAGDSDGDGKIGIDEFAALVKA** |
| TRINITY_DN54121_c3_g1::TRINITY_DN54121_c3_g1_i1::g.17567::m.17567 | gi\|1007754500\|ref\|XP_015816723.1\| | PREDICTED: phosphoglycerate kinase 1 [Nothobranchius furzeri] | 92 | RPLGVHPCCTKVTAVTRSLTNRQPKEAEDPQRGRCRKETNEASNM**SLSNKLTLDKVDVK**GKRVIMRVDFNVPMKDKHITNNQRIKAAVPTIKHCLDHGAKSVVLMSHLGRPDGNFVPDKYSLEPVAAELKTLLGKEVTFMKDCVG**KEVEAACANPAAGSVIL**LENLRFHVAEEGKGKDASGNKTKATQEQIDSFRASLSKLGDVYVNDAFGTAHRAHSSMVG**VNLPQKAAG**FLMKKELDYFAMALEKPQKPFLAILGGAKVKDKIQLINNMLDKVNEMIIGGGMAFTFLKVLNNMEIGTSLY**DEEGAGIVK**DLMAKAEKNGVKITLPVDFITADKFDEKATTGTATVAAGIPAGWMGLDCGPESSKAFSEAVGRAKQIVWNGPVGVFEWEHFAKGTKNLMDKVVEVTKTGCITIIGGGDTATCCAKWNTEDKVS**HVSTGGGASLE**L**LEGKVLPGVDALSSA** |
| TRINITY_DN52604_c0_g1::TRINITY_DN52604_c0_g1_i1::g.58872::m.58872 | gi\|542200547\|ref\|XP_005474449.1\| | PREDICTED: myozenin-1-like [Oreochromis niloticus] | 88 | M**PLGTPAPVNK**RKKPSKIIT**DLSHITQDEYE**SEPEASEFDLGTKIRTPKDIMLEELSLMKNRGSKMFKMRQQRVEKFIHE**NNPDVFSSESMD**NLQQFMGGQMGGEMINLGGHFVSKQGGLNFIGLQTAGGAPVPPPKPGS**KGAGAGGAGGAGGAGGAGG**AGGVGGGEHGKGGHHGEEGWSPLKGGGTDDASKKHIHVKIYVSPWEKAMKGDEALIATLKGGMPGPAMHKDHPHYKSFNRTAMPFGGFEKAKQFLK**FQLPEKEETKEEPEPAVVYQHEIG**CRPSFNRTPIG**WVGSSEPSSIHMENDVVPFDGETDEL** |
| TRINITY_DN45055_c0_g1::TRINITY_DN45055_c0_g1_i1::g.50849::m.50849 | gi\|7678748\|dbj\|BAA95136.1\| | myosin light chain 3 [Trachurus trachurus] | 94.4 | M**AEEAAAAPAAASEFTADQMED**FKEAFGLF**DRVGDGQVAYNQVAD**IMRALGQNPGNKDVTKIL**GNPSADDMANKR**LAFDAFIPMLKQVDALQKGTYDDYVEGLRVFDKEGNGTVMGAELRIVLS**TLGEKMTEPEID**ALM**TGQEDENGSVHYEA**FVKHIMSV |
| TRINITY_DN53071_c2_g2::TRINITY_DN53071_c2_g2_i2::g.16098::m.16098 | gi\|657583015\|ref\|XP_008295593.1\| | PREDICTED: creatine kinase M-type [Stegastes partitus] | 94 | M**PFGNTHNNFK**LNYKVEEEFPDLSKH**NNHMAKVLT**KELYGKMR**DKQTPSGYTLDDVIQTGIDNPGHPFIMTVGCVAGDEESYEVFK**DLLDPVISDRHGGYKPTDKHKTDLN**FENLKGGDDLDPNYVLSSRVR**TGRSIKGFTLPPHNSRGERRHIEKLS**VEALTSLDGEFK**GKYYPLKSMTDAEQEQLIADHF**LFDKPVSPLLT**CAGMARDWPDGRGIMHNDNKTFLVWVNEEDHLRVISMQKGGNMREVFKRFCVGLQKIEAIFKKHNHGFMW**NEHLGYILTCPSNLGTGLR**GGVHVKLPKLSTHAKFEEILTRLR**LQKRGTGGVDTASVGGVFDISNADRLGSSEVDQVQLVVDGVKLMVE**MEKK |
| TRINITY_DN49088_c4_g3::TRINITY_DN49088_c4_g3_i2::g.119045::m.119045 | gi\|7678750\|dbj\|BAA95137.1\| | myosin light chain 2 [Trachurus trachurus] | 95.3 | MAPKKAKRRQAAGDGGSSNVFSM**FEQSQIQEYKEAFTIIDQNRDGIIS**KDDLRDVLASMGQLNVKNEELEAM**IKEASGPINF**TVFLTMFGEKLKGADPEDVILSAFK**VLDPEGTGSIKKE**FLQELLTTQCDRFTPEEI**KNMWSAFPPDVAGNVDYKNICYVITHGEEKEE** |
| TRINITY_DN49401_c0_g1::TRINITY_DN49401_c0_g1_i1::g.75819::m.75819 | gi\|744782311\|gb\|AJD07313.1\| | pyruvate kinase [Trachinotus ovatus] | 91.1 | M**SKSKDSIMH**SQQ**MHAATADTLIEH**MCL**LDIDSHPAVSRNTGIICT**IGPATRSVEKGKEMIKTGMNIARMNFSHGTHEYHAETIKNVRTATESFVPGSVEYRPVAIALDTKGPEIRTGLIK**GSGTAEVELK**KGETIKLTLDNAFMEKCDEKVLWLDYKNITKVVQAGSHIYIDDGLISLKVKEVGGDHVTCEIENGGTLGSKKGVNLPGAAVDLPAVSEKDIQDLQ**FGVEQGVDM**VFASFIRKAADVHAVRKVLGEKGKDIKIISKLENHEGVRRFDEIL**EASDGIMVARGDLG**IEIPTEKVFLAQKMMTGRCNRIGKPIVCATQMLESMTKKPRPTRAEASDVANAVLDGNDCIM**LSGETAKGDYPLE**AVRAQHLIAREAEAAMYHRQMFEELRRITPLTRDPTESVAIGAVEASFKCCASAIIVLTKTGRSAHLLSKYRPRAPIIAVTRCGQTARQAHLYRGVYPVLYTKAANDVWAEDVDLRVNFALEVGKHHKFFKSGDVAIVVSGWRPGAGYTNTMRVVLVP |
| TRINITY_DN52779_c3_g17::TRINITY_DN52779_c3_g17_i1::g.123787::m.123787 | gi\|295792256\|gb\|ADG29132.1\| | troponin I [Epinephelus coioides] | 98.3 | MSHDKKMTSSRKHHLKSLMLQIAAGWIEQEKKDIAAAKVAYL**AETCPKPTISGDQAALME**ICKKIHASLDKIDEARYDAEVKVKKADKEIDDLKMKVVELAGVKKPALKRVR**MSADAMLH**ALLGGKHKVTMDLRSNLKQVKKEVKEEATEAGDWRKNIEDKADRKKMFETS |
| TRINITY_DN44517_c0_g1::TRINITY_DN44517_c0_g1_i1::g.10473::m.10473 | gi\|348523353\|ref\|XP_003449188.1\| | PREDICTED: keratin, type II cytoskeletal 8-like [Oreochromis niloticus] | 88.9 | M**SSTYRTSSYSVK**SSNAPRSF**SSSSYAGPGGITSRK**SYSVRSSF**GGSNRGYGGAGITS**SSAYGLSSSAGMGMGMGMGMGMGMGGM**GAQAPITAVTVNKSL**LAPLNLE**IDPTIQAVRTQEKE**QIKTLNNRFASFIDKVRFLEQQNKMLETKWNLLQGQTTTRSNIDAMFEAYISNLRRQLDSLGNDKMKLEADLHNMQGLVEDFKNKYEDEINKRTECENDFVLIKKDVDEAYMNKVELEAKLESLTDEINFLRSIYEEELRELQSQIKDTSVIVEMDNSRNLD**MDSIVAEVK**AQYEDIANRTRAEAETWYKTKYEEMQTSANRYGDDLRSTRTEITDLNRMIQRLTSEIDAVKGQRANLEAQIAEAEERGELAVKDAKLRIKDLEDALQRAKQDMARQIREYQDLMNVKLALDIEIATYRKLLEGEEDRLANGIKAINISQQSTSYSGFPM**DSMKSSYASTYS**SGYGSGYGSGYGSSSGGFSGGSAGGYGSAGGYGSAGGYSTTTQSKKNVVIKMIE**TKDGRVVSESSEVIED** |
| TRINITY_DN49882_c0_g1::TRINITY_DN49882_c0_g1_i1::g.120111::m.120111 | gi\|958229780\|gb\|JAO32154.1\| | PYGM [Poeciliopsis prolifica] | 77.9 | M**SKPLSDHDRKKQIS**VR**GLAGVENVAELKQNFN**RHLHFTLVKDRNVATKRDYYFALAHTVRDHLMGRWIRTQQHYYEKDPKRVYYISLEFYMGRTLQNTMVNLALENACDEATYQLGLDMEELEDMEEDAGLGNGGLGRLAACFLDSMASLGLAAYGYGIRYEFGIFNQKIVNGWQVEEADDWLRYGNPWEKARPEYMRPVHFYGRTEHHPDGVKWVDSQVVLALPYDSPIPGYRNNIVNTMRLWSAKAPCDFNLKDFNVGGYIQAVLDRNLAENISRVLYPNDNFFEGKELRLKQEYFVVSATLQDIIRRFKVSKFGSRDVVRTDFNKLPEKVAIQLNDTHPAMAIPELMRVLVDEEKLLWESAWDICIRTCAYTNHTVLPEALERWPVDLFAHLLPRHLEIVYEINRRHLEKVAAKFPGDHDRMRRMS**LIEEGGQKRIN**MAHLC**IVGSHAVNGVA**QIHSDILKATIFKDFYEMEPHKFQNKTNGITPRRWLVMCNPGLAEIIAEKIGEDFIRDLDQLKGLRKFINDEAFIRDIAKVKQENKLKFSVHLEEHYKVKINPNSMFDIQVKRIHEYKRQLLNCLHIITYYNRIKKEPNKQWTPRTVMIGGKAAPGYHTAKMIIRLITAIGEVVNNDPVIGDRLKVIFLENYRVTLAEKAVPAADLSEQISTAGTEASGTGNMKFMLNGALTIGTMDGANVEMAEEAGMDNIFIFGMRVDDVDAMDRKGYHASEYYNRLPELKQAIDQIAGGFFSPNQHDLFKEIVNMLMHHDRFKVFADYEDYIKCQEKVNALYKNPKEWTKKAIHNIAGCGKFSSDRTIAQYAREIW**GMEPTLEKLPAPDDKH** |
| TRINITY_DN49369_c0_g1::TRINITY_DN49369_c0_g1_i1::g.75809::m.75809 | gi\|734608233\|ref\|XP_010731357.1\| | PREDICTED: myosin-binding protein C, fast-type-like isoform X5 [Larimichthys crocea] | 85.9 | M**PEPVPAAKPEGDDAPPPDAVEAN**HAEEEQGSTELTGLFLEKPPESVVAVTGSDVTFIAKVDSTTLTRKPVMKWLKGKWLDLGSKAGKHMQFKETYDRNTKVYTYEMKIIKVVPGDAGGYRCEVTSKDKCDSSTFEISVEAAHAEEHTDILSAFKRADAGEDEGDLDFSALLKATKKKKKPQKVEPEVDVWELLKSAHPSQYEKIAFEYGITDLRGMLKRLKKMKTVVPKHTEAFLKKLDDCYSVEKGKKIVLKCEVVDPNAQVKWLKNGQEIKPSAKYVIEAVGNVRTLTINKTTLADDAAYECVIGEDKSFTEVFVKEPPVTITKLMDDYHVVVGERVEFEIEVSEEGAHVMWFFEDIELHRDKDTKYRFKKDGKKHTLIIQEATLDDIGMYHAWTNGGHTKGELEVEEKELEVLQDIADLTVKATDQAMFKCEVSDDKVTGKWFKDGVEVLPSDRIKMSHIGRFHRLTIDDVKPEDAGDYTFIPDGYALSLSAKLNFLEIKIDYVPRQDPPKIHLDTTGSMVSQNTIIVVAGNKLRLDVEITGEPAPTVVWSKGDQPITETEGRVRVEARKDLSCFVIEGAEREDEGKYTIVVTNPAGEDKAMLFVKIVDVPDPPEHVKCTGVGEDCATITWDPPKFDGGAAIKGYLMERKKKGSSRWTKLNFDVYESTTYEAKRMIEGVLYEMRVFAVNSIGMSQPSLNSKPFMPIAPTSEPMRLTVHDVTDSTCSLKWLAPEKVGAGGLDGYVIEYCKEGDTEWVVANKDICERQGYVVRGLPVGEKINFRVVAVNIAGRSPPATLGQPVTIREIVEHPKIRLPRDLRTKYIRKVGEKINLTIPFQGKPRPVATWYKDGQPLDPKMVNVRSSNVDTILFIRSAEREHSGTYELVLQIENLEDRASINIRIIDKPGPPLNVRVTEVWGFNAALEWEPPKDDGNCDISGYTIQKSELKTKEWFTVYEHNRRTNCTASDLIMGNEYQFRVYSENLCGKSEEPRFSKNTATIVKTGLDMKANPYKEKDMNCMPKFTQPLVDRSVVAGYSTAITCSVKGFPKPKITWMKNKMIIGDDPKFLMQNNQGVLTLNIRKPSPFDGGKYSCM**AVNDLGKDEVE**CK**LDVRVATNPDKQ** |
| TRINITY_DN47596_c0_g1::TRINITY_DN47596_c0_g1_i1::g.69150::m.69150 | gi\|657537490\|ref\|XP_008275186.1\| | PREDICTED: malate dehydrogenase, mitochondrial [Stegastes partitus] | 95 | MFSRAVRPTVSLVRSLSTSSQNN**AKVAVLGASGGIGQPLSLLLKNSPLVSQ**LSLYDIAHTPGVAADLSHIETRAQVTGYMGADQLDAALKGCEVVVIPAGVPRKPGMTRDDLFNTNATIVA**ILADACARNCPEA**MICIIANPVNSTIPITSEVMKKHGVYNPNKVFGVTTLDIVRANAFVADLKGLDPARVNVPVIGGHAGKTIIPLISQCTPKVEFPADQLSALTGRIQEAGTEVVKAKAGAGSATLSMAYAGARFTFSVLDAMNGKEGVVECAYVRSEETECKYFSTPLLLGKNGIEKNLGLGKLSAFEEKLVADAIDELKGSIKKGEDFVAKMK |
| TRINITY_DN87263_c0_g1::TRINITY_DN87263_c0_g1_i1::g.49173::m.49173 | gi\|1007771873\|ref\|XP_015824097.1\| | PREDICTED: keratin, type II cytoskeletal cochleal-like [Nothobranchius furzeri] | 90.3 | GLDSPLCVLCCSARTLSLPPFLSSQKKTKPAFATMRSSMRSTRTSFTSSGGSSGGFSGGSRISMGGGGGGAVKSFSSR**SAIVSGPRISSASM**MRSSMGGGGGGAGFGMGGGFGLGGGGGGGGFSMSSYSSGGGFGGGAGGFGMGGGM**AIAPITNVQVNQSLL**APLNLDIDPTIQTVRTQEKEQIKTLNNRFASFIDKVRFLEQQNKMLETKWSLLQDQTTTRSNIDG |
| TRINITY_DN27846_c0_g3::TRINITY_DN27846_c0_g3_i1::g.118352::m.118352 | gi\|269306947\|gb\|ACZ34179.1\| | glyceraldehyde-3-phosphate dehydrogenase [Rachycentron canadum] | 91.6 | M**VKVGINGFG**RIGRLVTRAAFHSKKVEIVAINDPFIDLEYMVYMFKYDSTHGRFHGEVKVEGDKLVIDGHKITVFHERDPTQIKWG**DAGAHYVVESTG**VFTTIEKASAHLKGGAKKVIISAPSADAPMFVMGVNHEKYDKSLQVVSNASCTTNCLAPLAKVINDNFHIVEGLMSTVHAITATQKTVDGPSGKLWR**DGRGASQNIIPASTGAAKAVG**KVIPELNGKLTGMAFRVPTPNVSVVDLTVRLEKPAKYDDIKKVVKAAAEGPMKGILGYTEHQVVS**TDFNGDTHSSIFD**AGAGIALNDHFVKLVTWYDNEFGYSTRVCDL**MVHMASKE** |
| TRINITY_DN43545_c1_g1::TRINITY_DN43545_c1_g1_i1::g.104599::m.104599 | gi\|908482009\|ref\|XP_013124986.1\| | PREDICTED: LIM domain-binding protein 3-like isoform X3 [Oreochromis niloticus] | 84 | MSTYTVTLNGPAPWGFRLQGGKDFNMPLTISRITPASKAAGGSLTQGDIISAIDGASTEGMTHLEAQNKIKSVTSKLVLTMQKSRRPAPVPTATPRMDSPMPVIPHQKVIANTAANVEYTPSFNPIALKDSALSTHKPIEVRGPGGKATIVHAQYNTPISMYSQDAIMDAIAGQSQAKGHDS**SSVLPVKDPVVD**CA**SPVYQAVIRPGGNQDM**SEWARRAANLQSKSFRTL**AHITGTEYMQDPDEEALQR**SREKFESEVKGPRFAKLKNWHHGLS**AQILNIQE** |
| TRINITY_DN56725_c6_g7::TRINITY_DN56725_c6_g7_i1::g.108808::m.108808 | gi\|499031057\|ref\|XP_004566239.1\| | PREDICTED: troponin I, fast skeletal muscle-like [Maylandia zebra] | 88.6 | MSEKKMTSSRRHHLKSVILQIALSW**LEQEKKDLVVAKQNY**MAE**HCPSPDASGDQAALMEL**CKKIHGIIDKIDEERYDAESKVQKADKEIEDLKIKVVDLIGVKKPALKKVRMSADTMLQALLGSKHKVTMDL |
| TRINITY_DN56777_c0_g3::TRINITY_DN56777_c0_g3_i5::g.108945::m.108945 | gi\|808859815\|gb\|KKF11904.1\| | Nebulin [Larimichthys crocea] | 76.8 | TPVDMMSVVQAKHASTVQTYAGYRKIPHNYF**LLPDNLH**LQQCRNMNTIASDCDYKSDWNNCVRGLGWVPMGSIGVETAKLGGDILSEHKYRTHPSNFKYSKLMDSMDLTLATANNKIMNKQAYTSAWDKDKLTVHIMPDAPEIL**LAKANAINMSN**KLYKAGLVEMANKGHHLKADAIPILAAKSGTTIASDYKYKLAYEKAKGHHVGFR**SLQDDPLLVH**YMQVARLQSDKNYKKDYHKAKLKYHSPVDMMSMTHAKHASAVQTYAGYKQVTSRYTVLPDAMNLELARHMQIIASDNQYKSDYNNYVRGTGWVPIGSVDVELAKAAGQALSEPRYRQHPSKFNFTKDMHSMDLTLATANNHIMDKQAYFKAWENDKTSIHIMPDAMEIVLARDNKSRYSEKLYRLDNELAKKKGHHVRADAIAIQAAKACTAIASDYKYKTGYRKQVGHHIGARCIQDDPLLML**ALNSAKIASDAL**YKKDFNKSKTKFHLPVDMISFELAKKNQIQVNHANYITRLHNWTCL |
| TRINITY_DN58690_c14_g50::TRINITY_DN58690_c14_g50_i3::g.63723::m.63723 | gi\|768960659\|ref\|XP_011618511.1\| | PREDICTED: LOW QUALITY PROTEIN: uncharacterized protein LOC101065560 [Takifugu rubripes] | 88.3 | M**ADQVAKTTQSLTTAAQMRETT**MMLMQPYANWEEYLTPAPLSVAIMGEIVFISSVQDFSINKNAPKDGFKYIRYPDSFRACLMQICNSGWHAYNEAHKNMDKIRLRTATVPDHMKAVINTLFNGSDEVIENLLPNQLENIRVIADDCVALADGVEKKYSDVINLIQELLEACVNAQHFYGEELENVKMKLEESKLREKTAQLLNERSKKALEDMGKQLEETQATFKQSMDSLPSGWEMIGMDFVEGLSTAVTGILNVVTLPLKLISTIKESVTDQQSSGEINENVDEFTQMTVNSKSGEILTLVGTLKHHVSGNEIDWKNLYDQEKKCPKTNWTESQFKRIFTEVGKLPGGKASEEVLSICQKGINICKELATYTPDQKWDEATTKQLINKITELNDEALAFDSRSKKALGTPAFTPKPPMMYKEQSKSSESTSASQRAAENARFKIEQSREQLKQTREQYEKSVENMEKNEKELTDILVEMQNCKIKEIDFETTIKMLVKGLDAMGRVKEQ |
| TRINITY_DN51492_c0_g6::TRINITY_DN51492_c0_g6_i1::g.126915::m.126915 | gi\|808882105\|gb\|KKF29537.1\| | Myosin heavy chain, fast skeletal muscle [Larimichthys crocea] | 89.7 | M**STDAEMEQYGPAAIY**LRKPERERIEAQNTPFDAKTAFFVIDTEEMYVKGKLTKKEGGKATVETAGGKTVTVKEDDIHPRNPPKFDKIEDMAMMTHLNEPCVLYNLKDRYASWMLYTYSGLFCVVVNPYKWLPVYDSVVVNAYRGKKRIEAPPHIFSISDNAYQSMLTDRENQSIL**ITGESGAGKTVNT**KRVIQYFATIAVVGGKKDPVPGKIQGSLE**DQIIAANPLLEA**YGNAKTIRNDNP |
| TRINITY_DN48706_c0_g1::TRINITY_DN48706_c0_g1_i1::g.37279::m.37279 | gi\|734608349\|ref\|XP_010731423.1\| | PREDICTED: glucose-6-phosphate isomerase [Larimichthys crocea] | 83.2 | M**GLTQDPNFQKLQD**W**YTAHALNLN**MRHMFETDKERFNKLSLDLKTEDGDILLDYSKNLITEDVMKMLVDLAKSRGVEAAREKMFKGDKINFTEGRAVLHVALRNRSNTPIMVDGKDVMPDVNKVLEKMKGFCHRVRSGEWKGYTGKAITDVVNVGIGGSDLGPLMVTEALKPYSKGGPRVWFVSNIDGTHIAKTLAHLNAETTLFIVASKTFTTQETITNAESAKAWFLEHAKDKAAVAKHFVALSTNGPKVKDFGIDTENMFEFWDWVGGRFSLWSAIGMSIALHIGFDNFEKLLSGAHWMDNHFRTAPLDKNAPVLLALLGIWYINFFHAETHAMLPYDQYMHRFTAYFQQGDMESNGKYITNHGARVNYHTGPIVW**GEPGTNGQHAFYQ**LIHQGTRMVPSDFLIPAQTQHPIRENLHHKILLANFLAQTEAMMKGKTTEEAKKELEASGLSGEALEKILPHKVFQGNRPTNSIIFKKLTPHTLGALIAMYEHKIFIQGVMWEINSFDQWGVELGKQLAKKIEPELKDTKEVH**SHDSSTNGLINF**LKKNFA |
| TRINITY_DN31230_c0_g1::TRINITY_DN31230_c0_g1_i1::g.51888::m.51888 | gi\|222087993\|gb\|ACM41859.1\| | PDZ and LIM domain 7 [Epinephelus coioides] | 84.8 | M**SGEHTVGQEAMNVYS**ITLAGPAPWGFRLQGGKDFSMPLTVSRLTPGGKAAQAGVGVGDWVVSIGDTNAEDMTHVEAQNKIRAATDSLTLTLSKAFKTGGDQKMEALDKGEATYFDEMYLSKHGLRAENMACFIPNDRSKKRLIE**DTEDWQPRTGTT**QSRSFRIL**AQLTGTDF**MQDPDDENMKRAREKFLTEIQSPRYARLRDWHHDRSARALNIKS |
| RRRRRTRINITY_DN55603_c0_g1::TRINITY_DN55603_c0_g1_i2::g.85642::m.85642 | NA | NA | 21 | AVAPEQGGAADGRTRTHLLVAQGALGAEVGGEPVVYRTGDDARQPGMTRLQENSQAVCDLSWLQYRLSKGQKALIHVERLGPVNVPCSCSTGRPLHLWPLGPDPECQNESVSGQDLGLLVGSAAAWHARHGSRQCPEHNYDEDYPILVPQGGWLHRIITAANNGSLGGSLLEAKCGCVEEALLAMYNASFMEGQATYGRSLATQVVTEMDISLQPQRLHAAMWLAVLGCQPGDQILSPMYTNVLLWQLDKRFSSDRQGLLLRAETHDGEVPTSSSAITQYLKKKRDSS**APPPPPPPPP**GPAPP**PPPPAPPPPPPP**SSLPCQVSM |
| TRINITY_DN45055_c2_g5::TRINITY_DN45055_c2_g5_i1::g.50857::m.50857 | gi\|7678746\|dbj\|BAA95135.1\| | myosin light chain 1 [Trachurus trachurus] | 92.5 | RHIHTHTPLPSAIQSLPGNSVDGGQEENSLIYIRPNGDFLLRVPSTLCYTPCVDTRTGNCLCFRNTVTLLPAEMAPKKDAKAPAKKA**EPAPAPAPAPEPAPVPA**APAAVDLSAVKIEFSPDQVEDYKEAFGLF**DRVGDNKVAYNQIAD**IMRALGQNPTNKEVAKMLGTPSAEDMANKRVEFEGFLPML**QTIINSPNKAGYED**YVEGLRVFDKEGNGTVMGAELRIVLSTLGEKMTEAEIDA**LMTGQEDESGGVNYEA**FVKHIMSV |
| TRINITY_DN58073_c4_g2::TRINITY_DN58073_c4_g2_i1::g.89483::m.89483 | gi\|734626895\|ref\|XP_010741611.1\| | PREDICTED: calpastatin isoform X10 [Larimichthys crocea] | 88.4 | ESPKLRPEDIVSEGKHKKEKGVFVGEREDTLPPEYRFNKEELMKLDPPKPEPSMGTGEALDLLSGDF**MSSSKAPAVQ**APVVTPSAPPAQSKVEDLSALDLLAGDFVASTKASGVHAPIPPPTKKVPERTVCPLEKANTIDVISFKPEERPSLKKGDSLSGDALSALGDMLPAALPKPESPKLRPEDIVSEGKHKREKGVFVGEREDTLPPEYRFNKEELMKLDPPKPEPTMDTGDALDILSGDFMTSSKAPAVKAPVVTPSAPPAQSSADFALDAL**AGDFVSSSAAPTVK**SAAFVPTETQPELEAGADNALDALSDTLKDIAPAPQPVPVVPAKDMVKEKKIAEERLIKMGERDDSLPPEYQPTEEDLKKMAEAKAKAAAAPKEKTMDDLTALDLLSSDFSAAPKPAAPAASSAATTKLQPPVLDSEPLKPMAGPALDSLSGTLLPDAIKSKSDKSKGKSKSKSKSKKHHADEPSAADQLSAQLSSDVVPTSTKKGGKS |
| TRINITY_DN33102_c0_g1::TRINITY_DN33102_c0_g1_i1::g.53609::m.53609 | gi\|30349202\|gb\|AAP22041.1\| | keratin 8, partial [Oreochromis mossambicus] | 93 | QNEIEAVKGQRANLEAQIAEAEERGEMAVKDAKLRIKDLEEALQRAKQDMARQVREYQELMNVKLALDIEIATYKKLLEGEESRIASGGSAATIHVQSSSSGFAAGSGGGMGMGGGMGGGFGGGMGGGYGSGMSMSMGGGMGMSSMSGGMSGL**SIGGGSSSMTKSSSIR**RF |
| TRINITY_DN54046_c11_g2::TRINITY_DN54046_c11_g2_i7::g.17261::m.17261 | gi\|499038049\|ref\|XP_004569670.1\| | PREDICTED: creatine kinase M-type [Maylandia zebra] | 96.6 | M**AKNCHNDYKMK**F**TTDDEYPDLSLHNNHMAKVLT**KDIYGKLR**GKSTPSGFTVDDVIQTGVDNPGHPFIMTVGCVAGDEESYEVFK**DLLDPIISDRHGGYKPTDKHKTDLN**FENLKGGDDLDPNYVLSSRVR**TGRSIKGFTLPPHNSRGERRAIQNLSIEAL**ASLEGEFK**GKYYPLDGMTDAEQEQLIADHF**LFDKPVSPLLT**CAGMARDWPDARGIWHNDNKTFLVWVNEEDHLRVISMQKGGNMKEVFRRFCVGLQKIEEIFKKHNHGFMW**NEHLGYILTCPSNLGTGLR**GGVHVKLPKLSTHAKFEEILTRLR**LQKRGTGGVDTASVGGVFDISNADRLGSSEVDQVQLVVDGVKLMVE**MEKKLE**KGEAIDSMIPAQK** |
| TRINITY_DN57016_c1_g19::TRINITY_DN57016_c1_g19_i3::g.105518::m.105518 | gi\|1007785761\|ref\|XP_015829638.1\| | PREDICTED: elongation factor 1-alpha [Nothobranchius furzeri] | 82.8 | KYAWVLDKLKAERERGITIDIALWKFETQRYYVTIIDAPGHRDFIKNMITGTSQADCAVLIVAAGVGEFEAGISKNGQTREHALLAYTLGVKQLIVGVNKMDSTEPPYSQKRFEEITKEVSAYIKKIGYNPATVAFVPISGWHGDNMLEASDKMGWFKGWKIERKEGGATGTTLLEALDSILPPSRPTDKPLRLPLQDVYKIGGIGTVPVGRVETGVLKPGMVVTFAPPNLTTEVKSVEMHHESLPEAVPGDNVGFNVKNVSVKEIRRGNVAGDSKN**DPPMAADNFTAQVIILNHPGQISQ**GYAPVLDCHTAHIACKFSELKEKIDRRSGKKLEDNPKALKSGDAAIINMVPGKPMCVESFSQYPPLGRFAVRDMRQTVAVGVIKSVEKKNPSGGKVTKSAQKAEKKK |
| TRINITY_DN56391_c1_g18::TRINITY_DN56391_c1_g18_i2::g.87075::m.87075 | gi\|734647473\|ref\|XP_010752926.1\| | PREDICTED: muscle-related coiled-coil protein-like [Larimichthys crocea] | 89.9 | M**TDKSGMPTGGGDDAGSIMAL**LERVAGLMDSVQTTQQRMEERQLELESTVKTIQADVVKLTSDHANTSTTVDRLLEKTRKVSRHIKDVRVRVENQNVRVKKVEATQGDLLAKNKFRVVIYQGDQEVKAVTPGNEPAEPSGSGAARAEVEPDKFELPPESDEEYMVVEEADSSAAGRVKKSGLTRIESFKATFSKQNMSKTRENLGTKVNKLGERIVTAERREKIRQSGERLKQSGERLKETITKNVPAKLKKERTVAEGQEGAEGASEGAVPVPPPKGRKGSPGAAMAADDGKAEESEVPMYDMKQLS |
| TRINITY_DN52352_c2_g1::TRINITY_DN52352_c2_g1_i1::g.38561::m.38561 | gi\|339896195\|gb\|AEK21799.1\| | tropomyosin [Siniperca chuatsi] | 86.3 | **MDAIKKKMQ**MLKLDKENALDRAEQAESDKKAAEDRSKQLEDDLVALQKKLKGTEDELDKYSEALKDAQEKLELAEKKATDAEGDVASLNRRIQLVEEELDRAQERLATALTKLEEAEKAADESERGMKVIENRAMKDEEKMELQEIQLKEAKHIAEEADRKYEEVARKLVIIESDLERTEERAELSEGKCSE**LEEELKTVTN**NLKSLEAQAEKYSQKEDKYEEEIKVLTDKLKEAETRAEFAERSVAKLEKTIDDLEDELYAQKLKYKAISEELDHALNDMTSI |
| TRINITY_DN52976_c0_g1::TRINITY_DN52976_c0_g1_i1::g.15659::m.15659 | gi\|574588302\|gb\|AHG32674.1\| | SET and MYND domain containing protein 1a [Siniperca chuatsi] | 56.4 | MTVGNMEAAALFDAGKKGRGLRANRDLKAGEVVFAEPSFSAVVFDSLATQVCHNCFRHQSNLHRCAQCKFAHYCDRTCQTACWDEHKRECAAIKKLVMAPSEHVRLAARVLWRIHKATGIASDSQLISVDQLEDHVSDLSEEELKKLQGDVHKFQEYWSCGRKQHSAEYISHIFGIIKCNGFTLSDQRGLQAVGVGLFPNLCLVNHDCWPNCTVILNHGNQTALSSALHSKRRIELRALEKIPEGAELTVSYVDFLNLSEDRQKKLKEHFHFDCTCDHCSQHIKDDLMMAAAESKPSADKVKEVTAFSKESLEKIETCRVEGNFQEVVKLCHECLAKQENVLADTHLYRLRVLSVASEVLSYMRKFSEAAGYAQKMVEGYIKLYHHNNAQLGMAIMRAGVTHWHAGQIEVGHGLICKAYGILIVTHGPNHSITKDLETMRAQTEVELKMYRKNKEEYHTMR**EAALLKPVTSS** |
| TRINITY_DN43965_c1_g1::TRINITY_DN43965_c1_g1_i1::g.101508::m.101508 | gi\|831567189\|ref\|XP_012733626.1\| | PREDICTED: ATP-dependent 6-phosphofructokinase, muscle type-like [Fundulus heteroclitus] | 78.2 | M**AEPHQVDPTTMGKGRAIAV**LTSGGDAQGMNAAVRATVRVGIYTGAKVYFVYEGYQGLVDGGDHIRPATWESVSMMLQLGGTVIGSARCQDFRTKEGRTKAACNLVKLGITNLCVIGGDGSLTGANQFRTEWNELLADLVRTGKITANEAKASSHLNIVGMVGSIDNDFCGTDMTIGCDSALHRIIEIVDAITTTAQSHQRTFILEVMGRHCGYLALVTALACGADWVFIPEVPPEDGWEEHLCRRLQEQRGRGSRLNIIIVAEGAMNRAGKPITCEDIKALVSKKLGFDTRTTILGHVQRGGTPSAFDRILASRMGVEAVMAL**LEATPETPACVVSLSG**NMAVRLPLMECVQVTKDVTVAMGEGRYEEAVKLRGKSFENNWNTYKMLAHVRPPDTKSNINIAILNVGAPCAGMNAAVRSAVRIGLLQGHQMLAVHDGFDGLAHGQIEPIGWSGVAGWTGKGGSLLGTKRSLPSECMEEISLSIAKFNIHALIIIGGFEAFIGGLEMVQAREKYEEVCIPIVVIPATVSNNVPGSDFSVGADTALNTITMTCDRIKQSAAGTKRRVFIVETMGGYCGYLATMAGLASGADAAYIYEDHFNIHDLEMNVEHLVEKMKTTVKRGLILRNERCNANYTTDFIFNLYSEEGKGVFDCRKNVLGHMQQGGTPSPFDRNFGTKMGIKSVLWLTDKLKDCYRHGRIFANSPDTACVLGMKKRALVFTSLEELKSETDFEHRIPKVQWWLRLVPILKILAKYKTALDTSEKAAMEHVIKKRGLVHQ |
| TRINITY_DN24229_c0_g1::TRINITY_DN24229_c0_g1_i1::g.120164::m.120164 | gi\|961876346\|ref\|XP_014896596.1\| | PREDICTED: keratin, type I cytoskeletal 18 [Poecilia latipinna] | 87.7 | MSYRRTVQQIPSSHISMTRSAPQYRAASIYGGAGGHGARISSVSSSSLR**SGAPMGSSSSSFKLS**SAL**DGGMGMGMGGRVS**MAAGGGSSSGGGGGAGILGNEKGAMQNLNDRLANYLETVRHLEKANGELE |
| TRINITY_DN50036_c0_g1::TRINITY_DN50036_c0_g1_i1::g.73177::m.73177 | gi\|780956767\|gb\|AJZ70420.1\| | peroxiredoxin 5 [Oplegnathus fasciatus] | 86.4 | MLCITSASLIKSTRIVQCARLLHISPVVKM**PIQAGERLPAVEVQ**EGEPGNKVAMDQLFKGKKGVLFAVPGAFTPGCSKTHLPGFVQQAGDLKSKGIQEVACVSVNDAFVMAAWGKEHGADGKVRMLADPTGAFTKAVDLLLDNDQIVQVLGNKRSKRYSMLVEDGVVKKINVEPDGTGLTCSLATNILSEL |
| TRINITY_DN55301_c0_g2::TRINITY_DN55301_c0_g2_i1::g.84690::m.84690 | gi\|1012734441\|gb\|JAR28267.1\| | Nebulin [Fundulus heteroclitus] | 77.6 | DPLLML**ALNSAKIASDAL**YKKDFNKSKTKFHLPVDMIAFELAKKCQIQVNDDRYRTRLHQWTCMPDSNDVIQARKAYDLQSDNVYKADLEWLRGCGWVAADSVDHVKVRKAQQIINERLYKKDAQDAFGKFTL**VVDRPEIL**LAKQNAANLSDLKYKETFNAEKGHYIGSEDTPQMAHSREVSKNVSEKLYKLDWDETKATGYQLDHEYIPLIMGRKGRDIASDAKYKDAHEKAKGHHMAGTLVDFPDVMRCGEQEKNKGLRLYTKDYHQTKTKTHLPPDVIANQVAKRCQDMLSDVLYRTYLHQWTCHPDQEDAIRARKTNEILSDVYYKEDLNWMRGIGCYAWDTPEIIRAKKSYELQSELKYRDEAKKEFNNYSIVTDTPVYVTAVLGHTWASELNYREAYHKEKHLYTTVLDTYDYARCHNFKYFFSNKNYTSAWDKIKAKGYQIPHDSNAMQHAKQQKVVLSNVKYKEDYEKFKSLYSLPKSLEDDPQTARCVKAGKLVLDRLYRGDYEKNKAKNHIPPDMLEIMSNRNTQSMVSGIHYRKYLHQWICLPDMQVYVQARKVNEQLSDIFYKDDLNWLRGIGCYAWDTPEILRVKHAGDLQSENKYRAKGIESFKEYSVVTDTPVYETAKQNAQNLSDLNYRYDYNVNVKGTNTAPAVTIDTERARLGNYIQSDNYYKEANKSLMPTGYSLPHDTPLIKQVKHNSIVASNVKYKEAYEMTKAKAYTLHPEGVNFVTSRKANKMINERLYRQLYHKQKDKIHTTYDTPDIRQVKMNQEHLSDLCYKEKFYNSRGQLISLPITPELLHCYHVNEITSELKYKEDLMWLRGVGCFLYDTPEMVHVRNITKQRVTYPVEAKKNLANYSVVLDTPEYKRVTELKTHMSNLIYKAHSKEEMSKVTSTLDSLDIKRVKWAQQLTNKYMYTDLAAKERAHFTPEIDTPNMGHARIMKVVYSDKKYKDQYEKMKHRYT**AIADTPILVR**SKKAYLQSSDLRYKETFELSKGHYHTVKDALDIVCHRRVTDDISEVKYREKYINSLGTWKSIPDRPEFFFSKIANDQISNIKYKEDLDWLKGIGCYVWDTPELVQAERNKTLYSERLYKASFEKNRGNFKYTCDTPFFEAVKNASVLINDRAYRANYEKSRSKFT**ITTDDPRYQLA**RANRKMSQWKYREQYERAKDKFTSILETPEYEAHKRSKKISDIIYRMEYNKTKAKGYTLPYDTPYQQHMKKVKDITSTLKYKEVYEKSKAQINIDPEARDIRAAKEAYKNISNLDYKKKYEATKNKWIWTTDRPDFLNHAKNALQQSDVEYKYDKEMMKGCVIPVVDDKLTVLCMKNAEMASDVKYKLKYEQNKGHYVPVMDTPQILHAKAVRVLASESKYKEASKKEMQSGSFTTLSQTRDTAHSKEVNKLVSGKLYKAKFEKEKGKSKYNNMIVPPDVQHAIDVAKAQSSIAYKKDAKAGLHYTTVADRPDIRKAAQASKLISNIGYRDKAREEASRGGSLAHRPDIALATEVSKLTSELKYKEKFDKEMKGKRPKYDLKESKIYKTMKEANDLASEVKYKGDLKKIHKPVTDMAESLSMQHSLSTSKLSSQVKYKEKYEREKGKPMLDFETPTYVTAKEAQHMQSQREYKKSLEQEIKGKGMLALATDTPDFMRARNATDILSQTKYKQTAEMDRASYTTVIDTPDIIHAQQMKNIVSQKKYREEAEKTMSHYVPVLDTPEMQRVRENQRNFSTVLYSDSFRKQVQGKAAFVLDTPEMRRVKETQRIISGVRYHEDFEKSKGSFTPTTSDPVTERVKKNTQDFSDINYRGIQRRVVEMERRRAIEHDQETITDLRVWRTNPGSVFDYDPAEDNIQSRSLHMMSVQAQRRSKEHSRSTSALSGMADEKSEQSQDADHHMSLYSNGFMTSSIGYQHAKTVELQQRSSSVATQQTTVSSIPSHPSTTGKTVRAMYDYGAADNDEVSFKDGDVIVNVQAIDEGWMYGTVQRTGKTGMLPANYVEAI |
| TRINITY_DN48282_c1_g6::TRINITY_DN48282_c1_g6_i1::g.55915::m.55915 | gi\|734625726\|ref\|XP_010740972.1\| | PREDICTED: guanidinoacetate N-methyltransferase [Larimichthys crocea] | 77 | M**TTAAQPIFSKGEDCK**ASWHDASAGYNETDTHLEIMGKPVMERWETPYMHSLSTVAASKGGRVLEIGFGMAIAATKIESFPIEEHWIIECNDGVFARLENWAKAQPHKVVPLKGLWEEVVPTLPDNHFDGILYDTYPLSEDTWHTHQFDFIKGHAHRMLKTGGVLTYCNLTSWGELLKTKYDNIEKMFEETQVPHLLQAGFKKEKISTTTMDIAPPSDCKYYSFKKMITPTIVKE |
| TRINITY_DN48662_c0_g1::TRINITY_DN48662_c0_g1_i2::g.37167::m.37167 | gi\|974084320\|ref\|XP_015240095.1\| | PREDICTED: phosphoglycerate mutase 2 [Cyprinodon variegatus] | 79.1 | MSKYRLVIVRHGESSWNQENRFCGWFDADLSEKGLEEAKRGAVAIKEAGMKFDVCYTSVLKRAIKTLWTIMEGTDQMWVPVIRTWRLNERHYGGLTGLNKAETAEKHGEEQVKIWRRSFDIPPPVMDKDHAYHKIISESRRYKGLK**AGELPTCESLK**DTIARALPFWNDVIAPEIKAGKNVIIAAHGNSLRGIVK**HLEGMSDA**AIME**LNLPTGIPIVYE**LDEHLKPTKPMAFLG**DAETVKKAME**AVAAQGKAKK |
| TRINITY_DN52149_c0_g3::TRINITY_DN52149_c0_g3_i3::g.68962::m.68962 | gi\|734634490\|ref\|XP_010745783.1\| | PREDICTED: alpha-actinin-3 [Larimichthys crocea] | 73.6 | M**TAVETQISYSTNSY**TITHEEAYMTQEDDWDRDLLLDPAWEKQQRKTFTAWCNSHLRKAGTQIENIEEDFRNGLKLMLLLEVISGERLPKPDKGKMRFHKIANVNKALDFICSKGVKLVSIGAEEIVDGNVKMTLGMIWTIILRFAIQDISVEETSAKEGLLLWCQRKTAPYRNVNVQNFHISWKDGLALCALIHRHRPDLIDYSKLRKDDPIGNLNTAFEVAEKFLDIPKMLDAEDIVNTPKPDEKAIMTYVSCFYHAFAGAEQAETAANRICKVLAVNQENEKLMEEYEKLASELLEWIRRTIPWLENRVAEQTMRSMQQKLEDFRDYRRIHKPPRVQEKCQLEINFNTLQTKLRLSNRPAFMPSEGKMVSDIANAWKGLEQVEKGYEEWLLTEIRRLERLDHLAEKFKQKCNMHESWTGGKEDLLSQKDYESASLMEIRALMRKHEAFESDLAAHQDRVEQIAAIAQELNELDYHDAATVNARCQGICDQWDNLGTLTQKRRDSLERVEKLWETIDQLYLEFAKRAAPFNNWMDGAMEDLQDMFIVHSIEEIQSLITAHDQFKATLPEADKERMATMGIHSEILKIAQTYGIKLSGINPYTNLSPQDISNKWDAVKHLVPLRDQMLQEEVARQQANERLRRQFAAQANIIGPWIQTKMEEISHVSVDIAGSLEEQMNSLKQYEQNIINYKSNIDKLEGDHQLSQESLIFDNKHTNYTMEHIRVGWEQLLTTIARTINEVENQILTRDAKGISQEQLNEFRASFNHFDRKRNGMMDPDDFRACLISMGYDLGEVEFARIMTLVDPNNTGVVTFQAFIDFMTRETAETDTAEQVMASFKILASDKSYITVDELRRELPPEQAEYCISRMTRYVGTDCPTGALDYISFSSALYGESDL |
| TRINITY_DN52091_c0_g1::TRINITY_DN52091_c0_g1_i1::g.68197::m.68197 | gi\|348537423\|ref\|XP_003456194.1\| | PREDICTED: apoptosis-inducing factor 1, mitochondrial [Oreochromis niloticus] | 64.2 | MLKCRTVWKKLAPLARDSSTLCRQNVKRAGLNNASAVRVPAAHM**SSGAAGGGGENKVYF**LLVGAACLGGGIYTYRTVTGDQRRYLDRMTRKSDAQISAAPSQSEPPAVPEPKPEAAPSPETEAAAPSETTEPSPVADSTPASLKVPSHAPYLLIGGGTASFAAARSIRARDPGAKVLIITDEPDLPYMRPPLSKELWFSDDPSVTETLRFKQWNGKERSIYFQPLSFYIQPDEFNSAQNGGVAVLTGKKVVHMDVRGNKVKLDDDTEISYDKCLIATGGVPRNLQAVERAGEEVMKRTTLFRKIDDFKSLDKVSRNISSITIIGGGFLGSELACALGRRSTESGLEVIQMYPEKGNMGKVLPEYLSNWTTEKVKKEGVKIISEALVKSVTYKEDKLEIKLKDGRLVKTDHIVAAVGLEPNVDLAKSAGLEVDSDFGGFRVNAELQARSNIWVAGDAACFYDIRLGRRRVEHHDHAVVSGRLAGENMTGASKPYWHQSMFWSDLGPDVGYEAIGIVDSSLPTVGVFAKATAKDTPKAATEKSGTGIRSESETEDTAVSPVASSTPAPALAPQKDNYGKGVIFYLRDKVVVGIILWNVFNRMPIARKIIKDGEEHADLNEVAKLFNIHED |
| TRINITY_DN56176_c2_g3::TRINITY_DN56176_c2_g3_i1::g.86492::m.86492 | gi\|554809542\|ref\|XP_005915666.1\| | PREDICTED: aldose reductase-like [Haplochromis burtoni] | 82 | M**ATTVTLNTGAQVPIVGLG**TWKSEPNKVLEAVKSAIAAGYRHIDGAYIYHNETEVGEGVQAMIKDGVVKREDLFIVSKVWCTFHAKSHVRKGCEKTLSDLKLDYLDLYLVHWPMGFKSGGEDFPLDSNGETINDDTYFLETWEGMEELVDAGLVKAIGVSNFNKEQIEAILNKPGLKYKPANNQVESHPYLTQDKLIDYCHSKGISVTAYSPLGSPDRPWAKPDDPSLMEDPNIKAIAEKYKKTPAQVLIRFQVQRNVIVIPKSITPHRIQENFQVFDFQLTDEEMKTILGFNRNWRVCPMQWSTKHKDYPFNAEF |
| TRINITY_DN49364_c0_g1::TRINITY_DN49364_c0_g1_i1::g.75641::m.75641 | gi\|808864067\|gb\|KKF15262.1\| | Heat shock cognate protein [Larimichthys crocea] | 76.1 | M**SKGPAVGIDLGTTYSC**VGIFQHGKVEIIANDQGNRTTPSYVAFTDSERLIGDAAKNQVAMNPTNTVFDAKRLIGRKFDDPVVQSDMKHWPFKVVNDATKPKVEVEYKGEIKTFYPEEISSMVLTKMKEISEAYLGKPITNAVVTVPAYFNDSQRQATKDAGVISGLNVLRIINEPTAAAIAYGLDKKVGGERNVLIFDLGGGTFDVSILTIEDGIFEVKSTAGDTHLGGEDFDNRMVNHFIAEFKRKFKKEINNNKRAVRRLRTACERAKRTLSSSTQASIEIDSLYEGADFYTSITRARFEELNADLFRGTLEPVEKSLRDAKMDKSQIHDIVLVGGSTRIPKIQKLLQDFFNGRDLNKSINPDEAVAYGAAVQAAILAGDKSENVQDLLLLDVTPLSLGIETAGGVMTVLIKRNTTIPTKQTQTFTTYSDNQPGVLIQVYEGERAMTKDNNILGKFELTGIPPAPRGVPQIEVTFDIDANGILNVSAVDKSTGKENKITITNDKGRLSKEDIERMVQEAEQFKAEDEVQKEKVTAKNSLESLAFNMKSTVEDEKLQDKISPEDKKAIVDKCNEVIAWLDRNQTAEKDEYDHQQKELEKVCNPIISKLYQGGMPEGGPGGMPGGMPGGFPGGAGGGSSSGPTIEEVD |
| TRINITY_DN47551_c1_g1::TRINITY_DN47551_c1_g1_i1::g.69020::m.69020 | gi\|734630814\|ref\|XP_010743767.1\| | PREDICTED: proteoglycan 4-like [Larimichthys crocea] | 78 | M**AVAYGAATQPGLLS**QQYPPPLLPKPGKDNVRLQKLLKRTAKKKASAQASQSAVHFRSSLSPVNEASPDLEHSDHSTPPRTPETPFNLYSVRQPQRFSVRPLYQHVASPYPQRAAYGRATSFSPQTVAMPSYSYSQHVTTVSSYSAPAYPPGPSPASGPFAQPAVPRISVSASSVPETIPAAEVKMTSFSTTAETHAGLRPSAGAVTPQPKSLGPTPYAAAGGQALIRPLTVLTPFVKSKSPRPTFKATEPSRSPKPMFDVPQIRMYTASTSYYETSRTPPVYDTSELTAIGSTVPQVVPTETKEDLTPVFEVRGGVTQVTQHTSLATDPMRKTPTSEIKRGATPTTEISTILTPTPEVKRATPTSEVKRATPTSEVKRATP |
| TRINITY_DN58239_c5_g44::TRINITY_DN58239_c5_g44_i1::g.44443::m.44443 | gi\|908512265\|ref\|XP_013121871.1\| | PREDICTED: phosphorylase b kinase gamma catalytic chain, skeletal muscle/heart isoform [Oreochromis niloticus] | 74.4 | MTKDEEIPDWVGAQEFYDKYEPKEILGRGVSSVVRRCIDKNTSVDYAVKIIDITPSDKMTAQEIEELREATVKEIDILKKVYGQDNIIQLKDCFESKAFFFLIFDLMKRGELFDYLTEKVTLSEKETRKIMRSLMEVVQFLHNQNIVHRDLKPENILLDDDMNIKLTDFGFSVQIQPGEKLKEVCGTPGYLAPEIIECSMDAGHAGYATPVDIWSSGVIMYTLLAGSPPFWHRKQMMMLRMILAGTYDFSSPEWEDRSDTVKDLISRMLVVDPSKRFTATDVLNHSFFSQYVVHEVREFSPYRRFKVICMTVLATMRIYCNYRRAKPVTKEVIQSDPYAVKPIRKLIDACAFKIYGHWVKKGQTQNRAALFENTPKAIL**LSIAAEADEPSHS**IW |
| TRINITY_DN46890_c1_g1::TRINITY_DN46890_c1_g1_i1::g.75494::m.75494 | gi\|658919436\|ref\|XP_008399461.1\| | PREDICTED: phosphate carrier protein, mitochondrial isoform X1 [Poecilia reticulata] | 71.5 | DVTKGQIDLPRGHSASPSSRLQLSGGAILRHRQTPPAGRTPTTMYPTSLTQLARGNPFSAPLFSLQKVEEPQQSLPGQRTRKLAAA**ATADEGDSCEFGSQKY**FVLCGFGGILSCGTTHTAVVPLDLVKCRMQVNPDKYKSIGNGFSVTVREDGVRGLAKGWAPTFIGYSMQGLCKFGFYEVFKIFYSDALGEENTYLWRTSLYLAASASAEFFADIALAPMEAVKVRIQTQPGYANTLRQCVPKMFAEEGLWAFYKGVVPLWMRQIPYTMMKFACFERTVELLYKYAVPKPRSECSKGEQLVVTFVAGYIAGVFCAIVSHPADSVVSVLNKERGSTAVGVLKKLGPKGVWKGLVARIIMIGTLTALQWFIYDSVKVYFRLPRPPPPEMPESLKKKLGLVE |
| TRINITY_DN48193_c0_g2::TRINITY_DN48193_c0_g2_i2::g.13059::m.13059 | gi\|657539597\|ref\|XP_008275951.1\| | PREDICTED: myozenin-2-like isoform X1 [Stegastes partitus] | 70 | MMMMMQASQNDMTKQRMFQAKALAKEARGGGLNLGKKISVPKDVMMEELNLPSNRGSRMFQERQKRVERYTVENAANAAYDTSNVYLEATHPPQVIPGPQTGKENQAFSIPGKHSLVMNLQKTVAKKGSPEVLAPGYSGPLRGVPHEKFNTTVIPKSYCSPWSEALGNNEELLNALNTQL**PELPQTLQSANYR**CFNRSARPFGGTMASKRVIPVMSFEAVESQNLPGIALDRICKRPNFNRAPRGWGADYSPESNEL |
| TRINITY_DN56108_c2_g2::TRINITY_DN56108_c2_g2_i2::g.120873::m.120873 | gi\|584022108\|ref\|XP_006805711.1\| | PREDICTED: putative peptidyl-tRNA hydrolase PTRHD1-like [Neolamprologus brichardi] | 95.3 | M**AASGAAAGAPSRLVQY**VVVRSDLVHKLSWPLGAVITQACHAATAAVHLHYGDPDTQRYLSELDSMHKVVLGAPDEAALSALSENLTQAGVSHKLWIEQPENVPTCLALKPYPKETVQPLLRKFKLFK |
| TRINITY_DN15360_c0_g1::TRINITY_DN15360_c0_g1_i1::g.127661::m.127661 | gi\|734613026\|ref\|XP_010733999.1\| | PREDICTED: NADH dehydrogenase [ubiquinone] iron-sulfur protein 6, mitochondrial [Larimichthys crocea] | 75.9 | WEVQAVVNMAAAVGRLLSFSKNAKVLVSPLRLSAVPAHRYSVEVSSTGEAITHTGQVYDEKDPRRARFVGRQKEVNKNFAINL**VAEEPVTDVEARVVS**CDGGGGALGHPKVYINLDKDTKVGTCGYCGLQFKQKHHH |
| TRINITY_DN43816_c0_g1::TRINITY_DN43816_c0_g1_i1::g.107385::m.107385 | gi\|410929655\|ref\|XP_003978215.1\| | PREDICTED: troponin C, skeletal muscle-like [Takifugu rubripes] | 61 | TDAQSDARSFLTEEMIAEFKAAFDMFD**TDGGGDISTKELGQ**V**MRMLGQNPSRE**ELDAIIEEVDEDGSGTIDFEEFLVMMVQQLKEDQAGRSEEELSECFRIFDKNGDGFVDREEFGVILHMTGEQVTEEDIDEMFGESDNNKDGRIDFDEFLKMMESVQ |
| TRINITY_DN87925_c0_g1::TRINITY_DN87925_c0_g1_i1::g.27020::m.27020 | gi\|1007773964\|ref\|XP_015824921.1\| | PREDICTED: cofilin-2 [Nothobranchius furzeri] | 56 | M**ASGVTVNDEVIR**VFNDMKVRKSSGQDEVRKRKKAVLFCLSEDRKKIVVEEGKQILVGDIGETVDDPYACFVKLLPLNDCRYGLYDATYETKESKKEDLVFIFWAPEGAPLKSKMIYASSKDAIKKKFTGIKHEWQVNGLDDIQDRTTLAEKLGGN**VVVSLEGKPL** |
| TRINITY_DN57646_c0_g1::TRINITY_DN57646_c0_g1_i2::g.61044::m.61044 | gi\|734628705\|ref\|XP_010742601.1\| | PREDICTED: uncharacterized protein LOC104929717 [Larimichthys crocea] | 65.3 | M**SSEKGPEVVGCDASIMM**NVWEIRLREHHQKLQLEHERLEKSALPTINKDWANRSTAKMGNYKRVERKAKPAETHTDNDNVWKSKRPQAPPTLPRGAGPGPHGRPGAQSGGFSPQASNKKEGQHNNYNNRLQLLMFLTQTQPSAMVWGKSWKYSKSLPSPAEGTAISDWGQCWMFATQQPYCEAGKPWANGPNMINPCSLHLWKRPDRNVEDSQELNLGLSTEEWHMSWRKPSNNNNKKEGTSSMNGENTGCFTLLMETQRLNEALCSSEWSESWRCTKHASEQEHGGLMDESVDKQDKLKEMSSKWEECWRFVNHHGCSQVPQFQGSENPECANWTAAMMIFNKHWNSDPSLKPHPDNAYNDFSEWDKSWQVTKNMSKPCEEIEKVLKALPPKMEMEAEKLEVKPKGQCSPSNEADLLYEKLKHDVIYWPKKESNQYKLPLLKQVENNLFSSEWSDSWKTLKNRLRMERRRVRPFRMSENRGDMKSTNTEWKDSWKFTCLSRLQEPELWQQDWVTTPQPRVDRARDQNYFGEFSKNVEESWEKSWRFSRGQHRSETSQGGSSTVCHHSDDSLVQKKCVRSVADWQAAWMVSESQFHHDKPSLMQWREAWKWSVFHTEKWFEKMIQDNRMEHGMEIQTLKEKIYLQTAKAKMIQSFVNQMVTERHPEENWRDSWKAGSLLNHQTNHYESHGKKINSTNQQLHPIASEHGSKWGRSFRLANPMPHVEQPWVESTPNPCHYTVMWSRESNIHNRINTSFSNNPAILRLWGNSHQFLQDGGVKSKGMGSTKAPVDPMVITTKTKIKRNLYGNINTDKQSGRKWAGCHLLGKTQPRPKRGSPSGKKLMLVEDETKAKFFEEWAESWKLLIQPGSLKKTPMKSLAGWAESWKFLLPPYQPMNVSKAK |
| TRINITY_DN58429_c0_g15::TRINITY_DN58429_c0_g15_i3::g.90372::m.90372 | gi\|657565229\|ref\|XP_008285902.1\| | PREDICTED: myomesin-1-like isoform X3 [Stegastes partitus] | 60 | IDRCEVGTHHWAQCNDSPVKYARFPVTGLVEGRSYVFRVRALNKAGVSRPSRVSEPVVAMDPSDRARLRAGPSAPWTGMIKFTEEDSTAGVIPGDPTDVVVTEATKSYVVLAWKPPLQRGHEGVMYYIEKCISGTDTWQRVNTGMPVKSPRFALFDLAEGKSYSFRVRCCNSAGVGEPSVSTGDITVGDKLDLPSTPGSPVVTRNTDTSVVVSWAASKEVKHLVGYYIECTVVGTDVWMPCNNKPVKQTRFVCHGLATGANYVFRVKAVNAAGYSQSSSDSDAVVVQAAISVPGKPTGVKLLEAVKDYMMLGWNEPSKNGGADVRGYFVDYRTVKGDVVGKWHEMNHQALTTTSYKAENLKEDIFYQFQVRAMNMAGVSEASLPSKSLECKEWTLTVPGAPVGLHVLEVRDTTVVVLWEPPAFDGRSPVNGYYLDHKVASAGEAGWKAGHEKANKKKYMKVTGLKAGTSYVFRVRAQNLAGVGKTSAVLGPVLAQTR**PGTKEIYVDVDD**DGVISMMFECSEMNESSEFVWSKNYKAITDTSRLTIVSEKGKTRAIFNSPSLEDLGIFSCVVTNTDGISSSYTLTEEGLKRLLDISHEHKFPVIPFKNEMAMELLEKGRVRFWTQVEKWTADCHVEYVFNDVIVTEGKKYSMNFDKSTGIIEMFMDSLEVTDEGTFTFNLVDGKAKGTTSLVLIGDEFRELQKKSEFERAEWVRKQGPHFVEYLDFAVTPECDVLLKCKLGNIKAETEITWSKDSIEIREDDEDAQKIEKKDGDMTFNIGKWIIKQEKRKEGKKAPADEVPPPPRPKISKSDAGIYEVFLKDERGKDKSTFNLTDAGYQAVMNELFRVIANSSSEVKVTSTEHGIILYSTVTYYNEDLRVGWLHKDAKIAASERVKAGVTGEQLWLKINEPTEKDKGKYAMDIFDGKDGVKRAFDLSGQVWEEAFEEFQRLKAAAIAERNRARVVGGLPDVVAIQEGKSLNLTGNVWGEPTPEVSWTKNERELTSDDRYKLKFEHGKFASITIAAVTTADSGKYALVVKNKYGTEAGEFTVSVYNPEEDEHKEEKKD |
| TRINITY_DN48852_c2_g4::TRINITY_DN48852_c2_g4_i1::g.72745::m.72745 | gi\|110808625\|gb\|ABG91151.1\| | skeletal muscle fast troponin T isoform 2 [Hippoglossus hippoglossus] | 89.5 | M**SDTEEVDAGEEKPKFKPS**APKI**PDGEKVDFDDIQK**KRQNKDLIE**LQALIDAHFE**CRKKEEEELIALKDRIEKRRAERAEQQRVRAEKDKERQARREEERRVR**EEADATKKAAEEAKKKSA**L**SSMGSSYSSHLQ**KADSKRGGKKETEREKKKKILAARRKQLN**IDHLNEDKLKDKINELH**DWMCQ**LESEKFDHMER**LKRQKYEVTTLRKRVEELSKFSKKGAAARRRK |
| TRINITY_DN54997_c0_g3::TRINITY_DN54997_c0_g3_i1::g.60110::m.60110 | gi\|734636214\|ref\|XP_010746735.1\| | PREDICTED: protein S100-A14 [Larimichthys crocea] | 29.1 | M**PQYSDLEKAIST**LVTQFHSASADNGPTLKTDEFKGLLSSQMPNLVKGIGSEQGLGEIMRKMGVGDGEGVSFKHFWSLIQSLATTQHGLLASNTSSPCSCMVL |
| TRINITY_DN49794_c1_g1::TRINITY_DN49794_c1_g1_i1::g.57338::m.57338 | gi\|583995636\|ref\|XP_006792942.1\| | PREDICTED: myc box-dependent-interacting protein 1-like isoform X1 [Neolamprologus brichardi] | 66.4 | SGIRVTSRETSQERERERERRGGGEEGGVGGGFYAPSVSRRHFSLQSRPDSNRRHRAAM**AEMGKGVTAGKLAIN**VQKRLTRAQEKVLQKLGKADETRDAAFEEMVANFNKQMTEGSKLQKDLKAYVLAVKTLHDASRRLQDCLADMYEPEWFGKEEMDTMVEDTDTLWLDFHQNITDKSLMSVDTYLNQFPEIKARIAKRDRKMVDFDSARHHFASLQKGKKKDEAKIAKAEEDLGRAQKIFEELNVELQDELPALWDNRVGVYVNTFQSLAGQQEKFHKEMSKLSQNLNDIMTKLEEQRLLKKGGTAAAKTEDGAKSEEANHSESASSAPKSSVTNGAGGDLPPGFLYKVKAVHEYAATDGDELELEIGDTVLVVAFDNPDEQDDGWLMGLKESQWMQKKDFSAKGVFPENFTQKI |
| TRINITY_DN58608_c3_g1::TRINITY_DN58608_c3_g1_i1::g.62351::m.62351 | gi\|657583703\|ref\|XP_008295972.1\| | PREDICTED: titin-like isoform X11 [Stegastes partitus] | 67.8 | DGGAPILGYSVKYRDFIRKPEPEVEEEEEEYEDEEEEEEEPESPEELARWVEAIPLTKSLEFTIKGLKTDAEYEFCVKTINKIGSSVRSLYSDAAAAMDRTSEPSFDVDIEMRKVHLVKHNTAFTLSVPFKGKPVPSVEWTKEGVDLKVRGTIEATDISTSLTVEKATRNDSGEYSVTIESPLGKATLPMVVKVLDSPGPPVNVKVSAVTRDSATLIWEPPENDGGDAVKAYHVEKREASKKAWVCVTSNCHALSYKVEDLQEGAIYYLRVIGENEYGVGVPQEAKAGTKITEVPSAPMKLGVANVTKDCVTIAWTRPEYDGGSRVTGYLIDALEKGQTKWVKCATVRTMTHTIKSLREGAEYFFRVRAENHAGLSEPKEMIVPVIVKEIQEAPEFDLKNYPKNTVYVKAGSNLTFEIPLTGRPMPKVTMSKNNVVIKGSKRLLTEVTPDSLIITLNESIASDAGKYDVTASNAGGTTKIFIIFVVMDRPGPPVGPVEIGEVGETTVCLKWAPPEYDGGSPVTNYVVLKRETSTPTWAEVSTNIARSAIKVTKLTKGEEYQFRIKAQNRYGLGDHIDSKSVMIKLPYTIPGPPSTPWVSFVSRESLTVCWNEPVNDGGNPVIGYHLQMKERSSILWQKVNKTAIPGNQWRITNICPGLFYEFKVAAENAAGIGKLSKTSEEVLAIDACEPPANVHVTEVTKNSVSLAWQRPPYDGGSKITGYSVERRDAPNGRWVKANFTNIIEMGFTVSGLSQDESYEFRVYARNAVGSVSNPSLIVGPVTCIDACGAPAIDLPPEYLDVVQYKAGTSVKLRIGIIAKPLPTIEWLKNGKELVATSTVSIENSTDSSAVLIKDATRHDTGSYEVKIKNVLGSASATIRIEILDKPGPPTGNINFNLITADRILFCWEPVPESDQGGSSLTHYIVERRETSRVVWSTISDKHSTDQTHVSVSKLMRGNEYVFRVMAVNKFGVGEPLESEPVIAKNAFVSPGQPHTPEVNIITKSTMVVEWDKPGVDGGSAVTGYYLERRDKKSSRWIRVYKDPISELKKTVYHLTEGNEYQYRVCAINKAGEGPFSDGSDYYKAAD**PVDPPDEPCKL**KVVDSTKTSITMGWSKPEWDGGSEVISYMLEKLVEGEEEWAMITSKGEVKTTEYTVHDLKPDVNYFFRVSAVNCAGRGEPLEMTEPVQAKDILEEAQIDSDVAMRTHYIVKAGKDVELTVPLKGRPSPTASWSKGEECIDRNPQYEFHHSDTTTVLVMREVTRLDTGKYTVKIENGVGEPKTLTLSVKVQDSPAQCRNLVLKEVTRGKVILCWEPPLLDGGAEITNYIVEKRDSSKRSFSAVTSKCTDTTYTIEDLSEKTSYFFRVLAENENGVGDPCDTPEPVKATETPGPVKEVSMKDSSKTSVTLQWLKPDYDGGSIISDYVIEKKLKDEEWSLGGTSRQCEFEVKKLKEHSDMFFRVASRNEKGQGDFVEIGPIKVIDYIITPEACLAEYPGGSISVRLGHNVHIELPYKGKPRPATLWLKDNLPLKESDQIRFKKTENKATLMIKNVKKENEGKYTLTLDNKVNRRSFHIHVITLGPPSKPVGPIRLDEVRAENIMISWDEPNDDGGGDITCYTVEKRDTSQNVWKMACSSVQDTQFRVPNLIKGLQYQFRVCAENRYGASEPLISQMVVAKHQFRPPGPPGKPVVYNITNDGMTIRWEQPIYDGGTPIQGFHVEKKEKNSVMWQKVNTMLVKENDFRILELIEGLEYSFRVYAQNDAGFSRMSDESKPAMAVSPVDPPGQPDYTDVTIDSVALKWDAPKRDGGSKITGYTIEKRQGHGRWFKANLTDVHECEYTVSGLATNERYEFRVIARNAIGVVSPPSNSSGLIVVRSENACPNIEFGPEYFEGLTVKAGDNIRLKVTITGRPVPKIVWFRDGVEVTKKMMDIINVAGSSTLFVRDADRTHSGLYTVEATNGSGSKKENILVQVQDTPGEPVGPITFSHISEGKCTLSWSPPVNDGCSEISHYIIEKRETAKISWALVSDECTECTFDASKLIKTNEYQFRVSAVNKFGVGRPLESTPIIAQMQYTTPDAPGTPDATEVTGESITLSWAPPTSDGGNPIQYYIVEKREKKTVRFYKVITKKPIEECGHKVIHLTEDMEYEFRVLAVNDAGVGTPSNISMPIKAAEPKDIPCAPSVVCVSDSTNTSISLEWSRPADDGGMEILGYIVEMVKGEDEEWKRVNEELIPETHYTVAGLETGSEYSFRVAAVNHIGRGEEKETPEPAQAVDRLTPPQVDIDASFRQTHIVKAGGSVCLGIHFRGKPVPTATWVKEEGELSVLSEITTTDGYTSLSIENCSRTDTGKYTVNLENASGSKDVTFTVKVMDTPGPPQDVSFMEVARGTVTLTWKPPLNDGGARIHHYVVERREASRRTWQQSGGKCTKHVLK**IEDLLEGVP**YFFRVSAENQHGMGEAFELTEPVTATAEPAPPKRMDILDTTDSSVVLGWLKPEHDGGSRIQGYVIEAKPKGTDKWVVVGNTKNLTYTIDKLNKGDEYDIRVKAKNEAGNSRPRETVAPVLIKEPHIEPAADLSEITNQLVTCRSGSTFIIEVPISGRPAPKVSWKLEEMRLKSSDRVTIKVTKDRTTISVKEAMRGDGGKYYLTLENVAGTKTFTIEVNVTGRPSPPSGPIEITSITSESCIVNWQPPEDDGGTDITNYIVEKRESGSTAWQLINSSVKRTTLHVSHLTKYMQYTFRISAENRFGVSKA |
| TRINITY_DN52732_c0_g1::TRINITY_DN52732_c0_g1_i1::g.123903::m.123903 | gi\|1737228\|gb\|AAB38887.1\| | lactate dehydrogenase-A [Sphyraena idiastes] | 58.1 | MTTKEKLITHVMKEEPIGTRNKVTVVGVGMVGMASAVSILLKDLCDELALVDVMEDKLKGEVMDLQHGSLFLKTHKIVGDKDYS**VTANSRVVVVT**AGARQQEGESRLNLVQRNVNIFKFIIPNIVKYSPNAIL**LVVSNPVDILT**YVAWKLSGFPRHRVIGSGTNLDSARFRHIMGEKLHLHPSSCHGWIIG**EHGDSSVPVWSG**VNIAGVSLQSLNPKMG**AEGDTENWK**GVHKMVVDGAYDVIKLKGYTSWAIGMSVADLVESITKNLHKVHPVSTLVQGMHGVKDEVFLSVPCVLGNSGLTDVIHMTLKPEEEKQLVKSAETLWGVQKELTL |
| TRINITY_DN53297_c2_g13::TRINITY_DN53297_c2_g13_i1::g.16570::m.16570 | gi\|734605520\|ref\|XP_010729866.1\| | PREDICTED: AMP deaminase 1 isoform X1 [Larimichthys crocea] | 83.9 | MPKVIVPEGGSHQKTDDKMRAFAEEVF**ASETKDENVRDEIS**MF**DVAEDCPIFH**HELAHHLHADDDHEKRKRLQRSRTM**AVPAAAASAPVVSLK**VETPTYLEVPDFQRVAIIGDYASGVTLDDFELSCKGLYRALTIREKYMRLAYQRYPRTTSQYLRDIEGESFKPEDQLQPVFTTPPKNGEDPFDPKDLPKNLGYVARMKDGVIYVYNDAAAADKHQPKDMPCPDYNTFIDDMNFLIALIAQGPTKTYTHRRLKFLTSKF |
| TRINITY_DN36664_c0_g3::TRINITY_DN36664_c0_g3_i1::g.7820::m.7820 | gi\|6138950\|gb\|AAF04404.1\|AF051370_1 | AF051370_1 cytochrome c oxidase subunit VIa precursor [Thunnus obesus] | 55.3 | MFATPVASAARRVLAAASHSSHEGKAKTWKILSFVLAIPGVSVCWLNAYMKGQAHSHEQPEFIPYPHLRLRTKKFPWGDGNHSLFH**NPHTNALPTGYESSSHH** |
| TRINITY_DN58819_c1_g4::TRINITY_DN58819_c1_g4_i3::g.92778::m.92778 | gi\|657592091\|ref\|XP_008300557.1\| | PREDICTED: calpain-3-like isoform X1 [Stegastes partitus] | 68 | FGLFCKFTLCFCTHKKKRGRTPLHKLPSETGM**SKAAAGTDNMGDNTGKVPVVEE**IQVKVLYETEAS**FEPDDKSDYSPTGAN**SIYSAILSRNEAVKDAKRLKTFLELRDKYVKKNVLFEDPLFPADESSLFYSQKPAMKFEWKRPSEICENPEFIIDGAKRTDICQGELGDCWLLAAIACLTLNEKLLYRVIPPDQSFTENYAGIFHFQFWRYGEWVDVVVDDRIPTCKNQLVFTKSFRQNEFWSALLEKAYAKLHGSYEALKGGNTLEAMEDFTGGVTEFFDLDEAPKELYKIMRKALERGSLMGCSIDAFSPSEMETRIEQGLVRGHAYSITGLEECNEVDKDTKVHLIRLRNPWGWVLWKGPWCANSKEWSTISVGDKTNLQKQTVETSEFWMCFDDFKKYYTKLEMCNLTPDTLQGDERHSWSVSVNQGRWVRGSSAGGCRNFPDTFWTNPQYRLQLYEEDDDPEDEKLTCTVVVALMQKGRRMQRHQGAKFLTIGFSIYEVPKEMRGQNQHLQKDFFLYTASKAKCKTYINLREVTARFRLPPGEYVIIPTTFQQHQDGEFILRVFSEKRNTSEEAESTIDPGQAQQDKKTKGKPIVFVSDRARANKEIEHDGILGEKKKKKRKMLEPEEETEEEKQFRAIYEKIAGEDMQICANELKTVMKNVLAKHSEIKTTEGFSLETCRSMIALMDTDGTGKLNLQEFKHLWKKIKEWQLIFKRYDKDKSSSISSFEMRNAVHDAGFHLNKQLYDILAMRYADEHLNIDFDSYICCFVRLEGMFRAFNAFDKDGDGLIKLNVLEWLQLTMYS |
| TRINITY_DN99034_c0_g1::TRINITY_DN99034_c0_g1_i1::g.27726::m.27726 | gi\|1012819516\|gb\|JAR66467.1\| | 40S ribosomal protein S19 [Fundulus heteroclitus] | 63.5 | ARFNTSWKPRSTLFPSLSTSKM**PGVTVKDVNQQE**FVRALAAFLKKSGKLKVPDWVDLVKLGKHKELAPSDENWFYIRAASTVRHLYLRGGAGVGSMTKIYGSRQRNGVCPAHYSVGSKNVARKVLQALELLKMIEKDPNGGRRLTSQGTRDLDRIAGQVAAANKKTV |
| TRINITY_DN47410_c0_g1::TRINITY_DN47410_c0_g1_i1::g.12333::m.12333 | gi\|657585462\|ref\|XP_008296929.1\| | PREDICTED: peptidyl-prolyl cis-trans isomerase A-like [Stegastes partitus] | 57 | LTFSKRRDFSLLAVPTSADQLSHITFANMLLLSNRIKFCSLAFTAARLY**SSGPAAANLNPTVY**FDIAADNEPLGRVTFELNAEVVPKTAENFRALCTGEHGFGYKGSTFHRVIPQFMCQGGDFTNHNGTGGKSIYGWKFPDENFKLKHTGAGILS**MANAGPNTNGSQF**FICTAKTEWLDGRHVVFGSVKEGLDVVKKVETFGSRSGRTSKRITITDCGELK |
| TRINITY_DN38261_c0_g1::TRINITY_DN38261_c0_g1_i1::g.102034::m.102034 | gi\|736178360\|ref\|XP_010767913.1\| | PREDICTED: collagen alpha-1(I) chain-like [Notothenia coriiceps] | 82.6 | AGPPGPAGPTGAPGPQGPLGNTGAKGARGPAGPPGATGFPGAAGRVGPPGPSGNSGPPGPTGPAGKEGPKGNRGETGPAGRAGEIGNAGPPGAPGEKGNPGAEGAPGSAGIPGPQGIAGLRGIVGLPGQRGERGFPGLPGSLGEPGKQGSAGPGGERGPPGPMGPPGLAGAPGEAGREGTPGNEGSAGRDGPAGPKGDRGESGPAGASGAPGPPGAPGPVGPAGKSGDRGETGPAGIAGPAGPAGPRGPAGALGLRGDKGESGEAGERGMKGHRGFTGMQGPPGPSGPSGEQGPAGTAGPAGARGPAGSSGAAGKDGMSGLPGPTGPPGPRGRSGEMGPAGPPGPPGPPGAPGAPGGGFDLG**FIAQPQEKAPDPFR**MFRADDANVLRDRDLEVDTTLKSLSQQIEQIRSPDGTRKNPARTCRDLKMCHPDWKSGEYWIDPDQGCTQDAIKVYCNMETGETCVTPTQPEVAKKNWYVSKNIREKKHVWFGEAMNDGFQFEYGSEGSQPEDVNIQLTFLRLMSTEASQNITYHCKNSVAYMDATAGNLKKALLLQGSNEIEIRAEGNSRFTYSVLEDGCTSHTGTWGKTVIDYKTSKTSRLPIIDIAPMDVG |
| TRINITY_DN48192_c0_g3::TRINITY_DN48192_c0_g3_i1::g.12991::m.12991 | gi\|14194770\|sp\|O13164.2\|HBB_DECMA | HBB_DECMA RecName: Full=Hemoglobin subunit beta; AltName: Full=Beta-globin; AltName: Full=Hemoglobin beta chain | 77 | M**VDWTDAERSAIS**ALWGKIDVGEIGPQALARLLIVYPWTQRHFSTFGNLSTNAAILGNPKVAAHGKTVMGGLELAIKNMDNIKGAYKSLSEKHSQVIHVDPDNFRLLAEITTICLAAKFGPSVFTPDFQEAWQKFENAVVAALARQYH |
| TRINITY_DN5454_c0_g1::TRINITY_DN5454_c0_g1_i1::g.1153::m.1153 | gi\|734603098\|ref\|XP_010728538.1\| | PREDICTED: 14 kDa phosphohistidine phosphatase-like [Larimichthys crocea] | 63.2 | DRDRTMLLSVVGTGRLAVYGLRRTVSGVLTKATM**ADALAKIPDVE**IDPEGTFKYILVRVKVKDGDAHKDIVRGTKSAEYHNHIFE**KVSPAVEALG**MECKCLGGGKIEHNSQEKKLRVFGESTGFGKADHSVSVEKLKSAY**SNYEITCSDDKK** |
| TRINITY_DN9484_c0_g1::TRINITY_DN9484_c0_g1_i1::g.1675::m.1675 | gi\|734595174\|ref\|XP_010730128.1\| | PREDICTED: lactoylglutathione lyase-like [Larimichthys crocea] | 68.9 | M**SDKGLSDEAAAAACKD**GDPVTKDFMMQQTMLRVKDPVKSLDFYTRILGMTLLQKFDFPSMRFSLFFLGYEDKKEIPADVKEKTAWTFSRRATIELTHNWGSESDESQSYHNGNSDPRGFGHIGIAVPDVYAACKLFEEQGVTFVKKPDDGKMKGLAFIQDPDGYW**IEILSPNNMVSITS** |
| TRINITY_DN49860_c0_g1::TRINITY_DN49860_c0_g1_i1::g.120076::m.120076 | gi\|348515631\|ref\|XP_003445343.1\| | PREDICTED: desmin-like [Oreochromis niloticus] | 66 | M**SKSYSSSAQSASSYR**RTFGSGVGSTPMSSLFSSGGGRSSSSSHMASRVYEVKSSPSFSSYRLSSGAGGAGYGSSTAMRAYSGEKLDFNLADAMNQDFLNTRTNEKAELQHLNDRFASYIEKVRFLEQQNAALTVEIEKLRGREGPGRVAELYEEEMRELRRQIEAISNQRARVEVERDNLADDLQKIKLRLQEEIHQKEEAENNLSAFRADVDNATLARLDLERRIESLQEEIAFLKKIHEEEIRELQSQMQDTQIQVQMDMSKPDLTAALRDIRVQYEGIAAKNIAEAEDWYKSKVSDLNQAVNKNNDALRQSKQESMEYRHQIQSYTCEIDSLKGTNESLLRQMRDMEDRMTREASGFQDTISRLEEDIAKMKDDMARHLREYQDLLNVKMALDIEIATYRKLLEGEESRITTNVPVQAAYSSIGFRETSPESQHQRSSEVHSKKTVLIKTIE**TRDGEVVSESTQHQQDIM** |
| TRINITY_DN49056_c0_g1::TRINITY_DN49056_c0_g1_i1::g.118941::m.118941 | gi\|551509463\|ref\|XP_005806258.1\| | PREDICTED: keratin, type I cytoskeletal 18-like [Xiphophorus maculatus] | 66.1 | **MKSSKQTTYS**MRSSTSSRAPAISISRTSVPVYKAPSIHGGAGGDRISISSSYRSGLGSGMGMGMGSMKAGGFSGGIQVTTSGNSADIMGNEKFAMQNLNDRLANYLETVRTLEAANHKLEVKIKEALEKSGPDFRDYSKYQAILDDLRRKVFDATVDNARLVLNIDNARLAADDFRVKFESELAIRQSVEADIVGLRKLIDDTNMGRMNLESEIESLKEELIHLKKNHENEVMEMRNQIAQSGVHVDVDAPKGQDLSQIMAEIRAKYEKMALKNQEELKAWHESQITEVQTQVSQSTEALKGAQTEVNDLRRQLQTLEIELESQRSLKGSLEGTLRDTEMRYNMEIESLNNVLLGLEAELTQLRNNIQLQTQEYEALLNMKMKLEAEIATYRRLLDGEDFTLQDALEEQKTVKKTKVMTVTQTL**VDGKVVSSSTETKQL** |
| TRINITY_DN115797_c0_g1::TRINITY_DN115797_c0_g1_i1::g.133756::m.133756 | gi\|736155087\|ref\|XP_010769179.1\| | PREDICTED: patellin-2-like [Notothenia coriiceps] | 79.7 | MEAAIQTLVKVFLKSAKGKENLGKKEFQSLVKSQLSNILSDTDSKEAVNNMGQGLDANQDGKVGFEEYMKLVGYLAVSLSEQRDLAKEEPAQNDASGQAAQSAPAKEEEKPEANLEAKEEAKPEA**SEEPKAEAVAEVK**VESNAEGEVEVKVEVKAEGETQPEAAKEEPAAAAVAEATAAAVPAAAET |
| TRINITY_DN20884_c0_g1::TRINITY_DN20884_c0_g1_i1::g.28864::m.28864 | NA | NA | 93.9 | MPGKPAPIKKSPAKKAVEKAPEPAPAPEPEPAPAEAPP**APAEAAPAEAPAPAEAAAAPAEGEAAPAAPAEDTPAAEE**SAPAAPAEAAVVEEPPKAPTPP |
| TRINITY_DN57974_c2_g3::TRINITY_DN57974_c2_g3_i1::g.44135::m.44135 | gi\|657574681\|ref\|XP_008291057.1\| | PREDICTED: titin [Stegastes partitus] | 63.8 | LLKGNEYLFRVRAVNKYGEGETLESEPIRAMDPFTIPSAPTDVEVTSATSDCMTICWKRPASDGGSRISGYVIEKREKQGVRWVRVNKKPIYDSRVKASGLHEGCEYEFRVFAENIAGLSPSSKTSECYVARDPCDPPGKPEAVVITRENITLEWAKPKYDGGSTITGYVVEKRELPDGRWMKANFTNVIENQFAVTGLTEGESYEFRVTAKNGAGVWSAPSESVKITAQDVIEGPTAFIDQKFKSVTGVQAGETFVIEADYFGKPLPDVTWLKDGKEIDKATMRMEIKNTLTHTTLTVRDSTRADGGHFVLILSNTGGTTSVPVNVRVLDRPGPPDGPLRVKFVSAERCNLNWNPPVNDGGACVSQYIIEKRETSRVTWTGVDTQVEAVSYKVSKLVPGKEYIFRVAGVNKFGVGEFLESDPIIAQNPFKTPSAPSTPMASAVTGDSIALTWERPESDGGSEIDGYILEKRDKEGVRWTKCNKRRLNDLRFRCTGLTEGHYYQFRVMAENAAGAGAPSEPSEYIKVCEATYPPGPPTNPKVTDYSSSTVSLTWSKPIYDGGAAINGFVVECKETDEDEWTVCTPSAGVAETNYAVKRLKENAEYNFRICAMNAAGIGEYVDLPGSVKAAEKLEAPEIELDSALRKIVNVRACSTLHLSVNIKGRPEPEVKWSKEGGALSERAQIEVTNSYTALLIENVNRNDTGKYVLTAENCNGTKSAFINVRVLDSPSAPTNLEVKEVKKDSVTISWETPLIDGGTKISHYIVEKREEARKAFTSVCSTCVRNSYKIDNLQEGSFYYFRVLAVNEFGAGLPVETTEPVKVSEAPLPPGKITLSDVTSNTVKLSWEKPDHDGGSKITRYIVEMQAKGDDTWTICAESKALEVTATGLTRGKEYFFRVSAVNEKGRSEPKSLLAPVAVKDTSAGPIINLLSNTFSVKAGNDLKIEVPFKGVPPPTVAWKKDGNILKETSRVNVQTSDSASQIMIKEATRVDVGVYEVTLSNSVGITSAEILVNVFERPGPPCDLSVDEVSADFVSLSWQPPHYTGGSEITNYVVKKRDTGSTAWQTVSATIARTSIKISRLTQGTEYQFCVAAENRYGKSQFVEFEPVVAQYPFKSPGPPVNLHVVQASKSVMVIAWSKPDSDGGSPIIGYHIECKDQSSILWTKLNRNPVTENQFKVTSVEEGLIYEFRVYAENMAGVGPCSTASDPAAARDQCDPPCNLTVTNITNSSVSLSWDKPEYDGGAKITGYIVERKELPENCWLKCNFTNLLDTFLEVTSLTEGEQYDFRVIAKNSADLLSAPSETTGPVTVQPDVEPPKIILEDKLRQVVIVKAGDFLKMDADISGRPNPTVFWLKNGRNIGTKGRIEVTATKSHTSLLIRESVRKDSGQYTLTLQNTGGTTSKVITIKVLDRPGPPAGPLEVSGLSAEKCTLSWGPPNETGGAEIMYYIVEKCETSRVSWTLMYDDMMATTCKITKLLKGNEYIFRVRGVNKYGDGEALESEPATAMDPFTVSAAPTNVQVTAVTSEAMTICWERPVSDGGSSIAGYVIEKREKTGLRWCRVNKKPVYDLRVKASRLRDGCEYEYRVFAENAAGLSAPSNPCPLTKAEDPQFLPSPPAKPKIIDSTKTTVTLSWNKPLFDGGAPVTGYRVEYRKSLDDEWFVGVQKTKNTEFTVVGLTPGAEYVFVVKSINKIGVSEPSPESDRQIAKEREDEPVFDISNEMRKTLIVKDGSSFTMTVPFRGKPIPSVSWTKPDVDLRVRAVIDTTDTFTSITLEKATRNDSGKYTVTLSNVAGTSSLTLSVRVLDSPGPPTHIEVKDVTKTSATVTWDTPENEGGGPVKNYVVDIREASKKGWTRLTDTCHRLTYKLTDLQEGEVYYFRVTGENEYGVGVPAETKEGTKITEKPTPPPKLGVTDVSKESVSLAWAKPEHDGGSRITSYLVEARENGQEKWVRCGVTKSIHLTVSGLRENAEYFFRVRAENHAGFSEAKEMLNPVLLKDQLESPEINMKDFPHNTVYVRAGSTLKCEIPLTGKPLPKVSLSKDNVLLKSTMRFNSEVTPDSIKITLRESIAGDSGRYDITASNSSGTTKSYVNIVVLDRPSAPVGPVELYDITEDSVSLKWLPPAYEGGSPITNYIIQKRETTTANWMDVSSAVARCTMKIMKLTTGLEYQFRIKAENRYGISEHIDSPTISVNLPYTIPEAPFTPEITAVTRETITVAWKEPKADGGSHVFGYHLQMKDRNSILWQRVNKMVIRATHFKVTNINAGLIYEFKVAAENAAGMSAFSKVSDAVLAIDACEPPINLRISDITKTSISLAWQKPNFDGGSPVTGYIIEKREGVSARWSKANLTNVTGTRFTVTGLTQDETYEFRVMAKNAVGSVSNPSMTAGPATCVDTYGAPEVEIPQEYLNEVKHKAGSNVELKFNITAKPAPTIEWFKDGRELEPTSQLSFKHTFEHTSLFLKETTRLNSGTYEVKVKNSLGSACGVVRLLIQDKPGPPAGEIQFKKVTADNVTIMWSPPADEGGAMVSHYIVEKRETSRVMWSIVSEKLVDCIVNVPRLIQGNEYIFRIRGINKFGIGDPLESAPVIARNAFVPPSEPSKPKVTMITKSTMTVIWERPALDGGSDIDGYYLEKREKKSLQWFKVVKGTIRDTRQKVSNLVENNEYQYRVCAVNKAGAGNYSEASDLYNAYDPIDLPGEPSKLRVVDSTKTSITLGWEKPVYDGGSKITHYILDQLNSEEREWVTINPQVKTCEYVVSHLKPGSYYFFRVYAVNCKGKGEDIKMYQPVQAKDILEEADLDLDVPMTTQYTAKAGRDVEVFIPLKGRPAPNVTWRRGDRNIAGDNRFTITSTERATTLLIPKVTRDDCGKYLLEIENGVGEPKIITVSLKVLDSPSTCQKLMIKNVTRGKLTLSWEAPRIDGGSPVTNYIVEKKASTMKAFQVITAECANTTYKVSGLEEDVAYFFRVSAENEYGVGDTCETTEPVRATETPGAVKQLVMVDSTKSSVTLQWARPDHDGGSHITEYIIEKSTKEDPTWTLGATCKRCSCEVTGLKENAVMDFKVFAKNEKGRSDFSQIGPITVMDFIIPPEANLIDYPSGELAVRIGQNIHIELPFKGKPRPVISWLKDNLPLKESEKVRFR**TTDNKTAITVR**GAKKEHAGQYTLVLDNRVVKNYFDINVITLGPPTAPVGPIRFDEIKAQSIIISWDEPKEDGGGEITCYSVEKRETSQANWKMVCSSVVRTTFKIPNLVKGTQYQFRVRAENKYGVSESLNSTDVVAQHQYKPPGAPGKPVAFNVTSDGMTIKWEAPGFDGGSPIVGYHVEKKDRNSLLWQKVNSTIISNKEYRIIGLMEGLEYSFRVYAENNAGLSPVSEQSKHALAISPVDPPGTPVCIDVTRDSVTLQWAIPKKDGGSKIVAYSVERRQGRGKWLRCNFTDVCETQFTVTGLSPGDRFEFRVIARNAVGTVSPPSNSSGYIMTKDESIAPEIEWSPDQVATLRAGENVRLGCSITGRPVPQVVWYKDGKEIDKRTMIDIEITTGIGTSSVFVRDADRNHRGIYTVEAKNSSGTKRADVNVRVQDTPGPVEGPIRFTGITAEKCTVWWNPPENDGCAAISHYVVEKRETSRISWAMVTSKCEACSYNATNLIKGNEYQFRISAVNKFGVGKPLDSDPIIAQMQYTVPDAPGTPDATHITGDSITLGWTRPRTDGGNEIKHYILERREKKSLRWVKVSSKRPITELRHRATRLTEGYEYEFRVMAENGAGVGPASSTSRLFKCREPTCTPSAPSMIKVIDSTKSSVTLEWTKPVFDGGLEIIGYNIDMCKASLLEWHRVNSQICIHTRYTAKGLVPGELYKFKISAVNGSGEGEASEMASAVQALDRLTSPEIDIDANFKQTHIVKNGGMVTLHIAFRGKPAPLATWSRADGELPIMADVNTTESFSTLTIEGCTRYEAGKYTLSLENNSGRKSITFTVKVLDTPGPPGAITFKDVTRGALTMMWDAPTNDGGARIHHYIVDRREASRLAWQEVSTKCSRQMIRVTGLDIGIPYLFRVIAVNQYGQGEPHELTEPVIATEEPAPPKRLDVVDTSSSTASLVWLKPEHDGGSRIRGYIVEFRIKGTDRWVVYGESKSQKMLVEGLIENTEYDFRVKAKNDAGISHPQGTHGSVVIKEPRIEPTADLSSISKQLITCKTSNTFTIDIPISGRPAPKVTWKLEEMKLKETDRVSIKTTKERTTLVVKDSKRSDSGKYYLTLDNAAGVKTFTVTVVVVGRPTPPTGPVEISGVSSESCTLSWSEPADDGGTDISNYIVEKRESGSASWQVVNSSVKRTTIKVTHLTKYMEYTFRVCAENKFGVSKSIESAAVVIEHPFVPPSPPSRPDVVSVSANAISIKWDLPYADGGSQVTGYWIEKKERNTILWVRENKLPCLECHYKVSSLIEGLEYQFRVYAMNIAGLSKASEASRPVMALNPVDPPGKPEVTDISRSSVSLCWTVPFNDGGSKIVGYIVEKKVYSTDEWDDNRWLKCNYTTITENYFTVTNLGEGETLEYRIIAKNAAGVHSAPSESTGPVTCKDEYSPPKAELDSKLARETVIVSAGSDLVLDGAVGGKPEPTVHWSKGEKILELGEKYSLTYTATKAMAVIKSCDRYDTGRYILTVKNASGTKTAAVNVKVLDTPGAPADKIVITRVTEEKCTVSWKIPLEDGGDSVTHYIVERRETSRLNWVIMETECKTLSCVSSRLIKNNEYIFRVRGVNKFGPGVALESDPIIARNAYTIPSPPSTPEVTAVGKEHVIIEWMKPESDGGSEIKNYIVEKREKSSTRWTRVNRTYTIYDTRLKITGLLEGSEYQFRVTAVNAAGDSHPSDTSPYILCKDPTYTPAPPSMPRITDTTKHSISMIWTRPMYDGGSDVTGYVVEILEEGSEQWYRATTKALKTNEYVAAGLAANKKYRFRVAAINNNGTGEFSDPSAEVEPLERIEMPDLELADDLKKTVCLRAGGTLRLFVSVTGRPTPVVAWRKTGVELQSRGFIETTDSYTSLIVEKVDRYDSGKYVLEAENPSGKKTVTILVKVYDTPGPPGSVKVKDYTKESVVITWDVPSIDGGAHVSNYIIEKRDANMKSYKTVTTECRKTLFRITGLEEGMHYFFRVLPENIYGIGEPCETAEAVLVCEVPSVPLNLHVIDVTKSSVTLHWERP**LHEGGSRLT**GYTIEACKVGTDRWSTVATVKSSVSQHTIQPLMENDQYLFRLRATNSRGASEPMDTVTPVTIQDIKVMPKIDMTNIPQKIVHVHRGKPIDLNIPIKAKPHPECSWSFAGVKLKDSLDRIKIDSNGKYTHLVIRETTINDTGDYTLEVKNAIGMATEVIKVIILDKPGIPVGPMKIEDTDGVSVTVSWEPPEKDGGANVGGYVVEQRDAHRPGWITISESVTRPCFKFTRLTEGTEYVFRAAAMNRFGVGGFLQSEVVECKSPKTIPGPPSRPEVLDVTHEGMTLTWQPPEDNGGSTIAGYIIERKEAHSDRWMKINKNPVTMTRYRSSGLIEGLEYEHRITAINSRGAGKPSESSEITVAMDPIEPPGCPVEPRVTDTTRTSVSLAWLPPEEEGGAVITGYLIEMQKVDQVEWTLCNTTPTKMCEYTLTHMPQGAEYKFRVIACNAGGAGEPAEIPGVVKVQEMLGYPDYELDHKYEEGYVVRQGGVIHLSVPIKGKPIPTCKWTKDGRDISHRAMIATHDDITELVIKEAHKDDTGTYDLVLENKCGRKAVYIKVKVIGRPDVPEGPLEFDDIQARSVRVSWRSPSDDGGSDILGYIVERREVPKAAWCTVDARVTETSLVVKGLKENVQYHFKISAENQFGVSRSLKSDEAVTPKTPLCPPEPPSNPPEIMEVTKTTVALSWARPRDDGGSRVTGYYVERREVSTEKWVRHNKTHITTTMYNVTGLIPDAEYMFRVVAQNDIGQSEPGPASESVVCKDPFDKPSQPGEIDIISVTKDCITIHWLRPEHDGGKEILGYWIEFRQAGESAWKKSNKERSKDRQFTMGGLMEATEYEFRIFAENETGLSRPRRTPMGIKTKLSVGEAPALKEDIKDVTTKLGESGTMTCGIIGRPLPEIKWYRYGKELIQSRKYKMSSDGRNHSLSVLTEEQEDEGVYTCRAINEAGEIETSGKLRLQAAPQFHPGFPLKDKYFAGAGTSLRLHVVYIGRPIPQIMWFYGKKPLNVSENVIIENTESYTHLVVRNVQRKTNAGRYKVQLSNKFGTVDTSLRVEIQDKPCTPEGPMVVEGLLKSSVIISWKPPKDDGGSMITNYIVEKREAKEGELWHLVSSSVSGNTCRVPNLTESAGYYFRVSAQNQYGVSEPLEIPSVLIIKSPFEKPGIPQQPFIMSSTNDSCVVCWKPPSSDGGAKITGYYLEKREKKQNKWMSVTTKKIAETNYEIKGLIEGFEYEFRVKCENMGGESDWSEISAPIIPKSEQAPRAPAFREEIRDMTVKYQANATFVTKVVGYPKPVVKWYRGGKEILADGTKIKAQEFKGGYYQLVIAAANENDATVYQVRATNSTGSISTTANLDVEVPAKIYLPKELQGMGAVHAARGDHITVKIPISGKPEPAVTWQKGQEILSNSAHHQVITTRSFTSLVFHKGVQRKDTGYYVITAKNRFGMDKQTIEVNVADIPDVPKALVVSNIGRDSITLTWEPPANDGGSDIIGYIVEKCPTTADRWIRAGQTTDCSITIINIFGKTKYQFRVIAENQFGLSHPSHPTEPITTKEDTSVIRNYDEEVDETREITKEEALFYKVKELSSKYTISEELARCQFGAVHRCVEIATKKTFMAKFIKVRGTDRELVLREIEALNVARHKNIIYLHEYFESMEEIILIFEFISGVDIFERLGTSNFELTEQEIVRYLRQVCSSLKFLHSHNYGHFDVRPDNIVYTTRKSTIIKIIEMGQARLLVPGENIRMLFTAPEYCAPEVHRHDLVTTATDMWSVGVMAYVLLSGLNPFAAESITKMIENISSCEYIFDSEAFKDISLEAMDFVDRLLVKDRKLRMTAHEALEHPWLRMKIELVSNKAIRTLRHRRYYQYLAKRIDTIVSSARIAYGGAFKNQRGLAVGKVKIGTEYQGLRAGPVMHGSAEEGGHVRFTCSISNYDKSTQVSWYFGNRQLHPSPKYEITYSNGFASIYVKDIEESDDGVYRCKVVSDDGEDSAYGELFVETVRSIRQHYISRSIKKLRRRVDKTKILQRPPEFTLPLYNRAAYIGEDVRFGVTITVHPEPQVAWLKNGERIKPGDDDSKYTFTSDKGLYQLMIHNLDMSDDAEYTVMAHNKFGEDSCQARLTVTPHPVSEETMRPMFKRLLANVDCTEGHSVRFELRVSGIPAPTLKWQKDGQPLQFGPKVVVIQEDVDYHVLHIRETLLEDSGVYKVTATNSTGSVSCQATLKVDRLTYTRREYKTEEEKYRHIQKQIEKTNKMAEHIAATEELVPLNPTAQEAIKFAAEMYKPAVSTKNVEGEFDITVHKSETKKLEEERRIFMPYEIPEPLHHDPTVLDEDKTIKQFVPLSDMKWYKKLRDQYEIPERMERIVQKRQRRIRLSRWEQFYVMPLPRITDQYRPRWRIPKLSLDDLETVRPARRSPSPESEVSFRSRRRSLGDLSDEELLVPVDDYLSRRRASAEKMMLEDELELGFSASPAISPVRIERRAVEHEERRHEVRHEAVEVSATKKKRTVSQFMRRRRSLSPTYIELMRPVSELIRPSRARPSVEVEVEGEIVERRSPTPERTRPRSPSPIRSVERSSRSSSRFERSARFDIMSRYEARKASLKAERKYQVVSQTPFSLDHAPRVTVRMRSHRIPLGQDTKFTLNIQTKPEAEIQWFHNGTVISESSEKYIFSNLSGVLSITILECQEEDSGTYRCVCSNSKGEASDYATLDVSGGGYTTFSSRRRDEDVPKAHVPEVTRIDHYHTTHFKAGYASQTHFEVEETKSKLTETREVVTRERYAASSERYSSAERYDSSVKYASTEYLSSGSSYSSDKFSLTGKHATSETKVKSSAAAVAEEVSVHKVKPSLSARILTKPQSVTVSEGETARFSCDIDGEPAPTVTWVHESRTLVSSHHVQVTTTQYKSSLEISSVTSSHEGSYTVIVENSAGRQEAHFTLTIRRAAPKEEVKAVKSPEPSVKSPTPSVKSPEPSVTSPTPSITSPVPSVKSPEPSIKSPEPSIKSPTPSVKSPEPSVTSPVPSVKSPTPSIKSPEPEGIKSPRSVKSPEPTSPAPGLKSPAPGVKSPEPEGIKSPRGFKSPEPRLKSPPPIKSPDRVKSPEPEGVKSPRGLKSPEPAGLKTPRGLKSPEPVGIKTPRGIKSPEPSGIKSPRALKSPEPEGLKSPPRMKSPPPIMSPKRVVSPPTVKSPIPKPPKVLSQVTAEAHEGSVRMSCVCESSVREVVWFVNGRRLSQSSHFEMHYSEGSCSLLIHDLADSDQGEYTCEMTSEGGVSKSSFSFTGQVFQSIRMKVTAYREQQLAVKGSMMMSHKEASSAMSSSMMMKKEVHTMEEASSFSSSSQQAMMSSMMESSSFSSMAAEMKFETMSMSSMSSMASESYAMSSSSLTEMASHMEGSSFRAIGSAPRIEALPEDISIEPGKVLTVACAFSGDAKHIEWSRGGRTIEVTAGGRFHIETTEDLTTLIITGVKEEDAGTYTLKLSNELGSDTAIVHISIRSV |
| TRINITY_DN55820_c0_g1::TRINITY_DN55820_c0_g1_i1::g.20194::m.20194 | gi\|923780003\|ref\|XP_013763892.1\| | PREDICTED: neurofilament heavy polypeptide-like isoform X5 [Pundamilia nyererei] | 71.1 | MAEADTSAAPSSSADGSNGAAAPQTTSPKSKGLGLLGKVKVSVELLIALAALLSWVVVGVVMFDFVEYKAVPDIQQIITDPVQAVNDAVDEVSSLLNKFQECAPDLSDPMSAATYAAEEISIAKDGFVQYFSDEEGNFYLSYIDPVVIGRQAFHSTNDFMGGVMGSFRDTLCAIVDTVLDTISDINKGIIDL**SYIDPVVIGRG**VFSVTNGTVGGIVGYIQDILCAILDSILDIVKGTTDISFIDPVVIGRNVFNVTNDFVSGVAGYVKDVLCAILDVIMDTVKGIQNAVGFNPMTVLQRTVEITTEQISMLVSYVSTTLFGEQGIVPEVPIDPMKVVEDAVLEFTDKKDLFVAYMSSM**IVGDQGEPAMPVVN**IVTEKDEAVASPSDVTLVRRKGEFLPPFEKDLKAAETRKEEKKEKEAKPEKTIEIKLKEEKLQTKTKPEAKPLEEKPKEEEVVKKLPKEKKEVRKPSKDEKKVKKPLKEEKVVTKLPKDKKEVKKPPKEEDDIKKPPKALKEAKKPPKDDKEVKKPSKDKKEVKKPPKEEKEIKKLPKEEKEVKKPPKEEKEIKKLPKEEKEVKKPPKE |
| TRINITY_DN31950_c0_g1::TRINITY_DN31950_c0_g1_i1::g.6433::m.6433 | gi\|221158258\|gb\|ACM07327.1\| | troponin I [Siniperca chuatsi] | 77.3 | MSHDKKMTSSRKHHLKSVMLAIAAGWLAQEKKDIAAAKQAYMAE**SCPAPSLGGDQATL**METCKKLHALIDKVDESRYDLQIKIGKGDKEIEDLRIKVVDLAGVKKPALKKVR**MSADAMLK**ALLGSKHTVNMDLRSNLKQVKKEVKEEPAEAVGDWRKNIEDKADRKKMFETS |
| TRINITY_DN58952_c4_g30::TRINITY_DN58952_c4_g30_i2::g.114965::m.114965 | gi\|657574621\|ref\|XP_008291025.1\| | PREDICTED: dehydrogenase/reductase SDR family member 7C-A-like [Stegastes partitus] | 68.8 | MALPFVVVLPLLIVVAAGVYYIYNEVMRFMSKSLVRNKVVVITDAVSGVGTECARLFHKGGARLILCGPSWDKLESLFDSLTSDADPKETFAPKLVILDFSDMDSMEDVVSEVLECYGGVDVLICNSSMKLKAPVQSVSLEADRNIMDVNYFGPSTLAKGVLPMMTSRRSGHIVLVNSIQGRLAVPFRSSYAASKHAAQAFFDCLRAEVEEFGIVVSTISHTFINASEPEPLAAAGPGPKPNFLAEFIVRQLTHGVRPSVLANEILQTVNRKRKEVVLAHPIPRVALCLRSFCPPFLFAVL**AAGVKDSVLAEQM** |
| TRINITY_DN50013_c0_g1::TRINITY_DN50013_c0_g1_i1::g.117766::m.117766 | gi\|958304908\|gb\|JAO51287.1\| | RS2, partial [Poeciliopsis prolifica] | 50.5 | SCYNDHQMADDAGGRGGFRGGFGAGGRGGRGRGRGRGRGRGRGARGGKSEDKEWVPVTKLGRLVKDMKIKSLEEIYLYSLPIKESEIIDFFLGSGLKDEVLKIMPVQKQTRAGQRTRFKAFVAIGDYNGHVGLGVKCSKEVATAIRGAIILAKLSIVPVRRGYWGNKIGKPHTVPCKVTGRCGSVLVRLIPAPRGTGIVSAPVPKKLLMMAGIDDCYTSARGCTATLGNFAKATFDAISKTYSYLTPDLWKETVFTKSPYQEFTDHLAKTHTRVS**VQRGQAVQAATS** |
| TRINITY_DN58121_c0_g3::TRINITY_DN58121_c0_g3_i1::g.89912::m.89912 | gi\|657583721\|ref\|XP_008295981.1\| | PREDICTED: titin-like isoform X20 [Stegastes partitus] | 61.5 | GCMITNYIVEKRESGSTAWQLINSSVKRTTLHVSHLTKYMQYTFRISAENRFGVSKSTESETIVAEHPFTPPGPPTKPSVFNVTANTMTLKWEEPYHDGGSKVTGYWIEKKERNNILWVRENKIPCFECYHKVEALVEGLEYQFRVYAMNSAGLSKASEASKGAVAQNPVDPPSKPEVTNVTRTTVSLKWSAPLNDGGSPIVGYIIERKPYTLTGEGRWLKCNYTNVTDQFYTVTALGEGEPYEFRVIAKNTNQVFSLPSESTGSVQCKTDFEPPKAQLDSKLMSETVMVRAGSDLVLDAAVGGRPDPKASWSKGNRDLELCEKYHLQYTSTKAMAIIKFCDRDDTGKYILTVRNVSGTKTAEVNVKVLDTPGVCEGSIEISKITEESCTLSWKPPVEDGGDDISHYIVERRDTNRLNWVIMNAECKELTCNISGLFKNTEYLFRVRGVNKYGPGVHLQSGPMIARNTFTVPTPPGAPEILASGKDFATIEWLKPESDGGSPLIHYLVERRERKSARWVKVNRDGAHLDSTLKVSGLTEGNIYQFRVTAINKAGESEASEVSLYVVCRVPTCTPAPPSIPRITDTHADSISLAWSRPVEDGGSDVMGYILEMQEAGAEEWSKAHEKTLR**TTEHVVTGLS**AGKKYCFRVAGININGTGDFSEPCAETEPVERIEPPDFELHDDLKKTICLRAGGSLRMFVHVTGRPTPAITWSKPGVDLHTRGFIEVNSSSTTLIIDKVHRYDAGKYTLVAENSAGKQEVNILVKVYDTPGPTGPLKIKELTKDSATISWEAPAVDGGAPVNNFIIERREASMRAYKTVTAKCSKTSYKIDGLMEGMLYYFRVLPENIYGIGEPSETPDVILVCEVPLPPNKLEVIDVTKSTVSLGWEKPEHDGGSRLTGYVIEACKFGTDKWMKVATLKLTDFEYTIEKLNEKEQYLFRIKAVNSRGASEPKELVTAITVQEQRVMPMVDFSSIPQKVVNVLAGKTLELDLPIIGRPPPVCSWYFHENKLKVTDRVKIKSTGKFSKLTMSDTTINDTGDYSLEVKNAVGVITEVIKVVVLSKPDEPSGPFRLDEIDATCVTCSWDPPARDGGAPISGYVVEQRDAHRPGWVPVSDSVSRPTFKFVNLIEGDEYVFRAAAVNRYGTGDFLQSEIVTCKSLKNVPGPPGRPVVFDVSRDGMTVAWEPPEEDGGLDISGYIIERKEVRSDRWVRANKNPVTMTRYRSTELIEGLEYEHRITAINARGQSKPSLCSKPAVATDPIDPPGCPQNPRITDTTKSSVSLAWSPPDDEGDARVDGYLIEMQKVGTMAWIKCNTTPSLICEYTLTKMPQGEEFKFRVMACNAGGSGEPAEVPGTVTVTEMLESPDYDLELKYKDVYVVRHGGVVRLSVPIKGKPHPTCKWLK**DSGAVSTKAMIA**STEDASELVIKGAERSDSGMYDLLLENRVGKKKAQIKVKVIGRPNAPEGPMVFEDIQANSVKVCWKAPTEDGGSEILGYIVERREATRNAWYTVDSRVTDTHLVVKGLREGTEYHFKVTAENSFGVSASLKSEEPLMAKTPLCPPEPSSTTPEIMDVTKSSVALAWSRPKDDGGSAITGYFVEYKLVSSETWSRYETKISSTMFTLPGLTPDAEYQFRIVAVNDIGESEAGPVSDPVTCKDPFEKPSQPGEIDTVNVTNNLIAIRWQAPECDGGKEVLGYWVEYRKSVESTWKKCNRDRLKQMEFTMRGLAEATEYEFRVFAENETGMSRPRRTVTCIKTKLSVETKPSLRKEMDEVTTKLGQPAVMKCQIIGRPVPEIKWYHAGKEIVESRKYEMSSDGRNHSLSIMTDQQEDEGEYTCKAINDAGDAETSGVLVLEAAPSFHPDYPLKDIYYAGLGTTLRIHAAYIGRPEPKIMWLHGAKTLENTDDISIETTEHYTHLVIKNVQRRVHGGKYRIRLHNHFGRADTPFTVEIYDKPDVPQGPIVLDALLKNSVIISWKPPKDDGGCMITNYIVEKREDKEGTEWELVSSSINGTSCRVPNLIDSAGYFFRVYAQNRYGNSEPLELTSPILIKSQLEKPSPPQSPVVSGITKDSCVVSWKPPLSDGGSKIKSYCLEKKHKKDKKEWTEWTPVTTDEIKQTVFSVKRLTEGVEHIFRVKCENLGGQSDYSEETTPMIPATAVDVRAPAFKEELRNMSVKYKSNATLVCKITGQPKPVIKWFRRGKEIHSDGKKIKIQEFKGGYHQLVITEADEEDSTVYQIRATNQGGSICATVSLDVEVPAKIHLPKNLKDKEAIPALRGEVVNIKIPFSGKPDPVITWQKGQDLIDSNGHYQVIVTRSFTALVFPNGVEKKDAGFYIVCAKNRFGIDQQTVELDVADVPDPPRGIKASDVSRDSVTLNWVAPANDGGSRVISYIIEKCPTTAERWERVAQSRDTRYTVINLFGGTSYQFRAIAENKFGQSAPSETSGPVMTKEDKSRVLLYDREVDDTGHVRKGKAPNSEAKNLHNKYAIAEELGRGQFGIVHRCVDISSEKTYMAKFVKVRGADQAIVKKEIATLNLAKHTSFLLLHESFDSPEELVMIYDFISGGDIFERLSSAEFELNEREVANYIRQICSALEFLHTQSYGHFDIRPENIVYTTRTSSNVKIIELGQSRHLTPGDQIKVQYTTAEYAAPEIHQCDMVSTVTDMWSVGVLAYVLLSGLNPFTAETNQQMIDNISNAAYSYDDESFTHVSVEALDFTDRLMTKDRKHRMSAAEALAHPWLTKPVEEISGRAIPTGRHKRYYQSMVRKEWSTVISAARVACGGSIRSQRGVFVAKVKIAPFEHGPLAGQVTHAVANEGDNVKFICNIDNYDSTTEVTWYCGVRQLEAGNKYEIDYEDGLAVITVSKVTRADDGTYRCKVVNEYGEDSAYAELFINGVRKYRDFFTTRVVKRTKRRVDTARMLQKPPEFTLPLVNRTAYIGEDVRFGVTITVHPEPRVMWHKSGQKLIPGQDDKKYTFITDKGLYQLIIHDLDKEDDAEYSVVARNRYGDDSCKSRLTVVPRPKPADLTLRPMFKRLLANVECREGQKVRFEIRVSGHPTLKWEKDGTPLAFGPSIEVVHEGLDYYILHVRDTLPEDSGVYRVTATNSAGSASCQATLKVERVAHVKKEYEPSQKEKEKAAKKEELDKKVRLSQILSGTVITPLPPAAVQAVREAATMFKPAVTTKKGEKTEAEIKKEQEERKKRADEKRLRMPYDVPAPRVINPAVLEEDVEIKHFKPFSDMKWYKKLRDQYEFPEPMDKIKQKRMKRIRLSRWEQFYEVPIRIKDQYKPKWRISSMTQDDLETVRPARHRTPSPEIDAYHRIRRRSLGDLSDEELLLPVDEYLSMKRTEEERLRLEEELELGYSASPPSLSPVRFELSALRPSSPRRAYSDDEEGEEVHRYDSYRIPSKYEAGPSFIDLRQRHDKTTHRPPREKQRVYAEREDQELLRPHKTAQRISSYKSELKRMEFEEKTRTTKQKSTVTVTAYSEGVESEHLTAFTSEYIPKPIKKLPTPEPERRRSPTPERAVKTDIVYPKVDFASRYEERKQALRSERRSVERKFEVVTQAPFSLDHAPRITIRLRSHRVPYGTNTRFTLNVQAKPEPEVKWFHNGKEIHQSHKYHMTNISGVLTLQIINCVTEDGGTYRVVCKSAKGETSDYATLDVAGEEYAAFSSLRRDEEPPSSHLPEMTRTEVYHVSSSKTTVRESVKETVKETTTVVERPRETPSVPAKILTKPLSLTVEEGDLARFECDVAGEPAPCITWMHEGAVIGSSARHHIVSTQYNSSFEISAVEMSDEGSYTLEVENAGGKQEAHFTLSIRKSESKEKVVAPQRVTSPEAKSPLAKSPEPVKSPQRVKSPEPVKSPQRVKSPVSPKSPTPKSPTPKSPTPKSPTPSEKERVTSPIKSPKRVMSPTIERKPTFSVGLSDVTANSDSIVKLSVKVTGEPRPTITWLKNGKALSQGGKYEIFEESGSVHLEIYESEVSDSGEYRCTAANSSGAVSTTCTVTVRASKISEEVVKQEMVHKEVVSSHSQMSITQGQSLTSDPAKRSDAPHFLLQPRSQIVDEGQNVIFTCEVAGEPSPEVEWLKDNVTISATSNIRLSCSQHVYTLEIREATVADTGKYTIKAKNQFGQCSATTSLNVISSPPKIEALPHDVSIEPGKSLKVEGLFSGDPAPSVEWVRSGRILPNGDERYRVENTSDLSTLVISAVKEDDAGAYTLRLNNELGSDSATVNIHIRSM |
| TRINITY_DN5796_c0_g1::TRINITY_DN5796_c0_g1_i1::g.101435::m.101435 | gi\|6230879\|dbj\|BAA86218.1\| | alpha hemoglobin A [Seriola quinqueradiata] | 57.3 | MSLSGKDKSIVKSFWDKVGGKAADIGADALGRML**VVFPQTKTY**FAHWADVGPDSAQVKKHGATIMAAVGDAVGKMDDLTGGLSALSELHAFKLRVDPANFRILAHNMLLVLAMYFPADFTPEVHTACDKFLQNLALALAERYR |
| TRINITY_DN3078_c0_g1::TRINITY_DN3078_c0_g1_i1::g.463::m.463 | gi\|961956273\|ref\|XP_014869118.1\| | PREDICTED: guanine nucleotide-binding protein subunit beta-2-like 1 [Poecilia mexicana] | 65.3 | M**TEQMTVRGTLKG**HSGWVTQIATTPQYPDMILSASRDKSIIMWKLTRDETNYGIPQRSLKGHSHFVSDVVISSDGQFALSGAWDGTLRLWDLTTGATTRQFVGHTKDVLSVAFSADNRQIVSGSRDKTIKLWNTLGVCKYTIQDEGHSEWVSCVRFSPNSSNPIIVSCGWDKMVKVWNLANCKLKTNHIGHTGYLNTVTVSPDGSLCASGGKDGQAMLWDLNEGKHLYTLDSGDTINALCFSPNRYWLCAATGPSIKIWDLEGKIIVDELRQEVISTNSKAEPPQCTSLAWSADGQTLFAGYTDNLIRVWQVTIGTR |
| TRINITY_DN57995_c0_g4::TRINITY_DN57995_c0_g4_i1::g.44197::m.44197 | gi\|657548411\|ref\|XP_008279112.1\| | PREDICTED: mitochondrial inner membrane protein isoform X2 [Stegastes partitus] | 63.7 | RGRSGATRASKRSKLRRRGRGSEGRKRKTRGERSQSVSQCSVCAGSSDHQCLKPPQRPRTMLRACLRGANATARKHCGRVPLNNLQHSRHYTTGGSSGGAAKVVVAGLLTVGGGVGGTIIYAKWDHKFRAAVESNVPYSDWVLGLALGPPSQDAGLPFKKQLEKAQPPSMLEKQMKAKAKSEKKAAEAAESSPVPTQPALS**LEEASTEATHIIS**AISEVPTVPAPCDTEAAAVKEECKECHDHTEAPVSAAVQPDPESAAAEPLRERPVEEVTARLAQQDLEEQDVVAAVSEGLEDSLSSSAKATLQAIGAQEAALQAITRHTLKLKEAMEAEVPPQEKSDQWKDLEAALTDRTSAVNDAQSALTKANEALDSLKSVIDKSKGLKVSAVRPLVLAAEENLHNMVVDLDKVITKVQSAESESKIVSQYSELVNEAKLQFQREVSSLTPEIQANWKGLTGKLSPDDLNALIAHAHRRIDQLNRELAEQRVREQIHMDGALEQQRLEDQKAQEKAVATALQHYKEDSRLEQERKLSELREVMEAEMRTQLRRQAAAHTDHVQDVLKVQEQELKSEAEQVLSSKMLEQETRYRQLSQEQLDNFTLDMNTAYARLKGVEEAIDSHVVAEEEARKAHQLWLSVEALNYALKTAEAGSPTAPLEGAAQ |
| TRINITY_DN47624_c2_g4::TRINITY_DN47624_c2_g4_i2::g.12600::m.12600 | gi\|657554793\|ref\|XP_008281877.1\| | PREDICTED: nascent polypeptide-associated complex subunit alpha isoform X1 [Stegastes partitus] | 85.5 | M**PGEATETVPVTE**QEMQQPQVETASSQQPQAASGPAKPKGKGAKNAQGSSAPKAVPGRRKRSSMSASSSSPTSPKSTPSTPLTPVISPLASSPASSSAQQANRSAPKVVKAGKQGKAKKGEEFVPAPTPQECKVTVAAEKPVEASPKQTVVEAKPAPAETKKPSPAAFKVTSKPAVAAPVSFSETLATSPPKSHVEVKVASPKAVAADDELPPLIPPEKPLKMPEIVPPAKVEVAVEAPKPAVEAKAAPKAKQEAAKPAVEVAKPAVEVAKPAVEVAKPVVEAKLAPVEAPKAKQEAAKPAVEVAKPAVEVAKPVVEAKLAPVEAPKAKQEA |
| TRINITY_DN46549_c6_g3::TRINITY_DN46549_c6_g3_i1::g.96573::m.96573 | gi\|379998736\|gb\|AET79258.2\| | parvalbumin 1 [Siniperca chuatsi] | 95.4 | MAFASILKDADITAALAACQAADSFKYKEFFAKVGMAAKSADDIKKAF**AIIDQDKSGFIEED**ELKLFLQNFC**AGARALSDAETKAFLKAGDSDGDGKIGVDEFAAMVKA** |
| TRINITY_DN29852_c0_g3::TRINITY_DN29852_c0_g3_i1::g.5844::m.5844 | gi\|657528877\|ref\|XP_008292573.1\| | PREDICTED: proteasome subunit alpha type-1 [Stegastes partitus] | 58.9 | MFRNQYDNDVTVWSPQGRIHQIEYAMEAVKQGSATVGLKSKTHAVLVALKRAQSELAAHQKKILNVDNHIGISIAGLTADARLLCNFMRQECLDSRFVFDRPLPTSRLVSLVGSKTQIPTQRYGRRPYGVGLLIAGFDDMGPHIFQTCPSANYFDCKAMSIGARSQSARTYLERCMDKFSDCNLNDLVQHGLRALRETLPTEQDLTTKNVSIGIVGKEMEFTIYDDDDVAPFLEGLEERPQRKVAQPADEPAGD**AAPAPDEPMEH** |
| TRINITY_DN51108_c0_g7::TRINITY_DN51108_c0_g7_i1::g.82614::m.82614 | gi\|657588840\|ref\|XP_008298779.1\| | PREDICTED: creatine kinase S-type, mitochondrial [Stegastes partitus] | 81.3 | RDWPDARGIWHNNEKTFLIWINEEDHTRVISMEKGGNMKRVFDRFCKGLKQVEHLIQERGWEFMWNEHLGYV**LTCPSNLGTGLR**AGVHVRLPKLSKDPRFPKILDNLRLQKRGTGGVDTAAVGDTFDISNNDRLGKSEVELVQM**VVDGVNYL**IECEKRLEKGQDIKVPAPVSQFRK |
| TRINITY_DN48294_c0_g1::TRINITY_DN48294_c0_g1_i1::g.55880::m.55880 | gi\|584018877\|ref\|XP_006804149.1\| | PREDICTED: ATP synthase subunit beta, mitochondrial-like [Neolamprologus brichardi] | 69.3 | MLGAVGRCCTGALQALKPGVQPLKALVGSPAVLSRRDY**VAPAAAASVANGRIVA**VIGAVVDVQFDEGLPPILNALEVKGRESRLVLEVAQHLGENTVRTIAMDGTEGLVRGQKVLDTGAPIRIPVGPETLGRIMNVIGEPIDERGPISTKQTAPIHAEAPEFTDMSVEQEILVTGIKVVDLLAPYAKGGKIGLFGGAGVGKTVLIMELINNVAKAHGGYSVFAGVGERTREGNDLYHEMIESGVINLKDTTSKVALVYGQMNEPPGARARVALTGLTVAEYFRDQEGQDVLLFIDNIFRFTQAGSEVSALLGRIPSAVGYQPTLATDMGTMQERITTTKKGSITSVQAIYVPADDLTDPAPATTFAHLDATTVLSRAIAELGIYPAVDPLDSTSRIMDPNIVGSEHYDVARGVQKILQDYKSLQDIIAILGMDELSEEDKLTVARARKIQRFLSQPFQVAEVFTGHMGKLVPLKETISGFQSILGGEYDALPEQAFYMVGAIEEVVQKAEKLAEEHS |
| TRINITY_DN50937_c0_g6::TRINITY_DN50937_c0_g6_i1::g.37905::m.37905 | gi\|974096226\|ref\|XP_015246606.1\| | PREDICTED: glycogen [starch] synthase, muscle-like [Cyprinodon variegatus] | 48.5 | MPLARSLSVTSLSGLEEWDEEFDLEDAVLFEIAWEVANKVGGIYTVIQTKARLTSEEWGENYFLVGPYVESNVRTQVELIEPTNPVLKRTIDKMNSSGCKVYFGRWLIEGSPYVVLIDVGFTAWSLDQWKKELWDLCDIGVPWFDREANDAVLFGFLTAWLLGEYAAQCEEPPHIVAHFHEWLAGLGLVLCRHRQLPIATIFTTHATLLGRYLCAGNVDFYNKLSEFNVDKEAGDRQIYHRYCLERAAAHCTHVFTTVSQITAIEAEHLLKRKPDIITPNGLNVKKFSAMHEFQNLHAQSKNRIQEFIRGHFYGHLDFNLDKCLFLFIAGRYEFSNKGADVFLEALARLNYLLRVNHSDVTVIAFFIMPARTNNFNVETLKGQAVRKQLWDTAHTVKERFGKKLYESLLVGQLPDVSKMLDKEDFTIMKRAIFATQRQCQPPICTHNMLEDSSDPILNCVRRIGLFNSSADRVKIIFHPEFLSSTSPLLPMDYEEFVRGCHLGVFPSYYEPWGYTPAECTVMGIPSVSTNLSGFGCFMEEHIADPSAYGIYILDRRDRAVDESCNQLTSFLFQFCKQSRRQRIIQRNRTERLSDLLDWRYLGRYYIAARHMALAKAFPDTYLYEPHEPSSASGFRYPRPASVPPSPALSRHSSPHHSEAEDDVDEDEDERYDEDLEAEKDRVNIRQPYTPPYKNKSLLG**ANGNGVISEKN** |
| TRINITY_DN49351_c1_g1::TRINITY_DN49351_c1_g1_i1::g.75759::m.75759 | gi\|657554845\|ref\|XP_008281906.1\| | PREDICTED: poly(rC)-binding protein 2-like isoform X2 [Stegastes partitus] | 79.3 | MDSSMVEGGLNVTLTIRLLMHGKEVGSIIGKKGESVKKMREESGARINISEGNCPERIITLAGPTTSIFKAFSMIIEKLEEDISTSMTNSTATSKPPVTMRLVVPASQCGSLIGKGGCKIKEIRESAGAQVQVAGDMLPNSTERAITVAGTPQSIIECVKQICVVMLESPPKGVTIPYRPKPSGSPVIFAGGQAYAVQGQHAIPQPDLTKLHQLAMQQSPFPIAHSNQGFQAGMDASAQTGSHELTIPNDLIGCIIGRQGAKINEIRQMSGAQIKIANPVEGSTDRQVTITGSHASISLAEYLINA**RLSSEATGLAAN** |
| TRINITY_DN51688_c1_g1::TRINITY_DN51688_c1_g1_i1::g.38040::m.38040 | gi\|657588631\|ref\|XP_008298664.1\| | PREDICTED: retinal dehydrogenase 2 [Stegastes partitus] | 62.9 | M**TSSKIEIPGEVK**SDPAALMASLQLMPSPVPNPEIKYTKIFINNEWQDSVSGKVFPAYNPATGEQICEVQEAEKADVDKAVQAARLAFSLGSVWRRMDASERGRLLSKLADLVERDSVYLATIESLNSGKPFLPTLFVDLQGTIKTLRYFAGYADKIHGTSIPMDGEYLTFTRYEPIGVCGQIIPWNFPLMMTAWKLGPALACGNTVVLKPAEQTPLTCLYMAALIKEAGFPPGVINILPGFGPTAGAAIASHMGIDKVAFTGSTEVGKLIQEAAGKSNLKRVTLELGGKNPNIIFADADLDLAVEQAHQGVFFNAGQCCTAGSRIYVEEPIYDEFVRRSVERAKRRIVGSPFDPTTEQGPQISREQQNRVLEFIQSGISEGAKLECGGKALGLKGFYIEPTVFSNVGDDMRIAREEIFGPVQQLMKFKTIEEVIERANNTDYGLVAAVFTNDINKAMTISTAMQAGTVWINCFNALSTQCPFGGYKMSGNGRELGESGLKEYSEVKTITMKMVAKNS |
| TRINITY_DN58283_c0_g37::TRINITY_DN58283_c0_g37_i1::g.44842::m.44842 | gi\|734645632\|ref\|XP_010751918.1\| | PREDICTED: methionine aminopeptidase 2 [Larimichthys crocea] | 66.9 | FPAASAIM**ADVVAEQAAEQK**AAPDRDREQLNGEAEDREEADPVETAKKRKKKKKKKSAATTGAEPEADGVGEVTKQLEKQAIEDKEKEEDGEEDGDDGENSAGKKKKKKKKKKGPKSQTDPPSVPICDLYPTGVFPIGQECEYPASQDGRSAAWRTTHEEKRVLDKANEEVWNDFRQAAEAHRQVRQHVRSFMKPGMTMIEICERLEDCSRKLIKENGLNAGLAFPTGCSLNHCAAHYTPNAGDTTVLQYDDVCKIDFGTHINGRIIDCAFTVTFNPKYDKLLEAVRDATNTGIKNAGIDVRLCDVGESIQEVMESYEVELDGKTYQVKPIRNLNGHSIGQYRIHAGKTVPIVKGGEATRMEEGEVYAIETFGSTGKGVVHDDMECSHYMKNFDVGHVPIRLPRAKHLLNVVNENFGTLAFCRRWLDRLGESKYLMALKNLCDLGIVDPYPPLCDTKGCYTAQFEHTILLRPTCKEVVSRGDDY |
| TRINITY_DN5925_c0_g1::TRINITY_DN5925_c0_g1_i1::g.71521::m.71521 | gi\|56565283\|dbj\|BAD77969.1\| | type 1 collagen alpha 2 [Paralichthys olivaceus] | 89.8 | MLSFVDTRILLLLAVTSYLASCQYSGPRGDKGPRGDMGPKGPDGKDGKPGLPGPAGPPGPPGLGGNFAA**QYDGVKAPDPGPGPMG**LMGPRGPPGPPGPPGPQGHTGHAGEPGEPGQTGALGPRGPSGPPGKSGEDGNNGRPGKPGDRGTPGPQGARGFPGTPGLPGMKGHRGYTGLDGRKGEPGQTG |
| TRINITY_DN50943_c0_g1::TRINITY_DN50943_c0_g1_i1::g.37726::m.37726 | gi\|617417706\|ref\|XP_007557445.1\| | PREDICTED: 60S ribosomal protein L3-like [Poecilia formosa] | 49.8 | MSHRKFHAPRHGHLGFLPHKRSKKHRGKVRTWPKDDPSKPVHLTAFPGYKAGMTHTLREVHRTGLKQSKREDVEAVTIIETPPVIVVGIVGYIHTIRGLRSLKTIFAEHLSDECKRRFYKSWYKSKKKAFTKYSKKWQDEAGKKQLDKDFTMLKKYCSTIRVIVHSQMRLLPIKQKKAHIMEVQLNGGSISDKVDWAREHLEQAVPISSVFYQDEMIDVIGVSRGHGFKGVTSRWHTKKLPRKTHKGLRKVACIGAWHPARVAFTIARAGQKGYNHRTELNKKIYRIGRGVHIQDGKVIRNNASTSYDTSQKTITPMGGFPQYGDVNNDFVMVKGCVVGTTKRVLTLRKSLLVHTSRKSKETIELKFIDTTSKFGHGRFQTAQEKRAFMGPLKKDVK**KTLPEPLSEDV** |
| TRINITY_DN36769_c1_g1::TRINITY_DN36769_c1_g1_i1::g.32743::m.32743 | gi\|765111561\|ref\|XP_011473251.1\| | PREDICTED: 60S ribosomal protein L9 isoform X1 [Oryzias latipes] | 80 | RHVRALQQRRDVLPSSFPLLPARMKTILSSQTVDIPDNVEIRLKGRTVIVKGPRGKLVREFNHINLELSLLGKKQKKLRVDKWWGNRKELATVRTICSHVQNMIKGVTLGFRYKMRSVYAHFPINVVIQENGTLVEIRNFLGEKYIRRVRMRTGVVCTVSAAQKDELVLEGNDVELVSNSSALIQQATTVKNKDIRKFLDGIY**VSEKGTVVEGDQ** |
| TRINITY_DN32831_c0_g4::TRINITY_DN32831_c0_g4_i1::g.31895::m.31895 | gi\|974097077\|ref\|XP_015247066.1\| | PREDICTED: ATP synthase subunit delta, mitochondrial [Cyprinodon variegatus] | 73.1 | MMAARFLRRALPVVRHARSY**AEAASGAPQMSFT**FASPTQVFFKEASVKQVDVPTLTGAFGILPAHVPTLQVLRPGVVTVFSDDGSAAKYFVSSGSVTVNADSSVQLLAEEAVPLDQLDVAAAKANLEKAQSELAGASDEAARAAVQISIEANEAIVKALE |
| TRINITY_DN54863_c2_g1::TRINITY_DN54863_c2_g1_i1::g.41310::m.41310 | gi\|657558875\|ref\|XP_008283410.1\| | PREDICTED: reticulon-3-like isoform X1 [Stegastes partitus] | 69.1 | **MDPMTQSAQISSSQGLADGQN**SAAKESKLSDSFLSSSPVSLIQSPQVKDLIHWRDPKKSGLVFGVSMLMLLSLAAFSVISVVSYLLLALLCVTITFRIYKSVVQAVQKSNEGHPFKTLIDKDVSIPPETFRKHVDASLTYINRALKQMSRLFLVEDLVDSLKLAVVMWLLTYVGAVFNGITILILADILLFALPPVYEKNKTQIDQYIDVARTQVNTTMAKLQEKLPGAVKRSKTEM**GDPATLVEPTTPAVVT**AIPTAVAGETQDGNKTQSANKNGKKADSGSSGSFFNWFIVLALLGVWTSVAVVYFDLVDYQGVLGKLVAYDTDGDGDFDVEDAKVLLDEKPGDAARGGAKREIFPKDASKNLREALKQQLAIIHERVEAKKLAKLALAEVRQLLAKEEEEKALELGRQEIRGRVQDRVAARLKEEEEKIEKEEMEKAVEKLRKEKAKQQDQQKEESEEEEEKSKKGEKKVEGEGKKAQEDKGRGKETTKEKKKKSKAEKSDKGKK |
| TRINITY_DN53268_c1_g1::TRINITY_DN53268_c1_g1_i5::g.16444::m.16444 | gi\|930771688\|ref\|XP_005950973.2\| | PREDICTED: fibrous sheath CABYR-binding protein-like [Haplochromis burtoni] | 62.5 | M**GDPATLVEPTTPAVVT**AIPTAVAGETQDGNKTQSANKNGKKADSGSSGSFFNWFIVLALLGVWTSVAVVYFDLVDYQGVLGKLVAYDTDGDGDFDVEDAKVLLDEKPGDAARGGAKREIFPKDASKNLREALKQQLAIIHERVEAKKLAKLALAEVRQLLAKEEEEKALELGRQEIRGRVQDRVAARLKEEEEKIEKEEMEKAVEKLRKEKAKQQDQQKEESEEEEEKSKKGEKKVEGEGKKAQEDKGRGKETTKEKKKKSKAEKSDKGKK |
| TRINITY_DN99037_c0_g1::TRINITY_DN99037_c0_g1_i1::g.27711::m.27711 | gi\|657534560\|ref\|XP_008274138.1\| | PREDICTED: 40S ribosomal protein S25 [Stegastes partitus] | 78.1 | MPPKQDKKKDTGKSKKDKDPVNKSGGKAKKKKWSKGKVRDKLNNLVLFDKATYDKLYKEVPNYKLITPAVVSERLKIRGSLARNALQELLAKGMIKLVSKHRAQLIY**TRNTKGGDEEAAAEKA** |
| TRINITY_DN51612_c0_g2::TRINITY_DN51612_c0_g2_i1::g.58145::m.58145 | gi\|974088169\|ref\|XP_015242208.1\| | PREDICTED: cold-inducible RNA-binding protein isoform X4 [Cyprinodon variegatus] | 57.6 | MSDEGKLFIGGLSFETNEESLAAAFGKYGTIEKVDVIRDKETGRSRGFGFVKYDNAEDAKDALDAMNGKTLDGRAIRVDEAGKGGRSRGGFGSGPRGGRFSGSRGRGGRGYSRDFGGGGYNGDRGYGDRSYGDRSFGGGERSFGGGGGGGYRSGGYSSGGGGGYRENRGQGGYGDRSGSYR**DGYDSYATHE** |
| TRINITY_DN98383_c0_g1::TRINITY_DN98383_c0_g1_i1::g.110710::m.110710 | gi\|765133271\|ref\|XP_011478710.1\| | PREDICTED: rho guanine nucleotide exchange factor 9 isoform X3 [Oryzias latipes] | 8 | LGSPLQNRDQMRANVINEIMSTERHYIKHLKDICEGYLRQCRKRVDMFNDDQLKVIFGNIEDIYRFQMGFVRDLEKQYNTEEPHLSEIGPCFLEHQDGFWIYSEYCNNHLDACMELSKLMRDGRYQHFFEACRLLQQMIDIA**IDGFLLTPV**QKICKYPLQLAELLKYTAQEHSDYRYVAAALAVMRNVTQQINERKRRLENIDKIAQWQASVLDWEGDDILDRSSELIYTGELSWIYQPYGRSQQRVFFLFDHQLVLCKKDLIRRDILYYKGRIDMDRYEVRDAIDGRDDDFNVSVKNAFKLCNKDSEEIHIFLAKKPEEKIRWLRAFHEERKMVQEDEKIGFEISEYQKRQAAMTVRKVTKQKGEATRRYQV |
| TRINITY_DN51167_c0_g5::TRINITY_DN51167_c0_g5_i1::g.70931::m.70931 | gi\|658878547\|ref\|XP_008422265.1\| | PREDICTED: NADH dehydrogenase [ubiquinone] 1 alpha subcomplex subunit 8 [Poecilia reticulata] | 16 | M**PTTVEAPTLQEL**KVDEVNVSSAVLKAAAHHYGSQCDKPNKEFMLCRWEEKDPRKCLEEGRKVNECALNFFRQIKGNCAESFTDYWTCLDYSNLGELRHC |
| TRINITY_DN57119_c1_g11::TRINITY_DN57119_c1_g11_i1::g.125802::m.125802 | NA | NA | 17 | MMSYRCGCCVSFRFMSMSSPSGVKPWRRRRWRTASLRTVKTLRVNPHQSDCTTTLTVSVSVAVVVR**LCVAAVVVVVSDDDVVVRR**TKARQTLLSFRLDRSVWTVRCYGFRVV |
| TRINITY_DN55199_c5_g1::TRINITY_DN55199_c5_g1_i1::g.18894::m.18894 | gi\|548429331\|ref\|XP_005743648.1\| | PREDICTED: ankyrin repeat domain-containing protein 1 [Pundamilia nyererei] | 35.5 | MGLHSVEELVTGKRPEGKESEDFKGGVYEAAVSQEKRDDITGVGGALAEEEVSVAALNTDKSGRLKLETVDDLFNILQLKKRRRERKSPVHKKQQQQPEPLPETVDEQSFLTAAMENKLPVVEKYLRDGGKADTADHFQRTALHKASFRGHLEVMRRLLEAGAAIDKKDKLEATAVHWACRGGSLPALQLLLDQGAKFTSRDKLLSSPLHVAVRTGHCECAEHLIHCGADVNAKDRDGDTPMHDAVRINRFKMIKLLMMYGASLTTKNSDGKTPLETLNSWQNGAKSL**LCNFSEEKTNQ** |
| TRINITY_DN46381_c0_g4::TRINITY_DN46381_c0_g4_i1::g.81824::m.81824 | gi\|583989691\|ref\|XP_006790048.1\| | PREDICTED: NADH dehydrogenase [ubiquinone] iron-sulfur protein 4, mitochondrial-like [Neolamprologus brichardi] | 76.6 | LFLRGSRTSLLTTVMASSMSLLGLGRLTVINAASKALLNPIRSTSTSTLRL**AEKPGQDTQL**ITVDEKLDITPVTGVPEEHIKTRKVHIFVPAKTAMQSGVNSTKKWKMDFDTRERWENPLMGWASTGDPLSNMLLSFSTKEDAIAFAEKNGWSYDVTDKRSSKPRVKSYGANFSWNKRTRRSAK |
| TRINITY_DN6726_c0_g1::TRINITY_DN6726_c0_g1_i1::g.100649::m.100649 | NA | NA | 59.8 | IIIII**IIIIIIIMI**SPAELLMKIFSMMQMKTVDRCELSHFKSQCRSLNSTPNAVFPCSSSSESSAAVQPVKLCTALRPDHSVCSAAILTRLSSRVMKQLLFSPTSTSPKPMSEVRTLMGPIAPFMWT |
| TRINITY_DN47499_c0_g3::TRINITY_DN47499_c0_g3_i1::g.36137::m.36137 | gi\|657524951\|ref\|XP_008278859.1\| | PREDICTED: dnaJ homolog subfamily C member 8 [Stegastes partitus] | 38.6 | M**AAAGGESSQPVSDEL**FQNFYTEVKQIEKRDSVLTSKQQIDRLLRPGASYFNLNPFEVLQIDPEATDDELKKRFRALSILVHPDKNQDDPDRAQKAFEAVDKAYKLLLDPEQKKRALDVIHAGKEYIEHMVKEKRKQLKKEGKMIDVEEDDPEMFKQAVYKQTMKLFAELEIKRKEREAKDMHERKRAREEEIEQAEKAKRDREWQKNFEETRDGRVDSWRTFQAKGKTKEKKNRSFLKPPKVKMEQRE |
| TRINITY_DN54183_c0_g3::TRINITY_DN54183_c0_g3_i5::g.59066::m.59066 | gi\|657737581\|ref\|XP_008324265.1\| | PREDICTED: mucin-5AC-like [Cynoglossus semilaevis] | 52.9 | QACTHWLPCAGLQPALTHPLHVRKGSQSISSPSLSKGSAKISISSGARTPFPFATQSKSEGEVRRVHSHRIIMKTNRVLLLLLLAFTPVSPSASDESTLPTSSVSPNNGAAPQLPSVSPNNGTASPATQDGTTQTAPSNKGTVRPTILATLPATTQVGATSSNNKSAESTQTPPVGTAPTPVNQTNKDESIDPQGNRPSNNPTAVNTTSSTIPGTVKANPTGNATPGTGTKQSPPVKENDAGSQTGGDKPAEPKPGNKLLWILLPVTLLAAAAVGFYLKFKSKRVNDCTETIDTGTENASFQSRPESTKDGVMLL**GVKSSGAEENAAAR** |
| TRINITY_DN47384_c0_g1::TRINITY_DN47384_c0_g1_i1::g.12259::m.12259 | gi\|410904401\|ref\|XP_003965680.1\| | PREDICTED: electron transfer flavoprotein subunit beta [Takifugu rubripes] | 63.8 | YHPKNRPTSPPWVKVHCGDCVTSTSGKLNVSSTMSGRVLVGVKRVIDYAVKIRVKPDKSGVVTDGVKHSMNPFCEIAVEEAVKLKEKKLIKEVVAVSCGPQQAQETIRTALAMGADRGIHVEVTGKDYDTLGPLQVSKIMAALAKKEEAQLVIL**GKQAIDDDCNQTGQMTA**ALLDWPQGTFASEVAMEGDKVKVVREIDGGLETIKINTPAVVTADLRLNTPRYATLPNIMKAKKKKIANVKPADLGVDLTSRLEVLRVDEPPQRQAGVKVETVDDLVGKLRETGRI |
| TRINITY_DN1904_c0_g1::TRINITY_DN1904_c0_g1_i1::g.68284::m.68284 | gi\|736253243\|ref\|XP_010788812.1\| | PREDICTED: sodium-dependent multivitamin transporter [Notothenia coriiceps] | 35.8 | ATFQSAVAILGAPSEIYTFGTQYWFLGCSYFLGLLIPAHIFIPVFYRLRLSSAYEYLELRFNKTVRICGTVTFIFQMVIYMGVVLYAPALALNAVTGFDLWGAVLAMGLVCTLYTALGGLKAVMWTDVFQTVVMFAGQLAVIVVGASQ**AGGMGEVWRKAI**NG |
| TRINITY_DN38385_c0_g1::TRINITY_DN38385_c0_g1_i1::g.67027::m.67027 | gi\|657530103\|ref\|XP_008296779.1\| | PREDICTED: succinyl-CoA ligase [ADP/GDP-forming] subunit alpha, mitochondrial [Stegastes partitus] | 71 | MSHSRLFARLLLQQSGVRHCYTGSRKHLYITQNTKVICQGFTGKQGTFHSQQSIDYGSQLVGGVSPGKGGKTHLGLPVFNSVKEAREGTGADATVIYVPPPFAAAAIIE**AIDAEMPLVVC**ITEGIPQQDMVRVKHKLLRQGTTRLIGPNCPGVINPGECKIGIMPGHIHKKGRIGIVSRSGTLTYEAVHQTTQVGLGQSLCIGIGGDPFNGTNFIDCLEVFLQDPKTEGIILIGEIGGNAEENAAEYLKQHNSGANSKPVVSFIAGLTAPPGRRMGHAGAIIAGGKGGAKEKIAALQSAGVVVSMSPAQLGSTMFKEFEKRKML |
| TRINITY_DN58865_c0_g4::TRINITY_DN58865_c0_g4_i1::g.24111::m.24111 | NA | NA | 81.7 | AV**TRLLHAENPPD**PRHHLVGRRVGRFVQVDDARPDVVADVPLQRVAAVGQRRVVSRPHVQFVEVLEEEGPLGRIQGNDLRLRLDEEVAALLQLPDFFITRPFRLFLLLC |
| TRINITY_DN44769_c0_g1::TRINITY_DN44769_c0_g1_i1::g.69910::m.69910 | gi\|348534170\|ref\|XP_003454576.1\| | PREDICTED: protein deglycase DJ-1 [Oreochromis niloticus] | 85.2 | MAGKKALVILSKGAEEMETVIPVDVMRRAGIAVTVAGLTGKEPVQCSRNVVICPDSSLEEASKQGPYDVVLLPGGMPGAQNLAESPAVKEVLKDQDGRKGLIAAICAGPTALLAHGIGYGSTVTTHPAMKEKMMAGDHYKYSEARVQKDGHYITSRGPGTSFEFALTIVEELLG**AEVAAQVKAPLVM**KD |
| TRINITY_DN55256_c0_g1::TRINITY_DN55256_c0_g1_i1::g.84956::m.84956 | gi\|657558036\|ref\|XP_008283118.1\| | PREDICTED: voltage-dependent anion-selective channel protein 3 isoform X2 [Stegastes partitus] | 79.8 | M**AEKEVRNMVQ**QEKTGKVKQAENKDCCVSCHHVPKGHGTMAVPPSYSDLGKSAKDIFNKGFGYGVLKLDVKTKSQSGVEFTTSGSNNTDTGKSGGHLETKYKVKELGLSFNQKWNTDNTLTTEVTMEDQLAKGLKLSLDTSFVPNTGKKSAKLKTGYKREFVNMGCDLDFDMAGPTVHAAAVLGYEGWLAGYQLAFDTAKSKLTQNNFALGYKAGDFQLHTNVNDGTEFGGSIYQKVDSNLETAVHLAWTAGSNNTRFGIGAKYQLDKDASLSAKVNNACLVGVGYTQTLRPGVKLTLSGLIDGKNVNGGGHKVGMGFELEA |
| TRINITY_DN50560_c0_g3::TRINITY_DN50560_c0_g3_i1::g.58047::m.58047 | gi\|734632941\|ref\|XP_010744930.1\| | PREDICTED: NADH dehydrogenase [ubiquinone] iron-sulfur protein 5 [Larimichthys crocea] | 39.6 | M**PFVDVQSRLG**INLDRWLLLQSGEQPNKRASRCHAFEKDWVECSHGIGQTRAKKECRLEFEDFYECMHRQKTHERLHAIRKQRDKMVKEGTYTPPACHSGKEDQSP |
| TRINITY_DN56216_c0_g3::TRINITY_DN56216_c0_g3_i1::g.86577::m.86577 | gi\|734602928\|ref\|XP_010728444.1\| | PREDICTED: myelin basic protein-like isoform X2 [Larimichthys crocea] | 47.2 | M**ASASSAQTTFGLG**RRKKNPGLLDQIGKFFGGDKKRKSKGSFRGALSPAPQKASATSPRKRAGENAVVHFFRTIGDQKSKKASGDGKGTLTRIFKMGSRSASPAKR |
| TRINITY_DN38699_c0_g1::TRINITY_DN38699_c0_g1_i1::g.56447::m.56447 | gi\|908504578\|ref\|XP_013127347.1\| | PREDICTED: PDZ and LIM domain protein 5 isoform X4 [Oreochromis niloticus] | 62.9 | M**SSNYSVTLK**GPAPWGFRLQGGKDFNMPLTLSRLTDGGKATKAGMAVGDMVLSIDGIATDGMNHLEAQNKIKSCTDNLTLTLQKASSVPKPPPVAPKTAAHHQVTKVPRKHVVETD |
| RRRRRTRINITY_DN6653_c0_g1::TRINITY_DN6653_c0_g1_i1::g.127620::m.127620 | NA | NA | 15.5 | LDRWLTSSQTLSISLRPSSPRGEIKPPEPLPLSKVSTISGTHGLSHPHPVPPIPPLPRRAPPPSPAPSSTNDECVSLVDSRSQRLIEFNQAAESYQQYLQAANFFRSNRKRREITDLVNRLEVEGWSKSRDDEEETTSFIRTRRDRAVGEQDEVEVLEEGRRRRETTKRIGRRRMRA**EPGLDRFSC**TSMLTGSGVPREAVKGGERSDAELEEGEEAVRGVEVAADRDDPEGGQREEESEGVIEEEEQSEGERSPTDSDYNLIPCSNPKYLTNSVSLYMEVEKQGSVEKE**IESLTIENGG**EESVRIEADAKELHLHDPPETEKDTSHEEQGETDLPTDAHTHTPTDGHMATCQDAHMDAQNQGMGRLGVVVLPEEDELEPESHQEDS |
| RRRRRTRINITY_DN44929_c0_g1::TRINITY_DN44929_c0_g1_i1::g.10843::m.10843 | NA | NA | 17.9 | AAAAAATAASKKDDEESDSGGSSSGDSAATPPKKGEGVPWAGPCAGGADSKEHNQKAQKDTAGSASDVPSSCRPNTETGSFPQFETFNAWGSGQTEAGGGASSDWGAQTQSSATGGSERFNATWGPGTESTNQESDEDEDSESGRDQERGGNEMSSRSSGESTQSVGFRTRSKAQTAYNIEKEEWIDEEEEADDFQRIREKCCAEFAAANASNDDADLNFNIEAIRDFTANINEDHESFEEDNFGFQDVFNATMQQIQYDSFAQQLSLDNPFPSEMDDDESSSHMNHTSVLEVTNRRNTERLTEDVFSEWRGRCDEPLEKILDSIQAQVPGKELNQ**VVMNAIRT**LHGMNGRRTGGESQIRDNEEWADLIRQVLRCKQFLHAVLANHISTQPDTTRGTEVAEAQGESGSAAPKGDHNQLGPSPREESSPHNLIAAVCLEVQFHLFNNWSYKFFLDLLLDMTALNCLEQCISPANTQLLAAVLRAVHLRANGLPQELVGVTTLIASKKPPDLLLQQFDKLHPEIANLISSNVTYSREYGQSYLDMLGELGSRRTELLTLLVQTGNVISAESREGEFLNKLLGAVSEQSELVALLPDAEVNEQMQNAQDRSLRIIDCLTQSANSQREEDKGTHILETLRQVLKEENLWHLVEQRLPAPEVCSILRLLLDMMASADIHKLVLGIFGEKKKLFTIVQETKRAILNGITKSFFSALLPNLPPDQELFHYLMELLSDDAGLKDNIISVDSTLLECAINPFKFRSREELDAPPETTILSVLEQMCHDQSLFLLLRRNQAKCEQLVDDEEMLERLTVDERDLLQDIHSTTHLDFKWFM |
| TRINITY_DN54181_c0_g1::TRINITY_DN54181_c0_g1_i1::g.59189::m.59189 | gi\|657578396\|ref\|XP_008293067.1\| | PREDICTED: aspartyl aminopeptidase isoform X1 [Stegastes partitus] | 61.2 | MQSVIMKSSREAVQSAAKEFLHFVNRGVSPYHVVDECRQRLLAAGFIELKETEQWDIKPASKYFVTRNFSSLIAFAVGGRFLPGNGFSMIGAHTDSPCLRVKPRSKRTKQGCLQVGVECYGGGIWNTWFDRDLTIAGRVMVKSGGRLLHRLVHVPRPLLRVPHLAIHLQRDINDSFGPNKENHLVPIIATVVQEELETGSSSSGDASTATTTAEKHHPALVKILCSELKVEPEELMDFELCLTDTQPAALGGVYEEFIYSPRLDNLHSCYCALKALVDSCAGDTLSKDPNVRMITLYDNEEVGSESAQGAQSNLTELILTRLSASASNLTAFQQAAPLSFMISADMAHAIHPNYQEKHEENHRPSFHKGPVIKFNSNQRYATTAVTASVVREVASQVDVPLQDVMVRNDSPCGTTIGPILAARLGVPVVDIGAPQLSMHSIREMCCTSSVLQSTTLFKGFFELF**PAVRSSLVVD** |
| TRINITY_DN34974_c0_g2::TRINITY_DN34974_c0_g2_i1::g.49976::m.49976 | gi\|734631191\|ref\|XP_010743973.1\| | PREDICTED: plasminogen activator inhibitor 1 RNA-binding protein-like isoform X1 [Larimichthys crocea] | 54.9 | M**PGQMQEGFG**CAITNRFDQLFDDESDPFELLKQAEVKKKKEAAAPGAAKTAAQAAKQPKKESQKDRKVPLTDKKEETQAPVPLKKDGPGMRRMGRKPEGGDGPRPQGGQGDGRPPTDRRPADRRPPRRFERPAGDAGDKPEGGEFSVEKPIGDRPMRGRGGPRGGGRGGRGRGMGRSDGFDSRGKREFDRHSGSDRSSLKGEEKRGGSGSHNWGTVKDELNELDQSNNTEETPEGEEHPPADSENKENEVEEVKEEGPKEMTLDEWKAMQDKERAKVEFNIRKANEGADWNKGFVLHKSKAEVKKDDLIDPEVEEPKGDDEHHFRKPANDITSQLEINFGDMARPGRGRGGPRGGRGGRGRGAAGGGGPGAGAGGGEATRPVRTGGRPDKSSASVPNVDDPEAFPALA |
| TRINITY_DN20883_c0_g1::TRINITY_DN20883_c0_g1_i1::g.28839::m.28839 | gi\|542220597\|ref\|XP_003440913.2\| | PREDICTED: ATP synthase-coupling factor 6, mitochondrial [Oreochromis niloticus] | 77.7 | FYRPRTSRLHVPTIHRGSSTFGILFCLSVKPKMALHRLFKLSSVLRSAVSLTLRRNIGISAVVFNRAKELDPVQKLFLDKIRDYNTKSKSSGGIVDAGPAYQKNVSEEVTKLQRLYGGGDFNKFPDFK**FTEPKFEEGTK** |
| TRINITY_DN40464_c0_g6::TRINITY_DN40464_c0_g6_i1::g.97446::m.97446 | gi\|734616970\|ref\|XP_010736155.1\| | PREDICTED: 60S ribosomal protein L28 isoform X1 [Larimichthys crocea] | 17.5 | MSSHLQWMVIRNCSSFLIK**RNGQTYSTEPNNLK**SRNSFRFNGLVHKKTVGVQPAADGKGVVVVMKKRAGQHKPAGSYEKITINKNSRATLNSLRHIISKNKYRKDLRMAALRRASAILKSQKPVVVKKKRTRAAKTA |
| TRINITY_DN104026_c0_g1::TRINITY_DN104026_c0_g1_i1::g.132918::m.132918 | NA | NA | 69.9 | CVCACACVYVCVDTSRSPWSQSLSLSLI**SSFLPPSSSMSLLPR**TARSPRVRFPVVGPAVPPLPDLLEMFPLLVGRADSRAQRRDVLPWGGKLLVLVGGPVLSRPHLLTDLVDG |
| RRRRRTRINITY_DN59229_c15_g167::TRINITY_DN59229_c15_g167_i1::g.98304::m.98304 | NA | NA | 80.2 | SCSQDLDGSTLGDTREGGRAEVRHH**EPEPEPEPEPEPEPEPEPEPEAAH**HDTGHQERPQPEEAAAARRGPQSEAHQPAAHPAARPAAAAAARLLVRGGHARAAGRH |
| TRINITY_DN57762_c0_g1::TRINITY_DN57762_c0_g1_i1::g.113136::m.113136 | NA | NA | 49.2 | SSSQSAPIAIWSSPVCIALDSISLHLILIESEVPGARGKVLINELAVTSVGSLLLLELLLELLSS**FASGLPSEGET**TGVVCSFLFSSEGLSPPPGSSKSFFHDLIKDFARDVTSSSVLCLRMALTDIPILVD |
| TRINITY_DN55400_c0_g5::TRINITY_DN55400_c0_g5_i2::g.41863::m.41863 | gi\|432912307\|ref\|XP_004078866.1\| | PREDICTED: muscle-related coiled-coil protein-like [Oryzias latipes] | 54.4 | GVSPSPFSPSSDLSLGVSGCWVLPLSLLSPCWLPPTALVPLSPKLKHPFFKQLFLKFKGFETFETEKNSAMDQHKYRTAGVQEKLEIVGVEDESGNPISALTILSLLERVAGIIDNVQSCQQRMEERQLELESNIKTIQGDVLKLAKDHTDTSGTVEKLLQKTRKVSANVKEVRTRVEKQNVRVKKVESTQDELLTRNKFRVVIYQGETEIPSVAVTKSPKGTGLDGLELEPDAYDLPADLSSDEEYLSVEETDSSRAARLKKSVAKSTEHLKAAFSKENMSKRKDNLGTKFHHLGEKVMPAERREKMHQAGERLKQSGERLKENIAKKAPSKEAFRIKLKKERAVAEGQEGAEGETDAKESPVPPSAAGVAYTEVGPETKR**EGPVEESSATRIG** |
| TRINITY_DN86998_c0_g1::TRINITY_DN86998_c0_g1_i1::g.26747::m.26747 | gi\|734626328\|ref\|XP_010741301.1\| | PREDICTED: M-phase phosphoprotein 8 isoform X1 [Larimichthys crocea] | 16.8 | RTPTDDRRKRREDSEPKLFMACDDNQDNQEPPEGADKSDRGQATLSLGMDLNLDWMSLDDFQKHLNGEDEILSGPPLSPSELRDAVKSGDYMAVKLALNSKEDYNLDQEDVSGMSLSMLAAAGGQDDILRLLIKKGVRVNGRQKNGTTALMHAAEKNFLTTVAILLEAGSYLNAQTLSGETALMKACKRGNADVVRLLLEYGADCNILSKHKHTAMYFAKLSNNLVVCDLIRDHISVLSSVAEETIRAYFESRLVLLEPVFPLACHRLCEGPDFSMEFAFKSQPQPEGSGILLFIFHANFLNEITARLCGPCSVHAVVLNDKFQ**LPIFLDSH**FIYSFSPIPGINRLFIRLSEAPTAKVKLLICAYRVQLQ |
| TRINITY_DN104049_c0_g1::TRINITY_DN104049_c0_g1_i1::g.132930::m.132930 | gi\|734631009\|ref\|XP_010743873.1\| | PREDICTED: filamin-B, partial [Larimichthys crocea] | 41.7 | EPCLLKRMANNHIGISFIPREVGEHRVSILKNGRHVANS**PITIMVVQ**SEIGDAGRVKVHGDGLIQGTTFTNSSFVVDTREAGYGGLALAIEGPSKVDIQTEDMDDGTCGVTYCPTEPGNYIVSIRFAEEHVPGSPFTVRVTGEGRIRESITRRQKAASVASVGSVCDLSLKIPAIDMKDVTAEVTSP |
| RRRRRTRINITY_DN40270_c0_g1::TRINITY_DN40270_c0_g1_i1::g.8496::m.8496 | NA | NA | 14.1 | HSLMESLTGGGGKQRFDEPWTSS**SLAAGSASFFTSGS**FSFRRFSYVMWCCEPMGWFLVYALKSRKAWTTSSMLRISVPSDSTAPDWWARWPSRSASSEM |
| TRINITY_DN51648_c0_g1::TRINITY_DN51648_c0_g1_i2::g.38095::m.38095 | gi\|348519606\|ref\|XP_003447321.1\| | PREDICTED: nudC domain-containing protein 1 [Oreochromis niloticus] | 11.6 | VSHVQDGVHSLDVLLLRVQTDPQEEEEEEAKGSGFSVALEWITVGNAAAHGEERKYEVRKRRLLRGKSVPHYAALEPRGRGLVVASEKPFVFTHVDGQKVEQQEAQPMEVEKTDPVYFWQQTPDDITVSVRLPEGVTKDDVRFRLTADSVSVAVRDFPPLLQGQMFAAVDPEASAWTFTDD**NKSLELTLQ**KRSAGAVWSELVLGDRRGEHVMSEEQAALIHQRLNHLTADDLGGPDGDKPPCNSQELEDCDGFPEDSSSLTRFDGETLRPTQVVNLSSHQYLFTVQVNPSEMPCLCLRHDVDALVWQPRPQEPGAMWEHVATFNALGYVQASKRDRKFATCAPNFSYASLCECLRRAFIYRQPSPVETVLFNRKQGRQVGQVAKQQVASLDSDKAILGFRATNERLFVLTSSHLFILKVNND |
| RRRRRTRINITY_DN5942_c0_g1::TRINITY_DN5942_c0_g1_i1::g.71498::m.71498 | NA | NA | 15.6 | QEPQSTFYDDTRFVSPVERRQHPAPRRPSAPKNLFGPVSMTRLIKKFAGPLTGLEGE**AQTVQEEMASVNAGV**MKVFAELKDIRRYIQRMEDSKQQIQPLNENVIQSTDNRIMDLMGVFEDVRTLMEELSKELCLIEEGATARIYSSYSLATHRLDEDDQEPDHQDTGPQRDAEEPSPPDV |
| TRINITY_DN56407_c1_g6::TRINITY_DN56407_c1_g6_i1::g.87323::m.87323 | gi\|657545575\|ref\|XP_008278096.1\| | PREDICTED: phosphatidylethanolamine-binding protein 1 [Stegastes partitus] | 30.5 | MPVDLSQWSGSLELQEVEEQPTQPLTVKYDSVEIDELGKVLTPTQVQNRPTCVEWAGCDPSKLYTLALTDPDAPSRKDPKFREWHHFLVVNMKGNDVSSGCVMSDYVGSGPPKGTGLHRYVWLVYEQPGSLSCSEAVLTNRSGDGRGKFKLQSFRKKYNLGAPVAGTCYQAEWDDYVPRL**YEQLAGK** |
| TRINITY_DN57096_c0_g2::TRINITY_DN57096_c0_g2_i1::g.125565::m.125565 | gi\|657586840\|ref\|XP_008297684.1\| | PREDICTED: glutamate dehydrogenase, mitochondrial [Stegastes partitus] | 50.4 | MYRYFGELLSRSAGSALASGCVDSALPASSSLMRVRHY**ADAADKPDDPNF**FRMVEGFFDRGATIVEDKLVEDLRTRESPEQKRNRVRGILRIIKPCNHVLSVSFPIKRDNGEWEVVEGYRAQHSQHRTPCKGGIRYSTDVSVDEVKALASLMTYKCAVVDVPFGGAKAGVKINPRNYTDNELEKITRRFTIELAKKGFIGPGIDVPAPDMSTGEREMSWIADTYANTIAHTDINAHACVTGKPISQGGIHGRISATGRGVFHGIENFINEASYMSMLGLTPGFQDKTFIVQGFGNVGLHSMRYLHRFGAKCVGVGEIDGSIYNPEGIDPKQLEDYKLQHGTIVGFPGAKPYEGSILEANCHILIPAAGEKQLTRLNAPKIKAKIIAEGANGPTTPDADKVFLENNVMVIPDMYLNAGGVTVSYFEWLKNLNHVSYGRLTFKYERDSNYHLLMSVQESLERKFGKQGGPIPIVPTADFQARVAGASEKDIVHSGLAYTMERSARQIMRTASKYNLGLDLRTAAYVNAIEKVFKVYNEAGLTFT |
| TRINITY_DN52217_c0_g1::TRINITY_DN52217_c0_g1_i1::g.68870::m.68870 | NA | NA | 36.5 | MGSGLTHYLEGLFGPFSQRFDVSSRFPPRQDVSLRVSSQNISGQTEHKALDELGLLVFSKDALFLSELSRDQCPEVD**VGFPACDDVGAV**AWVKLHRKHGLVGALDLCVLGGLVAVPHRQHVFVGVVDHTQERPGVRNREGHGSHSSLEGAEADLLQRDQGGGVPDVNTRLQGLPVFVHTRCLTRRHSHFVRMEGETANAVCVFAVVTLCSVVDVVEHDDAGDEVHRLARRQEVQVGPAVAPPVTVDPVQLQTLRGRLHLQVVVVLDVGGDVHGDGPAADEHLDRLSLAVGSSPGPRRPCGGDQLLGSVLQARGAREEVGRGRRLLIQTLPLPIR |
| TRINITY_DN44215_c0_g1::TRINITY_DN44215_c0_g1_i1::g.79637::m.79637 | gi\|831579180\|ref\|XP_012737738.1\| | PREDICTED: hematological and neurological expressed 1-like protein [Fundulus heteroclitus] | 40.8 | QQQQQQQQQQELAGRPVGKLTKLSSGNLKAQKQKTTMTSTNMFQGLETSSKPSSRVLRPPGGGSSNLFGGYEDDAAATRRPNKMASKVFSPAEEPQSVPRRSNPPGGKSSGIFGECEPPAQPQRPIPPGGSTSNIFGSAESAPVQSPLRSHPNKPKDNLSVGPESKPEPPAPKVKVSQPEVKEVAAPPAPAP**APAPAAAPAPAPA**AAPAPAPVPAKEEPAAVSAPPEPEATPPPSSSSSSSPPPDDMKKHEPHLGPKPRSHN |
| TRINITY_DN58685_c1_g7::TRINITY_DN58685_c1_g7_i2::g.63594::m.63594 | NA | NA | 49.7 | WASCSSRSMRTTSTRCFMTKRPFRWKTGMSQRYRANHTSLPGRPMSTCCSTNLCLIGSRA**SFASSHRGQGSL**VNRVRVGGALVEKGRSSRRGQVLRKDICFVSVPVPWVQRLPERSREVRSSRSLEQRLRVSCSFLLLLLFLLLLLLLL |
| TRINITY_DN40476_c0_g1::TRINITY_DN40476_c0_g1_i1::g.97533::m.97533 | gi\|734611971\|ref\|XP_010733416.1\| | PREDICTED: 60S ribosomal protein L4-B isoform X1 [Larimichthys crocea] | 31.2 | PTSFLLWPPYRQERGSRRAEKLFTKMACARPLISVYSEKGESSGKNVVMPAVFK**APIRPDVVN**FVHTNMRKNSRQPYAVSELAGHQTSAESWGTGRAVARIPRVRGGGTHRSGQGAFGNMCRGGRMFAPTKTWRRWHRRINTTQKRYAICSALAASAIPALVMSKGHRIEEIPEVPLVVDDKVEGYKKTKEAVLLLKKLKAWNDIKKVYASQRMRAGKGKMRNRRRIQRRGPCIIYNQDAGVTKAFRNIPGITLQNVNKLNLLRLAPGGHVGRFCIWTESAFRKLDELYGTWRKSASLKVDYNLPMHKMTNTDLSRILKSEEIQKSLRAPNKKINRRVLKKNPLKNLRIMLKLNPYAKTARRHAILQHDPSIKAKMLKPKKKPGKKGAPAKPKA |
| RRRRRTRINITY_DN44176_c0_g1::TRINITY_DN44176_c0_g1_i2::g.79581::m.79581 | NA | NA | 14.7 | RERLPLNPNSLFSLQQELSCVRFALYCSSLILLIILLTFFKLLQYEMQGLEAVPESLLGRDTQLAREELLQESKFPTDTASLSSTSNGLLDPLSSSVSVVSPKRRWKLVSPFE**DVVPFEDPTP**FEDLSLTPEEMFQKLSLSKGNVQLHTCIRRLVDYVSDRSLLSVFVYKQGTDTTIALGNPVLGATKHKKVLRVSAVPIAVKIDKGFLNACFCLWNRSIYLRGQLLIDRLLACSYVKMLIEDKPVCQFLKHYQSNYKSVTSGRYCKKVDEYSLDEILQFRFHGHTDDDKSPDLDQTSLLGTEESQEQQETM |
| TRINITY_DN48778_c0_g2::TRINITY_DN48778_c0_g2_i2::g.83449::m.83449 | gi\|348527198\|ref\|XP_003451106.1\| | PREDICTED: 28S ribosomal protein S36, mitochondrial [Oreochromis niloticus] | 26.6 | MGSKVSSKMAAPAARVIQAVRPHMPLIKFPNRHDVPKPNAQEALKTLAVNLPQHGTSPLAAAPPPISRTLTPISGTPDTLASIQLLPARYRRRAVAVDEME**FIQRGGPE** |
| RRRRRTRINITY_DN54808_c0_g1::TRINITY_DN54808_c0_g1_i1::g.84158::m.84158 | NA | NA | 91 | KKEADAVAPQKVELKCSVTVEGHDNKARCSYVGGDFNGPKRIELSCIGQVCIQRFKPDDGIIMQNKMWIVKPKPFGRVSCLLKTSYGVTTSRDTLSTTFKPAETFDHEKYELPKYEIGTKLIKAEEKSVAASESIGCKNESFVRFKYTNGMILDSITANLRHYHEVVTFWDGTKKDAKQVTYGTIETNGNDKPATWELAVNFGWTDVIKVSAPPGPLEVIQLTIPAKDEFDEVKVCMEYVGSDDRESTRIFLISDRDTSRINVRKKDLPEGNKTWTVVPKPKGTFPITLNIKDGVYAVYRQKLIRPLRVKPRDAVEKVLVAEPLAIPPSRGGANVAVVRIKLRDGTTQNKIVYRCSKQLDENVAKWDDTGDKCVEITYGDLGSHGITEPQSWIITVSTDNKDEVFLEVPASTPEDPPPPTPAKPEEAPPAAAADEAPAAE**GEAPAPAGEAGEA**PAGEAPAAEAPAAEAPAAEAPAPEPVPEPAPEPAKKEEKKAPEKGAAKKIPAPKSPM |
| TRINITY_DN33878_c0_g1::TRINITY_DN33878_c0_g1_i1::g.66530::m.66530 | gi\|300677970\|gb\|ADK27292.1\| | ubiquitin [Siniperca chuatsi] | 28.9 | MQIFVKTLTGKTITLEVEPSDTIENVKAKIQDKEGIPPDQQRLIFAGKQLEDGRTLSDYNIQKESTLHLVLRLRGG**IIEPSLRQ**LAQKYNCDKMICRKCYARLHPRAVNCRKKKCGHTNNLRPKKKLK |
| RRRRRTRINITY_DN3449_c0_g1::TRINITY_DN3449_c0_g1_i1::g.104468::m.104468 | NA | NA | 7.5 | KDLYVPLFTLVWIELNVASCFRSTQPKYWEVCFFSLDIQVHVNRFMVFFGMQVVTLDVPFSIVRSVQLLANGGTCAFCLMYSASVTLWDQRHSIIIKRK**AFVFFFSLFV**FFLSLISVVKDHFGGSFLGPNRMM |
| TRINITY_DN55094_c3_g4::TRINITY_DN55094_c3_g4_i3::g.60959::m.60959 | gi\|734596849\|ref\|XP_010739068.1\| | PREDICTED: synaptophysin-like protein 1 [Larimichthys crocea] | 19.6 | MDSVAQKVLTSGFTLDLGPLKEPLAFIRLLEWLFSIFAFATTCGYSGTTSFNIICQGQPSREIHAVFNYPFRLMDHPYDVPDCKVNQTTTTPTFLTGDHASSAQFYVCIGVLAFLYSTATLVLYLGYQHVYRESSRGPIVDLLVTGALTFLWLASSSAWAKGLTDVKWATSPATIVSVLPVCHGGTGNTCTPGAVPHMGRLNASVIF**GFLNLILWGG**NCWFIYKETPFHKEAHPPADVEGGVRPS |
| TRINITY_DN50583_c0_g3::TRINITY_DN50583_c0_g3_i1::g.57916::m.57916 | NA | NA | 40.4 | MGVHLRLDVVILTLPGFFVHRLVLCSGGELSNQRVVDGYCVVQTALPVPCGILLPRIKAGIGYLPDVHKPADEIGVCEEHLEHFAGTDVEDLVYVDSTLHLGL**SHLLLHFFN**AV |
| TRINITY_DN7343_c0_g1::TRINITY_DN7343_c0_g1_i1::g.1594::m.1594 | gi\|828407500\|gb\|AKK51793.1\| | peroxiredoxin 1 [Trachinotus ovatus] | 24.9 | MSSGNAKIGQPAPNFKATAVVNGQFKDIQLSDYKGKYVVFFFYPLDFTFVCPTEIVAFSDRAEEFRKINCEVIGCSIDSHFSHLAWINTPRKQGGLGNMNIPLVADLTKTISTDYGVLKADDGIAYRGLFVIDDKGVLRQ**ITINDLPV**GRSVDETLRLVQAFQHTDKYGEVCPAGWKPGSDTIVPDVEKSKEFFSKQ |
| TRINITY_DN36846_c0_g1::TRINITY_DN36846_c0_g1_i3::g.73667::m.73667 | NA | NA | 42.6 | YSGTAILFVTGKPYYFEQPLQSFCSPRQTCYAVQSRKQRVVPLSISLHLGFKRVERLHRRQGRPSQLKCSKINE**STSLPFVLRT**VFSPCVNRNLRSLLLFT |
| TRINITY_DN40727_c0_g1::TRINITY_DN40727_c0_g1_i1::g.117819::m.117819 | gi\|583984960\|ref\|XP_006787734.1\| | PREDICTED: collagen alpha-2(I) chain-like isoform X1 [Neolamprologus brichardi] | 70.9 | GEPGQTGALGPRGPSGPPGKSGEDGNNGRPGKPGDRGTPGPQGARGFPGTPGLPGMKGHRGYTGLDGRKGEPGAAGLKGEPGAHGAAGSPGLAGSRGMPGERGRAGPAGPAGARGADGNVGPAGPAGPLGAAGPPGFPGGPGPKGEIGPIGATGPSGPQGSRGEPGANGAVGPVGPAGNPGTNGLTGSKGAAGTPGVAGAPGFPGPRGGPGPQGPQGSVGPRGLGGDPGAQGVKGDGGPKGEPGNSGPQGAPGPAGEEGKRGPTGEIGASGPAGNRGARGAPGSRGMPGAEGRTGPIGMPGARGGSGSPGPRGPPGDAGRAGEPGAAGLRGLPGSPGSSGPPGKEGPAGAAGQDGRTGPPGPTGPRGQHGNIGFPGPKGPGGEAGKPGDKGATGATGLRGAPGADGNNGATGAMGPAGGPGEKGEQGPSGASGFQGLPGPAGPGGEGGKPGDRGVSGEQGVAGPAGAKGERGNPGAAGASGAQGPIGARGPAGTPGADGGKGEPGVAGNAGGPGPQGPGGMPGERGAAGPPGSKGEKGEGGHRGPDGNAGRDGARGMPGPAGPPGPTGANGDKGEGGSFGPAGPAGPRGASGERGEGGPAGPPGFAGPPGADGQPGARGERGPGGAKGEVGPSGPGGPAGQSGPAGPSGPAGPAGARGDTGPSGLTGFPGAAGRVGAAGPAGIVGPPGPSGNAGKDGPRGLRGDPGPAGPSGEQGMVGPPGLAGEKGPSGESGPPGPAG**SAGLLGPAGIIG**FVGLPGARGDRGSPGGAGVVGEPGRVGPAGPPGARGPPGNLGLAGMTGPQGEAGREGNPGNDGPPGRPGAPGFKGDRGEPGSPGAMGLAGAPGPAGPNGGAGRPGNRGESGPSGPAGPAGAAGARGAAGPAGPRGEKGVAGDKGERGMKGLRGHPGLQ**GMPGPSGPSGDTGAAGSN**GPSGPRGPAGPHGPVGKDGRAGSHGTIGSPGARGPPGYVGPAGPAGSPGLPGPPGPAGGGYDVSGYDEYRADQPALRAKDYEVDATIKSLNSQIENLLTPEGSRKNPARTCRDIKLSHPDWSSGFYWIDPNQGCINDAIKVFCDFTTRETCIYAHPESIARKNWFRSTEGKKHVWFGETINGGTEFTYNDETLSPQSMATQLAFMRLLSNQASQNVTYHCKNSVAYMDGESGNLKKAVVLQGSNDVELRAEGNSRFTFSVLEDGCTRHT |
| RRRRRTRINITY_DN38469_c0_g1::TRINITY_DN38469_c0_g1_i1::g.76300::m.76300 | NA | NA | 14.6 | LGCGQLSMSSLRSPLTHPSSSNEDKLEHSRQQFFDCPRFPFHTESQLTSATATTQSVRLDPGSWEPFGIATHKTSEHVCVCRLFCCFLLLLLLSRSKPVISVLCHECHRSVMWVCVCVRVFVCVCKKIWLSVFGRLCRFSFCFLVCHLLRFVCFKL**DLLSKSKQP**IRLLSLSHTHTKSHTHTHTH |
| RRRRRTRINITY_DN55744_c0_g3::TRINITY_DN55744_c0_g3_i2::g.19917::m.19917 | NA | NA | 15.2 | FYDRRRSDDSIFSPNWQAPYICSHRSGPLGTFPYLGNRELDSNRLAPSYY**IDTMQLLNKT**SGNEQMEITERGWEMSIRNNKPFDAYPSMQLDGSKFIIKNIDNKDKKCCTVILAIILILLLAGGAPSVVVAILKYFNGCNFGGFGFMCPVCTGNELKYGDGCELCSLDDPNHKYYGQLCQCYATGNTDTCTSRETDCQTKQAVCLSEVHYTLQYRLVMRCHSSCNRLFMQISNNVEASTVDTSISFMNIALILFGNEEKKSLTSRSYGQYVSLSANLLQIIERQIEHMIASKSIVSDNPPRKVSFTGLFTKARTCTRGDELDFGLPCECSFSGHTNVCRSGQPCPDRECEDRDQNCNPGTWAQQCLCTYEPGENSVCMGGNMCPNSVCPNGPPPLPTPAMTPQIDTPSTTVVLETTGRETQKGTTHLVETSRTPTSPTYTTTVDHSDAGPTGTTEIQMTTVKTPTAIHTSPGQ |
| RRRRRTRINITY_DN59368_c0_g1::TRINITY_DN59368_c0_g1_i1::g.92804::m.92804 | NA | NA | 23.4 | LLVVL**LETQLHDPGS**VIVAEDGREEERLLRTTRSQSQSSKIHLQCLHLVRNGLLGDTQHPHLEPFRGDLFDVSVFRRVLRDGVLQPPRHPFVVQGLPPQQPDDCLSLLVQLVPGVPVQLLQLRV |
| RRRRRTRINITY_DN51887_c3_g3::TRINITY_DN51887_c3_g3_i5::g.14682::m.14682 | NA | NA | 17.9 | GSGSGSGSGSGSGTSSEAGAHGGSNHEENHIKKHTTPSGKESPCPSPTPNCSESGILQTMYICYDLLTRTKMAGPLDQLEETSEKVADGGPLTEAKNEEGSVSSKAGSASLFHQFWEEKERGTRGFLYLTVPLQHDPLAHIETKEEEEQGEVLSEEGVEGEALTICIPYKKNWVRKRALGPPLLSVKCNSLQYTCSRLFVADRPSEDFAAWRPINARPQALRLRSGELTVYVSHTISPHFTEPDYGHTENMWGELIEPDTLKDDLPCMSSKFRSGKNSRTNSRQPAFMFLVVLGLMFGCAVGVSLGTLYPPLPLVFFGYALLGVPVLWHFPLSSGPVIGRPQAYTKRRTGRSRNQLEVVQLVDGYTQIEYHSDRSHTHHDASGSSKLLSGKQLRRLPTEFVGRHESDTRRFCKFEADEGSMERGCSGQSAPSDGAGSARGQKPSGSSEDGMIKSLMSLPDQMAESFKRRTDELEAILSSGRPETPSMSGTSPPSAWDDNEILGGTEPRAELIRPQTLQKWPHLHLKSDSKSLNVEPEVRRSIETSLSKVLSLLPKSRLSSSERPELDTSSSKVLNTTVLLGA**SSSSLDALSGP**SSPSHPLFPQSRHGQSLDLDIGLSLDPQSERAQSKDKSFAVTPGEDHHDLSFILKSEEM |
| RRRRRTRINITY_DN43189_c0_g2::TRINITY_DN43189_c0_g2_i1::g.71184::m.71184 | NA | NA | 16.4 | VTSENVTGGRQTQHGETDGVDGAYSRERRREDDDANMMMMLMLMARKLDLGTSSLQYCVCVASLVMIAEMVCDDSGSMGSLWLSLQSASSEAASTYQWFDPQSRAMSVAQLFNRN**DPPPPPPPPPPP**P |
| RRRRRTRINITY_DN96081_c0_g1::TRINITY_DN96081_c0_g1_i1::g.110591::m.110591 | NA | NA | 27.3 | YFSHQLLKSNVTAVLQKYFRNKKGCDPLFTVLSGLGSTWNVTPCLDKSL**LVLNLVLSGT**HSSAKVKGMWLGLVWSNSTSTFSALCSHESPVNVTGRLRRSSASSMVSFWC |
| RRRRRTRINITY_DN57181_c0_g5::TRINITY_DN57181_c0_g5_i2::g.21369::m.21369 | NA | NA | 20.6 | VSRTTTLCQNVVELLSSNE**LAAAVREK**DALQKNVAEESAHTGPTIHVDIKFRNPLSRLLKVKISLGILTAMSCHPITPTFEIGVFNEADNVNVRIQEVVNLEELSLPHEPDNISRILDFIERDDIPDHVDEDEDAATMLRDGSRQFIVPNANELRTGGSM |
| RRRRRTRINITY_DN49247_c0_g2::TRINITY_DN49247_c0_g2_i2::g.37590::m.37590 | NA | NA | 12.2 | TSKIGQFGCFNWRKELSAGVGTLEQKFTCPLRSLLAEVESPNIKTDLRQLYSCIYEISAGFPHVHLFTSIIGVLLAERESHVPSPPNKETPGEKEQSCSVVTTETNQNASEENGRD**NQELQMVHT**NQLNQLGEKLTQVQNKLQQKDDLSKELDAERQKLEERMKLLQKQMGQLAQQLFSLQQSHTSDEESRMPQNKGQEQKLLEVENRYADLQCRLSDNEEKLAEAQSVGSDESDKSDPTDEMDDDSMEMEEERRIGMLQENHMNRIEEAQKHWMDLNKRQAKSFHDWAKQKSAAQFVSVIQEFQGLIGSLATKFKEKAEEMEKEHQSKESMLRRIHSNSSQIMSYFNNANRRNVEGRELWTLLVRVAEAFKGDDKIKDGLQSCEHESYHVIPPPSPPRLRDEEMKRRHREERALMRQHCEWEYLDDRAQAFDVHLRGTDKKDTSSGLRIRYGSLFLAKDVTFEEAFRIHCFNKKSKRIATIDGCQGFVEMILQENANEPLGGVFVTKCGPPRDRTAPPPLNPNPPFLTCSKCHIIEKITPIDQSLPAPVIGLGPMMPNMPTIGAMMSLNPDFDPMLPQGTMGMPPMGLNNMATAWANPDSMPMTISNSDMTMNCIGEPPLVTRKRKKSNEAANELPPCESVLPGSSGNFQLPDNNTAPDFPLANLGTYQLLFM |
| TRINITY_DN20986_c0_g2::TRINITY_DN20986_c0_g2_i1::g.83723::m.83723 | gi\|734598400\|ref\|XP_010747287.1\| | PREDICTED: ataxin-10 [Larimichthys crocea] | 33.3 | MAADTNSNLDLLASIADILNENHCPEHLQALKTFTTALRDRQYRDAVEVQAFSRLHEVLTRLSDDLQTADGDDESAALQLQLIAECFRAQRNSCVQSTRNQTLLRELGFVDVSLKI**LSFLLNTN**MESRDAVYEPLRCGIQFLGNLAVGNQLCKDDIWQLSFPNLLLQLLSSDDEKTVNYASMVLHTCLDEAKVEELSEPSKIELALKVMELCRTQPDLDWTVLIATQHFLKSSALVESMYSGMSHHERLTLLELLFAQLREDDSEECGIPSSVARFLANSFQKGCGAVLSLATGSASNDEVLQEALTVISALDVLCEMTSDHRQFMFLQDHPDLLVTTVELL |
| TRINITY_DN46695_c0_g2::TRINITY_DN46695_c0_g2_i1::g.11770::m.11770 | gi\|657583353\|ref\|XP_008295780.1\| | PREDICTED: glutamate--cysteine ligase catalytic subunit [Stegastes partitus] | 45 | DSSSLRVWFCFVFLHLTSLSCAMGLLSQGSPLNWEETKKYADHIRKHGIIQFLNIYNKVKDRQRDVLKWGDEVEYMLVELDDKDEKVRLVLNGGDV**LETLQDQGEKIT**PITPRSGDQSTA |
| TRINITY_DN57931_c14_g2::TRINITY_DN57931_c14_g2_i1::g.43259::m.43259 | gi\|736294681\|ref\|XP_010790936.1\| | PREDICTED: centromere protein V [Notothenia coriiceps] | 45.7 | MDLVKHTGGCHCGAVRFEVLNSPDLHVFHCNCSICTKKQHHHFIVPKMNFTLLQGSENLTTYTFNTHVAKHPFCKTCGVQSFSIPRSNPDGYGVAPHCLDPGTVRSVTVDNFDGEKWEEAMEAHKTIR**DMSRPASDTA** |
| TRINITY_DN79260_c0_g1::TRINITY_DN79260_c0_g1_i1::g.26210::m.26210 | NA | NA | 32.2 | LLLRWDVADVPQTSLVAPQRVLGVFSVSDHHVDRLQLLQVDGGEAVDLLHLPQRDVQLGAQQRGGAGLLRRRQHLLRLLS**DGAQVLLQPLQ**LRLHLHLLGRLHGDGLQRLLVDRLAETSHRAVLTSLQTQTQLLDLRQVALTHRLVLLT |
| RRRRRTRINITY_DN18123_c0_g1::TRINITY_DN18123_c0_g1_i1::g.118198::m.118198 | NA | NA | 15.1 | NAKHHKKKNKVLIKGMLEMPSPLPAGSELPYKELPETLLADGFISRCYEAMKAQQKPSDVHNEFSLIVPFPSTKFACEAIAEIVEKFSIETTITFGHTIVPEEETTRGKWCDL**EICRCGSLLV**QKYMEVSSNGALQGATLYTNHSSNIFYHSLPLTMDESQDLKEPPIISNEEGNLYRAFGEMSIQGKQALSANPEYKDVLLQVQEPKLPPYLIENLRPDRQKNNIFDTMQEVTLYPRSKAGVDSFIHDLEPRSCINNLFMSYVDPTFDEQPISDNRGSPLGCNELATEVRKRDASFMRFINKVPIRGEPNLQLKLRTYGKELCAERSMNQVFLNSALTFLEEAWEKATEEQFAMFNL |
| RRRRRTRINITY_DN52684_c0_g1::TRINITY_DN52684_c0_g1_i1::g.110226::m.110226 | NA | NA | 19.6 | SSDLFAKLLSEAASPCASPSVRLCSLSSVRSRNSTSFSSSTCIPIFCSISSDMFISSFSTQSFCSARTSSLCCSSSRRVMLWCSLW**LDCLTSALE**LCFLSMM |
| TRINITY_DN58269_c3_g135::TRINITY_DN58269_c3_g135_i2::g.44917::m.44917 | gi\|1012730222\|gb\|JAR26581.1\| | Myomesin-2 [Fundulus heteroclitus] | 16.3 | M**ASKDIHWH**MKKHVSHVKTDYAYHHKHAVKHHSERKSSSKSASQVQARATEVMAEPAYTIPVFRQRTAEETEEYQRVSTNVGKGLAVIQEELHRMRIATKAQVDSLEIQREVRGMMSRRGDLLDDIPKMPDFLVALRPHTVWEKTPVKLFCTVQGHPRPIVKWYKGGMPVDPLSAPGKYKIENKYGVHSLIISRCVVSDTAEYSAVATNQHGTASSKAMVTVKRPSGTGESCQLGLVPHLTEIHPSKLEVTLLDRFSVSFGVEGGAISLVATMVVVPDLPNVPPLAQWYRDDHLLKPSKLAEMKVGGGAARLTLPHLAKDDEGLYTLRIFTKDGTAEHSAYLFVSDAAPSAPGAPGAPMSLKAFDVNSDYVMVAWKPPNTVNEAPITGYFVDRREAGSDAWVQCNDAPVKVCKYPVHGLSKGHSYHFRVRAVNSAGMSRPSRKTEKVIAMSADEHERLLGTDEGVPSAPGQVVATRNSKSSVFVQWDPPKHPNHLIGYYIDGRAAGSKDWFPCNHKPFRHTRFVVHGLIPGETYVFRVQAVNIFGLSEESQESSPISVEPALATPSAPFGITMLSCDGSSMTVAWKSPKHCGGSKVNAYYIDKRDADSHLWQEVNTSAVKERICTIEHLTEGTFYEFKVQAGNMAGVG**LASAPSAPMK**CSAWTMDEPGPAYDLSFSEVRGHSLVVLWKAPVYTGASAVTGYFVDMAKKGSKEFVTVNEEAVHHRYLQVKGLEEGHSYVFRVRAVNASGTGKASQVSEPVCAKALPGTKEIEAGVDEETGDIYLSLEACEICETSKFVWSKNYKPIGDCPRVAVTTHGRTSRLTFTNPDKDDLGRYSVVVTDTDGVSGSHTLTEDALNTMLELSHAIRNPIIPLKHGLNYEILEKGHVRFWLQAVKLSSAVSYRFIVNDKEVTSGEGHKISHDVATGIIQMTVDHFTRANEGTYTVQIHDGKAKAQTSLVLVGDVFKAALKEAEFQRKEHIRKQGPHFSEYLYFTVTEDCTVLLTCKVANVKKETTFHWYKEDDEIVPETPPNVMSGACALPLPLFSRKDHGVFKAVLGDDRGKDTSTIDISGQVFDDIINAIAKIAGASASDLVLQCTPEGIRLQCYMSYYTEEMKTVWKHKESKIASSEKMRIGGTAEMAWMQICDPSDKEKGHYSIEISDGVQTHTRTFDLSGQAYTDAYEEYLRLKAAAFAEKNRGRVVGGLPDVVTIMEQKSLSLTCTVWGEPTPEVTWFKNEQEVTSSEHTKVTFEGGKFASLVITKVTPDDSGKYSINVRNKYGGEFVEITVSVYKHGEQIPEPKLGQPRMAATPASTPATTPMPPKSPAPHSKTPTPTPSKSHTPAPSLKSPTPASTPIPKSPTPTPRSPTPKSPPSTPRSVRSPTPPRFMKSPTPPRK |
| TRINITY_DN58644_c1_g1::TRINITY_DN58644_c1_g1_i2::g.64019::m.64019 | gi\|657548070\|ref\|XP_008278992.1\| | PREDICTED: unconventional myosin-XVIIIb isoform X1 [Stegastes partitus] | 12.9 | GGGGGGGRGGAATESELVLQLECSQTEVEFVRRRLKQTEEKLETERETRQQLDSKVSALQAQLEQARRSVTELKRHGRRVTSDLQDARVLTDSLQGRMHELERKQRRFDSELTQALEEADSEREQKDKSFLENTALGAEIYKLRRDLQESRAEAERLQKQKEELCAQIRDLSRPVDLTCESLPDLKKQLRLLETQSSERSEEIRQLTARIQQQQQIHMRFEMEMERMKQMHQKELEDKEEELEDVHKSSQRRLRQLEMQLEQEYEEKQMVIHEKHDLEGLIATLCDQVGHRDFDVEKKLRRDLKRTHALLADAQLLLSTVDSPGLHLPNGSKDQIERLHCQLEESEASRVEAESVQTTLSQELENTQIELENICKQKSLVDEQLSMLQHEKLDLLKRLEEDQEDLNELMKKHKALIAQSSSDIAQIKELQAELEEAKKQRHSLQEELQQSVSRVQFLESSTVGRSIVSKQEARVCDLENKLEFQRGQVKRFEVLVLRLRDSVVRLGEELEQSAAAEARERDNAKYYLQRLQDMKLEMEDLIQREGDSGRRRMELEMQVEELSAVRQALQADL**ETSIRRIVDL**QAALEEVESSDESDSESVQTAVESLTRKKDLDSVSSVGSIGTEDVGEGIRHWLG |
| TRINITY_DN81987_c0_g1::TRINITY_DN81987_c0_g1_i1::g.26352::m.26352 | gi\|958665750\|gb\|JAO66309.1\| | RN213, partial [Poeciliopsis prolifica] | 26 | DLDVFTY**QGVEGSHVE**CLQMLLMYCGITDPSWAELRNFAWFLNLQLQDCESSDFCNDTLTEDTGLTGFKTFVVDFMILMAKDFATPSLSISDQSPGRLQMDLTGVRDEDLAPFKIRKRWETEPHPYIFFNDDHVSMTFIGFHLEPNEQNFVDAIDPTSGRVIKKNVMTRALYEGLRLQRVPFNMDFDRLPRGEKIE |
| TRINITY_DN53568_c0_g5::TRINITY_DN53568_c0_g5_i1::g.39152::m.39152 | gi\|498999453\|ref\|XP_004554488.1\| | PREDICTED: myozenin-2 [Maylandia zebra] | 36.5 | MSRFSTMTTQERKMQAAAICREVQAQEDLEMDLGKKMCVPKDIMLEELSLASNRGSRLFKMRQRRSEKYTFENIQNENNKQLNDAVVSQTENE**NAVDGHGGQNNTGVEQPSD**APETAVVPNPDSIAPGYGGPLKDIPAEKFNCTAVPKSYHSPWEQAIISDPALADTIVTHLPEPEPRADLPGFKSFNRVATPFGGFSKTPRPAPIKPVQVEPLPDFPELQAETTVDRPSFNRAALGWVSTGGPLTLPTVPLEPMFIPESEDL |
| RRRRRTRINITY_DN45575_c0_g1::TRINITY_DN45575_c0_g1_i4::g.34400::m.34400 | NA | NA | 16.1 | STSLDELTGEPDLGSPIQEESTSMWITRSGLVGSALTTWTTRGACSAGLGVRSVLGGLAWFFLEAWVTITFMTSFGDLETNEGGLRLSGKELDSLSKTFWSLDRAMN**LSSELDAPDL**FLSCTPRGPGLFFARIDRVRESKRVR |
| TRINITY_DN123084_c0_g1::TRINITY_DN123084_c0_g1_i1::g.136278::m.136278 | NA | NA | 37.8 | GVCGWRSGVGLCPLDHQGAGSD**GSAGGGVLPPL**ATLLDPAQSLSPLLLKHVSLVLGEVKALALQHLLLLSDLLHHLLLVLLLLAALPLLFLLVESYQFLDDGRVGLGGGGGRSGDGTLRHCCWACFL |
| TRINITY_DN54879_c0_g1::TRINITY_DN54879_c0_g1_i1::g.40787::m.40787 | gi\|657739072\|ref\|XP_008332233.1\| | PREDICTED: probable rRNA-processing protein EBP2 [Cynoglossus semilaevis] | 21.5 | MVIDGMSMESVEEEEQLGLESEEENSELSDNELQEAFAKGLLKPGMNVLVNKAKKFVNNVEGMKHCLADLRRDLPWVERLDLTN**LPAEDIISKAE**GKIPNKTNGDVNADDDFQREMFFYRQAQATVLEALPLLSKYDIATKRPDDYFAEMAKSDQHMQKIRKKLISKQMILEKSEKAKKLREQRKFGKKVQVEVIQKRQKEKKAMMTAVKKYQKGMTDKLDFLEGDQKAGKGSAQASKKEQNKKGPNAKRKFKDQRFGFGGKKSGKKWNTKESYNDVSSFRAKVANAKGGKGGKKGKGGKQNKRPGKSVRRKMKGRS |
| TRINITY_DN116106_c0_g1::TRINITY_DN116106_c0_g1_i1::g.130398::m.130398 | gi\|808876634\|gb\|KKF25251.1\| | Titin [Larimichthys crocea] | 20.7 | DDEGEYTAVVGDDKCAAELIISEAPTDFSVSLKDQTITEFEDAEFSCKLTKEKAEVKWYRNGREIREGPRYTFEKNGKFCTLRIKECRPDDECEYACGVDDKRSRARLFVEEI**PVEIIRPP**QDVFEAPGSDVVFEVELNKDRVEVKWLRNNMTVVQGDKYQMMS |
| RRRRRTRINITY_DN67429_c0_g1::TRINITY_DN67429_c0_g1_i1::g.64638::m.64638 | NA | NA | 9.1 | CFAVSPAFFGHSRALEASL**DITELTRGT**PLPLSVSENSRSLIFCSIKSSTNRSNCLTFSSIMSAMLCSSLRTLVVYDRLAVGRCSGGKRVMAGAAAATA |
| TRINITY_DN58243_c0_g1::TRINITY_DN58243_c0_g1_i1::g.44626::m.44626 | gi\|734610466\|ref\|XP_010732594.1\| | PREDICTED: collagen alpha-2(V) chain [Larimichthys crocea] | 39.3 | MMSFVHLRTFLFLVVSVAQVLIVTCQDGTSGDDMSCTADGQVYTNRDIWKPEPCRICVCDNGQVLCDEIQCDELSNCEKMVIPEGECCPICQTESPSTGGTDTFGGGGRIYKGQKGEPGDVPLVTGIRGRPGPMGPPGSPGDRGQRGNKGRPGLRGPAGYDGEPGVPGQPGEAGPPGHPTHPGGLEAQMASGFDGKTGPQAMLSGSRGESGTRGPPGPNGVPGHAGPQGPPGEVGDPGHMGSSGQRGPEGPPGKPGEDGEAGKSGNSGEVGFPGSAGSRGFPGTPGPPGLKGHRGHGGPLGQKGETGALGSKGATGPPGPMGGPGPMGPAGMPGERGRPGPGGIAGKRGSQGNIGKHGPMGPLGINGPPGYPGTPGMKGQPGPTGVRGPEGPQGQRGETGHLGRAGPVGLRGPGGTDGGPGSKGPVGHLGPQGAGGHPGPSGPPGPQGSTGQPGIKGQLGDVGVPGFKGEAGPKGEPGPPGSQGVIGPQGEEGKRGPRGDSGSVGPHGPVGERGAPGNRGFPGADGLPGPKGAQGDRGTGGPGGPKGSLGDPGRTGEPGLPGARGLTGTPGVQGAEGKPGPSGAPGEDGRPGPAGSIGNRGPAGTMGVLGPKGFSGDPGKTGEQGSAGVPGQRGPPGKDGEVGPAGSQGPSGSAGDRGEQGPPGVNGFQGLPGNQGPPGESGKPGDQGIPGELGAVGQIGPRGERGIPGERGELGANGLQGPKGIPGAPGPDGPKGSPGPPGALGDVGPPGLQGMPGERGISGPPGPKGDRGAIGEKGSEGTPGNDGARGAPGPVGPLGPAGPSGEKGEPGPKGPAGPAGSRGVPGARGEPGPIGAVGFSGPPGPDGQPGVKGEPGEQGQKGDAGLPGPQGLAGAHGPPGPVGVAGLKGGRGTQGAPGPTGFPGSAGRVGPPGPTGPVGEPGPLGPPGKEGPLGLRGDHGSPGRQGERGPAGPPGSPGDKGDSGEDGPTGPD**GPPGPAGTTG**QRGIVGLPGQRGERGMPGLPGPAGPPGKQGTTGSSGDKGPPGPVGTPGANGPRGDPGPDGPAGSDGPPGKDGVLGQRGARGDPGPEGLVGPQGLPGTPGPVGIPGDAGKRGEAGSRGPVGPPGSAGKRGLVGPQGPRGDKGDLGDHGERGQKGHRGFTGLQGLPGPPGTTGEQGTPGIVGPGGPRGPPGPIGPHGKEGYVGQPGPMGPPGTRGLSGEIGPEGPPGEAGPPGPPGPPGPPIAAMDDLFGGGPQDYDSGPPPPPEFSEDEAMPNSNSSTIVPVDPSVQATLKALSSQIDSMKSPDGSRKHPARTCDDLKRCYPMKKSGEFWVDPNQGSAEDAIKVHCNMDTGETCISANPDSIPRKVWWSSSRNKPVWFGADINRGTHFTYGNKDQPANSITVQMTFIRLLSKEASQTITYHCKNSVGYKDERNSNLKKAVVLKGSNDLELKAEGNSRFRYTVVEDSCSRATGNWGKTVFEYRTQKTARLPIVDIAPVDIGGPDQEFGVDIGPVCFL |
| RRRRRTRINITY_DN53035_c0_g1::TRINITY_DN53035_c0_g1_i1::g.16041::m.16041 | NA | NA | 19.7 | IESLAEIPQNSSHLTNVPTGKDEFAVRTGDSSGDCNILVQNCDEAEMLPRTKSHVNLDILEEFSWQNSKGNLGSVLGVKASDQENLFESGEVLHGKPAPSVAQTEETSIGNNNEKDDTEPKGNVADLPEEEQLEPEGSALDDSACEDKDAETTIDDGQANTECSMEGGDEDGNEPSKDDDRKGDPGDGKRTRKMIEEIRKKREMREQQNQQMIRDREQRVREAEELAKAEAEEREKQLEAQRVAEEEAMKVREEEAMRAEEEERVKREEALKKEEEVRKLRAEEELRKEEEKRLREEEERQVRLQEEKEKQERMLRRNEALIKA**AEAASNTGTAGKPD**DKEKTKSSDSSQPATAQVPCLEKSKSDRKRLTPPGTPTLAPKQIPPPKTSAPQPSPPRQKMAGPSPPRTRSGQKATGPSPTRKP |
| TRINITY_DN55183_c0_g5::TRINITY_DN55183_c0_g5_i4::g.18913::m.18913 | NA | NA | 48 | PRRGESVLQEAAAALAWRLEQLDVATQPGHKGPNHHVRHLWQLVVQQGGGGVVRYLLEDLVLGLGPDVQ**LSVLVVNVLVE**SADDGPVDEQRAHHDDGLQHLA |
| TRINITY_DN51608_c0_g1::TRINITY_DN51608_c0_g1_i1::g.58214::m.58214 | NA | NA | 19.1 | MVGTELDVVKTSR**ALICDLIISA**IDMTFDTGFGISVNSIICSDVFMFSGLFIKCGMAASNSFDFKFCNSDLLSTFDIVVFFSLQDASTVIISASLSTTSLVFSILVCSTIPKAIVSVSISTTFSFWASTNNSATLLLSTSSSTCISVPSTVFIAVSTWLCGVVGGETLHPGEFSAPPGLSTGDFCRSVKRTAGL |
| RRRRRTRINITY_DN51160_c0_g1::TRINITY_DN51160_c0_g1_i1::g.70811::m.70811 | NA | NA | 18.7 | GYLDDDAEENFVPGSGTGGSGHSPGSGGTASSPFRFSGFGRSQQLTQAFMEYKRIDNDSVSRRAFRMAEEFHDKRIEPVPDDEEVEMASPNTQRERERRIENEISERIALKCARQCIETLDAGSFGNTMKALFDLDVDKSIPSKRLNAKLISMRSKEDPLPIYILQDLRGPRLIAPDIIDPRNTAGIIFVNKKSSMGDMETLIQNIVRDAAGGGDGVNGGRAKAISDLEDFFLVCPAAQRAKDFIERVNAESEGFWMTLLEPGKISIFNAQCENAIAKALLTKGCGPPGYFLVGKSPTMGFKLFKDPHEVPYQVLEQLERKVDELGGIDEWTINPVEVVTERLASPNSQSLAWKFDDMTVALSNMVEADITEDELDILDMKKRIAQ**LAAESCLAAL**DAGVHGHTENAVQELDVDDALKMNKTHIQLIELRGTADPIGIDVERDFRGFRRLAPDISNPRNTAAMVIVHARQKLGDMLTLLQSVIRREVEGHTKERKPAIADLEDIFIIAPANKEAEEFAKRLNSESEGALKSMIEPGNILFFFAGTENAVARAILTKGTGPPGYLLIGRPPKVGIAKFLAPHRLPLEVMEKIQALQKRVGGIDDYGVENLSEEEDERRIPEGECHIVTDPAVICYPSPDTEVVKFEVARMGGRVLFIDGKRIPRYAELFYPKLYVEFLNGTIGEVTDDIPLVHIRKGYKVDPCPQISIVDGLRVRLNNRVVRNMRVKEDSCTDDSLVICVTERRKKGKMLVTDGRFLQLEDMKTQSLSVVSNDENISEDVILRNPRNKQKLIATALDDNKSEGGSAM |
| TRINITY_DN54347_c0_g5::TRINITY_DN54347_c0_g5_i1::g.17703::m.17703 | gi\|734609199\|ref\|XP_010731893.1\| | PREDICTED: SH3 domain-binding glutamic acid-rich protein isoform X2 [Larimichthys crocea] | 23 | M**VIKVFLAS**SSGSTAIKKKQQDVVGFLEALKVDYTQLDIACNEENRLWMRKNVPEERKPANGIPLPPQIFNEESYCGDYDTFFDAKEDNLVYSFLGLPPPPGSKEAAEADKADIVENGTHGEETNAEGNLDESIEARAQEEGEQEEADDDREEEEAEAQEEEAAE |
| RRRRRTRINITY_DN54000_c1_g1::TRINITY_DN54000_c1_g1_i1::g.79990::m.79990 | NA | NA | 25.7 | DLHAEEPEEDHHEHSCQQAPDVDLLEVSEEESGSAAGEAALAGSDNDATSEDAASAQLLLDVPSPCPPDEAQVPPEAPPDHSDVPLPSDGMPPSPSESSETPFSQPDTSEAPLQHPEAPPFGSDSFIDTSALASALDMTVQSSLEQSENQDEASSSLLVSDELEEVGSIEGEREEHMALSTNTMDISSMSLDLRDAVDAAEPNMVAVESDSFGPDMVRHVPPPLPAEQISVQEVDLVEESQSSPAMLPAETFGHTPLTPSPEEEPEPTPPKPTPVSPNVPIRHLYGYGIGCGLSGEGGWAGNPTVVVERCGDTQINYVLLKLPKGEHAEVLSFFDESDQLVQDAGVIYDTHPQLGALAAP**SSAEVDLVH**WVNESAGQYSCFRVSAGLLGQGGWMTSPVVELERVRTSKTSYVEMRVAKEMNAKLIEKLLENEENLRKSDLSLIFDFFPQLGAKEAPSNPQVGHVHYGYTGGESAESSQSLGM |
| TRINITY_DN56228_c0_g1::TRINITY_DN56228_c0_g1_i1::g.20225::m.20225 | gi\|657570952\|ref\|XP_008289022.1\| | PREDICTED: vacuolar protein sorting-associated protein 41 homolog [Stegastes partitus] | 12.9 | MAEVEEGRKSSEEFTDE**SEEEDSEEE**PKLKYERLSNGVTEILQKDAASCMTVHDKFLALGTHFGKVFLLDIQGNVTQKFEISSVKINQISLDESGEHVGICSEDGKVQVFGLYTREGFHENFDCPIKVVALHPQFTRSNYKQFVTGGNKLLLYERNWLNRWKTSLLHEGEGSITNIKWRANLIAWANNVGVKIYDISTKQRITNVLRDNVSLRPDMYPCSLCWKDNTTLIVGWGTSIKMCVVKERNPTEMRDLPSRYVEIVSAFETEFFISGLAPLADQLVTLFFVKENSDQMDEEFRARPRLDIIQPLPESCEEISSDALTVRNFQDNECRDYRLEHSEGESLFYIISPKDIVVAKERDQDDHIDWLLEKKKYEEALMAAEISFKNIKRHDVQKIGMAYINHLVEKGDYDSAARKCQKVLGKNMELWENEVYRFKTIGQLKAISQYLPRGDLRLRPAIYEMILHEFLRTDYEGFATLIREWPGELYNNMAIVQAVTDHLKRDPTNSTLLTTLAELYTYDQRYDRALEIYLRLRHKDVYQLIHKHNLFSSIEDKIVLLMDFDKEKAVDMLLDNEDKISTDRVVEELADRPELLHVYLHKLFKRDHHKGQKYHERQIGLYAEYDRPNLLPFLRDSTHCPLEKALEICQQRNFVEETVFLLSRMGNCRRALQMIMEELEDVDKAIEFAKEQDDAELWEDLISYSIDKPPFITGLLNNIGTHVDPILLIHRIKEGMEIPNLRDSLVKILQDYNLQILLREGCKKILVADSLSLLQKMHRTQMRGVRVDEENICESCHTTILPSDMAKPFGVVVFHCRHMFHKECLPSPGTIHGVQFCNICSAKRRGPGSGILEMKK |
| TRINITY_DN40649_c2_g1::TRINITY_DN40649_c2_g1_i1::g.8817::m.8817 | gi\|657574681\|ref\|XP_008291057.1\| | PREDICTED: titin [Stegastes partitus] | 19.7 | VASAIQKTTMRVIRLHDGVEYIFRVFAENKYGVGEYLRSDPVIAQHPFNVPEAPAPPEIVSIRHESAILTWADPKDTGGSPITGYHVEFKERNSLMWKRATKTPLRVKECRVTGLVEGLEYEFRVMAMNMAGLGKASRVTEAVVALDPIDPPGKPDVINVTRNTVTLIWTAPKYDGGYKLIGYMVEKLEAGGKAWMKANHVNVQGCAFTVPDLTEGSQYQFRIRAKNSAGAISVPSESTDLLTCKDEYEPPSITIDPDMKDGVSVRAGDTIVVSASKIVGKPPPTSFWSKGGRELKSSDIVTITSTPTSSTLSIKYASRKNTGEYTITASNPFGIKEEHVKVKVLDVPGPPGPIEASNISAEKCTLTWLPPDEDGGCSIKSYILEKRETSRLQWTKLAENVIDCRYVASKLIKGNEYIFRVSAVNQYGTGDSSQSGPVKMVDSYRPPGPPSIPEIDNVTRTSVTISWKRPVQDGGSDIRGYCVERKERRGMRWVRACKRTVPDLRFKVQGLSEGVEYEFRVTAENKAGFGEPSEPSSPVMTKDIVYPPGPPSNPRITDTTKTTASFAWGRPFYDGGLPVEGYIVEYKKDGHDDWETETQYPLKVTEHVIGKLQKRGKYHFRVSAVNSEGVGEPAEVEKVTELVDQEALPDFELDAELRRTLVVRCRASIRMFVPIRGRPVPEVTWSKDDTNLKQRAHIDTTESYTLLVIPDCTRYDAGKYNLCLENVAGKKTGFVNVKVLDTPGPPVHLKPREITKNSITLQWEIPIIDGGSKIHNYVIEKRDATKKAYTVLTTTWQKCSFKFTDLEEGAYYYFRVSAENDLGVGEPAETPEPIRVSQAPSAPENLYVTDVTADSASLAWVKPLHDGGSLITGYVIEAQKKDTDQWVHVGTIKALDYTVTDLIEGAEYTFRIMAVNASGRSDPRESRPAVIREQTSAPSFDLRGVYQKTVIAKAGDRVKVEIPVLGKPRPVVSWKKGDVSLKETQRINTETTPTSTILNIGEIKRTDGGQYSMTGKNMLGTVTETITVLVHDIPGPPTGPIKLDEVSCDYVLMSWEAPENDGGVPINNYIVEMRETTGTSWLELAATVIRTTFKAARLNTGTQYQFRVRAQNRYGIGPCIVSESVVAAYPFDVPGQPGTPVVTSFNKDAMTLSWNEPSSDGGSAILGYHVDRKEKNSILWQRISKALVVGNIFKSTGLVDGIAYEHRVTAENMAGLSKPSKPSETMYALD**PVDPPGRP**VALNITRHEVTVSWTKPEGDGGFSITGYTVERREMPNGRWLKANFNNILETIYTVSGLIEDATYEFRVFARNSAGAVSAPSQSSEAITCRDDIEEPRLDVDASYSSNVVVMAGEVFKLEANVTGRPIPSLVWTKEGKELEDTGKLEIKTSDFHTTLVNKDSLRRDGGAFTLTASNPGGFAKFTFNVKVLDRPGPPDSLTVTDVTAEKCVLNWLHPTHDGGAKIEYFIIQRRETSRLAWTNVATDLQANRFKVTKLLKGNEYIFRVMAVNKYGIGEPLESEPVVCANPYVPSDAPQQPEVTTITKDSMVVCWERPEHDGGSRINTYVIERRDKTGLRWVKCNKRTVTDLRFKASGLTPGHEYEFRVLAENNAGLSAPSPSSPFYKAVDTIFQPGPPGNPRVLDTTKSSITLAWNKPVYDGGSEITGYVVETCLPEEDEWTIQTPKKGWTATSFTITNLKENQEYKINICATNCEGVGEPAAVPGTPKAEDRLLPPEIELGAELRKVVCIRACGTLRLFVPIKGRPAPEVKWSREHGESLDKAIIEITSSFTTLQIENVDRFDGGKYMVTVENASGSRTAFVNVRVLDTPGAPQNLIVKEVTRDSVSLVWDAPLIDGGSRVRNYIVEKRESARKAYSTVCASCHKSSWKIGELEEGKM |
| TRINITY_DN51668_c0_g2::TRINITY_DN51668_c0_g2_i1::g.38123::m.38123 | gi\|657559650\|ref\|XP_008283695.1\| | PREDICTED: methylmalonate-semialdehyde dehydrogenase [acylating], mitochondrial-like [Stegastes partitus] | 14.2 | LINSNPYGNGTAIFTTNGATARKYTHEVDVGQIGVNVPIPVPLPMFSFTGSRGSFRGDTNFYGKQGIQFYTQIKTVTSQWKAEDATVTSPAVTMPTMGR |
| TRINITY_DN52590_c0_g1::TRINITY_DN52590_c0_g1_i1::g.58894::m.58894 | gi\|734640681\|ref\|XP_010749192.1\| | PREDICTED: dol-P-Man:Man(5)GlcNAc(2)-PP-Dol alpha-1,3-mannosyltransferase [Larimichthys crocea] | 13 | RSPRRLYAAEMAGGVRRKSPGSPSPLWGKLHSLWQDKHLVLFKTEYTLLVVSVLWFLEIGINVWVIQKVAYTEIDWKAYMDEVEGVINGTYDYTQLKGDTGPLVYPAGFVY**IFTALYYIT**GHGVNIRLGQYIFAVFYLITLLLVFRIYHRTKKVPPYVFFFVCCASYRIHSIFVLRLFNDPVAMMLLFAAVNLFMDGHWTLGCGLYSLAVSVKMNVLLFAPGLLFLLLSEFGFIRTIPKLSLCAVIQLLLGLPFLLDNPIGYLSRAFDLGRQFMFKWTVNWRFLPEWLFLNRYFHLVLLAAHLLTLLLFAFRRWKRPGESIFELLKDPGKRKTPPQKSTVDQIVLILFTSNFIGMCFSRSLHYQFYVWYFHTLPYLLWSGGVKKLAHLLRVLILGLIELSWNTYPSTNSSSTALHVCHLIILLCLWLAPPLPSAPAETQHTPVKDKRQ |
| RRRRRTRINITY_DN55561_c0_g2::TRINITY_DN55561_c0_g2_i1::g.85617::m.85617 | NA | NA | 35.1 | IRGGAFSRRGTLQSRLQHGLNVLSQPFKWHSMTLGTEGATFAYQSSCSTGLYFEENAAAATEEEETDAHDDDKLDGLTVIDSHDSSSLLSVHGKGEAGDGAESVCLTEDVTREGLTDDLSDGAREEVCGASASARTDQLEAEEVPPR**ELLEAGYEVA**AELGLTEIDSGENSIITWGCSSEADSSTESM |
| TRINITY_DN77549_c0_g1::TRINITY_DN77549_c0_g1_i1::g.94567::m.94567 | gi\|1012643614\|gb\|JAQ86588.1\| | RT pepA17: Reverse transcriptase (RTs) in retrotransposons domain-containing protein [Fundulus heteroclitus] | 20 | CAEDNKSSFDAAVVDTVLCNFYVDDCLRSVASEQEAVKLYQDLKAICLTGGFRLTKWMTNDRDVLSSIPPEDRATEVKNLDFDQELLAIERALGVQWCIRSDQFKFHVNIQQRPLTRRGILSMMSSV**YDPLGMLSPV**ILPAKNILQELCRLRTGWDDAVPDHLAQQWSRWMEELQQLTDVGVDRCFKPPD |
| TRINITY_DN49024_c0_g2::TRINITY_DN49024_c0_g2_i2::g.118898::m.118898 | NA | NA | 9 | MDNVLMAHEGCHVDGCQARLSDSLDRCSMFKQQLHHFDSVLLAGDVKWSE**AIQGLGVGVGL**LVNDELGHLVVTTVGGDVQRRQVVVGDVVHGHVVLEQKLDAVQVVPLSGHVERRQAVLGLG |
| TRINITY_DN53285_c0_g1::TRINITY_DN53285_c0_g1_i1::g.16306::m.16306 | gi\|657563859\|ref\|XP_008285221.1\| | PREDICTED: myeloid leukemia factor 1 isoform X1 [Stegastes partitus] | 28.2 | MFNSNIRDSDDDAFFSDPFRAHREHMRQMMRSFSEPFGGSIMPSSIMDGRSRGGHNMLEHPNSSVGLRDEHRDMSR**SLLPFGST**DSTDMMRNPFGMFDNIMANMRNRMQDMHQNFETMPSNSNTHSFSSSSVMTYSKVGNEPPKVFQATSSTRRAPGGITETRRAVKDSESGMEKMAIGHHIQDRGHVVEKKFNKKTGEKEFIQDFQNLDESEAQSFDDEWQEEVSKFQPSGPMSRLEEAPPSRTVRWAALTGPEQAQRDQSKGTAEGEGNIKGSGSTKQ |
| TRINITY_DN49335_c1_g4::TRINITY_DN49335_c1_g4_i1::g.75727::m.75727 | gi\|734603445\|ref\|XP_010728732.1\| | PREDICTED: LOW QUALITY PROTEIN: myosin heavy chain, fast skeletal muscle-like [Larimichthys crocea] | 61.7 | FKNKLH**DQHLGKTKAFE**KPKPAKGKAEAHFS**LVHYAGTVDYN**ITGWL**DKNKDPLNDSVVQLYQ**KSSNKLLALLYAAHAGADEAAGGGKKKKKGGSFQTVSALFR**ENLGKLMTN**LRSTHPHFVRCL**IPNETKTPGLM**ENHLVIHQLRCNGVLEGIRICRKGFPSRILYGDFKQRYKVLNA**SVIPEGQFIDN**KKASEKLLGSIDVDHTQYKFGHTKVFFKAGLLGTLEEMRDEKLAELVTMTQALARGYLMRKEFVKMMERRESIFTVQYNIRSFMNVKNWPWMNLYFKIKPLLKSAETEKELMNMKDNY |
| TRINITY_DN49996_c0_g2::TRINITY_DN49996_c0_g2_i1::g.117709::m.117709 | gi\|657588925\|ref\|XP_008298825.1\| | PREDICTED: CDK5 regulatory subunit-associated protein 2 isoform X3 [Stegastes partitus] | 18.6 | NNLMELLDMAEKALMSSDGQDENPELSDVCSRIKDTLQQVNALSNSESPSGVFGRRDSAAMQELQRHTDSLQEALWEQNRLNAELQEKLRDSGKAADAPAQQGLTGVSAGQDGECSTQRGAEKESDDHHGATGSSDDTGSNQEMTKALLNCLSATESAIASLAEHCTNPSSSASTRCSEISTNLQTHLDKLQRALQERKELEEATQPATKSSSSESTAAAAGTKGNQNLHLHQNLSLLYKVFNDLSHRISEMQASLQEERGRKEESEAHRAVQDGKGLPRSVQVQLEALHKALREKKKACKNLEEKLATALTETPSPETARRALEQDDKGVQVDLQDLGYETSGK**SENDREES**SSTDLEVGVNPSCSASSLPSLLKHEQATFSSTENLDSTSSTPYPSS |
| TRINITY_DN9426_c0_g1::TRINITY_DN9426_c0_g1_i1::g.1683::m.1683 | gi\|958809503\|gb\|JAO83317.1\| | SMO, partial [Poeciliopsis prolifica] | 26 | KNYRYRAGFVL**APIGVVLVVGG**YFLIRGVMTLFSIKSNHPGLLSEKAASKINETMLRLGIFGFLAFGFVLITFGCHFYDFFNQAEWERSFREYVLCEANVTIASQTNKPIPECTIKNRPSLMV |
| RRRRRTRINITY_DN22864_c0_g3::TRINITY_DN22864_c0_g3_i1::g.80412::m.80412 | NA | NA | 35.5 | FAGIETGWPRYPKSL**TSSPSVASLATP**SVLPFASFSLPFASLSSLSSLLPPSLKSTQVPLNLPLSGNPSVRLSETPPMVNLRSTGSAESTSSSSTPSSSSSSSSSSPPSS |
| RRRRRTRINITY_DN128253_c0_g1::TRINITY_DN128253_c0_g1_i1::g.138880::m.138880 | NA | NA | 14.8 | EFWLTLNTTHRIMRLLNPSNKEKDEKTWVSGVLESPRVARY**LESELLTL**QRAIEIPHLTMLEFSDVQGARCIHWEIPPPPSEFTINHSVGNSQTQLKRKIIKNISEVWKRMSKGRLQIKSTIYEELRSRLELDNEFDYFHHEVWQRFVNLVRLQVPQVYEKRFRQLEAAMPQDGNWLAQRDEETPEPEPIEIRDILLTLLEQPKCFSRYTTLFTRVFNPDAYMHYTLREILKGVTGAKIIPIGTKSQVKDEFVINEESDQVAFR |
| RRRRRTRINITY_DN9842_c0_g1::TRINITY_DN9842_c0_g1_i1::g.66836::m.66836 | NA | NA | 4 | ENEGEEPGELEAAHQREEEEEEEEEDTLGERKLADMDDSGDDSEATCIGYCMLSIVGLPLGFLFCGLLPSSRFVSMITSKADFAVRKIRQIIGDGGYAQASGNLVSSIFQVLQEMTEIPEGPLFYQENSTNLIIVSPVTVEGMILSNIYDNGDMHGFQFDRNFHERYEKAVRQILSKLRGSKETPDKEDIVAIAVLKGTEGLEYLTFGDIQLYGQFREKNVWSSLSGDEYEDYTFFGGDKFVVVAPLEPLTVSEPLVEESASFFYTYVILESAVDNYKEKLPSEGGIYLFLVDHRKMMHEFMHKSPLARVAPGSVRNAFEVIDDKTRPGKYNYALDGKLLKITPYGRVGFESAMGSYATADMKGVRVPSGSSKLEMGVDMWVPELKKCYGCWPAYFDVLWVDNVRTDKFTDDLDEVFALVASS**LFAAVVASI**LSAKVEAMAELQFVLCSI |
| RRRRRTRINITY_DN31503_c0_g1::TRINITY_DN31503_c0_g1_i1::g.107836::m.107836 | NA | NA | 4.1 | SCQLIIPTEVRESRGVH**WGRKCIRT**LYDTFDPRAIHLLFHPLDPLNNLAVIVNGHVCLEGANNDSRNALEAARHVVLVLQQPTIDVEQLDDLEPEREHVLVSVIGGARRRPLEVEFEVECFVHVRLVGYVQELVGAELKFLVFFFSDVRQVESLQEHRGTQNQTFVDLPERKVAAVVKTVQHLEQCVSSERLLFVV |
| TRINITY_DN58875_c0_g2::TRINITY_DN58875_c0_g2_i1::g.23877::m.23877 | gi\|657577209\|ref\|XP_008292423.1\| | PREDICTED: ATP-binding cassette sub-family A member 1-like isoform X2 [Stegastes partitus] | 13.3 | RDLCNATPLEEFVHIADRNVSRMTQEIICKSSSDWLDQAQSHFLSNLDFLKPIRRDVRSDPKVVQDVSAATDSLLESLGALAVELSSMNSWKDMRKEILYLTANATSSPNQMYQAVSRIVCGHPEGGGLKIKSLNWYEDNNYKALFGNHGNDSESEPLSAYDNSSTPYCNNMMRSLESSPISRMIWRALKPLLMGKILYTPDTPATQRIIHEVNKTFQELGVLRDLGGMWEEMRPKIWSFMENSEEMDMVRTLLQNNASAAFLNSQLSETEWHVSDVFDFLSKASEDHRPAGSTYTWREVFNETDQAIQTISRFMECVNLDKLEPVANEERLVNKSMGLLDNQKFWAGIVFPDIAHSNSTDLPPNVNYKIRMDIDNVERTNKIKDGYWDPGPRADPFEDLRYIWGGFSYLQDVIEHGIIRAITGSKEKTGIYIQQMPYPCYVDDIFLRVMSRSMPLFMTLAWMYSVAIIIKGVVYEKEARLKETMRIMGLNNGTLWLSWFISSLIPLLISAGLLVMLLKMGNLLPYSDPGVVFLFLGSFGVVTIMQCFLISTLFSRANLAAACGGIIYFTLYLPYVLCVAWQDYVGFGAKVIVSLLSPVAFGFGCEYFALFEEQGVGIQWSNLLASPLEEDSYNLTTSICLMLFDAVLYGIMTWYIEAVFPGQYGIPRPWYFPFTRTYWCGEKENKNLSTPLSKKGNAEAVCIEEEPGHIDPGVYIENLVKVYSHGNKLAVDGLSLRFYEGQITSFLGHNGAGKTTTMSILTGLFPPTSGTAYILGKDIRNELSTIRQNLGVCPQHNVLFSMLTVEEHIWFYARLKGLPEEKVKAEMEQIVNDVGLPHKRKSRTSTLSGGMQRKLSVALAFVGGSKVVILDEPTAGVDPYARRGIWDLLLKYRQGRTIILSTHHMDEADILGDRIAIISHGKLCCVGSSLFLKTQLGTGYYLTLVKRDYDLTLQSCRNSASTVCYSKKTEKEDSVSESSSDAGLGSEPESETTTIDVSLISNVIFKHVPEARLVEDLGHEITYVLPYQSAKDGAFVELFHELDDRLTDLAISSYGISDTTLEEIFLKVAEDSGVDAVELSDGVVPTRTRRRHAFGDHQSCLKPFTEDDFDFNDSEESRETDWLSGTDGKGSYQVKGWSLKRQQFVALLWKRFLYARRSRKGFFAQIVLPAVFVCIALVFSLIVPPFGKYPSLALDPSMYGEQFTFISNDMPEDPHTNKLLGALTEKPGFGTRCMEGEPIPDTPCSAVQDEWSVPEVSQSVKDLFDEGNWTMEDPSPLCECSCGGRKRMLPECPAGAGGLPPPQTKISDTDTLQNLTGRNISDYLVKTYAQIIGKSLKNKIWVNEFRYGGFSLGARNSHMLSNRDEIDDAIAELRRRFRLERGTAADRFFGSLSSFIQGLDTKNNVKIWFNNKGWHSIGSFLNVMNNGILRASLPTGKDATKFGITAYNHPLNLTKEQLSQVALMTTSVDVLVSICVIFAMSFVPASFVVFLIQERVNKAKHMQFISGVQPFLYWLANFVWDMCNYIVPATLVIIIFVCFQQDAYVSSTNLPVLALLLLLYGWSITPLMYPASFFFKIPSTAYVVLTSVNILIGINGSVSTFVLELFGSNEIGGINDILKNVFLIFPHFCLGRGLIDMVKNQAMADALERFGENRFRSPLAWDMVGKNLFAMAIEGVIFFCITVLIQYRFCIKARSSTSHLKPIGEEDEDVARERQRILSGGGHTDILELRQLTKIYKRKQKPAVDRLCVGIPPGECFGLLGVNGAGKTSTFKMLTGDSDVTGGEAYLAGKSVNSEIDEVHQNMGYCPQFDAINDLLTGREHLEFYAILRGVPEKEVCEVAEWGIRKLGLVKYVDKSAGSYSGGNMRKLSTAIALIGGPP**VVFLDEPT**TGMDPKARRALWNAILSIIKEGRSVVLTSHSMEECEALCTRMAIMVNGRFRCLGSVQHLKNRFGDGYTIILRVAGPDPDLRPVMDFIEHELPGSTLKEKHRNMLQYQLPSSLTSLARIFSLLSKNKEALSIEDYSVSQTTLDQVFVNFAKDQSDEDHLKDVHLNKRDAVVVDFSQLNSFLTDNKTRESCV |
| TRINITY_DN86307_c0_g1::TRINITY_DN86307_c0_g1_i1::g.26593::m.26593 | gi\|657595654\|ref\|XP_008302497.1\| | PREDICTED: 40S ribosomal protein S11 [Stegastes partitus] | 90.2 | RACIGLFTTLLAKM**ADAQTERAYQKQPTIFQ**NKKRVLVADGGKEAKEKLPRYHKSVGLGFKTPREAIDGTYIDKKCPFTGNVSIRGRILSGVVTKMKMQRTIVIRRDYLHYIRKYNRFEKRHKNLSVHLSPCFRDVTVGDIVTVGECRPLSKTVRFNVLKVTKAAGAKKQFQKF |
| TRINITY_DN58867_c1_g3::TRINITY_DN58867_c1_g3_i1::g.23632::m.23632 | gi\|923867160\|ref\|XP_013770704.1\| | PREDICTED: dysferlin [Pundamilia nyererei] | 16 | LSTVMEQAEDWAGRLRTMSEEPQNSLPDIVIWMLQGDRRVAYHRIPAHTVIFSQEHCGKHCGQLQTVFLKPPQSSGAEAKLPGQLRVKVWFGLAADVKHFNQYAEGKLSVFAETYENQTRLALVGSWGTTGLTYPKFSDVTGRVKLPKESFKPSPGWSWAGDWYISPEKTLLFDVDAGHMTFTEEVFENQMRLPGGQWIGMPEGYTDVNGEKAVPKDEVECPPGWAWEEVEWSEDLNRAVDDQGWEYGITIPPDRRPKSWVPAEKMYHTNRRRRWIRMRRRDQQKMEALRKQRPDEAEREGWEYASLFGWRFHLKPRKTDSFRRRRWRCRMEPLEKTGPAAIFALECSLSSIEDKNDDKSVTTTFGVNRPTISCFFDRGTRYHLRCYLYQARDLPPMDKDSFSDAYAVVSFLHQSQRTVTVRNTLNPCWDQTLIFYELEIFGDAEATTSSPPNVVVELYDQDTYGADEFMGRCVCQPTFTPSPRLAWFPIRRGDKSAGELLAAFELIQREKPSIHHIPGQEGDIMTTTHVLDELMFSGFLSENLQQWPDESDLPYPPPQREPNVFMVPQGIKPVLQRTAIEILAWGVRNLKSFQMSSVTSPSLQVECGGATIQSCVIRSVKKKPNFDVNTLVLDVRLPREELYMPPIVIKVIDNRQFGRKPVVGQCTIRSLEDYRCDPEAEGATVEEDEEGWRREMPHYSGEVFIDIDDELPLVTDQEEEFMDWWSKFYASTGEKNKCGTYLERGFDTLKVYDQELEKVEGFGGLSDFCQTFKLYRGKTQDEGEDPSVVGEFKGMFKIYPLPDDPSAPVPQRQFRKLPPNGIEECLVRVYIIQAHSLQPKDTNGKCDPYVKITLGKKTITDHDNYIPCTLEPVFGKMFELTCSLPLEKDLKVTLYDHDLLSKDEKIGETVIDLENRFLSRYGALCGLPQSYCVSGVNQWRDQLSPSRLLNRLCERRNLKKPVYQDDVVHFRGDQHTAADLEDKHESKRHLGPIRERLSLHVLRKLGLVPEHVETRRLFSPLQPDIEQGRLMMWVDLFPKSLGPPGPPFNVTPRRAKKFFLRCVIWNTSDVILDDVSLSGERMSDIYVKGWLDGHEHIKQKTDVHYRSLGGEGNFNFRFLFPFHYLPAEQLCVVDRKAHFWSVDKSETKLPPKLTIQIWDNDKFSFDDYLGHLVLDLNRMLRPAKSPQKCDVDLLQQPPEKLVSLFEQKTVKGWWPCTSEQDGHKSLAGKVEMSLEIVSEQEQDERPAGVGRDEPNMNPHLEEPQRPETSFLWFSSPYKTLKFILWRRFKWFIVLFIILFFILLFLGVFLYSFPNYAAMKM**VGPFGPATKQ** |
| RRRRRTRINITY_DN112158_c0_g1::TRINITY_DN112158_c0_g1_i1::g.135108::m.135108 | NA | NA | 6.2 | SVNLLASIGIADLVEKKSAHLASGLYLFPLIEVPGGQDHHPTGCSSCASDISSQSNLSPLSKTKLCFEPYESFFRDYGGKLLYIRTGSSD**RSLANLVL**TVTSDEKVSDSDQTREDYLVVASYMGSRFRGR |
| TRINITY_DN53068_c0_g1::TRINITY_DN53068_c0_g1_i2::g.16031::m.16031 | gi\|548525400\|ref\|XP_005751719.1\| | PREDICTED: T-complex protein 1 subunit delta [Pundamilia nyererei] | 38.7 | M**PEAMMAPKLS**VAGGRHKGGAYVDRDKPAQIRFSNISAAKAVADAIRTSLGPKGMDKMIQDEKGDVTITNDGATILKQMQVLHPAAKMLVELSKAQDIEAGDGTTSVVVIAGALLDACSKLLQRGIHPTTISESFQKAVDKGVEVLTSMSRPVLLSDRDTLLNSATTSLSSKVVSQYSGLLAPMSVDAVMRVIDPATATGVDLQDIKVTKKLGGTIDDCELVDGLVLTQRVANSGVSRVEKAKIGLIQFCLSPPKTDMDNQIVVSDYAQMDRVLREERAYILNLVKQIKKAGCNVLLIQKSILRDALSDLALHFLNKMKIMVVKEIEREDIEFICKTLGTRPIAHIDHFSPEMLGSAELAEEVNLDGSGKLVKITGCTSPGKTVSLVVRGSNKLVLEEAERSIHDALCVIRCLVKKRALIAGGGAPEIELAVRLAEYSRTLAGMEAYCVRAYADALEVIPSTLAENAGLNPISTVTELRNRHAQGDKVAGINVRKGGISNILEELVVQPLLVSISALTLATETVRSILKIDDVVNTR |
| RRRRRTRINITY_DN34201_c0_g1::TRINITY_DN34201_c0_g1_i1::g.112708::m.112708 | NA | NA | 55.4 | SFLFIKFTHMLLSLKADDPTAGTEVTATDGTGAATTATVRGTKVEVAAGPAAEATDATAVDTVGGGATVRGVAAGAGTGARAPAGAATATGGAAT**TVTAGGTAAPAG**EGGAATATPAGGGGGATPAEREIPATRSGQESGATDAGRWRKEGERGAKKGTRGEEGKRQQETKVRCMVM |
| RRRRRTRINITY_DN28079_c0_g1::TRINITY_DN28079_c0_g1_i1::g.5639::m.5639 | NA | NA | 23 | TQEVGWHVYDSSPQCRVAAAAAARKQPTKYGDAPSSTAELPRKRNEMNVPCVSQSVPAEEPPPSSSLFSLLPSLPSS**LSSLSLGALSLGG**PNGGRRRRSSSVASRYSQVFVSPLFQGPKTNE |
| RRRRRTRINITY_DN100510_c0_g1::TRINITY_DN100510_c0_g1_i1::g.132784::m.132784 | NA | NA | 21.4 | PSSDLLQQLQPQILNLFPKYGLCDTSAWSDVALLGNRVSQVTEQPVEPSDLFGIVQKIVNADRLILGIVVCWELCGAEMFVHLLYRLQVQSKHPGKNALEMSIHELEAQSLSLTSSNEDVMTWD**GDVVSSTET**LDTASGISYEEVKNTLLSLFADHTQPSHQNSHASSATDMDKARQGLRDLWNHNT |
| RRRRRTRINITY_DN57018_c0_g1::TRINITY_DN57018_c0_g1_i1::g.105264::m.105264 | NA | NA | 8.6 | TVGPKRFVRLAICGLFVAILGFFICGSISLLVRRKLLLNKTKFHDSNKSYLPREDRVGCCTYSSQKPDNQPTVFHKKSMGDIPQDEKFWTYNWTMNKSEVMMVECTLVLSDTSFVESWGTLLVIKAQPREEVNLQRVKSQDSHF**VEKTGRKV**KCSYLGTNATVVSSITHNNGSETLQTNDKYWLYEWGSSINFHCTFSVSDGTHMDEHEPNQVLTVNPKDDYVSLTLESSIISNVHRTKLKASCSYQGRHSASASSISLFRQDSDFSVAKGPQVKQGDKYWTYKWNTSGDNWCEMQMSESPFVDLWQTVKKLSPVPIELIRLYRGSPEASYESKDRKAVCKYTGSDSSSANHIPFDHNNPLPVEDKYWQYKWGSSVGVSCEFFVTEGEYMTNVDPNQTILAKPKEGQVQLQIAQSSDTHFVQNRGRKAQCTYLGSDATVVSNVSYMRDSAGLRNSDKYWLYEWGSSVNIHCSFSVSEGPYMVSYEPDQTLILRPKEGYVTLTLPNSSSSQVSRG |
| RRRRRTRINITY_DN56638_c1_g1::TRINITY_DN56638_c1_g1_i1::g.124257::m.124257 | NA | NA | 43.6 | GHGFRGLHRHPSGRHRPPLHLLLGDQLGAQHTPEASVASVVPHGGDGGGLRAGGGGAGHSEKKEEQIEEGSSQGHLEAPREHH**GAAGRAGQVPAH**RGVPLAAPLRREGSDPREKGEEESKGGVGVHQGLGTEARPHPAPQEPCMKAPQAPSPRVPGPPLDVHHRRGDCGSCAEPQCARGRGLGRQPAPPAHVHPPWLFLLRASRQHQRGAPRLAEGEDRRHRGLGKQHQLVRPFVCVQSRGHHPGGAEEGPPQRHHLHASRRDVEEPPFHLEGEAPTRGPQREAQSHQLGAAPGAAAAPVHEEALGRLRAARRAPPAAGGAVGVAG |
| TRINITY_DN108373_c0_g1::TRINITY_DN108373_c0_g1_i1::g.134391::m.134391 | gi\|734650230\|ref\|XP_010754438.1\| | PREDICTED: protocadherin gamma-A4-like [Larimichthys crocea] | 24.1 | YRRIHSELLLQFLLGGKTVYIVISSPYDIRKLKMDSKMSWVQMCSICFLLLFFNVAHGDMSYSFPEEMKRGSVIGNIAK**DLGLETGVLSKRRAR**IDTDGTDKRYCDINLNNGELIVADRIDREGLCGKKASCILKHELVLENPLELHRISLHVQDIND |
| TRINITY_DN9128_c0_g1::TRINITY_DN9128_c0_g1_i1::g.78330::m.78330 | gi\|657581759\|ref\|XP_008294911.1\| | PREDICTED: tectonin beta-propeller repeat-containing protein 2 [Stegastes partitus] | 17.2 | YSSPGPGGGVASHSGPANQTERTTDRQDDPDRDTHKPDQLAESWM**GYSGPGCGIL**SLQVTDRYVWCLDFKGGLFCSPLPETGLSWQRFEDNVHQVALSPTGNLLWKVEQKSMTAFACAKVAAKGKRHWYKAAEQTAFVALSDDSAWIIRTNGDLYLQTGLSVERPCARSVKVDAPCVFSQVCVRGGVVWALSEHKAVFYREGLSSFCSE |
| RRRRRTRINITY_DN134767_c0_g1::TRINITY_DN134767_c0_g1_i1::g.141463::m.141463 | NA | NA | 21.2 | IPGQNSHPWYSFIKLKDKVSALKFRCFNNGPDNIRTLDIQEFLFKLTKFFRNRFSQSSAVVFSLFFANN**SIFTTLDT**AYSFTHPTMFSFGAQINYSACM |
| TRINITY_DN43403_c0_g1::TRINITY_DN43403_c0_g1_i1::g.70386::m.70386 | NA | NA | 13.3 | QRRGSLIHNGRHDGAGVLCPEQRQHDPLSLFRGAHRGVLTEGASGAGVRVQQPSVGAAASRRAPLRVLSDHQHAH**AGQHISHLRT**QEELRPEEAARRLREDPGRPPQPGGLGPQLPPGGDTLPAPRLLSQGLPQPDPAESYNAQSGFLHPRRQEPAAHHRPREDRHRGRQAGARRRPPPAQPHRSLLCLSGRRNLDSHLPPSL |
| TRINITY_DN49775_c0_g1::TRINITY_DN49775_c0_g1_i1::g.57280::m.57280 | gi\|657557850\|ref\|XP_008283049.1\| | PREDICTED: 26S proteasome non-ATPase regulatory subunit 14 [Stegastes partitus] | 17.1 | MDRLLRLGGGMPGLGQGPPTDAPAVDTAEQVYISSLALLKMLKHGRAGVPMEVMGLMLGEFVDDYTVRVIDVFAMPQSGTGVSVEAVDPVFQAKMLDMLKQTGRPEMVVGWYHSHPGFGCWLSGVDINTQQSFEALSERAVAVVVDPIQSVKGKVVIDAFRLINANMMVLGHEPRQTTSNLGHLNKPSIQALIHGLNRHYYSITINYRKNELEQKMLLNLHKKSWMEGLTLQDYSEHCKLNETIVKEMLELAKNYNKAVEEEDKMTPEQLAIKNVGKQDPKRHLEEHVDVLMTSNIVQCL**AAMLDTVVFQ** |
| RRRRRTRINITY_DN58559_c1_g2::TRINITY_DN58559_c1_g2_i2::g.62478::m.62478 | NA | NA | 16.7 | VEVTVTPGNSTDELPTTETPASSEKAAEEKKDGDAVLPTNEAAGQQESSTTSAPQEAKEEAKEEAKVEAPQEAVKTENVIQVSDAAAAQATKVEGNQVPFLGELADYLFQYQELSTVMNQREKRLSKVVQFVDVLKETKASDLLNWMACFIGSRSSGDNCHVVATQSKSHKIEKMMDTLDQAKEPVESKGWKLFQFQKVSRSDKRKVHRILMNRCIFSPTIETSEVEVEFDGFTKKEKDWYACDSEQDEESCDSLMVITTARKQYVMLLFDSMTDPLPTQAAIFARQGWYGHIYSANIYKTADEDEEDSSEEEEDEDPESDRSHGEDVKLLVRNYDYPMVASTRNKKKNEETVGTNSTRWNKYSPLREFEDEMLTPEDSKQKLTSLTSHLESLSIETEGFQNHEVLAQHILIYQAEVQVMLCRQRRLRVVY**GYVDVRGEAELGE**MMADIGIYTGTRGVGASCHVVIPGSFFNKFANVRRRLKLLLHPEGPVGHDPWSMFQIHTVERETSKERKNILTLHRITYDPCHDESTLKVIFEEFIETERDPSPWYQACKVRNGEECRTVMVIISSQQEWVMRWFDGVTEEKPGQAAIYKKAEKYGDIYSANIYDCGPDGNGTSLQVRNYDYPLIDVYRNKPANYTKKAEKVTFRSFIRPVSQFEALFLRGEDALKRKYAELLAEAAIPEVPMLNEEDNATSILMMNETLDHSQRRKLIFIKYIVFLLAVTTLIILFVLFGIVAKDNYFTTVDKEAPESELRGNFVTVQVKYTTSYSLDKFEFKCKHNEKEKKVKGGLLLKARFKREPGNFPGHYECSVDITNHEPQPTKVKGNQVPEPKGIGTRERLETQTADREMDKYRPRVKCTYETFPNLGTVDCTGGPQHNRPTVTGTSHRKPHHLEDCSCDYSLNGLVPHCKQSTTDWTFQISTSTKDQLRNSITFDCDIRV |
| TRINITY_DN38010_c0_g1::TRINITY_DN38010_c0_g1_i2::g.125166::m.125166 | gi\|734610060\|ref\|XP_010732368.1\| | PREDICTED: A-kinase anchor protein 13 isoform X1 [Larimichthys crocea] | 14.4 | VDSKVNEHEKRRRLKEFHSRMDSKSIMMMKSGQIFAREDLLRRRLIHEGALQLKNSQGRLKDIHALLLSDIFVFLQEKDQKYVFAMLDQRSTVMSLQKLIVREVANEERGLFLITAGIEKPEMMEVLASSKEERNTWMQLIQEAMQSMEKDEDEGIPSETEDDKRQLETKAKEMRDLLRQKDTEIMSLLEEKVRLFRGMWEGLSPGEEACQQVEPFFRSTCSLEPPRGATIMNDALLEVETLQALVNGSLGGAMASVQEEGGVGPVCLPRRAETFGGFDSHQMHSNKSGDKEEGEDTVGDLRRTESDSVLKKGGNANLLLLLKRNSEQVLHSVSNLHLLLNTLQAVVVQQDSFIEDQRQALSERSSSCTSLSRPSSRPNSLIEQEKQRSLEKQRQELTSLQRQQAAHAEEKRRREKDWELREHHLTEREMLVNMQEEDVLKQHKELEWEKQELQSKKEEYQKDLERLRDAQRKLERDREAVQRQLDKMEELRLAERTPSTTSDESQFPGSSQSLELDPMELSSSSHASLPRLLPQQSKPKGKGLNPFTSSSTNANLKGSDSNSQISKSLLQLAKSKSKEGKEKKKKKGKGGSAQTADPQHRPEPPLDGEIFFC |
| RRRRRTRINITY_DN40656_c0_g1::TRINITY_DN40656_c0_g1_i1::g.8890::m.8890 | NA | NA | 15.1 | CFQMGAVRGNKMIVDRPSLFPLFECKEEKALEMEEPVARINTFGKRFCVFVRKAGCRLASTACDFATDGAGLVIVVGRIEPLAARCQCMGKKSASAVLPLFDKSTFFGQDMTLGQFIKARNAQPLGIGIFVAKFGEQRLSMLTMGNVGLGKECVVKVGLDKMLDIEFQVVEYPLRFQPIEATSLGGTYKQREFVTINDYGLRALFTACSISAPGCGILAIPTHYSEPMESPPPLEPNRVQAIGMKSFVETAFQQLGGINIPGEESAYLNCGGVCLDSTPCVMGCTLGLPNDSLISRAAGYYNKNAISTIFSKIDLNTPCSKQCPADACKLCRMAERLAGRESLTTHKIDDFNNELKVCSDCSREPNRKWHKKEKKKNATSVIQAHTKVRPNLALISEIDPLDKSLM |
| TRINITY_DN51738_c0_g5::TRINITY_DN51738_c0_g5_i1::g.58543::m.58543 | gi\|1007710542\|ref\|XP_015825806.1\| | PREDICTED: keratin, type I cytoskeletal 19-like isoform X1 [Nothobranchius furzeri] | 33.5 | MGMGGGAAFDLSGALDQSTVHLNEKATMQNLNDRLASYLEKVRSLEAANAKLEKQIREYYEQKGPAAERDYSKYWAIINDLKDKIAGATIGNANILLQIDNSKLAADDFRTKFEHELMMRQSVEADIANLRRLLDQTTLTKADLEMQIEGLQDELAYLKKNHAEELEAMRAQLTGTINVEVDAAPQQDLNKVL**EEIRAQYEGITD**KHRRDQESW**FNEKSATLS**KEVAMSTETIQTSKTEINDLRRSLQGLEIELQSQLSMKGALENTLAETEARYSSMLAGYQNTINMLEQDLANVRASIEQQGHDFKMLMDIKTRLEQEIATYRSLLETEESRPISTGGGKTTITTTT |
| TRINITY_DN60889_c0_g1::TRINITY_DN60889_c0_g1_i1::g.48167::m.48167 | gi\|962014024\|ref\|XP_014832040.1\| | PREDICTED: DEP domain-containing protein 5-like isoform X4 [Poecilia mexicana] | 18.2 | NQKTLEEQQQQQQAKPSAAVSEPSNITTAPTYVDSPRKDAAFILDFIRSPRSSYIYHSQLPVEANEAADKGV**QSGASGGAAAAQPAG**ESVASSSTDTSGLSAAGVLSLSSSSTPHELLEAIKHPTTGVQLLPEQKGLPFNCFISAEVVHWLVNSVEGVATQGMAVDIMQKMLDEGLVAHASGDAMRTFVYGFYFYRIVGEKDGQTSQLT |
| TRINITY_DN88439_c0_g1::TRINITY_DN88439_c0_g1_i1::g.27095::m.27095 | NA | NA | 30.3 | LGVDPEVLAGRPV**SCRVVAPDLN**EVMGVWLHSLQPGVVLLAGHHHLLSPTLTVFLIPPVLHLVSINIALCEGQPPHDGLCGGESFSVNTCRSFRWSILC |
| TRINITY_DN99402_c0_g1::TRINITY_DN99402_c0_g1_i1::g.66290::m.66290 | gi\|1007706830\|ref\|XP_015807780.1\| | PREDICTED: nuclear factor NF-kappa-B p105 subunit [Nothobranchius furzeri] | 9 | GSVKLTAL**LMAAGADPHRENF**EPLFFREEEEEEEEEEDEGYIPGTTPLNMAATAQVLELLSGKEYEAQSAAPVSTPPGDLSSLAVEVKQEVSRALESEGCWESLAHSLGLGILNTAFRLSPSPAKTLLDSYEVSHTHTHTHIRSL |
| TRINITY_DN57246_c0_g1::TRINITY_DN57246_c0_g1_i1::g.21598::m.21598 | gi\|734640730\|ref\|XP_010749218.1\| | PREDICTED: vacuolar protein sorting-associated protein 11 homolog [Larimichthys crocea] | 12.7 | MAAFLQWRKFVFFDKDTVKDPGDNGKNFALPLGISACDSGRGHIVLGDMDGKIWLMTRSLQLSCFQAYKLRVTHLYQLKQHSILVSVGQDEQGINPLVKVWNLDKRDSGNPLCTRIFPAIPGNKPTEVSCLSVHENLNFMAIGFTDGSVVLTKGDITRDRHSKTLTLHEGSSPVTGLAFRQVAKVTHLFVATLEKVHCYTLSIKEYPKIELDTHGCGLRCSSLADPSQDSQFIVAGDDCVYLYQPDERGPCFAFDGHKMLAHWHRGYLFLLIRDAKSPNKTGFASRESSPSDKQLLTIYDLDNKFIAYSASFDDVIDVVAEWGSFYILTREGKMFVLQEKDTQTKLEMLFKKNLFVMAINLAKSQHLDSDGLSEIFRQYGDHLYLKGDHDGAIQQYIRTIGKLEPSYVIRKFLDAQRIHNLTAYLQALHRQSLAN**ADHTTLLLN**CYTKLKDSSKLEEFIKCSESEVHFDVEIAIKVLRQAGYHSHAVFLAEKHMHHEWYLKIQLEDLKNYQEGLRYIGRLPFEQAESTMKHYGKTLMHHVPEGTTLLLKGLCTNYHPSGDTAERDSPERSRVNKANSEEFIPIFANNPRELKAFLEHMIKVDPRSPQGVYDTLLELRLQDWAHERDPQRKKVLQGEAVSLLRSDNTVFDKALVLCQMHNFKEGVLYLYEKGKLYQQIMHYHMQNEEYGKVVEACKRYGDQESCLWEQALGYFARKEEDCKAYISEVLQHIDQNNLMPPLLVVQTLAHNSTATLSVIKDYLINKLQRESQQIEDDERKIHQYREETAHLRSEIQELKTSAKIFQKTKCNMCNSPLELPSVHFLCSHSFHQHCFESYAESEAECPTCTPENRKVMDMLRAQDQKRDLHDHFNRQLRCSNDGFSVVADYFGRGVFNKLTLVTDLPGNKTVGSLEVNLQRDLLIHTKRNC |
| TRINITY_DN58520_c0_g5::TRINITY_DN58520_c0_g5_i9::g.90647::m.90647 | gi\|548380238\|ref\|XP_005734223.1\| | PREDICTED: histone H1.10-like [Pundamilia nyererei] | 52 | M**AEVAPAAPAAPA**AAAPAKAAKKKPAAAKPSKKSGPSVSELILKTVAASKERKGLSTVALKKALKAAGYDVEHNGAHVRRAIKSLVAKGSLVQTKGTGASGSFKAGKSAEKPAKKKAVTAAKKKPAAAKSTPKKAKKPAAAAKKAAAKKPAAAKKPKAAKTTPKKAKKPAAKKPAAAAAKKTGKSPKKAAVKKAAAPKKAAAAKKVAKPKAAKPKKAAAKKTGKK |
| TRINITY_DN35879_c0_g1::TRINITY_DN35879_c0_g1_i1::g.32401::m.32401 | gi\|734629481\|ref\|XP_010743032.1\| | PREDICTED: PDZ and LIM domain protein 7-like isoform X2 [Larimichthys crocea] | 41.8 | TNAEDMTHVEAQNKIRAATDSLTLTLSKAFKTGGDQKDSLAEASVQPKYSFAPSTTINKMARPFTAGGGSANSGP**VIKPVAYS**PKLNTPSSQGRASMQQPQNG |
| TRINITY_DN46987_c0_g1::TRINITY_DN46987_c0_g1_i1::g.98828::m.98828 | gi\|47218682\|emb\|CAG12406.1\| | unnamed protein product [Tetraodon nigroviridis] | 18.6 | M**ADKIKDAKII**FVVGGPGSGKGTQCEKVVTKYGYTHLSSGDLLRAEVSSGSERGKQLQAIMQKGELVPLDTVLDMIKDAMIAKADVSKGFLIDGYPREVKQGEEFEKKIGKPCLLLYIDAKAETMVKRLLKRGETSGRSDDNEETIKKRLDLY**YKATEPVIA**FYESRGIVRKIDSELPVDEVFGHVAKAIDALK |
| TRINITY_DN49859_c0_g1::TRINITY_DN49859_c0_g1_i1::g.119995::m.119995 | gi\|808861831\|gb\|KKF13621.1\| | Protein IMPACT-B [Larimichthys crocea] | 3.1 | MADIITQENEGDLQAQIEEVEALSSIYGDEWCVIDEASRIFCIKISNDLDKPKLTACLQIILPPDYP**SAAPPIYQIN**AAWLRGPERAKLANSLEDLYVEHMGESILYLWVEKIREFLVEKSQSSETVDQPEKVNLTAEEEVDDDDDDEDIPDFRALKLNTENAHLFMDHANGEELPPIKHGNPITDRRSTFQPHLAPVVTPRQVKMVLENLYENKKIASATHNIYAYRIYCEDKHSFLQDCEDDGETAAGGRLLHLLQILDVRNVMVVVSRWYGGILLGPDRFKHINNCARNILVEEGYTASSDESARAGGKTKKPKSKKTK |
| RRRRRTRINITY_DN54223_c0_g1::TRINITY_DN54223_c0_g1_i2::g.59145::m.59145 | NA | NA | 15.5 | EPQPQKELMSKLKKFKNKGMRNKNQPKMKKAKTKKEFRSGEKKGAKSAKTEEATKVAPKKAEAGKVTEGEMKAMKAKKPGTSSNKKGKSKVNAKDKAAKGNTQAKEKLGGAKGPVTKKAKDGKSPKEQPSET**VTEPAEPAE**SSEEAKGAPTTPIVNSFKKLKAVFFGDMNHSHPYFRRTLRLSPHFRREKFRTFGEKGFDLGTPVLKVNRKKLAYDVVWENEEVMISCTCYVLYGGSPSEANVSDIASLILEKQLHASRQIDAEDKSTKVAPDKAIVGTGSCPADLLVRDFGGMVKPFQRGDYNCVVTNTVGLRHINGVVSKLRDANADNAVITGTNRMLQAVYTTKGGPASSMDLVLEGEQPSLAMVPLFSSAGQLMYQGALYEPTAGIPVSSDYIVLGVKSWKGLPDLNVGRNILAQALDRRRTKLTNTRITVPRHIENAELFDVLESLPFLDILKEILFNNYSYYMCLDKKLLSIYEAREKGEERKTSFNCLVDINDKIRQYITKLDLPLVGEKEAEEAGPLRFKDMDDINAQVTDEEDMDADSKDEDDDDDDEEEE |
| TRINITY_DN9126_c0_g2::TRINITY_DN9126_c0_g2_i1::g.78281::m.78281 | NA | NA | 11.2 | ARLYRSSTAAAVYQLWKSHKRRTTSSRLCSAGPVELYHVSMVSVVVIVTHRLRQPISAERQATDGRRLGISHLALVCIDLVICKLVERTFSEQFNDQW**EWIINCIFT**IIYKIILNIQYIYTWEMN |
| TRINITY_DN54984_c1_g1::TRINITY_DN54984_c1_g1_i2::g.60393::m.60393 | NA | NA | 6.3 | LLLRPPRHRLLTKDAAGRSDGPRSSPGTGVAEVIRLSVVVVVGPQRAGVK**WTPPPTH**RHLLPGPRSPLSLSLTPENHSRLVFTDKTSGQDGSVGVSGGARPALQEEEEEEED |
| TRINITY_DN53776_c0_g1::TRINITY_DN53776_c0_g1_i1::g.76810::m.76810 | gi\|226524991\|gb\|ACO70859.1\| | glycerol-3-phosphate dehydrogenase [Rachycentron canadum] | 75.9 | MM**AAPKKVCVIGSGN**WGSAIAKIVGANAAKYDKFDTTVNMWVFEETVNGRKLTEIINTDHENVKYLPGHKLPPNVLAVPDLAESVKGADILIFVVPHQFIVRVCDTIKDHIKKDTIGMSLIKGVDAGPEGLKLISEVIRAKLGITMTVLMGANIANEVADEKFCETTIGCKDATHGPLLKDLMQTTNFRVTVVPESDVVEICGALKNIVAVGAGFCDGLGFGDNTKAAVIRLGLMEMIAFAKFFCTNCPVSPATFLESCGIADLITTCYGGRNRKIGEAFAKTGKTIEQLENELLNGQKLQGPATATEVHQILKQKNMVEKFPLFTEVYQICFNNHPVKEFIKCLQNHPEHM |
| RRRRRTRINITY_DN49506_c0_g1::TRINITY_DN49506_c0_g1_i1::g.76464::m.76464 | NA | NA | 16.4 | VGGADSYPQGALECCLRTSTLETVSVYFFFFFFVQQTLPLRNRRTVDPGGCGGPNYIRCPHRFLGRELRLLVRYSLLTPRDSSCCDHCWSSCLLPNQLTQICSLDHDPVSAKDEGQLLSAVHLSPRPCRFSSGLVRFCHPLQVPLEQSCAGIDHLR**ESEVGTVVFQ**HVSAVSLAEGPLLYVFRSTVQGALHGVLTISQHGSSDRLGGRVEAALTLIVNLGDELLLELDEDLFVYVEQDCLVELVHFLSLPCDLSQLGGALWHSRSHANSRVSVQVADARSVTSDSQDFSSSLNHANVAVEVWLGTHLSDLHHLLSHVLGPKLSVVHALRQGVVGRVAVALTEDVQWLEGETQLRPDQGSPVHLFIGTFRVVNPDTVDAAATSKFGIVDTGQSLLQRRLPAGQWSTRLLMAGPATAQLHVCLRPDSCPGTTVVPLCQLRRRTTREAGGHGCRVAPADGFAVRVQHLLLLGGGLLLQLSEKVLGVFAVSSRRRRRHM |
| RRRRRTRINITY_DN89913_c0_g1::TRINITY_DN89913_c0_g1_i1::g.108349::m.108349 | NA | NA | 7.3 | YCNAPVVSVIAAQTTPTKTMPADPATPPASTSTQLTEQSDSFCFAGFLRDPNAPWKVLGTKNKGCNMDPTLRPIVAVGEAIWGYKCTELEQQLAEQMQEKTAMTARVSQCA**DRAATFNLTYDDGE**ILLFVGAPRHGKPIQVLESESAPFLAAFCLLLFQTFCWVAAMNYNLHTYSSSAFRFFCMLHICLIFI |
| RRRRRTRINITY_DN42315_c0_g1::TRINITY_DN42315_c0_g1_i1::g.33740::m.33740 | NA | NA | 31.9 | LKGLKTEMPLIQSSQRTNHTKHTQEPRLSHIELKKHTPTHESTTKPEPLLLLLLLLLVLPPTFSSPPLLVGVPGAAQLLAVNGPVVLLEPHVEQASRREPGGDGVVEEEPVSSSLDRPLVEDVVVQGLSRHVPQ**LDDVLSVDL**DHESGPLRRHVPLVDPEVDAAHVRKLPVQLRSLVLQADLSLVPQRHDVGGLAVAGEQEAHEGGHQDDAEQ |
| TRINITY_DN46704_c0_g1::TRINITY_DN46704_c0_g1_i1::g.11750::m.11750 | gi\|808861050\|gb\|KKF12953.1\| | Stromal interaction molecule 2 [Larimichthys crocea] | 19.4 | MSYIHKVVNVLLRGLVIYAVLGSQCTTHSNDLPDHSSDAANVATTDPCLTVMPPCIGEADRFSLEALRHIHKQLDDDNDGGIEVNESVEFIIEDMKQQQTNKHSNLHREDQHITVEELWKGWKTSEVHNWTMDDTVQWLKESVELPQYEKNFRDFRVTGNTLPRIAANEPSFMSMQLKILDQRHKQKLNLKALDAVLFGPPLRPQHNWMKDFVLMVSIVIGVGGCWFAYVQNKSSKVHISQMMKDLESLQTAEQSLLDLQSRLEKAQEENRTVAVEKQNLEQKMRDEITGAKKEAHRLRELREGAECELSRLKYAEEELVQVRKALKRAEKEMQSERSVPEALQKWLQLTHEVEVQYYNIKKQSAEFQLCVAKDEAEKIKKKRSSVFGTLHVAHSSSLDEVDHKILEAKKALSEVTACLRERLHRWQPIEKLCGFPVVNNSGLPSLTASLYSDHSWVVMPRVSVPPYPIAGG**VDDLDEDTPP**IIPQFTSATMIRPSLTRSSSLCRSRRSLLSSPQSSLMSPDPDLLSMASSSLSYHPEADDEHIMFSSDRRGEPAQDGSSDTDSLNSSMGRKQMHNPGSETPYRKISREELLLFSQSSELPASASSAATHSSSSSGSLRDSTPP |
| TRINITY_DN52135_c0_g1::TRINITY_DN52135_c0_g1_i1::g.68639::m.68639 | gi\|657544995\|ref\|XP_008277879.1\| | PREDICTED: transcription factor BTF3 homolog 4 isoform X2 [Stegastes partitus] | 29 | VCCIAAGLSCAAASHSPAARTFPRAVSEFPHQKNT**MNQEKLAKLQ**AQVRIGGKGTARRKKKVVHKTATADDKKLQGSLKKLAVNNIAGIEEVNMIKDDGTVIHFNNPKVQASLSANTFAITGHAETKQLTEMLPGILSQLGADSLSSLRKLAEQFPRQSMDMKAVKEETAEEEDDDVPDLVENFDEASKNEAN |
| TRINITY_DN33998_c0_g1::TRINITY_DN33998_c0_g1_i1::g.6993::m.6993 | gi\|542227779\|ref\|XP_005452844.1\| | PREDICTED: eyes absent homolog 3-like isoform X2 [Oreochromis niloticus] | 18.1 | VGKAKGKGKKSEGSAPSDTDLERIFLWDLDETIIIFHSLLTGSYAQKFGKDPATVLNLGLQMEELIFELADTHLFFNDLEECDQVHVEDVASDDNGQDLGNYNFLADGFSGPSGGGGASGATAGVQGGVEWMRKLAFRYRRLKEIYNSYKGNVGSLLNPMKRDLLVRLQSEIEGVTDSWLSTALKSLLLIQSRGKCM**NVLVTTTQLVPA**LAKVLLYGLGDVFPIENIYSATKIGKESCFERIVSRFGKKVTYVVIGDGRDEEFAAKQHNMPFWRISTHGDLVSLHQALELDFL |
| TRINITY_DN31463_c0_g3::TRINITY_DN31463_c0_g3_i1::g.107825::m.107825 | NA | NA | 55 | MFSPTVTSPEWGFSVKSPVRSCLVSGGCPSSTTG**GASCAGAAVSSPAGA**GAGAGGAESGCGSSLDLAGGSSLDLAGGSPLDLAGGSPLDLAGSLALAAFLTGSGILNGLLSSTIVCDSLF |
| TRINITY_DN58798_c0_g5::TRINITY_DN58798_c0_g5_i6::g.92290::m.92290 | NA | NA | 14.6 | MRDDDGDFTLHELIEHFHGLTGLALICLDEVFQGDYQTWDDRVHLVLLFCADEGSYGCEGQNAIIQGCWV**AVVLQYSAQQLQ**QLAVVRLEGLRVGIHHFIQQQEANLSVIRIATLGGLTEEMQEGGPAVRCLMFDHHCTQLGQGVLHCVLCFLHEGLQHHLLEALSVVRAYLLPMFLYSHSQHHCSRLADLWVLGATHDSTQGWNNGWVFKSLFQLGCGLLNNRVVRLCHLQQDLLQFGCHLDSSAATLPAASSDDPCDR |
| RRRRRTRINITY_DN57902_c3_g6::TRINITY_DN57902_c3_g6_i3::g.128457::m.128457 | NA | NA | 10.8 | KKPRSGGGKEKEFQAIQNFINLLNAPLDKKPTAG**TALTVSRP**NGATVSRERIEDEERRKRGEKERKEGGVESLEERGRNEYRGGDFSNRKSRKSVSASNASYESRVSSRRSYESDASSCSVKRRRNDDLSRRRKSDSHSRKDEKGRERNKERRERSRSRREERDPSVSSRRRKARSEEGDGGKRERRSSDSEENESAASLSYLQCIRREEEADEEDREGFGPGGKQNLFTASTNPPAPYWEVEEEEEEDKEKRDRESKGEESRRSERSSIRRQRKGGRESRRRKKKKKKSRRRSSSSSSSASSSRTSTSSSSSSSASRKRKKKM |
| RRRRRTRINITY_DN13697_c0_g1::TRINITY_DN13697_c0_g1_i1::g.127795::m.127795 | NA | NA | 8 | PRRLSKLFERTIFGSGYAVLLGDKNMSSALMAFLAEYVGYKLLTVQDNLDLSSFGPVSKAFETLETVTEVSTCQCVHFIRVEADRDKLVG**AAGVMKAVL**TQEALQLTEMDHVVFPPTSTKGTLITRAKAKNMNFNKLYAEYIQRALTKQDAIQPDEVERDGTVMEAKLKLKESQPMRGFRIANHSMGVSLCKQFRCYQCKNRNKKQIKCRRECKDYELKLRITRRFFGKCGECAHVGYHYGSARDACVRCELNLSSSSSSFEDQGAAVGYIVSSPSSAPTLADLVTSGESGNSSSQYEPVDLSSLADNDISQSIDHLEEMGGMFDDDACLPSDLLPDELPSPPHYHSEMDVM |
| RRRRRTRINITY_DN128386_c0_g1::TRINITY_DN128386_c0_g1_i1::g.139519::m.139519 | NA | NA | 23.1 | VSRLPHLNGGLARQTGGLLNQTPTPYIEGVKLLSPLGTTLRGQSTNCWLSWPGVPPVQTKPTKATTRYLHQRGMIPTITLTPITTTT**TTPAQPIVA**ELPLKLRPCAQITGTGCTSRRITPAPTAGLQPGM |
| TRINITY_DN67053_c0_g1::TRINITY_DN67053_c0_g1_i1::g.64593::m.64593 | NA | NA | 18.6 | MLAGTHKTMYSKTLVVPLTGPHFCTKRLHQLHIQITKLPSTT**IARVCSCLLLT**HIIPMSLPCESSFLCTVIGVDGRYKWQQPVISDRQVGLFLFPLSSSLSS |
| RRRRRTRINITY_DN28317_c0_g2::TRINITY_DN28317_c0_g2_i1::g.5714::m.5714 | NA | NA | 8.9 | WCRQMVKYVDDPCRQPCAMRYGKEVQERAQQNTMGPYPCVGLSFTEWLLIGYSWVDSESSYRGYNLAEPATWKIPIQKLGSSSYVGDDEQRSMGFDSIKLVSGEGVLCNRAA**LDRLCVSPSPP** |
| TRINITY_DN59203_c0_g1::TRINITY_DN59203_c0_g1_i2::g.117000::m.117000 | gi\|657556149\|ref\|XP_008282392.1\| | PREDICTED: translational activator GCN1 [Stegastes partitus] | 19.7 | VPTVFSAPEKREGPVWKKMVEVQSLLVAEVVGGAKTIAHKSIVKNLNHLWEQHPGLVDQYISTLLSLDQSPTTLAMLGVCLDFCTAQKDKATIEKHKSALLDLYIKSVLMSKTKPQQHILDKSGSLLRHVSHSEFKELLLPTLQKTMLRSPENAMQTVSYMLSSVTLDLSQYAMDIGKAIASQLKANNAQLMEEAVQAMQNLAQQCSDPTAVQDIVTHLFKILGGSEGKLTVVAQKMSVLSGISSCSHHAVSGTSSQTLSSAVTVMFIPYLQQEVHEGTLVHAVSVLSQWSSRLTVEVPKALSDWLKKAFTLKTSTSLVRHAYLQAMVGAFKGDTLAQASDLVPLLLQTVEKAVAQNSQHALLAEGVAASVLLSRLALLDTQTEAKFTTFWNVILDEKKPLFTTEKFLSQASEETLLTELLLCERLFLDHAHRLNTSKSQMYHHATVAVLLSRSWRVRKRAQQTIKKLLSSLGGSSLAHGLLKELCVVINKHKVLPQDVLVSESGELTELGRSYVPPRVLLDALCVVCSSASQWSDPAEAENLAMEILIVTHHPSIVEARRGLWPVLLSSMNIKAEEFLEKNLEAVLPHLLEVKANNQAVKNAVGALSGLSPNKLLPRVISHVTEGLSQPALLQVTREEYAIMLTPDGELYDNSIIQSAQKESTKKVNMKRENKAYSYKEQIIELELQEELKKKKGIKEEVQLTSKQKEMLQIQLDKETSIRKRLQALDAELQSVVGLVEATLIEKPAQITRELPSVLQSLLPLLQSPLAAPRIQQVFLDIGVCLMPKPLHSLAVLVGHVTLRMLKPECDLDEAWELEDLDTAAERTVGLLHNHTVPQRESKAGDVAPLSAPAFSFCFPLLNAMLRESSDSTEETENMMTRALQVVNEHSQLRASTESDDIVIDENGPELLPRVTMLLLLTRIISTATPRLQVLASQCLTALCASAGGDMGCTVAEQPEIDVLLNALLSPCFSVRDAALRGLLEMEFALPTDSTEASGLSLLRRLWVARFDVEEEGRALAEKLWESLGLELVPELCTLLIGDVTHHEEAVRTAAAEALSTAVSEYRDQSPAVLGKLTELYHQKLYRPPPVLDALGRVISEAPPDQWEARCGIALALNKLSQYLDEAQVTPLFLFFVPDALNDRHTEVRRCMLDAALSALNTHGKDNVSSLLPVFEEFLKNAPQDASYDSVRQSVVILMGSLAKHLDKNDPKVK**PIVAKLIT**ALSTPSQQVQESVASCLPPLVPAIKEDAAGIVRNLLQLLLESDKYAERKGAAYGLAGLVKGLGILALKQQDIMTTLTDAIQDKKNFRRREGALFAFEMLCNMLGKLFEPYVVHVLPHLLLCFGDGNQYVREAADDCAKAVMRNLSAHGVKLVLPSLLVALEEESWRTKAGSVELLGAMAFCAPKQLSSCLPSIVPKLTEVLTDSHVKVQKAGQQALRQIGSVIRNPEILAITPILLDALTDPSRRTQTCLQTLLDTKFVHFIDAASLALIMPIVQRAFQDRSTDTRKMAAQIIGNMYSLTDQKDLSPYLPSVIPGLKTSLLDPVPEVRTVSAKALGAMVKGMGESCFDDLLPWLMETLASEQSSVDRSGAAQGLAEVMAGLGVEKLDKLMPDVVQTASKIDIASHVRDGYIMMFIYLPLTFGDKFTPYVGPIIPCILKALADENEYVRDTALRAGQRIISMYAETAIALLLPELEQGLFDDLWRIRFSSVQLLGDLLFHISGVTGKMTTETACEDDNFGTAASNKAIISSLGAERRNRVLSGLYMGRSDTQLVVRQASLHVWKIVVSNTPRTLREILPTLFTLLLGFLASTCPDKRTIAARTLGDLVRKLGEKILPEIIPILEEGLRSDKSDERQGVCIGLSEIMKSTSKDAVLVFSESLVPTVRKALCDPLEEVREAAAKTFEQLHATIGHQALDDILPTLLKQLDDEDTAEFALDGLKQVMAVKSRSVLPYLVPKLTAPPVNTRVLAFLSAVAGDALTRHLGVILPALLSSLKGKLGTEDEAQELCSCQTVILSVEDEVGQRIIIEDLLEATRGADPGLRQAAVTILNAYFARTRLDYSNHTRTLLSGLIRLLNDSNPDVLAQSWDTINSITKKLDASSQLALIDDLHRDIRSAAADVKGQHLPGFCLPKKGVTCILPVLREGVLTGSPEQKEEAAKALGGVIKLTSPEALRPSVVNITGPLIRILGDRFAWTVKTALLETLTLLLAKVGIALKPFLPQLQTTFLKALQDSSRAVRLRAAEALGQLVSIHTKVDPLFTEQLSAIRNAEDSGVRETMLQALRFVIQGAGSKVDPTIRKNITTTLLGMLGHDEDATRMASAGCVGELCAFLSEEELKSVLLQHVLADVSGVDWMVRHGRSLALAIAVKSAPEKLCGKDYYDTVTETVLTNATADRIPIATSGIRAMGYLMRHNLRTEGGGSISQRIITQLVKCLQNQSSDIRLVSERVLWWVFKESATPLMETSLIKPVLKSLLDNTKDKNTSVRAQSDYTIVNLLRLREGEETMQSFTAILDTASNDLLAECHRRSLKKIASLPDSNEEIDDTILT |
| RRRRRTRINITY_DN21715_c0_g1::TRINITY_DN21715_c0_g1_i1::g.51539::m.51539 | NA | NA | 20.1 | PKTWQIVIIDKTVHIAEPIGPPDCPDRAVCGESVKSCRGIGVINEAYVRYEYEISEELPHSKFTTDHILTKNIKVWLISNREKRELHYGLVPSGGDTVPEHWEVVQHDRSLSAIFPTGPPGPERYPYQAVVASSDLGHSKGYRNQAIIRFQYEAGTKLRHVKITTRACNITVNEWTATTTDRKQVIYNSIQCGGNHKPPDWSLTVSQTTVENFRVPGSPPGPKELVIVEVTTDKKGASNTVHISYMGGDDGAAEKISLTTHTRTNLINVRTTFKLPAGDKSWAVTPKPRGFVPIDINMDKGVHVSYSSFAPRVDPEFVLDKTMVPGALVKPDSKGKDNMATIRFLYEEGPNLNSVTAEMTKFTNVGSWKDQGKLQVELLYQIVRSGGDYEPKEWALSISTKTQDTVILNKPSQPVESVKLPDVITAPLGVGHGNEALVRFYYSCGEQLPDIKWSLAHCNTVVAAFTKRTSERKEVIYNKIKSGGDLLPPEWALTVSQNTIEKVKLNVPASPTDLVRVVVFASKTGSSNEATVTYKGSDNRSMKDIVLSTYSSTVEVQATEKLAAEDKDWKITPTPRGKIPIFLRLSAGAKITVIKRLEADIDLDPEEPRDQAVVAGSVDAHEGVGVKNIACVRFKYAQNEKLNPVEFKTKKVGTPPTCMIWEEAPAATAAADEAQAEAPAEVPEASMTTTCCEVIYGQIECGGDCMVKSWSLFVSSKTTDTVKPNLPPGPRFIPDLAKFFVTPSTPEGVGVANDAAVRFEYLHSELLGTVKFTLDTVTQRNCRTWRVGEKDHKEIIYGTIPSGGDESPAEWTLVMSDKKIHSVELSKPPGPTLYPDKIIVAASELPAGVGFQNVARVRFIYENGELLKTVKLCINEVKLDVVTWVLRSTERREVTYHSIKSGGDQIPPKWAISCRDKTVGTIILPGQPEGPKDLVVVNVQVAKEGGPNKLLLTYEGGDSLKAEKVSIYAKTPTNKIERHITNGLEQEGKMWHISPLPKGHVDADIKFNDGSNIVITQTYEPDISIRPPEIEDRATIPGTSYSPLSFVGAANRAIVRFEYQNDMVLGTAVYETEIINTFNAKQWRGEPLEKKEVIYGTVKAGGDYEPKTWIITVSDKSIKSAEPTGPPDCPDRAIHGESVKSMKGIGVINEAYVRYEYEMGPELGTTKFTQDTIISKNLKVWLISNREKRELHYGLIQSGGDNVPENWAVVMSDKTWAAISPTGPPGPLKYPYQAVICASVLVPGKGYRNEATIRFQYESGTKLRGAKIKTRALNPSVVMWTTTSTDRKEVIYNKITCGGDYEPRNWSITVSDSTVEEVKVPGGPQGPKDLVVIGINATTAGATNTLTILYQGVDDRTAEKISLNLMKDTAGFNVRTTRKLPQGDKVWSVAAKPRAVYPVEVTLDEGALVSFTNFLMKAVPAIVLDKVIVPVGIQRPDSAGKENKAAVRFMYEEGANLGSITAETEKVICSQSWKDVGKPQIEVVYCGIRSGGDHEPKEWSLTVSNKTVDKLTIKGPPQPKESVKISEGTEIPLGIGYENEALVRFYYNCGELLQSVTFTNHHCLANVTAYAKRTSERKEVIYNKIKVGGDNVPPDWTLTVAEKTVATIKLNQPASPTDLVRVQVTVTKSGSSNEVTLNYKGSDFRNVNEIVLLTYSTTTDITAREVPEGDGKTWKAQPTPRGRIPVFLRLSCCARLSVVKRLEADLDLEPPLLRDEAKTNDPVSVAEGIGENNVASINIKYEQNEK**LNIITFST**ALFGEKPTVITWEEEEEKELPICTEVIYGTIDSGGDYVPKNWAVTISSKTTDLIRPNCPAGPKYLGDTAKYFPSSVSPAGVGAANEAIVRFEYEHGVVLGTAKFQLDKVKRKNCKVWRLGIRDKREINYNTIPTGGDSKPAQWMLVMSDKTIATVEPNEPPDPVVYPNDAITPDSELSEGLGYKNVAMVRFIYENGKLL |
| TRINITY_DN42432_c0_g1::TRINITY_DN42432_c0_g1_i1::g.80733::m.80733 | NA | NA | 16 | SDGRSSPERLPELRRRLPPLSLSQTPRLRSRSYPPSRRVYRWTVFCWDGPAVPSPLGVRLRLLCSSFGKQIPLFWLNSRSLHEHFSPFLLYRFSTLYLLRNPLYSLCLQTVVFHRLSHRLPLFLFPYQRPFDSLLTDALPPFVPTPFPPLSLFFSSFSFAFSAG**LDGLSFPSE**ALSVFSTEFTSAVLSSCVFVGSCLTASASSGPGVLSPVLVASRFVSSSQLTLSFISSGFSVFSEGVLVSSGVVSVAAGLEASDSSSTFLTSEDSVLFLLSDVFSDFSTCEGWSAFLMTEEVLMICELSVLPPSFTFSVDLITDGDSVFLGLSDFSGGTSLTGGDFTPGFFSCAESSRCSTLVLDVSVSLASLSPASGFSFCIVLVSAA |
| RRRRRTRINITY_DN47500_c1_g1::TRINITY_DN47500_c1_g1_i1::g.36205::m.36205 | NA | NA | 21.8 | VGVYSFVDKILDNTRLPQQLVLGTQQHGMPQAVQGLHVQDQDHHLPLHRLHARVLLEHGCLSGERLEQPRVGGHVLSVIIFNWPASASSCLVAAYSSQSATRDDATLGLQGLGALLALVLVHLGLVRLDALQHLGVLGVHSPDLQRHEAGVQLG**DADKQGEDAQQ**QEQGM |
| TRINITY_DN96891_c0_g1::TRINITY_DN96891_c0_g1_i1::g.95900::m.95900 | gi\|734624149\|ref\|XP_010740109.1\| | PREDICTED: protocadherin-18-like isoform X2 [Larimichthys crocea] | 13.6 | GESTFDTSLIII**ISLGAICAVLMV**IMVVFAAHCNREKKENRHSYNCRVAESTHQHHPKKPSRQIHKGDITLVPTVNGTLPIRSHHRSPSATPPMDRAQMGSRQNHHSRQSLNSLVTISSNHIPESFALELAHATPPVEGQYQPRPRF |
| RRRRRTRINITY_DN88459_c0_g1::TRINITY_DN88459_c0_g1_i1::g.27092::m.27092 | NA | NA | 12.6 | ARALPSFFLQLPFQLFPPEQLFLLFSTRTSASSSSFPAVCSALESSARISDLLSSSSDPRCSSFFGPKTTSMSSRPLMMFLTLFALLLSSLRPSPWIT**FTDLIFPT**FFISSSSSLRSIIVGARLSASVFCPYLSM |
| TRINITY_DN43983_c1_g1::TRINITY_DN43983_c1_g1_i1::g.101609::m.101609 | gi\|657542915\|ref\|XP_008277143.1\| | PREDICTED: tetraspanin-18 [Stegastes partitus] | 20.5 | MGQGEASARGTTMEGDCLSCIKYLMFVFNFLIFLGGSFLLGVGVWVLVDPTGFREIVAANPLLFTGVYIILGMGGMLFLLGFLGCCGAIRENKCLLLFF**FMLILLIF**LAELAAAILAFIFREHLTREYFTKELKRHYQGYNNTDVFTSTWNAIMTTFDCCGVNSPEDFKDSLFRLINPNHMVPEACCQHASQAGELAHISQEQCLSGSMMFRNNKGCYSAVVDYFELYIYVAGALAIVVLTIELFAMVFAMCLFRGIQ |
| TRINITY_DN56039_c0_g2::TRINITY_DN56039_c0_g2_i2::g.121118::m.121118 | gi\|657581962\|ref\|XP_008295020.1\| | PREDICTED: vacuolar protein sorting-associated protein 4A [Stegastes partitus] | 9.9 | MTTSTLQKAIDLVTKATEEDKAKNYEEALRLYQHAVEYFLHAIKYEAHSDKAKESIRAKCMQYLDRAEKLKDYLKNKDKQGKKPVKEAQSNDKSDSDSEGENPEKKKLQEQLMGAIVMEKPNVRWNDVAGLEGAKEALKEAVILPIKFPHLFTGKRTPWRGILLFGPPGTGKSYLAKAVATEANNSTFFSVSSSDLMSKWLGESEKLVKNLFDLARQHKPSIIFIDEVDSLCGSRNENESEAARRIKTEFLVQMQGV**GNNNDGILVL**GATNIPWVLDAAIRRRFEKRIYIPLPEEPARAQMFRLHLGNTPHSLSEADLRQLARKTDGYSGADISIIVRDALMQPVRKVQSATHFKKVRGPSRSNNQVMVDDLLTPCSPGDPAAIEMTWMDVPSDKLLEPIVCMSDMLRSLSTTRPTVNTEDLLKVKKFTEDFGMEG |
| TRINITY_DN55015_c0_g1::TRINITY_DN55015_c0_g1_i1::g.60336::m.60336 | gi\|734647663\|ref\|XP_010753031.1\| | PREDICTED: asparagine--tRNA ligase, cytoplasmic [Larimichthys crocea] | 6.5 | M**AEITKGVEQVSVG**ELYVSDKCGNDQDGDGTEQKPFKTPLKALIFAGKEPFPTIYVESQKEGERWAVISKTQMKNAKKAFNREQMKSDVKDKKEAEDNERREKNLEEAKKITIEKDPSLPEPESVKINFLEPKRGQRVKVFGWVHRLRRQGKNLMFIVLRDGTGFLQCVLSDKLCQCYNALLLSTESTVALYGTVTPVPEGKQAPGGHELHCDFWELVGLAPAGGADNLLNEESDVDVQLNNRHMLIRGENVSKVLRVRSAVTQCFRDHFFSQGYCEITPPTLVQTQVEGGSTLFNLNYFGEQAYLTQSSQLYLETCIPALGDTFCISQSYRAEQSRTRRHLSEYTHVEAECPFISFDDLLNRLENLVCDVVDRVLKSPAGQLLYDINPNFKPPKRPFKRMNYTDAIEWLREHDIKKDDGTYYEFGEDIPEAPERLMTDSINETILLCRFPTEIKSFYMQRCPEDRRLTESVDVLMPNVGEIVGGSMRIWDAEELLEGYKREGIDPTPYYWYTDQRKYGTCPHGGYGLGLERFLTWLLNRHHIRDVCLYPRFIQRCRP |
| TRINITY_DN24643_c0_g1::TRINITY_DN24643_c0_g1_i1::g.51455::m.51455 | gi\|584022063\|ref\|XP_006805690.1\| | PREDICTED: DENN domain-containing protein 4B-like [Neolamprologus brichardi] | 23.5 | SSAVRVRLLWDTLTPDTDQWPPLYILWRIHSGVPMRSYGWRRHNHPFTLSFLEEVLRWVGMNEVHKAVTLL**LDTLAKQP**GSPRIQRSLYREFLFLTLAAMGKDHVAAFDKKYKAAYSRLSSSLGRDELRRKRVQPPSPKAIDCRRSFHPPLEC |
| RRRRRTRINITY_DN73312_c0_g1::TRINITY_DN73312_c0_g1_i1::g.64936::m.64936 | NA | NA | 8.9 | SDISHRLSDATSPRSGSRTPPRSNYRSPPLSGGKQKSLPYERDRNVEGGQSGPRYDATNIIRHSSPTVPRQLPED**VSPQPLLT**PKRKLKAMNTMIGLYETTLLSEGSLERPQPQLVEEKDLDTEMLDEDNDDEEEEDDSEPAEEEASNLQKIIFEIQRNLDPNDEKSYCRLIEEVSMERAEQRVSPICLISYLAGNVYPRVEHNEHGLLDTLVKLVHKANEACKRKGKTRLCLNMLLAASYELTYDTLCDSDQLEDVLWGILDDAIMATQQSRRLSLKQLAVLVNERALSDNEERRLAETLLKLLVPIQSLYV |
| TRINITY_DN101316_c0_g1::TRINITY_DN101316_c0_g1_i1::g.139505::m.139505 | gi\|1012671156\|gb\|JAR00360.1\| | Reverse transcriptase [Fundulus heteroclitus] | 15.8 | ASLQNLSLETLRSTWETDIGEAISPEQWGSALDGVHSSSICARHGVIQFKVIHRLHFSKVKLSKIFPDVDPTCNRCKRSPASLAHSFWQCSSLSTYWNSIFKTLS**DVFQLTIR**P |
| TRINITY_DN45220_c0_g1::TRINITY_DN45220_c0_g1_i1::g.73436::m.73436 | NA | NA | 12.4 | MLSVCVLYSMMVLGGRLRTLRRVSRMRAVVAWSLAVLFQMEMMSSWNRTDTTAPQISSPTMNCSPSIARIRFSQQ**RDARPFLS**RMIHFPPILLASSSHIGLIPSLKRWKSLWPVKSPGRIM |
| RRRRRTRINITY_DN56705_c0_g1::TRINITY_DN56705_c0_g1_i3::g.124552::m.124552 | NA | NA | 16.6 | TTIKAIVGQNVPAPKGCAKVCLEHTDFRNKNGGCGGYWFRTCAKSHADYHWKLVFKACTGEEKPLQCIDVATLAVPVPLMEVQEGQQVQQQVQQQPEPETEPKDLPTTNVTSIGQNVRKHEAAKTTVIGKRTSVTQGEATHASVVVHYTQASELNDVALETSSVNTKLVLTHDVLRTVTVEFYVFLKPDPSSWRLKLGSSTINFVHLEDADKQKRQHVAQHNEHHKQHNEQQQSSSFPNKLPQDSQFWKCNKSVEPSMYFANEISIYKPLLQSFTQLHKDYFQSLSDLRKFFVDSPESAMRSLVRSDGATLRNGVGMIVLFYGRCKVETAIRVLQEGEEEVGGTVFLILVKRDRPLPAQEFVLEVTSEIAAALARGGELQPTKDLLFRVIDQASHHETLGIDVHIPSGTQNHLYEYPAQQIVSVRAHHLSSTPNSSVHLHEVIQAIYRKIETFVTPYTSESSDVVFAMDIDVDTTFSRRDRTSLPPVYDCMQDPSCFDFCIHCNMVKQIVSNYQASGPSPLVIVQAMATNNIQLARTLAREERSVLFLTAIGADHLRLAANTVAQVQGLQEGVFFIAVKRVLFGSRVRKFTNQALFSMATELNRSKNTSLTQLGELRQVLARKRLADAFRVETVVESNYLTLAVRAGRPCNSEAITIDKVLRLATERMRSFAQRNVQKSADLAIALETPYLPCELKGYCCPCNDRIKKVLDCQVVGPGRPGKDGYPGAYGVEGKQGPTGINGSVGRQGRNGGPGPGGQTGPDGAAGKPGPFGPFGEDGKRGKPGVVGFGDRGDEGPEGRPGLPGVVGKPGPEGPEGKRGISGRRGKEGIPGSDGGPGRPGPNGDEGRPGPAGNPGTQGRSGGIGPEGPEGAGGPK**GANGPQGPPGRR**GNAGPRGLEGPGGKKGDQGPDGAPGADGAEGKPGARNNDQGSTGPDGRIGKEGSDGAQGKTGKPGRRGPNGRDGPPGAVGSKGEEGNIGDLGIEGFEGKEGSYGRSGKVGRQGPCGQFGQTGNVGPPGREGPGGEDGPHGQSGPLGPNGKSGPLGVAGRDGRNGICKCPVGCCQREAINDLEEVLEYDLDMINVRLPRTYRFGRGYELMLAEEFKNVRELGVLIFSNVGSQQLEVVRRKMEAYPADLGDTLHIVVKVADDQRTKFRNNYASITKGNLLFPGRNRLARFGEFLEDANNTFDLQAPESASDMALMGVQVSPIQGSSCSIPAMKSIRQLIAGMKSQLNTQSSFINQGSVDFGVLVEVNCTKPTDQVGVCLKGKVEDDLTELIQENLESLELFTSARAVTSSESAINELENGTNGVGVAFVRVGKSKLGLAATKSDDASRGGTVVMLIQPTGDDMRSGKERTFHVDQVHRIAAGINIKRGGKYETREIADIIEQKNKFTNLYFADTVDAAFHALSFQLNDRDNYFLDILNIIFSMVEKFNERGLNISGDVLFVIDAKKSPIGTDIDVTPPEVPITGHLRAVVKDQLASLGPFTPVQLVDDSTTAIQSLQRTDASGAGVGICSVRSSKLLPGYISVDQSSRGGTLVVLHQSAETPRVGASPRLENQMVYNIADALDSSRGGMQRLRKIASIVAPKNNHSNLSFETKVRDAYQVVAVRVQDPQVDLQQVLQLIFDRIHAIGGAGTNDSGDILFIIDKKGLHIPDIEVPTYSAIIDATSMTKVSDILQSEVRPLQQFNDVTYVLEPKLSIQKLEDPDANRSGIALPAVQGRKLQDAPGSVDDASKGGTVLILFQPVGDEIRSGASRQYINKSVWELARGTNLRNGGKSRIRMVAQRVEEKTSYENLLFELNPDDSYQVVAIRVKQMGVDLKDVVRRIMERISPFESRMATTGDILFVVDRKEGGTSTPTIVDPTPFVPRMNQIADDSLETITTILNDQVTHLSPLDDVTFVFNPVYSVVRLETPNVDRTGISLPIVRSQKLSSAPRSIDDTSSGSSISIVFQPVGEQKRSGRAATLVDQSVFQLAQGLNRQRGGRQRIARVSNLVGEKTTFSNLYMDAHASDGYQVVGIRIKNEGVNLSEVVRRIFERIVPFDRGVGDSGDVLFVIDKRPGVAEAKELVEILQQKPLVLINNLRSPITSLETFEKVNFAFSPNHSIDQIQKPDANKVGVGFPIVGDTKLAGAPEKVNDRSRSGALIITFQPVKLAARGGNRELFITDKMFRLAGGTNLSRGGAPSMTNVARLVEDKTQYTNFFFVPTPKESHQVVAVRVKDTGIDLPELIKIIFRRIHEFEGRVNDTGDILIAVDREAGSVVKEEPLAMDTDGVVTILKPMVVEAMAPINSFSVDHYALDPVFAVTRLLAEDAQGSSVVFTLVQNVALEDAIKKVEDQPPGGTILVLVQQVGQRRRSGTSSQFMTDLAHRMAAGTNLVSGGMLRLQSIRDVVDQRNNFQNLYFEINPYEAFQLLGIQVRDPRVDLQNVVNIMFDRVYPLNTSGVYNSGDVLFVIDRVVRQTQVTTIEVVPETPGETVIVGALTSLADVIARPNRNLARFDKLMFVVSDAFAIQKLEQENAAKSGVALTLVGMRHLSTAPTEVNDSSKKSTLVLLLQPVQQQIRSGKEPRLMNTRVFDLAAGINVSSGQRPKIAGLAATLAESSGHSNLDIELRPTNGFQAVGVQVQDPGIPMTDVFRKIFERVSNILTSGMTSDILFIIDRKGVTLVEQADLKPLLGAFLVGLLACLPLLRHRRM |
| TRINITY_DN51815_c0_g1::TRINITY_DN51815_c0_g1_i1::g.58500::m.58500 | gi\|734601409\|ref\|XP_010727607.1\| | PREDICTED: EGF, latrophilin and seven transmembrane domain-containing protein 1 [Larimichthys crocea] | 18 | LKVKSRLSASMESLLKSPMKLVLLTAWLSSLMDPCSLSDICAHPCHKLASCKTINGSNSACFCDHGYTGDGTTFCDDDDECRNVTNICGDRGTCTNTEGSYFCTCVSGYTSTGKDQFQPNDGTECHDIDECQKVDVCGPNSKCHNTIGSYNCTCQRDYVPSSGAKHFHPESGVRCKEHPQKYCHDNIGCITQTVNKTLEYMSNLTEPQSLLKEIAKQTSGELTSVEVIAYVEALCRSTSTLAAGEKDYTVKPSVINSTLSKLVKAVNNLVEKDELMAWSRIKEERREHTITKLLHAVEESALTLANNYKTPAELEIKASEMEMKVFTFDARHTKAKLSASMGGDRIDLTPKLRPEEDRNGSVSVVFVRYDSIGGILKPSSDPGVTDYSRYAETGEIIVNSQVIAAAVKPADVYQLDHVTFTLRHNEPIDTKADVTKCAFWEYEPDSLQGHWATHGCKTVHVTSNTTTCSCNHLTHFAILMSSGRANLVAHYTILTRITQLGMIISLICLSMCIFTFWFFSEIQSTRTTIHKNLCC**SLFMAEFI**FLVGINMKTHKLFCSVIAGLLHYFFLAAFAWMCIEGIHLYLIVVGVIYNKGFLHRNFYIFGYGSPAVVVAISATLGSRYYGTDKVCWLSTENHFIWSFIGPACLIILVNLLAFGVIIYKVYRHTAVKKPEISHYENIRSCARGALALLFVLGATWTFGVLHILHETTLTAYLFTIANTFQGMFIFIFLCVLSRKIQEEYYRLFKNVPCCFECLR |
| RRRRRTRINITY_DN62702_c0_g1::TRINITY_DN62702_c0_g1_i1::g.93291::m.93291 | NA | NA | 34.4 | Q**LTASASPIQ**PHQGGLWLGGLSLSALATPDLLSPLGGTSSLLQLLQEQNMFPFAEALPLPAPSDGVASHTPPNHDVLRSSLPGPSTAALPKTVTSVKPNVCGSQDAGTFHNEQSSMEVDTTRESASGEGQPLTSSAALLALVHNSLDLPKPSTSIAMSVNGQSHSLDVSTSIGNGSLEMAVTGEPAAEQ |
| RRRRRTRINITY_DN101993_c0_g1::TRINITY_DN101993_c0_g1_i1::g.134716::m.134716 | NA | NA | 14.8 | SSLSKKVKKATPLVT**EGVAAAPVASAPT**SVASATIEGGAVLHEMEAAASTKMFDIREEKTYAMKFRPDLLTAMDLLKETTDDDYKESLYKIIGEKITKTLPSDDEQSKLIEHKFIHLVPKVYSVSVYN |
| RRRRRTRINITY_DN99536_c0_g1::TRINITY_DN99536_c0_g1_i1::g.27938::m.27938 | NA | NA | 8.4 | FLVCVVAHLVVNFLHYSAATLESFLYNMRFTLVTLPRYSKHSREEAMPTGWFDNLFLNRLPTSPRLDKNDLIASVDDFVFGCSLSNWYCG**VVLGTLLLI**EKWSVVAM |
| RRRRRTRINITY_DN55782_c1_g2::TRINITY_DN55782_c1_g2_i2::g.20017::m.20017 | NA | NA | 16.4 | SPSSSSSSSSQPLFISRDYTGASPISTVRPPSEVRKRRMTPTVSGPRSKSDRLRLTSSDGAVPPHLQLQHREEVLRKILQDRESLREQLRGLQLDDCRPERRRCREERERLENELAVIRAQLHTQASSYESVLSQMEKKHERRIKEVEERRKDEEREKKKEEDEEEEKVDEEKREEWHKQSEELKEMEVRLKEVEEKLRQAEEELQADRKKRLEVTEEEKEETEVNKQISKEKLQKKLTANERQLELMACRSEDLQREFVQNQNQQKLDQQLAMMQTHAQLQAQLDEIQQMHRQTMQRSWETHQQTLEARQKDLELHHMTLLRNRESLVDEKAKKELTQLAMTHSHREEALSQSQPEQLLDRLKKNIEKTEQVEKRLRDIEPNGHCDDGQCAKKEEVEDLQGQLESMQTQMEQERNQWRQESEAVAANMEIRFEARLRDECEARIRDETHRQSQELLSRETNLRSQELVKEELRHKDARLSAIERQQRDWQKEADEQVRALRSRTQHLEEELRSCHPGCSAAAEPAAKDKKEQQQIASELRCIENTADKLKTRLEEREKELENRLEEGARREEELRTCVEQNTKKESSLAEQVSHLEAAHRRMVEEIAKRVQEKLRKRDEEAKEVLRKKEEELKQQVNAREAEVQEINKDLQSRVRVREVELEKRQRELEEEKERRLREKIEEVEKHLGEKEEERERRLTNRDKEFQDRERQLSEREKDIEKQLKDREDDAHKQIRRLEETQARVMESRLSSLAEAHQRIQRAHDSNRGSSQLELERNRSQLRSAQSRLEEVESCVCVECGGRNLLEEARSQLEDEQLLLREKAVALEEEARKAVGEAESVRRRDLELEEELDQIREQLKVTNRKSEALRELHSEVEAEARKVRERLASHMGKKEEEEREEWRQREERWEAEMKKRVDENEESLNNVKQEWQEREEKVAQRKREEARDREEKLAKVELLLAKVRELDKREETQKEEEDKKNDKDERQREEEKREKDEDAAKQLEDCKADLTQIQEKAKELEMSLRLSEEQTASNQARAEQLEEEAAQLREEFRKRLEVETEREREEALIRFCEFDAQVQAGVRQQEDLSVQLELLRTRLVTGEGEHCTDMLIRHLEEHHAQSLALLAAEQEENKMNLSYIVKTLQAIKKCMKSHMQYDYLEQEINPF**EAATGNVSSGPG**PPQAAKGAGSAMIFGGGGGGGGGW |
| RRRRRTRINITY_DN52665_c1_g2::TRINITY_DN52665_c1_g2_i1::g.110264::m.110264 | NA | NA | 12.3 | RGVPPHCECAQRIACLMIEVLATEQRDTLAEEGAMPEDLLLSIMTEWDKLLESACEWLSDVLYAGHEHLESELFFFVTTKILSGNLSQRGRRPLGEEEPGRHSFLKKFLFEGASVAIPRHASYVLHYVSECDEATLVEDSSQLVLTLLKVAQVAVDYEKDLTMSVIRDKFRSTFLELRTCLERNYFLGQLATLCKLRVEGQKDHMTWGVYKLYSDNLFADSYLKMWVGIEEICIARIEAIADRYRHVFVGKFIANMMNEIEDQNEQLEKRKQLLLELRDNARKPVAKNREAEYQRQTNDMNISLNLAVNVLATMLKMAALTSTHRFARVQSDSLGTLLSIVTDMMYEDYIISYQCQRVLVSIFECFSSKFKKWQPGAITLPYDGSDEDFEETMRRIIESNQMNRFMEGSVVGKCGSCQIFFNILDLLAVDRDHKYSEIWDDVVSQMASRGLKVVEFLMMNEMGNEQHHGNLRGAGKG |
| RRRRRTRINITY_DN42608_c0_g2::TRINITY_DN42608_c0_g2_i1::g.82791::m.82791 | NA | NA | 14.8 | KWNFTRGVLEFFKKNPEHFFQYLKQLIAPAVHPHARGKSDHLCRERGHDRLCYFGKTKNFYFNSANIQPELKLFREVKKMEPFPDRILEDGDVVHISELPFYQLWNQMHMYYLSRNLAKYGLNIEGDKVLISEIPQYRKHKQLRNYFVQTYDSLVRETPDRLILLLKIDPNMQHIRKPVKSATFYAPTKEVTLQDPYAYPMQSLYWPLGKTFHSEWDFFHVENQAAAVSSHL**SLMEILARTGG**KRVGIIIIQPLQQLTGNPHTTTGNDTPSSTPPSPPGEEVMPRSPIPPSQIAFLLLGLLLAAM |
| TRINITY_DN37519_c0_g1::TRINITY_DN37519_c0_g1_i1::g.127159::m.127159 | gi\|348527560\|ref\|XP_003451287.1\| | PREDICTED: metaxin-1-like [Oreochromis niloticus] | 13.7 | LFRGEGFTSGRKMAAPDELFCWEGDWGLPSVSTDCLVVLAYAQFAGAPLKLRKICNPWRSPSGALPALRTNQKETLSRVSDIIIHLRKQKYNADYDLSAKEGADSLAFISLMEEKLVPALIYTFWVEPKNYVEVTRRWFAEHMPFPLNLFLPGRMRGVQLEKLRLLRGDEGLEAGDELEKELYR**DAAECMNLL**SQRLGSHKFFFGDSPSSLDAYVFGHLAPVLRSKLPNMKLQQHLKSLENLNLFCSNILQLYFPRDGRESGG |
| RRRRRTRINITY_DN112831_c0_g1::TRINITY_DN112831_c0_g1_i1::g.140067::m.140067 | NA | NA | 22.2 | QKS**QTYCSILY**KEWDSDQKRMQLQVTSRKVLGINKLKNHKAHGNQALRLSLSAGRRMAGLKGKELFLEVTVKSGQPYLRAYLTRNHVMAKFWGVAEESWDRGNVPTVNALSVQVAQLPLAAMDPTLEKINLLSLCATGGHDLMRVEVKISTEREADTSNNDGSVGCIKTVQARCWQEHQPFWGMVQTGIDPICNQEAYSCSAKWRDTVLAQLKTHS |
| TRINITY_DN91867_c0_g1::TRINITY_DN91867_c0_g1_i1::g.95589::m.95589 | NA | NA | 8.9 | MSVRRLGCCFKSDLKNEWKTIFGLPHFPFETTFRLIEFSNSFNCLETSRATLKSQIFQFYVIV**DRRVGLSSF**FHFYTSSLSFVVIQIFIKLLKQTKSLPEI |
| TRINITY_DN23576_c0_g1::TRINITY_DN23576_c0_g1_i1::g.68493::m.68493 | NA | NA | 10 | MLPGDQTWLLGKRLKMCPPPDRDHRFPSLFPLWSGHESPEFLQKTPHWALEDRGSH**FRHTLAVYPLE**SGHLEHLGWENLCPSAQHIWGYACSRSRAHNSHSESPSDCHES |
| TRINITY_DN56777_c0_g3::TRINITY_DN56777_c0_g3_i3::g.108931::m.108931 | gi\|1012854798\|gb\|JAR72817.1\| | Nebulin [Fundulus heteroclitus] | 62.6 | MTTVVEEYVEYEEEEEEEGEEIVQESGLPTRKVRKKVKVDTSKFMTPYLAHSQKMLDQFSHNKYRHAYDMSRGQPPAITTESPEMIRIRKAQEQLSEVKYRMEGNKARTGSMYDGEAREIAHVKHVSELISKVLYRQKWDETKDRYLLPPDAPELVLAVKNAANYSKKLYTEAWDEEKTMCYPYSDSPELRRVAKAQEVLSDIIYKKGHHERKAKYTSLADPPEVELARKVDKQRSDLKYKEDYNKNVRGQWCETPYFDVATARVAMDNLSNRKYTQHHEDTKDQIYFMQTTTPVYDANKKARIAASEVQYKKEYEKSKAQCDYNTLPATENPLLRQLRYAGTILSDKVYKANYEKSRGSSINYCDTPKFQMDSVLKQFSDAHYKEKYDNEVKGHYIGSYEDVYMLHCQKVEEMKSEQAYKADYEDIKTRCFYPQTITPEYEVSKKLQQCNDKVYRQHPDKVKFTQVVDSPVQVQAAINAKQLSDLNYKAKYEEIKFKNTLPPDYPFFIQSRVNAFNLSDNCYKYDWEKDKDKKFEVKGDAISILAARAHTNIASDVKYKKEYEKNKGQMVGALSINDDPKIMHSVHVAKIQSSREYKKDYEKIKTKYHTPLDMLSVTTAKKSQAIASMAGYRSISNKFFLPYDSVLLDLAKKANIIQSDNEYKSDYNNLTKGTPWVPFGSIEVEKAKKAGEILSERKYRQHPDTVPFTPIADHPVMMQAKVNQLQRSDLFYKRGLEEVLQKYSLPPDAPEFLQARCNSYNVSEKNYKLAWQELIAKGYDLQPDAISVKAAKAARHAASDVQYKKAYEKDKGHHVGFR**SLQDDPLLVH**YMQVAKLQSDKNYKKDYHKAKLKYHSPVDMMSMTHAKHASAVQTYAGYKKIPHNYF**LLPDNLH**LQLCRNMNTNASDFNYKQDYENSVKGSGWIPIGSLDVERAKTAAAALDETKYRQHPDTIKFTSVTDSMNMELAKANAKIMNMKEYKASGEKFVHTYHLPPDVPELMQARYNAVNISQNYYTYAHRQDILKGHHVKEDAISIVAAKSSRDIASNYRYKLAYEKAKGHHVGFR**SLQDDPLLVH**YMEVAKLQSDKNYKKDYHKAKLKYHSPVDMMSMTHAKHASAVQTYAGYRKIPHNY**FLLPDNMH**LQLCRNMMEIQSDNHYKSDYDACVKGSGWVPIGSIDVELAKAAKAALDETNYRQHPSTLKFTSKSDSMNMALALANSKTLDHAAYKASGEKFVHTYNLPADCPEFLQAKFNAQTLSESHYKQKWLEDIAKGYDMKPDAISILHAKHGRHIASDVQYKKAYEKDKGHHVGFR**SLQDDPLLVH**YMQVAKLQSDKNYKKDYHKAKLKYHTPVDMMSVVQAKHASTVQTYAGYRKIPHNYF**LLPDNLH**LQQCRNMNTIASDCDYKSDWNNCVRGLGWVPMGSIGVETAKLGGDILSEHKYRTHPSNFKYSKLMDSMDLTLATANNKIMNKQAYTSAWDKDKLTVHIMPDAP |
| TRINITY_DN58414_c0_g1::TRINITY_DN58414_c0_g1_i3::g.45922::m.45922 | gi\|958219378\|gb\|JAO26969.1\| | ACON, partial [Poeciliopsis prolifica] | 14.8 | YNCVTSPPSSTSLSVNKMATYCLTVARLQLALGHGARRLHVSAAYRAK**AQVSMSRFEPTS**FVNYQKLQSNVDIVRKRLSRPLTLSEKIVYGHLDDPHNQDIDRGRTYLRLRPDRVAMQDATAQMAMLQFISSGLPKVAVPSTIHCDHLIEAQTGGVKDLARAKDINQEVYNFLASAGAKYGVGFWKPGSGIIHQIILENYAYPGVMLIGTDSHTPNGGGLGAICIGVGGADAVDVMAGIPWELKCPKVIGVKLTGSLSGWTSPKDVILKVAGILTVKGGTGAIVEYHGPGVDSISCTGMATICNMGAEIGATTSVFPYNHRMKTYLEKTGRGQIALLADAYSDLLVPDKGCEYDELIELNLDELKPHINGPFTPDLAHPVADVGAVAEKSGWPLEVKVGLIGSCTNSSYEDMGRAASLAKQALDKGLKCKAQFTVTPGSEQIRATIERDGYAKILSDVGGVVLANACGPCIGQWDRKDVKKGEKNTIVTSFNRNFTARNDANPATHAFVTSPEIVTAMALAGTLNFNPETDFLTAPNGDKFKLEPPTGDELPSRDFDPGQDTYQHPPTEGGSVKVDVSPSSNRLQLLEPFDKWNGKDLENMRVLIKVKGKCTTDHISAAGPWLKFRGHLDNISNNLLIGAVNIENDAVNKVKNQLTGEYGGVPDVARDYKAKGVNWVVVGDENYGEGSSREHAALEPRHLGGRAIIVKSFARIHETNLKKQGLLPLTFADPADYDKIRPDDKISITGLNSLGPGKPLTAVIQHSDGSQQSISLNHTFNETQIEWFQAGSALNRMKQLQH |
| RRRRRTRINITY_DN33412_c0_g1::TRINITY_DN33412_c0_g1_i3::g.51737::m.51737 | NA | NA | 13.8 | LHFTKALAKLEPAPLLDLAEELDGLDKETELFGNKVLEQAVPELDSSIEEYDLKNVQLWKLKRQFLRVYLKQGMVSLREFAHVPSMDDQNFLARDDENELVAQLVTRFNRLYYPLRPPHFGEVTQEGGTQQAGFEPLSDSNIVAD**SIESIEESSL**LPDGSSTSPIETERQEAVATEAVTKNKSSSLEVPERSEEFKAKKRFGATKGTSPTQKKRKALKSSTPRLTPADVRLLTEPKQTKQKLDRGKESNLLDAVAAPTEAETATLDIVTVSDTKNESSEILVNEKQSSSLTEPPDTEKEPLEPAHKGDTQTRECPKHYKRSLKSSLSGLDITRVVTKSHIERPAPPQQNNNKKFYPSTKTEKVDEEKRRDREPSKTPDKRPSLQISPVVSNSASAASSDGREFNQCQLDIHENIKFKPVLHGCLPCALKSPPQSSFFSSIPTVTAASASMGATVSGKKKSKSLSLSRRPKGTDDRQTM |
| RRRRRTRINITY_DN47660_c0_g1::TRINITY_DN47660_c0_g1_i1::g.12525::m.12525 | NA | NA | 37.1 | FVRARNCISNHSDLFLDAAFIWRGFMDTLVSLPIFSGPLTFRHAAATLVLASTFVGADTGFSLTEMVVLDAAGRTGAVVATLAALLLSVR**VHTLAVHAVP**QRRVPHGTLIHVLALLLIQASTLTLLTNVGRPLMLTVASM |
| TRINITY_DN76281_c0_g1::TRINITY_DN76281_c0_g1_i1::g.94487::m.94487 | gi\|808866658\|gb\|KKF17335.1\| | Cysteine-rich motor neuron 1 protein, partial [Larimichthys crocea] | 17.5 | WHDGCRDCYCHSGREMCVLISCPVPSCSHPLVRPDQCCPTCEDESGSGQPEGMDMVVCRAPGGE**FYVEGET**WNLDECTRCTCRKGRVLCDTEVCPPALCQTPIRNKDTCCHICQEETLSPLLPVNTSQQEYCITSDGDVLLAGDSWKAN |
| TRINITY_DN55676_c0_g1::TRINITY_DN55676_c0_g1_i1::g.19098::m.19098 | NA | NA | 28 | LLFCGSCVRPRIATAATNGSGCKLRCPGCLH**PSKPQFFSTDSVR**WRPKHAPRSPSTSSKPTCLQKSIQSKVSRSPARARSCSAGVFRRSRSYRLCHKHRN |
| RRRRRTRINITY_DN44675_c0_g2::TRINITY_DN44675_c0_g2_i1::g.10722::m.10722 | NA | NA | 18.3 | RQAAGPRLLPAAAGAVPSGPLLLLLLLFLRLVDGPPLGLVGHHLNSVGPHDAEGCSDDGEDQGLPLHVFVSQLLELDSLPHFIRVFAVI**FAAAVVAVCLPG**VHVLLPQPHSYHSA |
| RRRRRTRINITY_DN51685_c0_g1::TRINITY_DN51685_c0_g1_i1::g.38236::m.38236 | NA | NA | 17 | VQGDQIRVRADGRVGPLVGVHALSGCPHLRTFLTAAFAVALALSH**AGLFLLPL**GYGGGAVEQLGLLMLFLASDMGRRMIFVSSAVAGEGAQGVTGNGAMHGGGGMM |
| RRRRRTRINITY_DN30597_c0_g3::TRINITY_DN30597_c0_g3_i1::g.118032::m.118032 | NA | NA | 14 | SPISSPPRPAALQVLKMDAVTFFSSSVMTASCMLSSLRPSMSESLPIFSPASLFLPLS**ASLSSWPEAS**PQLSAPGREGRGGGEPRSGGGGGGASRSGGEEEEQEEEDEDDEFFVLNRGGGSLFVGSEMS |
| TRINITY_DN56105_c0_g1::TRINITY_DN56105_c0_g1_i2::g.121309::m.121309 | gi\|583989866\|ref\|XP_006790134.1\| | PREDICTED: PDZ and LIM domain protein 5-like [Neolamprologus brichardi] | 42.2 | RLQGGKDFNMPLTLSRLTDGGKATKAGMAVGDMVLSIDGIATDGMNHLEAQNKIKSCTDNLTLTLQKASSVPKPPPVAPKTAAHHQVTKVPRKHVVETDIHFYHVPTHADASRKRIMEDTEDWRPRTGTTQSRSFKILAQITGTENAQEEEAEKAKKTKVNKTTTRMVIGPKYNELRDSHHRVS**ARTLNVVQ** |
| TRINITY_DN46814_c0_g1::TRINITY_DN46814_c0_g1_i1::g.12144::m.12144 | gi\|734611888\|ref\|XP_010733370.1\| | PREDICTED: peroxisome proliferator-activated receptor gamma coactivator-related protein 1-like [Larimichthys crocea] | 11.1 | SRSESLDVDPRLHSTAHFESIQQESSAASPQRPASPSSSSSPTTEPKPPVS**LANQTHTAPLQ**EINKKFTEIASDVSRRPPALCPPSTRPEPASGCSRPPPQQHTTSPTTGTQLEPRALQSPGPDPARHVKCPSSTPRSAQTPPAPDAHVALKEEVAPRSISSPQEPPSPLQVGCGGQRAASDSGIEAPDLTSLLEQFEETQAKEERVDDNDSTAAPPSNVEVPGNCVGSEKTSGSPPTPSVGPVKPVGVPEARPQLQTSEPVDIPEPLATEIVLSTPARRKPPPSKAIQIIDPRPLPPKKTHIGLSEPPAAHSSPHMYSAVSCDHDYCAPVDQSLTRVKPSLPKDSPKPTNDLQATAVDSSAAAAPAARTTQSASQHQSEKPRTNLPADTDRALQFSGRSVAMGDAGAGEAETAPCSLPTPPPSPVRGRQKRRYRRRSPRSDSSSSCSSSSSSSSSSSSSSSSSSASRSPKRQKLHRRRSESSSCSSS |
| TRINITY_DN7657_c0_g1::TRINITY_DN7657_c0_g1_i1::g.52125::m.52125 | gi\|657793852\|ref\|XP_008323851.1\| | PREDICTED: tyrosine-protein phosphatase non-receptor type 11 isoform X1 [Cynoglossus semilaevis] | 11.3 | PEPEPKPSAAKPRKSYIATQGCLQNTVSDFWRMVFQENSRVIVMTTKEVERGKSKCVKYWPDVSALKEYGAMRVRNVRETAAHDYILRELKLSKVGQGNTERTVWQYHFRAWPDHGVPTDPGGVLDFLEEVNLKQESILEAGPIAVHCSAGI**GRTGTFIVIDILI**DVIREKGVDCDIDVPKTIQMVRSQRSGMVQTEAQYRFIYMAVQHYIETLQRRIEEEQ |
| TRINITY_DN30026_c0_g2::TRINITY_DN30026_c0_g2_i1::g.6026::m.6026 | NA | NA | 18 | PLSAHPLLPPTRQPFCSQCPFFCFVFPC**GVCPLSTYV**DASFDIYPTLVKPRQTFSSPITMAKVTFGTDERASVVNKRPGRRTLLQAISVQSINFQDVRQK |
| RRRRRTRINITY_DN44142_c1_g1::TRINITY_DN44142_c1_g1_i2::g.79405::m.79405 | NA | NA | 16.9 | HN**AAASTPRPLN**QETIPQVRFRRSQLEAASFEEWWCSCTIRILPNLSSSASSSGQSAGHSRFASPWHFRPLMLFPLQQHFHHGSSPDTGIRCCPLIVRIPLVLHDHKRQQHSARFFSLCHRGAM |
| TRINITY_DN50163_c0_g1::TRINITY_DN50163_c0_g1_i1::g.57559::m.57559 | NA | NA | 8.5 | PPFPSPLPQPWLWENE**ISATSDKDAEL**CNNDSRTNTRRWGADGRARSGRGEIRVRSHPTDHSALLCSPQLTHSHSSSPGTLLHHHTDRTRYPPRAKKAACVCVNCPPNTHTHTVAYISILAARLGWLSP |
| TRINITY_DN49475_c0_g2::TRINITY_DN49475_c0_g2_i1::g.76360::m.76360 | NA | NA | 9.8 | IRFRPWSK**LASADSKLLAT**TRMKPQRPWLWSGGIVRVSGMAVQRRCTVGQVPPGNSALDVCPWMCLAPLYSGPGSKAMSLLCGVNELTSLTVTGSSDVACRTNKTSITIRLG |
| TRINITY_DN50542_c0_g8::TRINITY_DN50542_c0_g8_i2::g.57896::m.57896 | NA | NA | 63.3 | RGEGRPAAAQRAHLAAHAAGAAAHAAERHRADDQRQLQDQLLTAGDVQVDHEGVVHLDDVTAHQRGLQSGDADHLPLPHVAHPRDDVVPSDRRQLLAQHLGAAVGHVAVLGDDGGLGHH**DPGAGVLEL**GVDVDVLALAEALPVDLSADGHLAVRDEQT |
| TRINITY_DN54679_c1_g2::TRINITY_DN54679_c1_g2_i7::g.18160::m.18160 | gi\|734624466\|ref\|XP_010740284.1\| | PREDICTED: transcription factor SOX-6-like [Larimichthys crocea] | 7.6 | MMSSMRATPPFHPPSEEEEAISQDNAAWAKEEREREGPKETASPSSHDQPHPDELQPVRDKLKDIEWDIVGPAAMDNESSKGCSVYSYRTNSTSPHKPEECSRDRGEPMIGLTFGTPERRKGSLADVVDTLKQKKLVELTKTEQDEPSCMERLLSKDWKEHVDRLNANELLGEVKGTPESLEEKERQLSTMIGQLINLREQLLAAHDEQKKMAASQLEKQRQQMELARQQQEQIARQQQQLLQQQQKINILQQQIQVQGHMPPLMIPVFPHDQRSLAAAAAAQQGFLFPPGMSYKPAGENYPMQFIPSTMAAAAASGLSPLQLQQLYAAQLASMQISPGAKMAPLPQAPNSSDPLSPSALKSEKR**ACSPVAQIK**EEGSTQPLNLSARPKTAEALHSPTSPTHSLFSGNKTSPSGMGKGRIPSPLPSLGRNTSLDILSSLNSTALFGDQDAVMKAIQEARSMREQIQREQLHQQQQSGHHSLEAKLSALSGMTLNNGNKERLHYETLSQHLGKLGEDAGKMVHRVIDLTRPEDLDGNNITEARVFREARGRNNNEPHIKRPMNAFMVWAKDERRKILQTFPDMHNSNISKILGSRWKSMTNQEKQPYYEEQARLSKIHLEKYPNYKYKPRPKRTCIIDGKKLRIGEYKQMMRSRRQEMRQFFVGPQPPIPLGSSPGGVGVYPGAITMATTATPSPHLTSDCSSNSASPEPATAVIQSTYGVKTEP |
| TRINITY_DN114298_c0_g1::TRINITY_DN114298_c0_g1_i1::g.141192::m.141192 | gi\|657800553\|ref\|XP_008327408.1\| | PREDICTED: periplakin [Cynoglossus semilaevis] | 18.2 | VIKYKTDPATERELERLRNEIVDKSHQTEKSEMEIRQLRDDIQRWKDTKPQVQTKEVVNEVLQYREDPKTKEEVEVLKRKLADEQKKRLDLERDRSSQEEKIRVRKIDLSQVREKIVQQEVVKMEEDPLLRSECDTF**SLNISTEQRNRD**GLKTELSQLQRQKADLDLQLEELERERRSRRDAELEIQRLRVRLSELEIRDKDNREKVTVKQKVVLQQDPQQEKEHSILRLQLDEEKHKRTLLEKELSALVQQQHALERMDVKERVVRTEKVQVERDPEAEIEIENLRRTLEEEKRR |
| RRRRRTRINITY_DN53829_c0_g1::TRINITY_DN53829_c0_g1_i1::g.16745::m.16745 | NA | NA | 16.1 | LNCAVGALACFVGEYTACFLAHAVVLGLEAHLEWDRPLHVQPRVFVARPFGAEEVPDVGAELPVFVVLRMVVEVVRRGLDDLSVLEVDEVWVQDVFAGGRLIEELGTGVVLVKTNQPEVGARGVLVDGELDHVPLPLVRVSVTEATLKGVPDELQPEVELSVAVPSLLGRELISWSGRQQTKLWVCHITKTTCDNKYQNT**GAVILILN**NCSWIQVTTQFIFQLATQFNCM |
| TRINITY_DN48163_c0_g1::TRINITY_DN48163_c0_g1_i1::g.13080::m.13080 | NA | NA | 20.8 | SILSDGTDRSAISSSNSSSSRSSSSLSSSSSSSSSSSPSFSAA**LSLAGFSLLAW**LCAFFLLLSLRCWTNFIRSAGSVMILKLNFSSSPSSMTSLSVSESSP |
| RRRRRTRINITY_DN53574_c0_g2::TRINITY_DN53574_c0_g2_i2::g.39036::m.39036 | NA | NA | 4.7 | LYIVFGPLKGVPGSHTGADHISSGINALLDPIQLSAAMQQHGCRASQHVHHLPALNSQVVAVVQDQILCITHQVHAEFRLDSADHFLVGHWMFANPLSHHETSGEGFLDLDQGPIEQLLINEQGHAPHAGGRLVDSLLHHPHLLAVLPGEEQVQEASCALVRQSQHKHLGLFTCVGQLV**EQVSLAIGA**GTDM |
| RRRRRTRINITY_DN42507_c0_g3::TRINITY_DN42507_c0_g3_i2::g.80811::m.80811 | NA | NA | 4.5 | GCTDLVAKQTSLNQRGDDHPSPFAQPSNDAQSSSIATNFPLLEAKFSLSNIIAVSFDDGSSSKNPEQVILQELMIVTCLVTYKLLRIAALQLDFRVDGKVEVDARCGTPVPSQKVLGAALRPVFDLAMLRQKLVGLLALEAGFHLVTFAGDWHLRTSNHGGPIADLMSLPQSG**HAATILDQT**PVDQAELPRQKGMEQM |
| TRINITY_DN52412_c0_g1::TRINITY_DN52412_c0_g1_i1::g.38550::m.38550 | gi\|734603309\|ref\|XP_010728656.1\| | PREDICTED: collagen alpha-3(V) chain [Larimichthys crocea] | 36.7 | MDHLVRTRSRRRISLFICILLHVTATQAADIIDVLRELEVSENMEGVSLEAGLCSSRRGTEEADLAYKIDKKIQLSAPTKQFFPDSKFPENFSLMATLRAKKGSQFFLLSVYDDQGVQQLGLEVGRSPVFLYEDQHGQPAPEMYPTFKKINLADGKWHRIAYSVQDKSVTLYLDCQKVETLDLLRGDDAVVSTEGVTVFGTRLLDEDVFEGDIQQLLILEDAQAAASYCVNYIPDCDSALPYSSHIPDLQENARLGSDALMQSDQPDEPEEAPKKDRKGKKNKKKRDKGSKGKRKGKGKGKKGSRKKKHQEEGLEDGFLRVSTKLPEYLSQEPFLTTELPEIKTTKETAIPAEVLDELLMEMPTHEPDSTTEASALVPSAVPESVVTEEEDKPVKKPVVEEYEDDLYKDLYEDLSVSTVTVGPNVTEYELVQYEDYKNDTEYEEYEAYTDGFD**FAERDRAET**WDGQGSVRGEKGQKGEPAIIEPDSLAAGPPGLPGPEGLTGPAGPTGPPGPRGDPGELGPPGRPGLAGVDGIPGPPGTLLMLPFQYGGDSQKGPVVSPQEAQAQAILQQAQLSMKGPPGPMGLTGRPGPLGIPGPSGLKGDSGVSGHSGPRGLPGPPGINGKPGKRGRAGVDGGRGAPGETGTKGDRGFDGLPGLPGNKGHRGDRGKPGPHGPVGESGEKGSDGPVGPRGQPGEPGTRGLVGPRGPPGPPGQPGIQGTDGAQGQKGNLGPAGEIGPPGQQGNPGVQGLPGPQGPIGLPGEKGPQGKQGMLGLPGIDGPPGHPGREGPPGEKGIQGLLGAQGPVGYPGTRGVKGADGVRGLKGSKGEKGEDGFPGFKGDMGIKGDRGDNGAPGGRGEDGPEGAKGQGGLIGDAGPLGIAGEKGKLGVPGLPGYPGRQGSKGSLGFPGMAGAPGEKGRRGPAGQQGPVGQRGPNGARGGRGVNGPGGKPGEKGSSGQDGPPGSPGEQGPGGAQGRHGEPGPRGPNGQPGKDGIPGHPGQRGEPGFQGKNGLPGPTGVVGPQGKSGETGPTGDRGHPGSPGPPGEHGLPGVAGKEGAKGDPGPPGASGKSGPAGLQGFRGSRGTPGAMGPAGLKGGEGLPGVAGAIGATGERGPSGSAGAIGQPGRPGGVGPAGPMGEKGEPGEKGPVGPAGHDGEMG |
| RRRRRTRINITY_DN58155_c0_g3::TRINITY_DN58155_c0_g3_i1::g.22324::m.22324 | NA | NA | 12.9 | KSLGEEEEKPVSSPLDPQSPTKDKKGRSLGWRDWRSRRDGESAKESHVSSASEISEKSAAAGTQKALQNEPIGMGKLGRGESQSHRHRTFLPTRKGETDEKREESQSRIKTSIEAVKNEFKHRMALVPSDAVKKSEKQVLEKQSGGVDKEKREESQTRIKASIGAVKSEFKRRMASVSSEDAKRQEREEPQKPTTTDLSKSRGIAWSVRKSAAPEQQKEEKGKEGKKDVSTSRKNRLSLRGIIGFGTEKDEGRLDQVSKSKEQLGKSVVELPAGRAGPRYVEESGEGAKRSLVPSKEESKNRTLKRLGRTLSSERKKFRAEFVEESDIDKIDTRLVPQRPAAPLDPNSSTSVSAPSRDATAVKAFVAPHQPLTPSDTGKTQAKDSSKVPGSAAQIVRSTKAAERKVDKENIHLQQHEKREEEEDDEEIVIIPTIVSPSRSESEVTDTQTKTRKEKDEHVKVNNQKQQQDVGPTPLVFSTRKTPSTSTSEMTTQETLASMSTAEQLKKGEMSESGVSPIELPAGFRRHLPMPESDGQTFSASKRFVKTKPTDEGPSDSSAARTMTPPGARSRRLNRDLSGTQREDEMGLTEFLPGRLGSMKKDMSGGRLLRQRMLELKLAYDEQSLKAAGPSPSRNPLEMSVAKKLSKSSTESAEAGKGEEPDIHLLLASDASSGRRMTARRAKTESEDAEVSSGKKLPVRKTKEAKEGSPTEEENESRKGAGEGEGTGAVETNKRTGDALVGPWGPGDEDGPIENLSVRSGSFVMSLPMPIFPLEDVDADSDSSSSLQPSGEKLHRAVAISVHSSSDN**LLEPISR**MIMKAKYSILSRQWRRRSLVQKLMATSISKNTPSKFWPHRLCEVASPRLRDVVLLKIVFGKAEKCLDAFMSEEFAVNYNRINLATARDNEGAFPSVGTLCLYTIVGVPWIDTATSVPTQNVIEPAVYEPTGYKCYYGESTELRVANGFDCIRIQDSGPSAMLINEPKIDLHAINKQHLYRLGELVQQISCRIELEMATTKKAMRELFEEHCLETILVVVDKKEFADHFYLVRENDLESLLEMERLALAKRKGRASTFKAAFEAKGKKEKVRKVYSFAGRGIEKHIDYYDTLRRMKRLITGEDEMPEPAEKRETHVTLEAKCSNSGALNKATCTYVGSHEPRANLVVLSYEPDDYVFTFHSSESLLVDDKYWLIDPDPKGEVVVALRCTESIHVDVDEMITEFKPPVALALQLTCLDSGFQNSATVVLQGIDTGRIRQLKLTHQDDDPMSMECTG |
| TRINITY_DN52552_c0_g1::TRINITY_DN52552_c0_g1_i2::g.58836::m.58836 | gi\|734633780\|ref\|XP_010745392.1\| | PREDICTED: signal transducer and activator of transcription 2 isoform X2 [Larimichthys crocea] | 15.2 | MAQWEKLCQLPTVYGQQLHELYDRDALPMDVRHYLSAWIEKQEWQRAARDHDLAVVLLQVLLENLDIQHSRFVQEESFLMQHNIRRYKQNFQRYLEEPCALASTILWFLEKENEILQSADLAEQVQFLQVQQETMETSSQQDLERKMAGLRNEVQCMEHTIICLEEQQDEFDFKYQTHKMEAVADEATKRDQVKVLQQLVNRLNDCRKRTLSDLTKLLDRAEDLIGTLVQKELVEWQRRQQKACIGAPDDVGLDQLENWFSCVAVCLFQVREFISKLEELVGKVSYDNDPVKAQRPALQKRADTLLKDLLKSSFVVETQPSMPQGKGPLVLRTNVQFSVKTRLLVKFPELNHSMKVNVSMEREIPQIKGYRRFNVLGTKSKALNMAESHNGGMVADFRHLTLKEQKSGGGGKGGSDIPLTVTEELHIIVFDTVFEMKGLSVELQASSLPVVIISNSSQQQSAWASILWFNMISLDTKDIMFFAN**SPAATWPQ**FGEMLSWQFLSATKRGLNDDQLEMIAHRVFGKHQNYDACKVAWSKFSKENTPDTFWVWFDGILVMVKTFLEDLWRDGLIMGFVSKGKEKSLLKKKQRGTFLLRFSESVIGGITFSWVEITATGEPEVKTVQPFTKVDLIQIPFQEIIRNYQILEAENIPENPLLYLYPNIPKDEAFGKYYSTKTGDDNPYIKYIKTKLMFVSKENTLEAKSPMSCDMTQGEGLEPFTGLCGEAAGESGSPCCPNQLL |
| TRINITY_DN46052_c0_g2::TRINITY_DN46052_c0_g2_i2::g.34840::m.34840 | gi\|657578826\|ref\|XP_008293302.1\| | PREDICTED: asparagine synthetase domain-containing protein 1 [Stegastes partitus] | 14.4 | MCGIFCLLSRSPAHFEWDKTVHEHLRRRGPNSSQDLTVTGGSPPRYQCLFSAHVLHLRGRLTPQPVQDDAGNVLVWNGEVFGGLPVTPAENDTAVVSEKLSSCSSPSQILSVLSSIRGPWGFVYYQKAGDYLWFGRDFFGRRSLLWKPEAEVLTLTSVAAHASDSDPSAWKEVPAVGVYRIDLKTMAETSNMMLEVFPWASAGNDASSACIEPPLESLPHGCTAVMNQSGLVLTSPVCPLNMSIPASLNETEMNPNSHSSVSDLEQLLASREKDDEVKCLIEVLSEAVRRRVQSLPFEDEDSSLSNNQARVAILFSGGIDSMILAALADRHIPAHLPIDLLNVAFKLQEPKKQQESAKKKHKNKAAIDSKTTEAGSRTSGPFDVPDRITGKAGLKELRHLSPERRWN**FVEINVT**QEELQRTRQGRICHLVHPLDTVLDDSIGCAVWFAARGTGFITEDNDQRPFTSPAKVILTGIGADEQLAGYSRHRVRYKMSGHQGLIQELAMELGRISSRNLGRDDRVIGDHGKEARFPFLDEDVVSYLNSLPVWEKADLSLPRGVGEKLLLRLTAKQLGLGQSAVLPKRAMQFGSRIAKMEDSHEKASDKCTRLLTG |
| TRINITY_DN54034_c0_g1::TRINITY_DN54034_c0_g1_i1::g.17270::m.17270 | gi\|734608814\|ref\|XP_010731681.1\| | PREDICTED: dipeptidyl peptidase 9 [Larimichthys crocea] | 8.5 | MHRVKRVKLCDTKEGIWKSCVTVSMTAVDGLSHSTE**VVEMEDVP**SQYFVEKHSWEGLRDIIHCSRKYSGMIANKAPHDFQFVQKKDENGPHSHRLYYLGMPYGSRENSLLYSEIPKKIRKEALLVLSWKQMLDHFQATPHQGAYSREEELLRERKRLGAFGITSYDYHAQTGLFLFQASNSLFYCQDGGNNGFIQSAPVKPVEIKTQCSGTRMDPKVCPGDPDFIAFINNNDLWITSIKTGEERRLTYCHKGVDNVKEDPKSAGVATFVIQEEFDRFTGYWWSPSAVKDPDGGKTVYLLYEEVDETEVEIIHVPSPALEERKADAYRYPRTGSKNPQATLKLAEIKTDHQGRIVSTQDKELVVPFTSLFPGTEYIARVGWTSDGKYGWAVLLDRSQRRLQLVLLPPALFIPVTDDPAQRQESVEAVPTNTQPYIIYEETTDVWINVHDIFYPFIQTTEDEFSFIWVNESKTGFSHLYKITSLLQPGCYHWTEDYQHIEGDFKCAIKEEITLTTGEWEVLARHGSKIWVNEATKSVYFQGTRDTPLEHHLYVVSYDSPGDVVRLTKPGFSHSCSVSQNFDFFVSHYSNVSTPPCVHVYRLTSSEGDPLHMIPEFWASMMESPGCPGDYSPPEIFDFPGKSGFQLYGMVYKPHNLQPGRKHPTVLFVYGGPQVQLVNNSFKGMKYLRLNTLASLGYAVVVIDGRGSCQRGLEFEGALKNKMGQVEIEDQVEGLQYVAEKFNFVDLSRVAIHGWSYGGFLSLMGLIQRPNVFKLAIAGAPVTVWMAYDTGYTERYMDVPENNQQGYEEGSVALHVDKLPSEPNRLLILHGFLDENVHFFHTNFLVSQIIRAGKPYQLQVYPNERHSIRCPESGEHYEIMLLHFLQQYL |
| RRRRRTRINITY_DN5241_c0_g1::TRINITY_DN5241_c0_g1_i1::g.77170::m.77170 | NA | NA | 14.5 | SLIKDRANQIVLQPFNEDVAEEPQPEASVGSVATASRSPCSAASLGGGGRKRPTDWKRRGPSMPGPNLRNECVNIHVPSERPINLLRSGDHILGSGSSLRTHLATYSVPTSHSLMCHSTQAPSFTSNAESVTSCVVAGASAITLVR**QRAMNALGI**RTRTRQCASIAPSNQLSKIQNLTFDLKKWLSTMVAPVEPCTEKAKAAVYENLITTLGKGFISLVCAPITGDATTHEAYEKLNPSEHIFAQSTATLGEEQLYGLVLRAVDSPLLM |
| TRINITY_DN48713_c0_g1::TRINITY_DN48713_c0_g1_i2::g.37266::m.37266 | gi\|734610615\|ref\|XP_010732676.1\| | PREDICTED: mediator of RNA polymerase II transcription subunit 8 isoform X2 [Larimichthys crocea] | 9.1 | PEVHLSRAIQSLKFFSLASISTMQQREEKQLEASVESLISRVAHVKNALHNFIYKLENEYERLTWPSVLDNFALLSGQLNTINKLLKNEKTPSFRNQVIIPLLLSPDRDEDLAKLTEQRVPVFSHEIVPDYLRTKPDPEVEEQEKLLSAEAARIGPEVAQKQIQTLNKLCSNLLEKLSNP**RDERDAET**AAMRQNKPSFNPADTNALVGAVAFGKGLSKCRPPGPVAQGPMMSGGPTLQQVTIGGGSSQQAGMGGPVPPQQQGQPGKMPSSIKTNIKSASNSMHPYNR |
| RRRRRTRINITY_DN49611_c0_g1::TRINITY_DN49611_c0_g1_i4::g.101150::m.101150 | NA | NA | 16.2 | LCGGFQRRS**SLPCGSLSDL**LLALMCRKQLCLWCLRPRPLPCLLPGHLRISYSPMSSPLISSPNLTKTKTKVYSIHRLQPLSLFVFSFAFLFVCFKRFISLFFVCIVTLDMKRGQVTGNFLIGPAIELVACPEPETRLLTAGCKALHCHFFFLYTHEKGARSDDHTNM |
| RRRRRTRINITY_DN46075_c0_g1::TRINITY_DN46075_c0_g1_i2::g.35014::m.35014 | NA | NA | 26.6 | **LQESGLTVLGG**AVGEAVAVETQSSSPVGVVVGTQVDVRQDWCAAWGVVLLCAVVWQGEGSVSVETGGGEWRYGAVEELSATEGQSAGQEWMQFGRKSGWLLSRPLSYFALPFPMNSCHWSWLTM |
| RRRRRTRINITY_DN26193_c0_g2::TRINITY_DN26193_c0_g2_i1::g.100351::m.100351 | NA | NA | 34.3 | SLCWFLDQTLAPSFSCAQCISCLWPSGC**PSLFLGASLCVC**VCVSLPLPLPSLRSTFFVSFFYTFVSTFPFFLPLSSCTRSLSSTQMSFVVSTYWHNLLVPIFLFP |
| RRRRRTRINITY_DN7208_c0_g1::TRINITY_DN7208_c0_g1_i1::g.96807::m.96807 | NA | NA | 16.9 | LSVTDSRLLLFSPSSLSFSLSLLSTLFSPSSKPLFSSLLSL**FLQPSSLFSHILSST**LFIFSASSLHCSFFCSLPHYFFFFILNFLSPHLLALLFVLHPRPPLFLFFIFPPSLFLPLSPSKPLRTFNFHSRTLIFSSSSILLFLPIPSFFSQHPLFSSPLSLSLQPFFSLFFSLFFSLFFSLFFS |
| TRINITY_DN58750_c0_g2::TRINITY_DN58750_c0_g2_i1::g.92144::m.92144 | gi\|808880847\|gb\|KKF28558.1\| | Kinase D-interacting substrate [Larimichthys crocea] | 14.5 | MDTTTSLKMTSLAVQSLFSYVEEENLAAIKAHLDKFKDVDSRSDNGQTPLMVAAEQGNLEIVQELIRRGANVNLDDVDCWTALISAAKEGHIEVVRELLENNANLEHRDMGGWTAVMWASYKGCTDVAQL**LLEKGANPN**ITGQYSVYPIIWAAGRGHAEIVHLLLQYGAKVNCSDKYGTTPLIWASRKGHYECVMHLLANGADVDQEGANSMTALIVGVKGGYTEVVKELLKRNPNVNMTDKDGNTALAIAAKDGHTEIVQDLLDAGTYVNIPDRSGETVLIGAVRGGHVEIVRALLNKYADIDVRGQDGKTALYWAVEKGNATMVRDILQCNPDTESCTKEGETPLIKATKMRNIEVVELLLDRGAKVSAVDKKGDTPLHIAIRGRSRKLAELLLRNPKDGRLLYRPNKAGETPYNIDCSHQKSILTQIFGAKHLSPSESDGDMLGYDLYSSALADILSEPTMQPPICVGLYAQWGSGKSFLLKKLEDEMKTFAGQQIEPLFQFSWLVVFLTLLLCGSVAVVLGFTVDPKLAIAVSLSLLALLYIFFVLVYFGSRRERESWNWAWVISTRLARQVGYLELLLKLMFVNPPELPEQTTRALPVRFLFTDYNRLSSVGGETSMAEMIATLSDACEREFGFMATRLFRVFKNDEVQGKKWKKTCCVPSFVLFSLTLGCLITGMALLAIFKVDGKNLTVNAVLIAMASVVGLALLLNCRTWWQVADSVLNSQRKRLHSAANNLHKLKSEGFMKVLKNEVELMSKMAKTIDGFTQNQTRMAVIIDGLDACEQDKVLQMLDTVRVLFSKGPFISIFASDPHIIIKAINQNLNSVLRDSNINGHDYMRNIVHLPVFLNSRGLSTAKKLCMAAPGNGEALPVEGWHEEMDRKMSQSSLGQDQAKFGSKNALNRRDTYRRRQMQRTITRQMSFDLTKLLVTEDWFSDISPQTMRRLLNIVSITGRLLRANQIIFNWDRLASWINLTEEWPYRTSWIILFLEETDGVSDQVTLKTIYERISKNIPTTKDVEPLLEIDGDIRSFEVFLSSRTPVLAARDVETFLPCTVNLDPKLREIIADVRAAREQMHLGGVTYPTLPMQEAVPRPQPGYGHHSAACSPTGSFTGSLPPQPHSSYFSGMTGPQHPFYNRPYFPHHVYHLPRHYPHHVPPSSRSSIKPSGHPQDPNGLDVIAEDARESLPSCPSEPTMVSPAVLLSSMNTDAVCERLKQMDGIDPNMLPQYTATIKKANINGRVLSQCNLDELKKEMEMNFGDWQLFRGMVMEQRHAESQALIQDESRAVSEQGSSVHHGEPSRRSAGAQRESGAFSLNLSFEELSGAGLEEPPRHSNNTHWPVANHRTSSMSSLNSQESSNDICKLTDKQQAEYRDAYREYIAQMAQVEMSGGGGERPVQPHPGQFLQAASSEDKAAKEGADQDGRKSFTKRGSKTSDATDFPSGTDAQTLDPISEEDEKLDHSSSSRTPGTRKKGAGAYYHKLPNDEDSGPEEADNTTPLLHREDEGGAPSIGLLTKPGFLNEILLDKKESSDSGMRSSDSSSDRSLEEAEGDDALKPSPIELELEGLVKKRGLLPSSLSGLQDATVARMSICSEAPSEASLMASSPDEGWPSSGVNNLNRTASNTTLNNNTSSPSDTTTNTNTNNSQQQQQQQQQQQ |
| TRINITY_DN53354_c0_g1::TRINITY_DN53354_c0_g1_i7::g.72043::m.72043 | gi\|657577699\|ref\|XP_008292691.1\| | PREDICTED: transcription factor E2-alpha-like isoform X1 [Stegastes partitus] | 34.6 | MAAVETDKELSDLLDFSAMFEPPVSNGKNRPTTLGSSQFGGSGIDDRSGSSPWGPGGHHSPSFNQGRGYGEEGLYSQHEGMASAPIFGSGIVGKAERGPFSSFAAQPGFMPSEIPMPSPDPLSPPGLKSNSQFYSSYEGSNPRRRPAQDPIEPQPKKIRKVPPGLPSSVYAHASGEDYNRDNAGYSASKAGNVYPPPFYMQEGLHPPSDPWGSAGSMAQPGFSSMLGNSPHLNQHGPFTAINPHDRLKRQPLPLSPQNYPLHGSEVNGAHHAGFHSGSSSFGVANHTPPIAGTDTIMANRGAVPGSSGDEIGKALASIYPSDPNSNAFPPSPSTPSGSPQAVSGSGSQWTRSSGQATPSPNFEGGIQSMSKMEDRLDEAINVLQRHASVQGGPGLAEIHSLLASGLGLPPGFSSAALGLASRLPGLMSSHHEDSAGLPSSGGILHSHHGPTSGSQPEGFSGLPGSLNRSSVTDIKQEGKEDDENCSITDKSEDDRKEMKIRPRTSLDDDDEDGDEGPVEFKAEREKVRRLANNARERLRVRDINEAFKELGHMVQLHMSNEKPQTKLIILQQ**AVNIILNLEQ**QVRERNLNPKAACLKRREEEKVSSMDPQMQLGGGHHGLGGDGHNPVSHM |
| TRINITY_DN63012_c0_g1::TRINITY_DN63012_c0_g1_i1::g.64465::m.64465 | NA | NA | 86.5 | MSFPAPTASLFRMRLIFKLFPNSRSAVSTPMWMKLLPVVLYGRAFIVADACLSGGIPGSAVFAVTVRL**APPATVLTVTF**AMPLGPVMPGTFPGCKRTSIPFAGV |
| TRINITY_DN21806_c0_g3::TRINITY_DN21806_c0_g3_i1::g.51629::m.51629 | gi\|1007788934\|ref\|XP_015830866.1\| | PREDICTED: small G protein signaling modulator 3 [Nothobranchius furzeri] | 20.8 | MSGTYTPAPGGPFSALTPSMWPQDILAKYHQKDSSEQPELLYDEFGFRVDLEDGEEPKSWLGTEGSPQREDPQQRLRWQAHLEFTHNHTVGDLTWELIDPVLSRSERLRSLVLGGIPHSMRPQLWMRLSGALQKKRTSEISYREIIKNSSNEESTTAKQIEKDLLRTMPTNACFCSLTSVGVPRLRRVLKGLAWLYPDIGYCQGTGMVVSCLLLFLEEEDVLWMMCALIEDLLPPSYFSSTLLGVQTDQRVLRQLIVQYLPALDRLLQEHDIELSLITLHWFLTSFAS**VVDIRLLL**RIWDLLFYQGSLVLFQITLGMLKIKEEELVTSENSASIFNTLSDLPSQLRDGPAVLGEAMRLAGTLSQDTLEAHRHKHLAYILNEQAQLNNGNNTALNTNLNKVVRRQSLRRKSTLSSLLFGEDEAEALKSKNIKQTELVAALREAIARTAEHFHCL |
| TRINITY_DN55488_c1_g1::TRINITY_DN55488_c1_g1_i2::g.119369::m.119369 | gi\|657581304\|ref\|XP_008294660.1\| | PREDICTED: glycerol-3-phosphate acyltransferase 1, mitochondrial [Stegastes partitus] | 12.4 | MEMSDGLLLQVNNGEQWCNRWKHPNDDSDRSTSPSVLRCVTSTWKEGLLNRKRPFVGRCCHSCTPQSWEKLFNPSIPSLGLRNVIYINETHTRQRGWLARRLSYVLFVMERDVNKDMFTRNVVDNVLNNSRVETAIEQVATDLDAAASQSGQEHKAVSKVKQKARAFLQEMVANISPAFIRMTGWVLLRLFNGFFWSIQIHKGQLEMVKKAATEQNVPMVFLPVHKSHIDYLLITLILFCHNIKAPHIAAGNNLSIPILSTLIRKLGGFFIRRRMEETGDGKKDVLY**RSLLHAYTEE**LLRQQQFLEVYLEGTRSRSGKPSTARAGMLSIVVDTLRTGSIPDVLVVPVGISYDRIIEGNYNSEQLGKPKKNESLWGIACGVFRMLRKNYGCVRVDFNQPFSLKEYLGTQRSRHIPPPESLEHTLMPTIISAQPDAQLFEGQEEQMNRELPEDIFRRQLINNLAKHVLFTANKSSAIMSTHIVACLLLYRHRQGVALSKLVEDFFNMKEEILSRDFDLGFSGNSEDVVMRALHLLGNCVNVTSSANRNGEFTIAPSQTVPALFELNFYSNGLFHVFISDAIIACSILSLQRELVVESESSNQSGGHSSLLLSQERLIRKAAGLSHFLINEVAVAPPCLTIYQVLHDAVTRLIQYGVLYVAEEDQEELSPSPTDEPWPKKFPEPLSWRSDEEDEDSDFGEEQRDRYLKVSVSAEHQEFFIFLQRLLSPVLEAYSGAAIFVHSLSQPMAESDYTGRLFRYLLTRTERGVAAYGESATHYLVKNTVRTFKELGVLKERRENKVTTLELSSTFLPQANRNKLLQYILGFTLL |
| TRINITY_DN74706_c0_g1::TRINITY_DN74706_c0_g1_i1::g.94312::m.94312 | gi\|808877379\|gb\|KKF25853.1\| | PiggyBac transposable element-derived protein 4 [Larimichthys crocea] | 20.3 | VLCQHTNKNAERRPLTAQWKPVDTEAIMKYMSIVIYLGLVKPSAMRDLWRKDRLHSHPFPSSVMAGYRFELIG**TFLHMSD**PAADLVNDQLRGQPGYDPLCRLKPLQDQILMACKAYYHPYQNLAIDERMVASKARHGMKQYMKDKPIKWGFKLFVLADGKTGYTCNFNVYQGKAHTPSGNGLSYDAVVNLLDVPFLGTGYNVYVDNFYTSTALFLHLHQIRYGACGTIRENRLGFLNALPKTAERGDMRWLREGPLLYVKWKDTRDVTVCSSLHKAYGGDTVQRRVRNHDGSWTRRTVPVPEPVREYNKYMGGVDLSDALIKYFSVTQKT |
| TRINITY_DN99833_c0_g1::TRINITY_DN99833_c0_g1_i1::g.96013::m.96013 | NA | NA | 41.7 | GANGCWLLSLAACSCSLANRCFSSRVRDILLGCEAADDAAADPFSFPLTPVLPKISSIWLTELGGLLVSSDWPPGFVMF**ILLCCSCM**TCSCSSLVLCSASMRLSMSRMTIMNELN |
| RRRRRTRINITY_DN87172_c0_g1::TRINITY_DN87172_c0_g1_i1::g.26848::m.26848 | NA | NA | 30.4 | CAVSGLLMRLGRQAARMLCTFSEAGSTRSAAKSAKPVAMFTTPSFATFGKKASKSLSLSSLIFSHESFTSSGPPLFPAPPPPPPAPSLFVAA**LAGGAASAGVLDE**AGGLFVDLAAELLGDPPGGLFPLGVEAPAPFDLFPASLLSTVSVSNDVSTHSSKAAFTSGAEQRCCSSEALQFSLSALMSSNWLATSVM |
| RRRRRTRINITY_DN57361_c0_g4::TRINITY_DN57361_c0_g4_i3::g.103141::m.103141 | NA | NA | 12.2 | SWETTNLRDSKVRGRCRYDGSHSSSAPSIRYEHQDPPKLSTPTEWEYTWETDGGQIECSVTITEGRYIEPWNPQLTVVARNPAINSIRISDSDESYYVPNGRGGRCRYLGGQSVSIRGSSLFVADQTTLTTYSQDGRYWSYTWDDSPNVSCTLTVSGGAPIERDDARLQAKPKSSVTKTIGESWQTSSYWDRRNRGRCKYKGSRVASTRWYNVDTQHTTSRPTTWEYWWETDGGGEIDCTLTITEGSFLQSWNPQSTLTVRNSFTIEITVESGHTYIVPDGRGGRCGYTGGQSVRIERSQDGDRRSLDEDSGSTRRRYWYYRWEAPKDVSCTLTVSDGVPITTEGALLTATPKDSVNLSVINSRKYQWHYAFCQYEGSYWTQLPLIFRKNSRYQFFGLNDKDIRYQWDTEKGEKVDCIFTVSEGTFFTSWNPELTLVVDEAHGSYLLSMLVL**ITSFFLGL**VCPSTQGM |
| RRRRRTRINITY_DN47536_c0_g1::TRINITY_DN47536_c0_g1_i5::g.68992::m.68992 | NA | NA | 26.7 | VKHGGASQVFGLALEPEFHPWWGGLTPPDQPQGVGVAVAGQLLWECPHSVVQSRWGGRPGAQEAQVTRCSWQQDAGGAAPTVSAFHDAGALPEAGSEFHGAQLQGQLMRLGTQELPAEATRLKSGVMAGGESEWGALSAVGARETSLGGAAALSCSWEEVPRLEAFVPEQSH**EETLMLLAALE**PEEM |
| TRINITY_DN87086_c0_g1::TRINITY_DN87086_c0_g1_i1::g.26817::m.26817 | NA | NA | 15.5 | MEVLRMLRESEEEFE**DDDSSDIDSVDS**VEEDAFEQGEDVLLDQINEDASPSCPCPVPGPSTALMQSPTIPRPPPATPPQSPSPNSQSSSPTVERPCKRAATVSTTRTPTKTPRRTPTKTPTRTPTRTPTRTPTRTPKRTPTKRPAVVPQHQDQNQ |
| TRINITY_DN57136_c0_g1::TRINITY_DN57136_c0_g1_i1::g.21581::m.21581 | gi\|543891010\|gb\|AGV76842.1\| | cytochrome c oxidase subunit 5B isoform 1 [Sparus aurata] | 22.1 | ETRVMAGRLLLRAGSTTLRLRNNVALRPTYRAM**ASEKGIPTD**EEQATGLERRVMVSFKEGKDPYSIKQPKFYSGTREDPQIVPCTLDKRLVGCLCEEDNTAIVWFWLHEGKPQRCPSCGTHYKLIHHDLPH |
| TRINITY_DN35285_c0_g1::TRINITY_DN35285_c0_g1_i1::g.54828::m.54828 | gi\|657577590\|ref\|XP_008292632.1\| | PREDICTED: coiled-coil domain-containing protein 22 [Stegastes partitus] | 8.9 | VNLAAQWEKHRAPLIDEHRRLKEICGNRDMESSRKLSEIKSLHDKIRVSTEEAR**KKEEMYK**QLVTELENLPQDVSRSAYTQRILEIVSNIKKQKEEITKILSDTKELQKEINSLTGKLDRTFAVTDELIFKDAKKDESVRKSYKYLAALHENCNQLIQTIEDTGTILREIRDLEEQIETENGNKTVANLERILDDYKAIRQENSALAAKVREG |
| TRINITY_DN3122_c0_g1::TRINITY_DN3122_c0_g1_i1::g.29254::m.29254 | NA | NA | 11.5 | QIICLHLLVCYEEDNYHFFYNLGFILIVLAAVSIDYNNTALHNGIVEIFVALSDLFHSQISTINLSTVSVVIDRNVGLNYVKYLKLNQSSKLLDPSRTVS**FSHVTDVH**RVLER |
| TRINITY_DN118457_c0_g1::TRINITY_DN118457_c0_g1_i1::g.130681::m.130681 | NA | NA | 22.6 | FELLEVEQQEEAPLGVDGVDQVPKGALGDRGDQVRVEDLVGGAVRVVSFHQRDPVVLLGDLDELKQVIHAIVISALCDSRALTAPKHQQQKYPCQLHGA**TPTCSAACLI**KTAKEK |
| TRINITY_DN42411_c0_g1::TRINITY_DN42411_c0_g1_i1::g.71640::m.71640 | gi\|657558081\|ref\|XP_008283134.1\| | PREDICTED: phosphatidylinositol 4-kinase alpha isoform X2 [Stegastes partitus] | 14.2 | MYEQLRDISIDNICRCLKAGLTMDQVIVEAFLASLSNRLYISQENDKDAHLIPDHTIRALGHIAVALRDTPKVMEHILQILQQKFCQPPSQLDVLIIDQLGCMVITGNQYIYQEVWNLFQQISVKASSVVYSATKDYRDHGYRHCSLAVINALANIAANLQGEQLVDELMVNLLELFVQLGLEGKRASERASDKGPALKASSSAGNLGVLIPVIAVLTRRLPPIKEAKPRLQKLFRDFWLYSVVMGFAVEGSGLWPEEWYEGVCEIATKSPLLTFPSGEPLRSELQYNSALKNDTVTPAELSELRATISNLLDPSPEGSALINKLDFAMSTYLLSVYRLEYMRMLRSNDSDSFQVMFRYFEDKAIQKDKSGMMQCIICVGDKVFDVFLQMMAEKPKTKEHEEELERHAQFLLINFNHTHKRIRRVADKYLSGLAETFPHLLWSGRVLKTMLDILQTLSLSLSADIHKDQPYYDIPDTPYRITVPDTYEARESIVKDFAARCGEILKEAMKWAPSVTKSHLQEYLNKHQNWVSGLSQHTGLAMATESILHFAGYNRQSTTLGTTQLTERPACVKKDYSNFMASLNLRNRYTGEVAGMIQFSEATHSQSDLNKLMVLQMTRALDRKDPEAFTQAMFKMAALLITTKNCDPQLLHHLCWSPLKMFTEHGMETAIACWEWLLAAHNGVEVPFMREMAGAWQMTVELKMGLFSEAVVETGPLAVSEESQPAPCAPDVIPHFLWIEFLVQRFEIAKYSSADQVEIFTTILQRSLSLSVGGPKSSLNRHVAAIGPRFRLLTLGLTRLHADVVTNATIRNVLREKIYSTAFDYFST**TPKFPTQT**DKR |
| RRRRRTRINITY_DN40086_c0_g4::TRINITY_DN40086_c0_g4_i1::g.125289::m.125289 | NA | NA | 18.7 | PQGSDRILFTGQ**LADSLIDHA**EEMAMPGWYYGSHQLQQYTCKVLEYEAPSSFPRLHTPWSDADAETVLQRWTEAEDRLNLKSWHLIDLQRETPEQSDAKVREPSQEPGGPEEPAESQSQTEATANQQRSQALKKELNDRVMRCHLIKKKVVQQVM |
| RRRRRTRINITY_DN44483_c0_g1::TRINITY_DN44483_c0_g1_i1::g.10550::m.10550 | NA | NA | 12.9 | KIQLPQIQLIVPMGSSSAGSDASSPKEVSADKAETSSDHTPNVPTAKTESSGATSATPVTQDSYKAEPELKSESSAETSWKLGVELGLVEELGVGLGSCGERIEQKMTVVEMNEELFEEIEELTPQFGGPPEETEVNSWNLYDINEEKVPGRKAPEDRWSDCLLTSLPM**SAASGAPVSNTGGLSAQ**SLLHDLISDQSEASSNSGYSSSLVQSDPSSCSRPSSLESLDDESLPSPMVQYSGLDTVMDSLRYAAPSGGYPLFTEESVPFNDVM |
| TRINITY_DN137400_c0_g1::TRINITY_DN137400_c0_g1_i1::g.137893::m.137893 | NA | NA | 14.3 | PPSSPFYNIYFFFLLNYPPSFLLSFPPFFFPLPFSPPPPLPPFFSFLPPFFLFSPPFPFPSLPSALGLCPRRFFLCLLFSSSGAGCNKSFARGLLARPARKSPLL**HSLPLSES**LLPSSS |
| TRINITY_DN46428_c0_g1::TRINITY_DN46428_c0_g1_i1::g.35479::m.35479 | gi\|542197803\|ref\|XP_005473406.1\| | PREDICTED: V-type proton ATPase 116 kDa subunit a isoform 2 [Oreochromis niloticus] | 21.5 | CISELGELGLVEFRDLNPNVNAFQRKHVNEIKKCEEMERILGYLLREIKKADISLPEGDVNPVAPLPKHVMTIMEQLQRLEVELGEVTRNKEKLQKNLLELTEYTHMLRITRNFVQRTSERETLQVQYEEFPFLEKDTMMDYNSMQRLGAKLGFISGLIQRVKIEAFERMLWRVCKGYTILSYAEVEEYLEDPDTGEPTKSVVFLISYWGEQIGQKVKKICDCYHCHLYPYPSSNEERNDVVEGLRTRIQDLHTVLHRTEDYLRQVLIKASESVYTWVIQVKKMKAIYYILNLCSFDVTNKCLIAEVWCPVNDIPNLRRALEDGSRKSGATVPSFVNRIPTNDTPPTLIRTNKFTSGFQNIVDAYGVGSYREVNPAPFTIITFPFLFAVMFGDLGHGLIMAVFAAWMVLCENNRKLKNTRNEIWNTFFEGRYIILMMGLFSIYTGLIYNDCFSKSLNIFGSGWSVKSMFKQKVWNNDVLRENRFLTLDPNVTGVFKGPYPLGIDPIWNLATNRLTFLNSYKMKMSVILGVIHMSFGVVLSTYNHLHFRKKYNLYLVFLPELLFLLCLFGYLVFMIFYKWLVFSAHNSRHAPSILIHFINMFLMQGDTIQPLYPGQTGLQVFLVVIAVLSVPVLLLGKPLYLYWLQSGRHRLGMYRGYERVRRNSEEELYLMRAHDMEEGSS**LSDLSSSGEH**QTEEFDFADEFLHQAIHTIEYCLGCISNTASYLRLWALSLAHAQLSEVLWTMVMRVGLRMDTTLGVLFLLPVFGLFAVLTVSILLVMEGLSAFLHALRLHWVEFQNKFYSGTGVKFCPFSFSLLPSSFEQDGLL |
| RRRRRTRINITY_DN56422_c0_g12::TRINITY_DN56422_c0_g12_i1::g.86927::m.86927 | NA | NA | 18.3 | QEAEKEVPKPLVDESKFRLAESTHSAIIAREIFPGMDAPRHSCVDILVTQLNSLIQEKPMDLTAIKTRVFCENEVMYEHGTKFLQLMKPINTGVSGRKSKPFKKRLKNKLPVLKPLMEPVTVYFDASVEDDLIQQVLEAGGVFAAGNDQAVKVQNADETFVLVKNMETQLPHPLHVTSVFAEVRRKKELKMNLKLDVYVNQDSPTFDLLQFSKLLDLADSVDYVTRPYYRMVYVDDVPAWATRGYPKHRNTDDIVRQEKKEEKEKEQLEAAVAPVGGIGRDAEKAAMQAKVLGNKECKSKAKRDAKDKSQQEKWNSLDIILKPEYYHNVESDRWILNDLYSPSSVAKYNCTHSCQLDCYKNEHHEDGDEDPTWSREPPFLSSYDFSPEDLFKERDGDAYKRPDVQVEEEDTVDSLARPDVQVEDPDHGSHLHWDAESLQSEHYRDWTHSHSQDMLLIDDVEDEIHFPHLSVPEDLPFSYDAMYPHALAEEATLRDMPNFTLIKELFDLA**QPSVDPLL**KALPTHPKSMDNRIFVPIVSHLEQRDEERLVPISELILQMQELEHAGAFLTKGTLMEAFICGAAWMDIAKTYNNPSLLLRPSRYWKTVLGESLHGKHSYHPDMIRALGFDGIKLVLDETNVFLNAPKLDRHLVNASHIYKLGRLLQYMFLRAHGESLLGRELLQCLDTEMYEQVIYVSNVETLSVVDETLRRGNPGLTEFVKVINDHDLRRIIKIERLAHKVSQPDTLIIKKVAVRKDCDTDVASFVLGNGGYGLPKLDMYRSGLDFGHINMLSEFKEAM |
| TRINITY_DN24534_c0_g2::TRINITY_DN24534_c0_g2_i1::g.54645::m.54645 | gi\|657561116\|ref\|XP_008284223.1\| | PREDICTED: chromodomain-helicase-DNA-binding protein 8 [Stegastes partitus] | 12.2 | QLEKDKRIHQKIKRFKTKHAQMRRIFQEDEEPFNPDYMEVDRILDVSHSVDKDNGEPVIYYLVKWCSLPYEDATWELKEDVDEGKVEEFGKIQNRQPRLKRAARPASGTWKKLEETREYKNGNTLREYQLEGVNWLLFNWYNRQNCILADEMGLGKTI**QSIALLSE**VYAAGIQGPFLVIAPLSTITNWEREFSTWTHMNAIVYHGSLASRQMIQQYEMYCKDDKEHLIPGAYKFDALITTFEMVLSDCPELREISWRCVIIDEAHRLKNRNCKLLDSLKMLDLEHKVLLTGTPLQNTVEELFSLLHFLEPAQFPSETEFLRDFGDLKTEEQVQKLQAILKPMMLRRLKEDVEKNLAPKQETIIEVELTDIQKKYYRAILERNFSFLSLGANSNSNVPNLLNTMMELRKCCNHPYLINGAEEKIVAELREVYDPLAPDFHLQALIRSAGKLVLLDKLLPRLKAGGHKVLIFSQMVRCLDILEDYLINKRYLYERIDGRVRGNLRQAAIDRFSKPDSDRFVFLLCTRAGGLGINLTAADTCVIFDSDWNPQNDLQAQARCHRIGQSKAVKVYRLITRNSYEREMLDKASLKLGLDRAVLQSMSGNKESNVNGQIQQFSKKEIEDLLRKGAYAAIMDENDEGSRFCEEDIDQILQRRATTITIESEGKGSTFSKASFVASENRNDIALDDPEFWEKWAKKADIDMDTINRKNTLVIDTPRVRKQTRQYSTLRGEGGDLSDLESDDEYPPANSRQSRSSRRSDRHSGGGYGRTDCFRVEKHLLVYGWGRWKDILSHARCKRRLSERDVETICRVILVFCLLHYRGDENIKSFIW |
| TRINITY_DN29977_c0_g1::TRINITY_DN29977_c0_g1_i1::g.70140::m.70140 | gi\|1012781246\|gb\|JAR49503.1\| | Thrombospondin-4, partial [Fundulus heteroclitus] | 28.2 | HLGALGTHVHQVQHRVVVKVVLTHVAHAVRVRVLLVGVDDESAVVVVVEDAVVVVVVVTVVAQAVVVRVQLGAVGDVGAVVSAVLETVAVGVLIGVTDVSHQVVVHVRLHGVTELRAAVTGVSHSVSVSVRLHRVRHLRTVVQDVGNTVPVYVLVAGVPLSVVVRVSLVA**VGDVGTVVTGVA**ERVAVRVLLVFVRDQPAVVLHVLDPVSVCVLVTLISDPVVVGVFLTRVWREHTVVLFAVLVVVQTGQRAVGVLPVVLLAVESQQHSAQ |
| RRRRRTRINITY_DN38203_c0_g1::TRINITY_DN38203_c0_g1_i1::g.8029::m.8029 | NA | NA | 8.7 | TLYFARKVKEDASLDPKSRERHGGCLSSSSSSSSSSCPAAVEERQPIRFGALNLLDRIMQGKIAVDLATNSHLSPSINVELIWPKLNEDLMIDFGFLEHCSYPTKVHMKLLSNVYPESGIITKIVIDKIKEWLLTTNVGKAGLYQWLAKLAWKHGQCAKDDSNTQYESNKKNVSYNTLHMFKNGLTKMSSSYKCSAFRVLGDTFIYIRLPDYSTVYVYIRLDFKNGSILYPKHLYKQVLLPRKRPMQSWKHIVQIGIGRASAPPKIIWKQRSGGDEWAKRLLKIDQPLVFTRPFFSFEQKGYRAQMKSLNRWLRDKRGIQFTGPFHNLKQHEDLSKFGPSKMHHGWCGLWDHSKKTAKFHSRGVTHKVVNPTVPSMKWKLLRRQEVPLLEVKENATSFYLTPSIFPFLSPVLAPREERDDEAGLSMPEEVGSLLTISATSSAGNISSCDSDDDDSCANELEDPLEEEGGDVQMVCESGSAQTEGVLREETTLPSHSSNSSSSSSMSAPQGQKTLHISSIQTTVSGVSTGAPPSSVDPIIDAPAPQQQQQQSASVSASLVTPVPGSPCLSVEPPGTRRCSSL**TSEAPYQSETAQKN**VDQSAMTPAKSSTTAALVAPPGGTVQLQNNLDSIITCSNQTQCCKTAAKASQCRKTSVERATSRIEPTTSDGGPADQGLTAGRHEGPTLCLPPVSPVSCCDAAELQPM |
| RRRRRTRINITY_DN124586_c0_g1::TRINITY_DN124586_c0_g1_i1::g.131171::m.131171 | NA | NA | 9.5 | ALPKVIKMSIYNPLSYLLIALFLIVITLLILVIFLCRFRRWVIFKMTKCPNTFWFFSTEPRKPPDLKPNMNPEDRGKGAPKEDVDAESVIELTMEVKGGLARQGDKEISCPWWGRVSQQAFLSKALEKKNPVGVELVDMMDLSCKDPNKAPAVLDRLDLEVTGLYDDLSFKDNDWIQVILKPPIRFESKDLSWFHEKKSVLCLQEAPLYDFEFVFRWNFNGDGDLSRYHVDTKQKDEEMGPMWGKVYIDSMNEG**TISTEDLTVD**TTNWVVARLFYKKAKRPTIDFPPGPVGISKPFVDVWMQLKGQSLNPQFSSYLTRTEVHEPVLGQKRLVHLCLRERAPGLHQHVEKNNEFQALTYDKGNFTLSNGNDETRPKSLGKLRALNELIQSPKLQDRWQNIGSICYTQPLGCYSNYRSLFRNELDIVTEGVKEDRSLLDYDCVSIKLDKDQPLFC |
| TRINITY_DN58526_c0_g2::TRINITY_DN58526_c0_g2_i1::g.62059::m.62059 | NA | NA | 9 | MKVSWWALVTEPITSSSPLSDALGEDELGLVSIVTVGSGSELPSDWLERGEEEGEQEEAEPP**SSEVGSVVLAG**SGLAMGKVWLLCSWLCSWLLFIMLFIALCISSSMRCFSRLGIRPKRTESLDIPTIRHFLKRHCWQRFLLILMMGQLSFLRHFLYWMFCWMLLLKKPLQPSQAWTP |
| RRRRRTRINITY_DN53957_c0_g1::TRINITY_DN53957_c0_g1_i1::g.79740::m.79740 | NA | NA | 3.9 | YRYHDPKFPGEAPLGLYKAQKDSLKTL**KVGLKDLHAA**AVQEDLKKPLFYVGLPYKSTNTWLEIQALVQNTFSNSMVFSPHGMACGLNVLRGEALIIIHRGSKMRYRDVQPKINIKEAANKTLWSMDIECDFHGINCVIADDKMNEFHHGLIIDECGTTTVFINGEKSAEDMTTVEYGEMAAQLANIPDIETVIVRAGFGRLAQVCGKGVDGYGAVVAVKGAIMVDTARKIGDILSERCGYLNDFKSKTVSDNVNIAPIKLDGKKMMKYLNHVGTTTEESVGRIGTLLKPYKQHVLNTLDGGDDLIMNLPQGDKFYITQEICWVYEEDTEGKWAYVPIGAKAIAAAAHDQTSFINCSSWQVEAGLAILTEILVATQLTMHLCGAIRAGKLPKSQGYMERMKMLGPMENEAIDIAKRGWEALSIDAVKFSLKDSM |
| TRINITY_DN120282_c0_g1::TRINITY_DN120282_c0_g1_i1::g.135001::m.135001 | NA | NA | 8.1 | SGSGCVRAVCGGLYSAAGMPHYVMPCQYTLHFRTVESLHQVCLQVMLPEHSQEVQSLLSFADHCCCVGVPCQVVSHMYTTRNLK**EETLSTQTPL**SAVGRGLLSSSSSPSPSPWLFIEHHVTSSL |
| RRRRRTRINITY_DN46169_c0_g1::TRINITY_DN46169_c0_g1_i1::g.77472::m.77472 | NA | NA | 25.5 | KLQLKHQSQGVWTFSNHVELSEPSTSKHRLDIIVDVHAMSCNSLTLTVPVLCLSDQVFPHTYTEPYHLNTKILQSLEASPVVVPLPVESKFKLLVMEQAEEKPTLSTAEKGITQLAVHLQGELILQKNDEVVYAKWIVIINLDLENCKKVINSIDSARDESSMSGQSKSNSTTRSSAVDVRQPESTRCCRFFFDGCPTSSSIIRENGLNLDAFTYKQVADSTAQHPKCRTARFCLKARERSTLKCDKDRSPNICKHLRWNQSSSSVQVIHFERVGSESTNINEVDIFVLTGGSGKDDLSHHPSVSSRSASAQVSLSRSACIFVTHRLVRHSIKLGKEISEYYFLFSIEHVGEQDPGRLWLPLQTSEGPQLTSGEIPIDMVSSLQSFDEASVLMESAVSGPQPMTALTKYASCSEASNPSLPTDPTTAQSGFTFFDPHTSAVRLGRLAVSSVNCFEVYAKRIEGCLLATPFQLFFVEMLPMSPTIIPDLRRDPGHRVVMKDEKTQNLRPGQIKLDQKGRVVMLSTTQQGDTGPATDGSSTEALNYVVGVINLQGTRHPLLRLRATKTEEPGMNFDLITETTVIDDKQAVGRALTEENTITEGELAEATKQNAPSAADATFRWLLSLNSLALPVKLPNRFSVEVIIPEEVVALPHRLNDTQSNLCCQTPQFTGPVTGRNAAAVLQEELHCWMTALDQDYEQDLSVHTAAQKEGEALRREHGFYVRTASSHICPLPLQPLDVENGGMVSKYVYLYERLFAAQQTATQRSDNTLIQRFAVVANEPQRLTFSQRGITFNIHDEALSWGREKYVQMAQCYCRLAHRRQGAKSFRHGALIMHFAFKRVMPNRMNIFCHAAQELLLASRLDSDESTMKILLTAADSYKAQSKLIEASLLACREALVMNKCVDRYTQIATDMYHAPYPRAAGAQLFASVAAMELAGAAYLMAQDSLFDKKATHYCSYALEYHQVLFCLDAMKRIQLEPAEPPYLLGCTSKPESISKEPGKGGGFWKKTATFLSRSLGKRSVLQDNLQRINKEIHPLLGRFTFEQIFQRIHDHDNLTLFAGHSGAGGATGEPDRTGKKMDTNVASITHLSGSGSESDLQLPHSELSNSVGDKETPHPLDVEAAGISNE**AAASNVTPDDLT**DQNQLNNRHLYQSWPDPIQEDEEPSSTRSNMKLLYCGQPGYRQKMDEYVADARQEDGESMDHLLVYYKLTNPIFWKPNTYEGSHQVRHQEQSLKLFQEVPVAETSSVVLLCALYHNLFEHESAPMTQLFNERYAEFWPTTASFNLDYDGATIVSSAA |
| RRRRRTRINITY_DN7173_c0_g2::TRINITY_DN7173_c0_g2_i1::g.1539::m.1539 | NA | NA | 22.1 | RDLWCISPYRDSLTKRLQASQSKDRTEVVCEVKAKFLESKESHRRHCIDLLRVDERSLVDLLRAMDGPWEGLQVSFKSVRDDLVLARDVCQRVLELEMNASSVVVAVRKGRLEPLQGALIAALGMAGETDVTSREFEQFRLMAVLSDEESVTIVKDVLKKALQFTNIGLSHEMLDGYLKKNPNSHIDKVPNDTKLSQLLLPFGEPEVGIILTQSNLHK**IAAATGALLG**YQGAAPVVVADLKHVQEYIEMGVTGLGALYEASEDEEVYLYGNEKALHRAHNQSDRGTSGYS |
| RRRRRTRINITY_DN58243_c0_g1::TRINITY_DN58243_c0_g1_i1::g.44626::m.44626 | NA | NA | 43.1 | LFCVPGIDVGFEQDPGGIDVPAIDVIPLRATKQTRYEFVTKGWNGTARSCSDEVVTYRFRSNGEAKLELDNSGKLVVAKKLNSNREDKYGVSNKCHYTITQSAEKSLLRIFTMQVTISNAPQDKNGYTFHTGRNIDAGFWVPKNRSSSWWVKRPISDPNASICTEGTDMNCHVKIADEASGQNPDVWFEGSKKMPYCRKLDDCTRAPHKRSGDPSKMSDIQSSLAKLTAQVSPDVPVITSSNSNPMAEDESFEPPPPPGSDYDQPGGGFLDDMAAIPPGPPGPPGPPGAEGPPGEPGIEGSLGRTGPPGMPGPQGVYGEKGHPGIPGPPGRPGGPGVIGPTGQEGTTGPPGPLGQLGTFGRHGKQGREGHDGLDGKDGRPGQPGVLGRKGASGPPGVPGRSGAEGRKGADGPIGVPGPTGPLGQPG**VLGEPGPDG**RAGRQGLVGDKGPPGDSGAPGDPGPDGRPGNAGPTGVPGPPGKDGSSGTTGQKGPPGAPGPLGPMGREGRQGPLGVIGRQGTTGAPGPPGDPGTPGDEGSDGKDGPSGPPGAPGREGQRGPSGHDGRLGLPGEKGPPGLPGPEGVPGTPGPPGVRGASGPFGTPGPAGQTGRGGKLGAVGVPGPPGHAGALGQPGPLGADGKQGQEGPEGKVGPQGDPGPPGSFGVAGIPGPEGRAGPVGRSGAPGAPGKPGPEGKEGSPGAPGLPGVPGPAGRAGDNGPTGESGKEGIAGRDGKPGPPGSIGREGPMGQLGPPGVDGLAGPPGPSGKPGDPGPAGPIGKPGQLGNAGLEGREGPIGREGRPGIQGVAGLEGPIGQDGPKGSEGPPGQNGPLGQFGNVGPPGQEGRDGASGSPGQSGAPGVEGDKGPPGRQGPVGASGQEGTKGPDGSFGKPGLVGMTGAPGRNGISGAPGPRGDEGPAGSPGPKGEAGQVGPTGTLGRAGPLGPEGTRGPDGLSGKPGGPGGTGRDGQAGKPGPLGDAGPFGRNGPAGREGVPGHPGVSGSDGRPGRKGEEGQPGIVGQSGPPGPEGKPGAEGKFGPVGVDGLQGKIGPQGTSGQPGPPGSPGPHGGAGQPGLHGVPGKSGPGGDTGGPGRLGVPGARGLHGTEGRQGQPGEPGRVGTPGPQGKMGPTGPYGPPGNIGLPGMPGHKGINGQSGRKGAIGGPGPRGREGPMGAPGMPGPGGMPGPPGTAGKSGLAGTEGKQGLPGGHGRHGKLGPPGPTGPFGRSGASGPFGVEGSNGSKGAEGDEGPKGPPGEPGRQGSSGMHGPDGVEGPPGQPGAHGPVGNPGPPGRTGSEGRSGSLMAQPGTKGDFGSAMQAELGGPHTPHGPPGAEGPQGPVGPEGDYGAPGRLGPRGKNGRQGRDGPSGPPGMPGPRGRIGTVLPVDGPEGKQGKYIRGGGGFTDTGGTSPSETQCIPCCEGEPIVMKECNSLEDCQIEDCLVQGNDCVCIRCPEPKWIDRNTYVQGDATCSMDDGSTGDQCTVILVQAVSVVLFLFTRLHVFSMM |
| TRINITY_DN57514_c0_g1::TRINITY_DN57514_c0_g1_i2::g.122339::m.122339 | gi\|554841313\|ref\|XP_005931074.1\| | PREDICTED: pre-rRNA processing protein FTSJ3 [Haplochromis burtoni] | 25.4 | MGKKLKVGKTRKDKFYHLAKETGYRSRSSFKLIQLNRKFMFLQKARALIDLCAAPGGWLQVASKFMPVSSLVIGVDLVPIKPIPNVVTLQEDITTEKCRQALRKELQTWKVDVVLNDGAPNVGANWQHDAFTQAHLTLMALKLACDFLTKGGTFVTKVFRSKDYQPLLWIFQQFFKKVQATKPQASRNESAEIFVICQGFLAPDKIDSKFFDSKYAFKEVEVQAKTVKELIPVKKPKAEGYTDGDLTLYHTFTATAFLKAENPVDFLSKASEITFDNADLESHPGTSLEIKECCRDIKVLGRKELRLLLNWRSKLRRFLAKKLRTEAKQLDREINLSSDENESHSEEEPEKKKEERKDGEEVEEGEEKMDTDEEEEMEKKLAELKAEEVADLRRKKKKLLKERRKQRERVELKMDLPGVSIADGNDTSMFSLATINKQKALADISKGDMKIADTLVDEDDDLHLSEDDEEDDEDADEMSLASDLDEDDMEEVEHRQKELEKKAPKKKVQFAEEEDDDDEEPEGGLLVELEGKDEKKERETNLWFSKGIFSEIDLEGDAENELQQTEWLQNKQTEKGKKRKAEEQEEEDIAQPEEEQAGPSQEAAEESDSDSDDSSD**DENEISRMKQAKGAAGK**SGEAGDDDFQVVPVESTSKKARILDAEGLALGCQIATSKKTARDLMDGSFHRFANSEEAWEVPEWFLDDERKHRKKPVPVTKEMVEEYKQKWKEIDARPIKRVAEAKARKKRRMMKKMEQAKKKAEAVVNTVDISEREKMAQLKSIYKKAGVGKEKREITYVVSKKGAGKKVRRPAGVKGVFKVVDGRMKKDMRGMQRKEQHAKGGKGRGGKGKGRPSKGGKGGMKGGKGRKGK |
| TRINITY_DN58026_c0_g1::TRINITY_DN58026_c0_g1_i1::g.90009::m.90009 | gi\|1041114050\|ref\|XP_017280573.1\| | PREDICTED: aryl hydrocarbon receptor nuclear translocator isoform X5 [Kryptolebias marmoratus] | 14.1 | MLFHTDMSSSNPELPDPNLGMGESGTQAGGRAVMPKGTNKRPAAPDFDDDDGSKLFRCDDDTGGSNNDKERFARENHSEIERRRRNKMTAYITELSDMVPTCSALARKPDKLTILRMAVSHMKSLRGSGNTNTDGSYKPSFLTDQELKHLILEAADGFLFVVSCETGRIVYVSDSLTPVLNQSQPEWLGSSLYDQLHPDDTEKLREQLSTAENNNTGRMLDLKTGTVKKESQQSSARMSMGARRSFICRMRCGTCPVEPLSMNRLNFLRNRNRNGLGPAKEGEPQYVVVHCTGYIKSWPPAGVSLTDDEADNTQGSRYCLVAIGRLQVTCCPSDTDINSISVPVEFISRHNCQGMFTFVDHRCMATVGYQPQDMLGKN**ILEFAHPE**DQGLLRDSFQQVMKLKGQVLSVMFRFRSKSREWIWMRTSSFTFQNPFSEEIEYIICTNVNVKQFQQQQQAELEGAGARDGLYEAGPITLSQMPVQPVQPVTAAGPDHSKSLEKPELYSSLFQGPDQTKAMPSTSTPSTQIYPPANNFTANRPNDAYRPVNMTPQMAQPTHSAGQMLARMSRPNGAPQSVTPSSTGSPLHGGPAGGWPGAGAGPQFNNNQQVAPQAPKTMSPSFAPMGGFGGSSSNSCGQMPTGAAPTPTSGANYPQMNARASLNTNGYNGNQSGAQFPSRAAEAVWPQWQGQQHSQSNAEQHPHAQGNQQDMFPDVLSMLDQPANFNSDDFEIPMYPSFNE |
| RRRRRTRINITY_DN75141_c0_g1::TRINITY_DN75141_c0_g1_i1::g.25850::m.25850 | NA | NA | 29.7 | LLVIFGSSFGAAVTRGSPSVALSRILGASLNNYALRFEHQLGPKRPDIFRLVSDASGFVVSCHPAPMTTVATIPNKGDVAEYFRIQKGTYQDWLHVTGDCSVVEQSAELQGVYFVSKRHDSYTLRPEVEQTGDGHNYLPWLKITKDKSGSLFYDEGALPA**LCKITGSHG**LFSQLRIQHFHFHPDQQNLGIEYQWYALWNGQLSRGSRPLEQKLASDEVTPGTVWSTSFSALA |
| RRRRRTRINITY_DN42009_c0_g1::TRINITY_DN42009_c0_g1_i1::g.80702::m.80702 | NA | NA | 17.6 | HPREGTHSRTHVNLHSKCSFGKGCHTCTYPLEGTHSRNHLLLEGSSLFSKG**CTTCLYN**KEGKHMQVHRTLGAKSFSKGCITCLNSPGQHLREHKQLSYKCLFGKNCQSCPFIRGRKHLRQHQYFGSPSTFGKDCLRCHYRFSHLKSHTKLQNPHEFVKDCTPCKTEEETFNKNVARTSLFRKDEGTREKRRRCKKNEVNDKKQKREQKKEVSEKQKDKKKEANLEKSKLQTKARPRKVCVKQIPASKQHERQIHEQVKRKQRHAFSCQPCIFLRSLPATEKKALWEDATVDSSQGDSDVGEVYKLAPLEQENDYVDDEESYAETFKGVTIQPPLALMSLLIDEESSSPATSLTGLQRHHLLLTTMAIPESLHQGFEESVSTTDTLSAAIQQLDPTPLLQELRSLLESVLQELKQDYASGYNAPFVEKFFSEKEFPVNLLSQVLNAFNTYSTKLIDSSAMQDASSVEISCTHIRDLHSQIAQLNAVGDDRCLELVLRARLGVVLQVRHLPSLLEPVIETVLSIFDVLKDYQMVNHQQITQWIFASMLRVPPVLLHLSSLPLPVGNSKWAM |
| TRINITY_DN46467_c0_g1::TRINITY_DN46467_c0_g1_i1::g.35360::m.35360 | gi\|657545506\|ref\|XP_008278071.1\| | PREDICTED: tyrosine-protein phosphatase non-receptor type 21 isoform X1 [Stegastes partitus] | 10.2 | MPLPFGLKLKRTRRY**TVSSKSCL**VTRIQQLNGEFVEFTLSVESTGQECLEAVAQRLELREITYFSLWYFNKQNQQRWIDLEKPLKKQLDKYGLEPTVYFGVVFYIPSVTQLQQEITRYQYYLQLKKDVLEGRISCSLDQVIRLASLAVQADFGDFNRYDSQEFLQKFVLFPIDWIQDERVLEEATQKVALLYQSFRGLSAPEAEMLYMQEVEKMEGYGQESYQAKDSTGTDVTLGSCLDGIFVKHKNGRPHILFRWDEINNMSHNRSFFALELANREESVQFQTEDMETSKYVCRMCLARLKFYKINKSSLEECDPLPSEGSQKSLLTLSFPRFPMLSRPSLPSNKGQAQPTVVNPVRRRSSTRISLPKPQAYMMPPPQMHYNGHFTEPYTSSQDNLYMNSQNGYYYHSQTSLDCSPLEYGSGGRLRNGSVYSAHSTSSLTNPQHYLQPSPMSSNPSITSDITRPDYVPSHRHSALIPPSYRATPDYETVMRQKTRGTGGGMVLSQEHRQSHSMRNLNIGNSYAYSRPDPLVYSQPEIRGEHGGAAQHHHYPFHLGSSFHSPSPYPYPTERRPVVGAVSVPELTNVQLQQAQEYPAPNIMRTQVYRPPPPYPYAHPRPANSTPDLSRHLYVSSSNPDLIITRRVHHSVQ |
| RRRRRTRINITY_DN44194_c0_g1::TRINITY_DN44194_c0_g1_i1::g.79464::m.79464 | NA | NA | 19 | LKESSPIWIYPGSQEEIILKIKNYIEELSDGQVIATFFEGFDQELKVAKDFVKNAQEYTQRKNMEMLAEISK**PKIFIAIP**YLQAQQLRKIANGSVDLICHKGREAVTRVSLISTGYLNENFQGAEIFKNDQIDKEMQERSGVFHYDQGDMENERRPRTTHPVCSGFKHPFESILDDNVRDKMPGLIIVPRTYHIEQRIVPEYSLITDEQGKSSSESDSTNSTLCQESEVLEQVINEKSKYFPFKRSLNFSKKRKVKVPRNSEIMGTRAHFKVTKLRAREKKEVRKKSPIVGIQESEGHPTVLRAQWWEDDSANIVHLIDGYSFSLGQSPLCSDRTRDYDFLARVYLSRKESTRLSGSGSSMSSNMMQE |
| TRINITY_DN59050_c5_g4::TRINITY_DN59050_c5_g4_i1::g.46567::m.46567 | NA | NA | 27.7 | TIFFYFKKINTLLPAEKHQKQSNSYTSKDFLFRGADPSSAELLLQLIQEILPKPPRLVAGAARPPAAGQQVSQQLDALHPRRQEVALHDAGGVRVPLLSGQLVQLHQDGGLLLQLEQQQLQSRQHGAHGLAARLLDVSAAVVAPPLCQVAQHGLRLLLLLLRLLVEERSHAR**QRDVVPLK**HTGQSLQSVRAVQQQAAHPAQRLLHGGRQVVSGPAGGRGAPVRLTQRQAAL |
| TRINITY_DN46549_c6_g2::TRINITY_DN46549_c6_g2_i1::g.96570::m.96570 | NA | NA | 27.2 | MAANSSTPILPSPSESPALRK**ALVSASVRALA**PAEKFCRNSFSSSSSMKPLLSWSMMAKAFLMSSADLAAMPTLAKNSLYLKESAAWQAARAAVMSASFRIEANAIFNSGLPLRLEWIRSDEPDESAHCSEALSDSHKLIYFHSPEI |
| TRINITY_DN44772_c1_g1::TRINITY_DN44772_c1_g1_i1::g.69897::m.69897 | gi\|348517094\|ref\|XP_003446070.1\| | PREDICTED: V-type proton ATPase subunit S1 [Oreochromis niloticus] | 11.5 | KMASARAAHLRGVSAGWAAVWAAVLGLFSVLSAGSGDEQVPLLLWTSEGISLPIQSLPTAGHIVGQQQLASYLEKVLNVGPRNVVLFLQDKMSIEDFTMYGGAFGNKQDSVFPNLEGALMSSSSPLVLPAVSWPASNAVIGQLQDQLDTSPLYMDPETLSQLRLNASSPALLVFRLPYGIGADLMSAKEILSGNDEVIGQVLSIMKTQSVPYTAIYTALRPS**REAASLSMEAGVGGG**RSLLQARGGYRERERERERQRRIKERAGIYAPVEFKEGEETCILLWAKSLSVSILRSGRWEEQDLTPLTFGEGVSPKLHGSSCDKTKAKLVLNYEDVLDHRSFKLIFAMSQRHYKVSARRWFTLDAVELEYDKIKATFNGSRHVYAPAEYSYRCESVTNFRWPVLIPRSSKDPANQWRVSFEDFQIQGFNVSGTEFSYASDCAGFFSPGIWMGLMTSLLMVLVLTYGLHMIMQLRTMDRFDD |
| TRINITY_DN17548_c0_g1::TRINITY_DN17548_c0_g1_i1::g.80166::m.80166 | gi\|734631583\|ref\|XP_010744183.1\| | PREDICTED: rho-related GTP-binding protein RhoN [Larimichthys crocea] | 30.9 | MESKGLLRCKIVVVGDTQCGKTALLHVFAKDSYPENYVPTVFENYTASFEIDKQRIELNMWDTSGSAYYDNVRPLAYPDSDAVLICFDISRPETLDSVLKKWQGETQEFCPNAKVVLVGCKLDMRTDLNVLRELSKHR**LIPVTHEQGTNL**AR |
| RRRRRTRINITY_DN44004_c0_g1::TRINITY_DN44004_c0_g1_i1::g.101614::m.101614 | NA | NA | 15.7 | VEMSNKFANNVIARLLLRDGIQSPGRFTFMDIARLTPHAQCLRSAAITAVNVMWAMIQGGFTNVQHNAHPPLVLEVSEVKTKEAAVANEVASTDAQQISGHSLLDRIIEDHVMRVRRREAAVSYEVHEKQTRPVIPKLIVKQGTHTRQTVFTACAHCVRWHRDSFLDECSVCIGVEMSTNFARNVKAKINVVQGVSITHEFHIDDMSATVCPSGAHREASLCATSDIWKLLQGVSLEQRHNSHCPMV**IQSMKLETP**NVSEEEEEEEEEEDKEVDGFVRSTMRSLQSRSVVIDLCTAPQRV |
| TRINITY_DN29815_c0_g1::TRINITY_DN29815_c0_g1_i5::g.5823::m.5823 | NA | NA | 38.7 | MVEGAESLGSNQTLRLLLCFPAVRALLRGPAVSAPPGPPGHQGPGSSLLLLPVQLLLLHLLLLLLLLQP**ATTTAAAAAAAAAAA**PGKSCSNTAPISKCVPVKSVRVGWFSASDYPASGG |
| TRINITY_DN10772_c0_g1::TRINITY_DN10772_c0_g1_i1::g.101234::m.101234 | NA | NA | 25.7 | VLLGTVAEPVAVGVMMVLVVLVLVAAVEDMLLVRAEVVGLVGVVLQLMRRLGPVLGVLLLLMVVLVLVLVDVLLLLLLVVLVVVELHFLRLLLRSLPFVRGRRQL |
| RRRRRTRINITY_DN58174_c0_g1::TRINITY_DN58174_c0_g1_i1::g.23087::m.23087 | NA | NA | 11.2 | TAVSTVIQVVEGNEGQIAGQIAEKVEMDTQLAPALEAAQVAATHDPVPALQVYQVQIDPPLSSGEVATAGGDASAVAILQAGHLVQGDQGVHVQHIQLNGESDQTIQVEEGQPVQAIHLQGQPIVQVQGDEGIRHFQVQQNALTYHLTQQTATEMHATEEGMGGETVSSRPTRKSFSKVALSQGRQKRRWATRCKSDISQKIRYWDSESIGFKHFLHCRIGYIMLPDLQKKGHKGQGS**LNSMAQVE**RHFLYDLLTLAMKEATRCNTSIHLMDAPSVPCRVRMEPNNEDGLWTGEPYDDLSSPLFPEEFVAVC |
| RRRRRTRINITY_DN43783_c0_g2::TRINITY_DN43783_c0_g2_i1::g.107257::m.107257 | NA | NA | 20.9 | YLCRTPRSSEAGCHGHREGTAPTAAATATATATTRPTSPWARTAMTVPAPRCVCAGGHCGPRNTCLLSRTSRRLSWRQQPTA**PASPCLLFLPPLSTQH**SSTIAVCNIVPHYTTEKKKKRHGSRWFGHLCCVLRM |
| TRINITY_DN57301_c3_g3::TRINITY_DN57301_c3_g3_i1::g.21922::m.21922 | gi\|584006915\|ref\|XP_006798435.1\| | PREDICTED: uncharacterized protein LOC102798648 isoform X1 [Neolamprologus brichardi] | 20.2 | MDKAKQLKWFLFTVLVAAPTILYITVLTPKSYTSVPTPHGPPKSCPFH**ISEETITPL**NNTKHFLVSAYMDQRVNGFDIRIISMFRKDSNQPLHCFFCCAGYTSR |
| TRINITY_DN55754_c7_g2::TRINITY_DN55754_c7_g2_i2::g.19983::m.19983 | gi\|734598865\|ref\|XP_010749703.1\| | PREDICTED: protein VPRBP [Larimichthys crocea] | 26.3 | EEEEDEEDQEDDDQEEDDDDDEDSDDDVDTDPLIAELENENGGEDEDDEEEDDGNDEFSPSDEEVARLLEEDVDVGDDDDEDDNDEDDSDNDDVDLEGDNDSSDNS**DLEDDIIL**SLNE |
| TRINITY_DN28943_c0_g1::TRINITY_DN28943_c0_g1_i1::g.56623::m.56623 | NA | NA | 16.5 | QSLIKVFSSLPGLCVFTRCHIYPGFQDCPLSRAIGEFNHSVRVLPCLVISPQQPEKHPSVVKTRGRCTFTIVF**DLSIKFLYD**FSVWVCLFFLSIDKRHQRAERHIYIISYCVELY |
| TRINITY_DN12118_c0_g2::TRINITY_DN12118_c0_g2_i1::g.2423::m.2423 | gi\|734635644\|ref\|XP_010746420.1\| | PREDICTED: semaphorin-6D-like [Larimichthys crocea] | 14.5 | RELDRDHHALFVAFSSCVIRVPLSRCSDYSTCKKSCLSSRDPYCIWLKSGSCATVSPGFKGGFEQDVENGYHQ**HPDTCHDVLA**TTRKQNTALDSAYGKTTPTSASTTYHGAAFPKEAGDPERGTGPDGPPPVGEQGSPDSEPISVEMEGVRRPPELDKPNHSVHYTLLIACVLVAFVLGAFLSGFLVSCYCNHTGHKTKKLSKDPEAPIPHALSLRSLAKLNGLLDSQGKDEKMEVSSPKIYNSFFANSKEHHPPRRNGHHGMTMGDLVHPHHHHHHLHHSDLQVYTHWKVSAWSGP |
| RRRRRTRINITY_DN23663_c0_g2::TRINITY_DN23663_c0_g2_i1::g.121537::m.121537 | NA | NA | 10.5 | VDPAHGHGEHLKPKHSCTPKREHDRQSKQRWDHFIWSSYLRKWGTLPIFCVPTQSFRKQSVYKTNKTLGSARVQQVRAQLLDGLTEVLNTFTPRDAPSAEWCALMTSYIEPTSYEPARMRTG**AKLRHCF**EEDIHLGPYPSAGLSFIEWLLIGYSWVDSQTTFVKDFISEPSMWKLPLRADGKRVYDPDKYIDRALGFDCIKVVKNDSLLVNRAALDRHICKRSALYEMGRAVQFSFSIL |
| RRRRRTRINITY_DN118723_c0_g1::TRINITY_DN118723_c0_g1_i1::g.138488::m.138488 | NA | NA | 16 | HSTHVSWGRHLDGVFVHPEEDGVVGGVGGQVLEDLLQLFISDGGYHSHALGGVVGGQHHLLSDLNQIPREVVSSRGGVLDHVIPQVQQLQLEPLLGAAADSDVTHGSQPSSRNQDCEGQDFTP**HFIFISDL**ENSLLTSSREVALLQEVQLFITWLHAQAEFIG |
| TRINITY_DN68349_c0_g1::TRINITY_DN68349_c0_g1_i1::g.104526::m.104526 | gi\|657555180\|ref\|XP_008282049.1\| | PREDICTED: histone-lysine N-methyltransferase 2B-like isoform X2 [Stegastes partitus] | 15.8 | SRGRHLRCDRCGQSGATVGCCLATCQSNFHFMCARAQHCVFQQDRKMYCYKHRDLVSEKMVSGKGFEVSRRVY**VDFEGINFR**RKFLTGLEPETINMTIGSLQIQRLGVLSELSSNGRMLYPVGYQCSRLYWST |
| RRRRRTRINITY_DN53650_c0_g7::TRINITY_DN53650_c0_g7_i1::g.39619::m.39619 | NA | NA | 19.6 | AALFADSSWISRSQLLRLSTYLPNSGSQEAASVCVCVCVCVCVCVCGFQQLLTSTLNSIFRKFSHNSKRHKISSIDCASYFRI**IMIIITILA**PTNHTHSSSLVSARM |
| TRINITY_DN51639_c0_g1::TRINITY_DN51639_c0_g1_i1::g.38075::m.38075 | gi\|734621434\|ref\|XP_010738618.1\| | PREDICTED: alpha-2-macroglobulin-like isoform X1 [Larimichthys crocea] | 75.7 | PVALQVVMEMKSVTTVVLTEVVKEDFYRCVTFQVPAVLSRTVANIKVTVQGENGAMTKTSKVAIEPPSFMHIIQTDKPIYKPGQTVQFRIVSIDTNFIPVARVYKIVELQDPNSNRIAQWLDKSIDGGILDLSHPIIPEAPQGTYQISATTDKGEQIGYSFDVKEYVLPKYEVKVHLPSVISILDKEATMQICGKYTYGKPVIGSVKAEFCRLPARFYWYSRQDMICKTYQLTTDKSGCATQIVNVTEFSPDKTMYDDNFDVTAEIEEFGTGVVLKGSAQTSFSSSIRVVTFEDVPAAYKPGIPFEGKVNVVGPGNKPVADELVYLFVGDAQNETLRTDKSGMASFSLDTSLWKSSELLRASTKKTEDFETFERNVRRPSYRSAHHHVTAFYSKSSSFLKLMQAGEISCDKDATVRAQYIIAGEELKTGQEVLDFFYLVMSRGGIVQHGRIPVTVKTGTVNTGELSVTLRQLTGLAPFAQVVVYTLMPSGEAVADSQDFPVQLCLSNKVSLKFSSVQSLPAEKTTLSLQASPRSLCSLRAIDQSVLLLQTEPEISTKQVYSDLPLQRLTGYSYDVEDSEPYPCYPVLVPVPVV**EPEPEPEPEPEPEP**KPEQCSWIITKNNKHINTVSIDLLMSVILLNLIIN |
| RRRRRTRINITY_DN65456_c0_g1::TRINITY_DN65456_c0_g1_i1::g.112674::m.112674 | NA | NA | 39.8 | GLRHGTTRHAIRIESLRVVIEDLEALFDPDHRRSRLQLYKKKRKRKQRKLKDYGPCGHSRRKCKHKHKEKHGLLSSAPAAPHPRRPDPSSRAGHPSPSPSPHEMLMASHRRRGRSNLGVGPG**MASGIVGPM**LLGSGMGPPMGIGGLGGAGGAKEGRDSTSNNDTGIGSDSPVTEESHSESVPSPTAEKLESLPAPSTPHLPRPPSLKHYHYHQRYHHHHHHYGRCSKKQSAAAAMSSGLGPNRPNPASFTP |
| RRRRRTRINITY_DN45755_c0_g1::TRINITY_DN45755_c0_g1_i1::g.104132::m.104132 | NA | NA | 11.8 | KMNNGIWDKFNAVHTYVTYLDPDCEGQDYGFSVLGLLRWLGKSERQGQAFGLSPNQNSTARNDTTASLVLIGGTDSPCINSSGYDPKQSACLMNDTVSVPVGNRAYQQECPVIDALQVVGVRAREDGGLSSDPLPSWGTVLGQGSTVEEAQSESLCLPLVKEGVRAKDLLKVVAMDSDLINPDYNPHVAISAVRLHQLGKSQRRDDRYHKGVVVKIKAADLPYVKGLETVCHAAVVVSRQNVLAGSCVLQWDSAEDHNGTGARGDKKLSGALSDAKTVKTEIGNTSRRYIAALWPWHAEEPREPDFTPHKGCVPSCSVHRGSWKGTKLCTRRSSGSHQYFQSACEYEIHTYLHHFGPSLQPLVPPGKTPGDSQLQRGLMSSYFKHVPTKRSPTQPPMVRQKVLESVKPERCARICLPQKGSWTGDPQCSRRTDGSLAYSHNCFFEVTQPELGPVVQHYGHYLRPLPPCSKESFSQNVEKEKEKDKEEKERELEEETKEAPDKETKDEKITAKEKPKGDDEGG**GRYLIYQTIN**VRTPIIKRGPEPSGITNNEKKTDKTDYTVTNNKTMELEFIEVTNEGESIVTRLKTDNPGVKDPDRKGNVQGEKRGQEEQGKDKIVVIETIDTKGTGVNIDPSDEKKEPPTVAEPEKKEVTNLSNDTDKSDVKDKTSGGPKESPTNIEEENRDEAVPKEPITGTYKDERDVTTTNEHETNIDKGKPSEKEGATNEQSKEKPTNIDKDSYTKTEQGSETSKAKNNEKDAETLTNNVKTCVPPIGSWTNNPLCTRQSIGSLEYPQDCLYQLAILVDNPGYVLFHNGHAPKPPLSCVREAPVCEPAPASWTGDSLCIATRYGRLEFGPDCRFTIRAGSYHFVGETSGHTPVPPRRCGLVNECRKGTYGALCACHYTYSTDLICTGDHLCPSSSCASSEQFIAFFGDFNKYGDSVFLIHLSNGSSHIPAPRNNGCFRGIVGSNINDGDRVEVYDYRCSHHHELSLMMFRLEITFPR |
| RRRRRTRINITY_DN26775_c0_g1::TRINITY_DN26775_c0_g1_i1::g.5204::m.5204 | NA | NA | 6.9 | VSAIVIMLHPSISSISNLLDPALRSLHSESFECPPFIQVVYAPQNSGPNPVNIIGAAQKAQLYTIFGSKLAQTQGLVDEQDRGCPLALLLCYDNDVQRARYTTMRRAVSDLQSAELRMRQVIRLLPGGEPPPLSRQALLTNGSVFHLQVAAQDNKLALLGQWVIPYKTLLQVMDVSRHVSHDLKADPRPQLSSSPPVAPPPTPLFSPPGKQLSLDPKHTLSMPSTMPSQYSPSVT**DGAIRSIH**SLDSSTAHRLHSSEPKSDSGGGHSQSMKSRHGLDPEPPVQSSTIGQNALSHGPFQSPFGRMDRYVPFSGTGGPHMLQVGHTHPSVMVPSSAHVGRIRSESSPPHILKGELPKLSEKTSLEIAKGVIPSKSPGS |
| RRRRRTRINITY_DN36542_c0_g1::TRINITY_DN36542_c0_g1_i1::g.7636::m.7636 | NA | NA | 20 | RTIASAEPTSPASPTTFPNRAVVPESELPEGVGYKNVATVRFIYENGPLLKTIKYSIAQIKPEVMTWSLRSTERKEIIYHSISAGGDHSPHNWHLYCKEATVGKVELPGDPPGPRDLVKVNIPIMKVGGVNVLKLVFHGGDVRVCDKVTLITRTLTTKVEMRQTAKDIENGEKLWVVEPLPKGTYDADIVFTEGAHVVTLDKFKSDLTAEPAEIEDKATIEGTSHSPR**SSVGAANRAI**VRFEYVCDETAGSYTFQTDIINTFSAKMWRTDPHKRREVVYGTIASGGDYQPKTWQLTVHNRTIDIAEPTGPPDCPDRAVTTESIKSAPSIGAINEAYVRFEYEIGEELETTKFRTDPILLKNLKTWLLSNKEKRELHYGLVPSGGNNTPPKWEVVMSDKVAASIIPTGPAAPESFPYQAVVAPSIIASSKGYRNEASVRFQYEVGTKLNTVKITSRAITASVIQWATTTTDRKEVIYNNIQCGGTYEPPEWSLTVYNSSVEEVKVPGTPPGPKDLIIVTLEETATGVSSTATLIYKGSDEKNADKICLKTSSQTSTVNLRTTEKLAHGDKKWEVKPVPRGIIPIEIKLDDGHLLSYTNVAIKFGPEITVDKATVPAALSRPDSRGKENFAVVRFLYEHGTSLGSVTGECTKTTTA |
| TRINITY_DN39983_c1_g2::TRINITY_DN39983_c1_g2_i1::g.33393::m.33393 | gi\|736153680\|ref\|XP_010764284.1\| | PREDICTED: receptor-type tyrosine-protein phosphatase eta-like, partial [Notothenia coriiceps] | 22.5 | ETEGDTKKLTTVTTPQPVGELSVTVFTTSSISVTWTKPEGYSFFYRVQWTNGNDSHTTNVSQTSITIPELTAGVKYAIHVTAVADDNHTEGQSVSVSHHTRPTKPGNISGQGTLNLSISWDLPGGKVDQYVVNISNIMLQDFNTINTADTKAHFNDLKPGRIYNVTVTALAGDLSNTSDLTSLATNPTPPGSLNIIERTNSSLQLNWAIPVQMENAPGISYNITYQPVVGEVQHNTALNTTVLSPLSSGIRYNITVVTVGPQDLRSTAVYASSYTLPNPVLNLRASTESTTSIKVEWQYPHGAQPYYKYLIKANSATGKPVNETTDNN**SIDLTDLEPG**SRYNISVSTIIEEESGLVSTEEETFSYTMPKAVIN |
| RRRRRTRINITY_DN58270_c2_g1::TRINITY_DN58270_c2_g1_i7::g.45040::m.45040 | NA | NA | 9.6 | HPRTQTIMKLRSQDIVDLLNKADVALAHAAMLMQKKYDKQLSTMVYQQALKMKAILEALDSNLLKQAMEIERHTSAPLVPITEDVTALLTRLALGVEKVMPVYEEPAAPQIRNSMEIVAKVLGTVNEYVKDNSRDLNATPPPSIEQPQLKVGENYSDVPCLSALNTLHSPAGPRPPKKPPAVHEPKGVPQYIHQSGGPAQLTGDERDISGRSNRSEAKMFSEEQELWRQDDEMQQQQMMLREEMMAQGVCGGDEMNPSWLANHEPRHQNWSDTPPYSGGVGGVGGHASPPFHNHQPSSFFGESSRPSPYGPRSPKPPAEDSGGSDWSVTVQRRMEMRLREEQQLKEEELITSLQTKLETFRPRKSPDYSWCKTMLSYLTPPCQPPMALREGNEIRGIVDNNKVGQFPKIGYMLIEWMCVGFMWVDSASTFRRFNISEPAMWKIPLKGKSAKYYSSDEMYRSLGFDGLMVCDVSSVLVNRAAIDRHVFRKSELYALATSLQYAFLILTALDLSYKRVQLFSRLEGLTCLEMIIWVPNETIVGILKVIHPHDFQRMTLAEQLFKERVSDSTCNKCTKIAVPLGPKDPSNYVGQHVDGFQGEGICRGLEIRDRQIEYDRAEVVGYSMSPMTYTDEEDVIEAYDDTEDGSLDENPPSDSCFVFPLLFRRAKTPKPPTSTVLDSVSIARVGSRVAELKKENNSLKPISPLAREGEKQFRVIFSHTEMSILRCYGDILDALNEAMTLSATTVTLPEAAGAVNLQLMGKRDKEEMASYQISQVQNFNALHTPTSGKDTLYSIGEEPGIALEVQIVWSSGLACKFCEKDYRYIPALIELFKLICQEDNLNAVQKFTQQILKRLSKAKVSDLLDKPFFRRLGVDKELLEYNSKKDLANGRMEKFFRRIELCGLKLAVDQEVQDGIDTMYDTKVQHYFYNLTPKDETFQQVFGKPLYRIRLEYRWEENPRAQEYKERVHSVGMDPHLWHLQDRSQSNSLRLGFCSTWRVKHIDLIKQIIGRVDTADGYRINSSWCSNETNTEFHHFVKLVRDVSGAMTRDSGGASLRSKAGGLLTNNLNPDLYATAM |
| TRINITY_DN45326_c0_g2::TRINITY_DN45326_c0_g2_i1::g.11399::m.11399 | gi\|928054232\|ref\|XP_013876370.1\| | PREDICTED: 26S protease regulatory subunit 4 [Austrofundulus limnaeus] | 20 | ADVTPERQATMGQSQSGGHGPGGGKKDDKDKKKKYEPPIPTRVGKKKKKTKGPDAASKLPLVTPHTQCRLKLLKQERIKDYLLMEEEFIRNQEQMKPLEEKQEEERSKVDDLRGTPMSVGTLEEIIDDNHAIVSTSVGSEHYVSILSFVDKDLLEPGCSVLLNHKVHAVIGVLMDDTDPLVTVMKVEKAPQETYADIGGLDNQIQEIKESVELPLTHPEYYEEMGIKPPKGVILYGPPGTGKTLLAKAVANQTSATFLRVVGSELIQKYLGDGPKLVRELFRVAEEHAPSIVFIDEIDAIGTKRYDSNSGGEREIQRTMLELLNQLDGFDSRGDVKVIMATNRIETLDPALIRPGRIDRKIEFPLPDEKTKRRI**FQIHTSRMT**VADDVTLDDLILAKDDLSGADIKAICTEAGLMALRERRMKVTNEDFKKSKENVLYKKQEGTPEGLYL |
| TRINITY_DN56997_c0_g1::TRINITY_DN56997_c0_g1_i2::g.105558::m.105558 | gi\|657564694\|ref\|XP_008285610.1\| | PREDICTED: protein FAM49A isoform X2 [Stegastes partitus] | 25.6 | MGNLLKVLTCTELEQGPNFFLDFENAQPTDGEREVWNQVNSVLQDSESILSGLQAYKGAGQEIRDAIQNPNDLRLQEQAWNSVCPLVIKLKKFYSFSLRLEEALQ**SLLESLTCPP**FTPTQHLEREQALAKQFAEILHFTLRFDELKMRIPAIQNDFSYYRRTISRNRINNMNLDIESEVNNEMANRMSLFYAEATPMLKTLSNATTNFVTENKTLPLENTTDCLSTMASVCKVMLETPEYSSRFSSEDTLLFCMRVMVGVIILYDHVHPNGAFNKSSKIDMKGCIKVLKDQPADNVEGLLNALKFTTKHLNDESTPKNIRTMLQ |
| TRINITY_DN38384_c0_g1::TRINITY_DN38384_c0_g1_i2::g.66945::m.66945 | gi\|736301518\|ref\|XP_010793302.1\| | PREDICTED: nexilin isoform X2 [Notothenia coriiceps] | 16.8 | KAEEEAKRRMEEERRAFAEARKSMLVDEDDEALMALLNLEGSKPGKICASFEELERQRREEEQKKAEEEAKRRLEEEKRLFAEARKSMVVDEDGEGLVKSDSQEALNPRKLE**INFEELL**KEKEKAERRRKAEERKKKMEQEKQEFEQLRQEMGEDEVNESSDVVSKEYEELTKLKRTGSIQAKNLKSKFEKIKQLTEEDIQKKVEMERARRKAIDDEIKEREAERFQEEDEERESTPVRAEESPFKQKVDMKARFEQMAKAREEEEKRRIEEQKLQRMQFEQQEIDAALQKKKEDDGEDEGSIINGSAAYEDEEDHARSGAPWFKKPLKNQSVVDSEPVRFTVKITGEPKPEVTWWFEGEMLQDCEDYQYIDRGETYCLYLPETFPEDEGEYMCKAVNSRGTAASTCILTIETYDY |
| TRINITY_DN58549_c0_g4::TRINITY_DN58549_c0_g4_i1::g.62889::m.62889 | gi\|657550921\|ref\|XP_008280000.1\| | PREDICTED: zinc finger FYVE domain-containing protein 1-like isoform X1 [Stegastes partitus] | 8.7 | MSGQGPAVDKGMNTVLVCQESYACGGTDEAAFECDECGSLQCARCELELHRQERMRNHDRVRIAPGHVPFCDSCKGDSSCSGNGGRLRAVVRCQGCKINLCLDCQKRTHSGVNKRKHPLTPYPPAKAPQENSVSAGEAEIEILKAKLEKVCSFLLVDEKEEMQVKDDDEFVGRLGCRPDELLKVVSIFGNTGEGKSHTLNHTFFLGREVFKTSPTQESCTVGVWAAMDPVHCVV**VIDTEGLLGAGA**NQGQRTRLLLKVLAVSDVVIYRTHADRLHDDLFKFLGDASDAYLKHFTRELKATTSRCGLDVPLSTLGPGVIIFHETVHTKLLGSDKPNESAERLLQERFRKLGLFPEAFSSIQYRGTRTYNPPTDFSGLLRTLEQQLDNNTTRSPRSASVIYKALQALSERFSGEITDDYMTSNSFFPDEYFTCSSLCLSCGSGCKRSMNHLKEGLDHEAKHRCRYSAQYDNRIYTCKACYEGGKEVIVVPKTTASSDSPWFGLAIYAWSGYVIECPNCAVIYRSRQYWYGNQDPVETVVRTEIQHIWPGSDGFLKDNNNAAQRLLDGVKYISQSVSELSVKPAKAVTSWLTDQIAPTYWKPNSLILQCHKCAEEFQPNDTKHHCRSCGEGFCDACSSKTRPVPERGWGLAPVRVCDACFHNRGIPTELLDAALEEEGGTLIARKVGEAVQNTLGAVVGAIDIPLGLVKDAARPTYWIPDQDITSCCECQREFAPRLSIHHCRACGQGVCDDCSQERRPVPSRGWDHPVRVCSGCNQKPGEL |
| RRRRRTRINITY_DN57666_c2_g2::TRINITY_DN57666_c2_g2_i3::g.61717::m.61717 | NA | NA | 10.7 | VISKLTDLAAKPEEPECPAESDAIARHYEQLISIVLDHGFQMGDDLPTNGWRDKMHPNVKCVETLFIVAEVHGEAAAIHLATRSDYDRQEMDIASLAFRRLASVDGSYAAFMLNVVSKNPDDDSRRRPDHKKTFHRLNDYNHFNFNSVLEQCFHIGRVSNGLRDLPPSWCMMGMVNPVVLLVAGSVGSKAPLGVHFAFQGSFDYMGCSHMLSLTNRVAEASLV**REGTIPCIGG**NALTSAMVSGSECTVEISCLQFYFDLAATMDANDPFCKKEKLYYGIAYNRDGTERESQFTANSFGVFETGAMSKLFEMVYDFREAKNSHPKILSSIVIAGANVMPNHPKDEENLSLKNFKFGSPEKGVFQHVRESGLEHVAIAYELPKVCSQLCLPVKTDGVSYRQGDITCLSAGWLDSSFKALQPIYDAVQGGEQQQASDFMKDIQQTFEDFNPIIFKKRFAKTLLMINNGVCRKFFKKDMMVPGVSDRASQRMQRVCDRLRPDSTLLGTKRLASIFQSLPIMEKGETITYFLLDEMSSMLRSRNSHGQDVEPGKQHHFARHTHPSAAVWCPLPVPQQTRSLVINDAGVRASIGANKFLQLIGPNATRLSKLCHM |
| RRRRRTRINITY_DN54378_c0_g1::TRINITY_DN54378_c0_g1_i1::g.17758::m.17758 | NA | NA | 11.4 | HLVRLVPDPQNLDDGLVALGQETLGVPPLHYLFHRLKPDSSSGEVARIVVP**RADLAAPLAG**REDHGDRLRRRGLAFDHQLFVVAPADLRLVDEAPHRRVHGMPDLRQTPRLLQRLLPAGVRGVRDLHEQEGAQRLRGGEEGRQGDLEGAPDLEDEHVHTVPVVVAAQHLDAHRLGGGVDELHRLAYIAIAVTRPLEQRQSGHVSEQTRDLGLHVVQEDETQDEDVQQGPHVEEDCLSCCHFSKHNQDSCQSGVVGDQQHGPKRNQSQQLQGVQVGQVAVAQLQEAVGVGGGQGAHHSGVPGEVGGVRGQEQGGVQGVAQQDVVELLLPAAAGAAALVAGGVQLPQGAQPVPVGGVQQVGDGGRQLVEGRVLRVPVFVGVQQQLAQGEGVQPQARQDVQVVDLGQGGPPEPARVCVTCSLPQQQGLVRRLWM |
| RRRRRTRINITY_DN51640_c0_g1::TRINITY_DN51640_c0_g1_i3::g.38275::m.38275 | NA | NA | 49.3 | ATRVPPVTLDPLDELDLPDLPVLPDERELLELLDPCVRCALSDETDRTDEIELRVLREETERPVLPALLVVSVLLALPVLPVLLAVREVTVLRELPASVAL**PDLLVLPVLP**DLMALSVVIERPALLETAVLPVM |
| TRINITY_DN56629_c0_g2::TRINITY_DN56629_c0_g2_i1::g.124889::m.124889 | gi\|657582301\|ref\|XP_008295204.1\| | PREDICTED: uncharacterized protein LOC103368577 isoform X1 [Stegastes partitus] | 26.6 | LFSHVKDYETKTGLRVLPALHSVLQSVPSVWIINLSERKTSILLEVLKLQPEKKPVKLTGWSDEESEVRSFLQCLPYISQLSFSEEFDVSDGVKLFGNLICAAAEREHQTG**EKILEMLT**SVYRYETFPLSDRDMNDYFKRRYQCSFLLDLFSHV |
| TRINITY_DN50020_c0_g1::TRINITY_DN50020_c0_g1_i2::g.117602::m.117602 | gi\|734639227\|ref\|XP_010748390.1\| | PREDICTED: glucosamine-6-phosphate isomerase 2 [Larimichthys crocea] | 21.1 | MRLVILDDYDLASEWAAKYIRNRIIQFRPSSERYFTLGLPTGSTPFGCYKKLIEYYRNGDISFKYVKTFNMDEYVGLPRAHPESYHSYMWNNFFKHIDIDPANAHILDGNADNLEAECQVFEQKIAEAGGIELFVGGI**GPDGHIAFN**EPGSSLVSRTRVKTLAKDTIVANARFFGNDLSKVPTMALTVGVGTVMDSKEVMILITGAHKAFALYKAIEEGVNHMWTVSAFQQHPRTIFVCDEDATLELRVKTVKYFKGLMHVHNKLVDPVLSIKDQ |
| TRINITY_DN73442_c0_g1::TRINITY_DN73442_c0_g1_i1::g.94183::m.94183 | NA | NA | 36.2 | MRLGSGDPCLQKQRQTRLVEPPGGLHCD**EDVLVPQL**RLGDTCALRARESTVSSTTSSTTTVIISTSRLERIVGREDGGSLLDGAVVRAGGWMVVDHPESGGVAGA |
| TRINITY_DN56898_c2_g7::TRINITY_DN56898_c2_g7_i1::g.43127::m.43127 | gi\|657564868\|ref\|XP_008285705.1\| | PREDICTED: elongation factor 1-delta-like isoform X1 [Stegastes partitus] | 21.1 | M**PKQSTNMSGVQC**LATENIWFDKHSYDEAEKRFYEGVNGPSTQQQQVKTALQQPKGRQQKRQHRNSSSHGGDQELVTRMKSLELENQTLHKVVADMRAALQKLESRVAS |
| TRINITY_DN55970_c0_g1::TRINITY_DN55970_c0_g1_i2::g.86185::m.86185 | gi\|657754097\|ref\|XP_008312570.1\| | PREDICTED: peripheral myelin protein 22 [Cynoglossus semilaevis] | 7 | MLLLLLGIILLHVAA**LVLLFVSTIVS**VWTSGETSTSDLWINCSTANGGFHCDPASTGEWIQAVQALMILSIIFSCLSLFLFFCQLFTLQKGGRFFLTGTFQILASLFVMSGAIIYTVMSPEWVPDTNAFGYSYILAWVAFPLALISGLIYVILRKRE |
| RRRRRTRINITY_DN94585_c0_g1::TRINITY_DN94585_c0_g1_i1::g.95793::m.95793 | NA | NA | 17.2 | QELEAIRDEAVVLKAQLDKEATCPEVKDLLSQSVQCLLEEKAQLKQETE**ELSNGLAT**MKVLAEKLDEEKREVLREYMEKTQRIEEEKERLFQSMKQFEQTAREYSHELTYYDAEET |
| TRINITY_DN60101_c0_g1::TRINITY_DN60101_c0_g1_i1::g.92996::m.92996 | gi\|736230689\|ref\|XP_010782413.1\| | PREDICTED: chromodomain-helicase-DNA-binding protein 8-like [Notothenia coriiceps] | 13.9 | QRNVRSVACCDETGLSERGALPLGLYGDGVGSSLEDLVDVFLTEFGA**FVFFVHQSSVRSLS**QQVLNLLLGQLLDPAVGVLPSAHALQHGLVQSQLQTGLVK |
| TRINITY_DN58729_c0_g1::TRINITY_DN58729_c0_g1_i1::g.92118::m.92118 | gi\|734629649\|ref\|XP_010743126.1\| | PREDICTED: fructose-bisphosphate aldolase A-like [Larimichthys crocea] | 71.9 | QYKKDGADFAKWRCVLKITSTTPSQLAIMENANVLARYASICQM**HGIVPIVE**PEILPDGDHDLERCQ**YVTEKVLA**AVYKALSDHHVY**LEGTLLKPNMVT**AGHSCPKKYKPQQIA**MATVTALR**RSVPPAVPGVT**FLSGGQSEEEA**TLNLNAMNQCHLHRPWALTF**SYGRALQASALK**AWGGKKENGKACQEQYITRALNNSKAAVGKYVSSGDKGAAAQESL**FVADHAY** |
| RRRRRTRINITY_DN12320_c0_g1::TRINITY_DN12320_c0_g1_i1::g.83169::m.83169 | NA | NA | 16.3 | LLLLFLLYPLTFPPLFPSILLPIPQT**LSILSSSF**IILLFFTHLLLFYLFQTLFLILYQSPHILLLLHFFPLTLIFQQFFFYFPHLPLYPYSP**IILLLLPF**SFSLSFSSSPFFLSLFLLFIPLFPSSISFFIRFHSSLPVLP |
| RRRRRTRINITY_DN58368_c2_g9::TRINITY_DN58368_c2_g9_i1::g.45504::m.45504 | NA | NA | 37.5 | KTLLLRTYGDATEIKINSEDSLEEGDLYWQVKIPERGIYPIKFAAKQGKKVTVPTKFKELEETDFRPPDEVFIQAETKRGDAEFKYTGSSEETVKHIKLRHFTGDKTITIGDESKIEEGDKYWIGQCDERSVKCEIEAAEGVIAKCDSLAAHFHVGPDVVPEEVEVLKKKSRKGKKGTKGGDGQNTTEEDNSDGVVPEEVEVLKKKSRKGKKGTKGGDGTTEEDNSDGIVTDPVLPGDRARRRKGKGGEGGEGGEGEGELDDDSFELSDFDEDGFEGGVG**AGEGGAAGGAGAGGAGAAGDVGGA**GGAGGAGGAGGAGGAGGAGGAGGAGGAGGAGAAGAAGDAG |
| RRRRRTRINITY_DN49208_c0_g1::TRINITY_DN49208_c0_g1_i1::g.56946::m.56946 | NA | NA | 21.6 | SLSGSLSTITLDRQQLEGSLKSVEKRMGEASHPQQARHLEERLKKMEAVSSDCEAKMNRLDQETQSQDARQRTVKDLKERLCALEAKLQVESTEAFHLKSATRELDLRMTEIGTSGQAAVCDELSRIVQDKDQLVQNLRAAENRLRQCELSTRRVSTDQSDLLAHLEEREGSFVQRERQHQGVCMDLPRLRQLELELEQSHLSDQIRVLQSRLSQVESCCERHEREKRCSARQSTVH**TLEETLSKNQAE**LATLQTQYQIHQQEWEACRRHYEKLKDHLQTVESLEAEKSRASSEKFQKRQLKHHYSRKLKELEECVKQLRKEYKAVLEQRDTQADEVQKHLLKIELDRREILSKSTLLEEETTKLRYGMTQIETEWERRQHNVMIDIQRMLEQLEPECSSLVSSLDPNQLSGLYAEM |
| TRINITY_DN34194_c0_g1::TRINITY_DN34194_c0_g1_i1::g.112725::m.112725 | gi\|734624207\|ref\|XP_010740142.1\| | PREDICTED: stomatin-like protein 2, mitochondrial [Larimichthys crocea] | 11.8 | MMLRTLCRAGGALLQQSQRTAPRLWVTPAQQRWASSLPMNTVVLFVPQQEAWVVERMGRFHRILEPGLNFLIPILDRIRYVQSLKEIVIDVPEQSAVSLDNVTLQIDGVLYLRILDPFKASYGVEDPEYAVTQLAQTTMRSELGKLTLDKVFRERESLNSNIVHSINQASDDWGIRCLRYEIKDIHVPPRVKESMQMQVEAERKKRATVLESEGTRESAINVAEGRKQAQILASEGQKAEQINKAAGEAQAVL**AKAEAKAKAI**RMLSDALTEQNGNAAASLSVAEQYVTAFSNLAKESNTILLPSNTGDISGMVTQAMTIYGKLAKTASPAAATATAEAVEKEMEEDQNQSTSTQ |
| TRINITY_DN39161_c0_g1::TRINITY_DN39161_c0_g1_i2::g.77899::m.77899 | NA | NA | 13.5 | MRAAQPATHSRPSWARPCGTRRCPTTGTTSSWSTWIWRSSCRRTVSLPPTRPRASRLSRRRSSSSRHNNRLLRHH**RPRPLPPWST**SAAARPPRFTRAWRLRPASAAPAAQQHCQHPGTPPAPSTQSPFRCPSPTTPTRQTWLCPASPARKCSTQGNASSPQRS |
| RRRRRTRINITY_DN55113_c3_g13::TRINITY_DN55113_c3_g13_i1::g.60702::m.60702 | NA | NA | 15.9 | ESATRCCGTLISVDTRQRKGKIKKYKPELSWRSRVKDDTVHRAALAASSTKGNQFSNQR**FRLLLGRAN**RASQFSETHVYVTPLLRRDCAGGGKFAPPPPQREYAGGG |
| RRRRRTRINITY_DN57974_c2_g2::TRINITY_DN57974_c2_g2_i2::g.44166::m.44166 | NA | NA | 57.4 | MSRIHINVTASDSGLENNLRLTYAGADDEKVASIVLTSLDSTNEVRYREDGNPLIRGSRVWEVSPAPDGSFLGEVKLSKGPEISVDHPLAEIKPPSSIVNLSTTASCQGFQNKAKITYKGTDAVTAERIELTYVHQSCSLRINSTASITVNDKLWEVEPSPEGAVECTFIVNQGEDVIQSRPQLLFHPADSRKAPDSTLSQGQTISMQSHSSVVEKHVMEQKVVEESIKSARVTVTCTTSVAGSSNAATCRYEGSDSVESEYIELHVSGSEEFIEYKGGQSLAKGNKLWTITPRPEGTVKVSLKVISDSNATVDSLGVSFTPKREITPSMVRKPSKIPSTVREKESPTPSKPTPSKPTPSKPTPSKPSVPSKVRQPSKVPEPSKVRQPSKVPEPSKALPSKAEPSTVRQPAVVKEKSESKRISLTFHAEQKGGANEVELTYSGEDSMEVASIEFSSNYQTSVIHHRASSGIVAGEHMWTICPAPEGAVDCEFRALDGEEVTLSLPKTLIKAPVSPTERPREVVTTTEKVTEKVSERVTTKSSSVHYVETRTMEPLHSSPPEEDRRLSSFAAYEEGAVDLTAYDSTEGKASKCVVRYTGGDETVCNIIQLTLVGSINTMHYKHSQHIEKGNHFWKVEPEPKAQVNLTFRTNTGYPVRHSRLRITIRPAHDLSFPAQTVVEFKREVSRRESRLAQKREEYRSAFDVKPYVIDTKVAREPTPSRRREPEPTPLKKIPKPIYESTFATLHESEVGESYATVTVTSKQKTTRTKEEFEMRKLESKYSSIRQATKHPRLLEQDEREAYVRQKERPPRHTTKDHRQRLDIFSPGAEYKSPIRYSDYRHVEEGEEDDSYARRPSSPRLASLEFRVPSLSPPSASYGLELEEELRLREEETRKMSLYEDVPLLLEEDSLDGLSRRRIRHYADIEPSPTRHRAPRVTELDDQTMSSIRWKPKYQDKIRIPVEYFQEWRSLRIRKMRKQKIKDMPEPFEYQDRLKKYWKMDSFPKFHKIEVDEELVAPNIVRPAPVDYPMRLRKEDARKKREEQEKKIEAETKEGKKTTVAPKFMTAAERVAQVAAPPLPTIVTGSLIQSLRVKKDLEEKKAAKEKEKQSPEYEKKVHAVREVKLTAQCSASGASNTATVRYVGSDEPLTDRVHLIYYDLGEHVVEISPGFALPTGDKEWKLTPHGSVRIEFRVKQGERCEVNALLRKFMPRLTLDAPKPRPVVTLRSKCSDDGYRNRAVVSYEADDEKDLDHIILQYLGKDTIFTYKKDDQGPILKQGSKHWMVRPEPHVTITVGFRVDEGIYATRNVLPLTFEPPKQLMRATDVRRKTRKVVRTTFFDRYKRVGNIFLEAYASDEGYENVVKCRYTGDDARTVKSVTIVALGDEYDIEYKNGAELQRVGCYWTVETTSDYNDINCIFKVNDGENAVAHTVQGALPGHEFPAIKVKAVFVGRQSRISGGCAVRAASIVTSWEKRVMSQYYRKHRGTPIARGSIEEVPKTLWPHALAEAASMRHKRDKTMLRDTFDLAEVSVHTFSEDDYSYAANSINDIMQQNTEATFPNLGSLLVYALVGVSWMDTVTSVMDCQHIEPAAYEATTYQVKIQDGPTLHRSQGLEIIKVNSSTRTTYVINEPRIDFHGYSQTHLFELASCIQRIYNAVERENLEFEASSLREFIDGGSIFDYIMVLEEPSDFSEHLLLFSTHKALNLTAIEKKVIAQDAGRVKVFKAMYTKESSIDVCRHVIGFQGRGLEEAIAYKNHLNKAESNPAKGKRVHGTDDVERDYLLVRSKDEKTMVPGSTESPASQGFKNEAIARFQYSTGGFLNIVTYRTDRSQAVREWREATTPCKEIIYSIVRSGGDNAPAVWNLTVSDRSVDSAKIGRPPDPVDAVDLEVTQQDIGFRNKACVIYFGADKKEVGNPFVLATFSRTVIVQYHGNSDILDQGKQWTIVPDPKGSFPIKINVVEGRLAPIAEKDKLNKPLHIKAPVEVDLSVTACISGGQNTARIQYVTSDEEDAETIVLQHYGGKFEQIKIKKGDSHIEKGRRFWKIVPKPQGTIKCVLTANSKYKVSMNRLEEKFAPARVDVATAPIMPTTEESYDSQGGLNECKVRFIHEVGETLRKVSFVTQKIEDTTVPTWETWEKKDKKHKKELCYSKIKSGGDSLPPKWSVVCSDKTIGSVVPSQPPSPKELQSKILIPSTLELPESNGYRNQAYVRFFYGASDILNPVRCSTGNISSSVLEWETGEKDERKEVIYNTIMCGGDDKPPKWSIIVSNKLLADLVIPGQPVDPKDYIEVTFPTDARGFHNHLRIRYKGGHVRRQVNKIVLHTYHETTEISIDDTNELTKAGHLWMIKPEPRGIYAAHIRLTTGLGAYYIDKLPYDPHFSPAAELVLVGSTEADGADNIAKCTYEGEDEQQDTMISLSHNRGDSSMEYKRSEVIEKGAHYWKIEPVPRGIIQCKMVAPQGLKTTVEDMEKRLSPKTEVSLKTKICTVTRRPRSMGTENEAFVRFEYETAEALGRMTFEMQKLRDRNCKKWTSEVSKRYEVWYGLVEKGGDCEPAQWRIAILNNTVNVTDIEGPQSPKEFPDKCTVPDSVPGAESEGIDNVAVIRFQYEADPTLGPLTFMTSSIKTEYRSWTESSVLKYEVFYGTIASGGDDKPRSWALAVSSKTVDMIEPTTSSPEPPCLPTKAMLPEESKLSASVGFSNEATVKFHYETGERLGKVVLHTDTVRSDVTYWANRTAERREVIYGLIESGGDETPAKWCVKVSNAQIDEFVMPGEPANPRGIVKVKIQAKKKGVRNELLLDYMGSDSREAGKIVLESADETSAIMAKTSVAGSDKLWKCTPHPKGKIPVSLRVVGGHRVVYVDKYKLELDYDPSELMETVTVTGPVEAPEGSGGANCAMVRFKFEEGQPMKTLTYECILSPTTNCKIWAMTGVKQMEILYGDVRADGEDDPPSWALSVSSKTTDTIRPNQPCGPPDIPDTAVAPKSCLSPKSQGRANIATIRHEYELGEILETSRYRTMTVPNKNARVWRDSRVEKREIIYGSIDLGGDEEPPEWAVTMGDRSVDFVVPRGPPGPVNKLSKCTVIESQLFDGTGYRNVAAARFVYEDGEILNVFKFTPRSVSDSVPVWGPRHADRQEVVYGSIPAGGDRAPPDWSCTVCTADIEDLRFPGSPEDPKSLVVVKIVETIVGVANKVELSYDGTDNITTDSMTLKSFKGTSKIKVRDTVKLKNEHFYWSCVPPPRGIIPLDLELTKGALVNVVKQPISSFDVMPMVRQEQVTIATVLEKPESAGRSNVAKIRFLYQEKENLKEITYEFDTLKLTAVKMWKDTGFKCAEIVYGTLRSGGDHEPKEWGLSVTSKTVDIVELKNPPLPVECVLIVDPTESPEGIGYINEPLVRFYYLMGEMLGDIKYSTKSCKATVTKYARMSAERREIIFNNVPAGGDVAPAEWSITASDKTLEKIKLPGTPGPTDYVKVLINVEQKGASNEAVLTYKGADYRHVKDIILTTSSSNVEIFGRTHLDVGPKSWTIAPTPRGTVHVFMRLSGGARLCITKKLDDHLEFDPPEIREVPETEACPESFDGTGNINIGAVRFCYKKGASLGTVVHETTRLTKEHAKSWEEAGAEQMELIYGMVDSGGDEVPRSWALSISDAHTDTIRPISPPAPTCTPVRCVVYLSVESAESEGAKNIATVRFQYINGETLGSVKLTSDLHAGDRNVKVWRASKRERREVLYHILPSGGDSEPKLWEITAFDKGSALIEPAGPPTPVTFTNRAIMPGSQLHVGPGYKNVGRVRFLYETNKFLGSINCTLEKCEANMIVWNLRNTDRREVIYHSIDDGGDEVPPKWSLTCSEETIKSIEISGECVGPTDLVKVNVEATKTGSVNRVTLIYKGTDDRDCFKIIAMAKTSTYQLHYKECLELDRNGKSWSAKPDPRGGVAADLVLDSGARVMVTESMLKSDLQAKPPEFDTKCQVSGTSESPLSFVQNTNKAIVRFEYPEGEGLATVTYFQDTVNTYNCKLWRGEGTLTYPKREIIYGVIPSGGDNLPASWKLSVTTRTVNTVEPKSPPDVPNQAVAGKSAESAKSLGASNMAYVRFQYELGEVLAEVKHYCEFCPIKNERVWLINNREKKEIWYGTVKSGGDHYPEEWKLTMTNATVNFVSPKTPPGPPTFPHEAVITESETSKSVGFRNEASIRFTYQMYKTLHSVHLTTRKVSSNILQWATSGSERKEVIYNTIMCGGDDKPPKWSIIVSNKLLADLVIPGQPVDPKDYIEVTFPTDARGFHNHLRIRYKGGHVRRQVNKIVLHTYHETTEISIDDTNELTKAGHLWMIKPEPRGIYAAHIRLTTGLGAYYIDKLPYDPHFSPAAELVLVGSTEADGADNIAKCTYEGEDEQQDTMISLSHNRGDSSMEYKRSEVIEKGAHYWKIEPVPRGIIQCKMVAPQGLKTTVEDMEKRLSPKTEVSLKTKICTVTRRPRSMGTENEAFVRFEYETAEALGRMTFEMQKLRDRNCKKWTSEVSKRYEVW |
| TRINITY_DN57211_c0_g5::TRINITY_DN57211_c0_g5_i5::g.21089::m.21089 | gi\|542186679\|ref\|XP_005469859.1\| | PREDICTED: sorbin and SH3 domain-containing protein 2 isoform X12 [Oreochromis niloticus] | 18 | MNTGSDSHSSDLDSWRSRSATDGLKNGDASNSSLAAKGFRSVRPNLQDKKSPTQGQVLSHAMNGSTSHPQRPLSPPSYPPPPASLHTGLPRQSRSSEGSECVTRESVVSGHTSVCSTVPIARFSEEEKKVSVIKAPHYEGIGPVDESGIPIAIRTTVDRPKDWYKTMFKQIHKVHKADDDYSDTYNATYAVINNDDYSLSSTATMAHPPPRTHTYRPLSKSPSDNGGHLGPREPSPSPVPPPPPPMPSLLQLRARDSDRDKDSPDMNEWGPPDRKVDTRKYRAEPKSIFEYEPGKSSILEHERPTYDDIDLENEPWYKFFSELEFGRPPPKKRLDYNPDISARQRIETSLHIAPADKAPERPASAASDYRKRRKSEPSSSQVNAQSQSRSVISPKPVDAYRPSSSLKKPVIRSSPSSPSRAKDQDVSRSYSTMDGRHTPQSRRPTPDREKQPARAIYDFKAQTAKELTFKKGDAVNIIRQIDNNWYEGEHRGRVGIFPISYVEKMPSTEKQQPIRPPPPAHVREIGEGVARYNFNADTNVELSLRKGEKVIVIRQVDQNWYEGKIPDTTKQGIFPVSYVDIVKRSPSKSSAHHIDPHGHPGIRTPSSTPVKPFYHLPPPSTTRDLPSSPHPPLRRLDLLAVTDEWLSLTMVPSASTPPHSIAATPLPPTPPPLPPDLTFLQRAQERRTPSPSPAPSPAPTQRGLALLP**QTFSRTPP**LKEGRLGVGHAPPALPLSFPPSPVPSPPPPATHFSLTSSPCDSSLRDNSSWEFIETPQKLRNAKPEFFSCTAPEPIKSPTQTPQSHRSTAPVNKSHKPPDIELQVKDPYDDLLTMILDGSTSAENVDFSRWSPIDSPPAPLTHETQSSLKLKAEEPNQTEWETQVPTPAGQPAGDVRLEVQFHKPMTMEPLSITWGEQPETLNDDEAVEFEMPLSVEGKGFTELFIEEEDDDLEEKEEDIRVLSDRLSPQADVSPSALTWLSHPGLSSPSLPPPLTSTPPPPASSLSSPSSVPPPPPPPPPPP |
| TRINITY_DN55782_c1_g2::TRINITY_DN55782_c1_g2_i2::g.20017::m.20017 | gi\|657573930\|ref\|XP_008290650.1\| | PREDICTED: LOW QUALITY PROTEIN: protein FAM184B [Stegastes partitus] | 15.3 | RERDQFEKDRNTLRREREEEKEGLHKEVEEIKERLRREKEEELERQRKELEVERVRVRSQLDKNIEQVEAERANVQQKLEEEKKRLVEKAEEDRKRLKEQVRKAIEEVMRRHAAELHSVQEALSSEKKTNQEVCTRLEEERRAGEELRNELEKEREELRTKLKDATNEICRLESAIQQQEKKDKAAPEAAASCGPHCSRLEEELHQTRSRLARVQEDAEKQWDRQQREIASLRADKHRLEEKVLEQSRLNTERSLLEQSQRHTEDRIRAECEDRLRAEFRIEMNAAVAESEQRWQNREQEMQTQMSELQGQLDEVEEKKACQGDDCHGNPEIDRLRKEVQETKEINKKLRDLLQEPQSQSLAEERHSHTMALQTLEKKAKEDVLSERNRLLTMHHLELDKQRAELTQQHTEWSRQMTQRHMQQIEDLQAQLQAHTQMMALQQDLKQQNQNQVFERQLDESRCAMLELQRENATLKKQLKEKSIQKNVETEEKEEETVELRKKRDAQLEEEAQRLKEEVEKLRVEMEKLEESQKHWEERKEEDVKEEEEDEEKKKEREEDKRREEVEKIRREHKKEMQSLVSEYSSAQTHLQARIVALENELREREERCRRREPRCDDLQLGRLQERLSERDQLIKRLVEERHQLQLHPPVAGDSSTLRLRDSKSRPGSVTPTMRRKRVESPPRVTSIPSAGTYDRSIFLPQSSSSSSSSPS |
| RRRRRTRINITY_DN57594_c0_g1::TRINITY_DN57594_c0_g1_i1::g.88869::m.88869 | NA | NA | 16.2 | SATDDPRKLVASYKMPAG**DAKLQVEVLT**FISIVQEVPAMEADEPIWVLTLESKKGVKRHHFSNLGAVSKCYIRKQVGYRLVLVDFSQDLVGMVMAESDLPGCEKVFVGFFLESSLEQVRKSVTKKDNCHSAQKQVQKTSLGLHPGCKLSSALLRHVIIDAYRRIPSTFHTYFPVNLAYHKFLAEDTVEGTCFYLAMQMPRSCMHTLVEKRASSYEDDGITTNLSKHLAGASSLDINIGLQDCLEQLEDVMKTKPPPHRRLLALQPFKRYIHNATAINALLMFEEVLKNSDRYQYIYCGQPMMTEKDLTFSLKLQDLRLAGGSFRQARLNKAISHLNLVAQHIEDIPHVPDVPPLEEASFMKEPAKIMSQAHDYSLKVCSRIVSRGFWESLIKGEPTIKWIVSFTLRDTLPNLSCLEECLLRPLMPIVKQVMYVSTARQSAVADLTNNEEVFYSVDAIHVGVEFNGDPLQKCSLADDLDRATAPDITFICENRLDKRQSMEEPPITWPLNKPLCDLVEDSFESFDVDYETLIGETEPEIEGAQGLTKALRGEAFNNDAPWDTIRCIFLTSAYDGPRSTFDEPCDSLPVNIRPVRHDVPSFMAFPKDPLFKLFGTAARSHKREVIYVVKATKQLIEGNPRPAPPDELQGGQETCHDEVIVDPKAAHEAKKQMKQVMHEKQAESESAGECDTDPRVVKWQEQPLVQVVVVDGNLARNRSVIGDLFIDWTDDPSPIFAEHYKKPNIRLQGQILEGRKLGHSVEESTMYPEFVQKRPGRGGKNKRKDAPSNTTSARPQQMKKDKGHPSMPRSLQTDVKTGERSKEEEPHKPLNKKKSKATKDQDQGSAGAKKPSQPRGVLMDAGKSDKNKKQQKQSKNMENPTLGALEEGSEVDGDHSTATDRRQGRRQRDPMGQKRECDQLTNRSSLKEMYLSLSTEEAKNRLSSPSFDSSDSFDNPVQDRKRQPINLRDSQAKLLTSPGNGERQCSLDKAYQELYLSFKSSRHQSLLRAYADKQPPTSSQNQSRKPERSQNAKKSQRPSDMHCGKCSAAESIGTSTTYSLQRESSFALRSVAANVEET |
| TRINITY_DN53444_c0_g1::TRINITY_DN53444_c0_g1_i3::g.106400::m.106400 | gi\|734642277\|ref\|XP_010750064.1\| | PREDICTED: lysine-specific demethylase 4C-like isoform X4 [Larimichthys crocea] | 7.7 | MAGAEVFTPANPTCKIMTFRPTMEEFRDFNQYLVYMESQGAHRAGLAKVIPPKGWKPRRTYDDIDDLMIDAPIQQMVAGQSGLFTQYNIQKKPLSVQEFRRLANSDMYCTPRYLNYEDLERKYWKNLTFVSPIYGADVSGSLYDEDIEEWNIGHLNSVLDVIEEDCGVSIQGVNTPYLYFGMWKTTFSWHTEDMDLYSINYLHFGEPKSWYAIPPEHGKRLERLATGFFPNSFKGCEAFLRHKMTLISPSILKKYGIPFDKITQEAGEFMITFPYGYHAGFNHGFNCAESTNFATLRWIDYGKVATQCTCSKDMVKISMEPFVKRFQPDRYPNWMVGKDSTPIDHTHPTPSTTPELQSWLQGRHKTKPTNKGSSHSRMRSKRLRTTEEPVGLDGSSTLSSSKRKGLGGPSGPKVRRSVTGTTCREEKEKKKEVEPKHNLNENSLQLPDPCRQMCVVKVNRVESNSLGFSNKRPSHSSSCSPSPSPASPVKNDVKAETVAHDDPQHLSGSGATSRTPEGQHPEATQMHPGINQSRCCSPDPQDLSTSQDCSGLSGVTHLEANKPNPSSHTTSTSAETEMNLCPPDCEAQTSSSSCREDTLSLTPNTDISMDTGTENYTNDTAKAEKMDTLADRKHITPHSTTEALYDLKAEPVEGDRRTSCGSFSPSGHTCSLEKSSTEENLFPPLLQRNAADMPQLTPEPADKVGICPLPPVLTQEMPSLTPADDVLTDEPKSRLRSHHVAPVLQRETPTGSVSAGGAKRGEGENELMTSKESPTVEHANNWHFDSPYNCKGAAASSLEGNTAHLAANAMHKITSGGAHKMLIMPDIAVQKGHTKL**ENLTPGQSSATS**GQLVQSGAPLTQQQSNSVELASTDDSKESKERDSTFSVSSAGSILTGASDNNPPAPSPPLCTQSDSHSALQLNHHTTFSPSHCSSQNPYMEPKSFSSSIWKNFSSQSPAVLIQSLHPELPSDFTHDPLPYTMWTEPQCKEVTDLEDPDQVLRESENQEEEGGPLTWAQLEPTSLVSVGAVEPLGLCGDYGLHRGEEEGAEGLSLCRELGRQREAGESLHSDAAVSPLGTREREQDGVSDMEIGGSDGEEAEQQSSVKGESSSDSSDEEEEEEEEENDTSNYECDESGLEPGEVCAYPALSVKRTTKSWRHPLRKPTARAVPTAVKQQATSDDEPPEASLAEEEEQEMEVWAKPLVYLWQNRKSCFTAEREYNAQAATMQPCCAVCTLFMPYYQPEDKAEDNRPATAKDTSKSSSPTEGPTRPCGGLRRTKPLIPEICFSFREQNCPPTPTNPLLQEDGTSPLLYCQGCCLQVHASCYGVAADDVSEQWSCDRCTEGSFTAECCLCNLRGGALKKTQNDKWAHVMCAIALPEARFSDEAKRSPIDTSRIPMQRYKLKCIYCRKRCSGKRQAGACIQCSCGRCPTSFHVTCAHAAGVIMEPDDWPYVVSITCHRHQSRSSSAKQRACQASISLGQTVISKHKNLRYYSSKVTQITSQVFYEVMFDDGSFSNDTYPEDIVSRDCANLGPPEVGEAVQVKWPDGLFYGAKYLGSNVSYMYQVEFEDGSQVLAKREDVYTLDEDLPKKSTASSMRFQDAFFTTQGERKRQRTPNSRFQKDYVALPGLRTTAKSTWEQRSHKGK |
| RRRRRTRINITY_DN39870_c0_g2::TRINITY_DN39870_c0_g2_i1::g.104826::m.104826 | NA | NA | 31.4 | RALQLRPAPLSAHQLQKRAHKTYGTENKFNNVAQRLFKNAILNDPSVDSQKCTYCIHEESDLLATRICDDCYSNGCCPIVVADTMLDNCIPCLLEDPIPDTDDESSSQDHPVFPPREKKGQAYAEADIAPIAYEGTSTLMAGKTGPEAKVMFSQPIGKSIRVPKPGEVSKDQVMLMPCQRIYHGAKGCRFCTYHAPPPGVAKKSYHIPDYEHNSQSMMAKIKDEESANADVLNATKALQALSMSPSSDTPRSSGVVATDSRDVIFTRGAPKVG**GLPTRRVIVS**SHKPIHAEDDTYEERTQANTIQLDCDTAKLRERAMIQRKLEN |
| TRINITY_DN112195_c0_g1::TRINITY_DN112195_c0_g1_i1::g.135113::m.135113 | gi\|548418414\|ref\|XP_005742228.1\| | PREDICTED: immunoglobulin superfamily member 10 [Pundamilia nyererei] | 25.3 | GEAVMVVQLEVTSMLRRPIFRNPYNERIVSRIGKTTVLNCSADGNPMPEIIWTLPNGTRFISGLDHGSRHHLGTDGSLIIYSSHKDDSGKYRCGAKNIMGYIEKLIILDIGQKPYILTRPRGIIRSVSGEPLFLHCLSDGSPRPRIYWTIPGGHTFTRPQVLGRYQLLENGTLVVQDTTLHDRGNYICRARNDAGEALLTVPVLIIAYPPRITRGPPPSVKAVTGTPIQLHCAANGVPKPEITWELPDHSVLSTAEQGRPTGSELLHPQGTLIIQRPTVSDSGTYKC**LAKNHLGT**DSKVTYVHVL |
| TRINITY_DN43618_c0_g1::TRINITY_DN43618_c0_g1_i1::g.104607::m.104607 | NA | NA | 22.3 | ALLDVEHRGSDGGHRLGRLSGWHVEDGAALVHGEHVGARADVDAGVLGLDVLDGQDAVEVHGPVGKLPVTPAGPHQGVGRGLALCSTDEADCRPHPDSLRFGLLNRHLNGFSLDACDNYLSAAPVGAALVSA**GADVDAGVTGLDVGEVQ**LCSLSASVCSAGDGPSVLSGPVEIVRRRSGHLAAQGDGAALGRQDPLWINLHHQGGCSIVRTTLLPLLWVSNVSRSTTFTVLAGVAGLAGRALVASVSEALTGGRELVVGVSVGAGAGLAILRSANSRVAEEAGGALLAEFTLSVMQAARADAGLWVAGVRVAVTFTQLTVTQV |
| TRINITY_DN58550_c9_g1::TRINITY_DN58550_c9_g1_i1::g.62529::m.62529 | gi\|1007751297\|ref\|XP_015815187.1\| | PREDICTED: protein timeless homolog [Nothobranchius furzeri] | 40.4 | EEEDGPRSPAGDPQEEDEKEEKEKEEEEKE**EVSGEQRAQ**ALRALLLARKRKQPPGSEPTAPVSDSTPSEKTHSPAERSQSRKTSAKRSRSRVLEDDDDDEEEEKEKEKEDGDSSSVMDVDGDADSDGELNTSAPVK |
| TRINITY_DN54061_c0_g1::TRINITY_DN54061_c0_g1_i2::g.17164::m.17164 | gi\|808865870\|gb\|KKF16666.1\| | UPF0364 protein [Larimichthys crocea] | 18.1 | PSPSATMMAADQTVLGVPPSLSGKVVGSFAYLTIRDRLPTILTKVIDTIHRNKNKFFEEYGEEGIQAEKQTISLLSKMRNELQTDKPILPLTDALQDTESWNQYLQRHRGSHGDEESVSWFKSPWLYVECYMYRRVHEALWLNPPISDYDVFNEGKTQSFFESQQAVMTLCTYLGGINKGMEEMSKNQLLEHFNKLLLVSLWGNKCDLSISAGQDNSQKTSPIDSLSSLQPFILVDDSDMVWSTLISSQRPGQSEKITGGRVDIVLDNAGFELVTDLVLADFLVSSGLAREIHFHGKSIPWFVSDVTANDFQWTIRQVMAANHKWMSKSGFQWQNYVKEGVWSYHDHPFWTQPHEFCDMPADAPDLYATLQGADLVLF**KGDLNYRK**LTGDRDWDHTVGFDTALRGFGPAPLCSLRTLKANIQVGLQQGQGEKLSSQDPDWMTSGKYAVIQFNCRKSEQ |
| TRINITY_DN88080_c0_g1::TRINITY_DN88080_c0_g1_i1::g.27057::m.27057 | gi\|808858922\|gb\|KKF11228.1\| | Tyrosine-protein phosphatase non-receptor type 13 [Larimichthys crocea] | 29.2 | SRQAWDRSVYQTPSSNLGLGHYGSTGHLDDTVHSTFYSPNQSMTRSDLSKRHPSSPVAADLESSPLPMVSSPTAPS**PDPLPPPL**PLPLNLTMAGNGQDIEEFVPEVELKVSLVKSEKGSLGFTLTKGNDHGCYIHDIVQDPAKGDGRLRPGDRMIMVNTTDVSNMGHT |
| TRINITY_DN28035_c0_g1::TRINITY_DN28035_c0_g1_i1::g.5665::m.5665 | gi\|583968584\|ref\|XP_006779751.1\| | PREDICTED: EMILIN-1-like [Neolamprologus brichardi] | 11.6 | LRPSYSLYTGGHAHSARATSRHRNWCAFVVTKTVSCVVEDGVETYVKPDYHPCNWGSGQCSRVVVYRTYMRPRYKVAYKMVTEMEWKCCHGYSGEDCTDGPVGGAGTQIASTRPQPRPGQGGGTSHGQGGGGSASGSSGGHGGGADREKMRQLEEK**IESLTKNLQD**LRSTLEQESNKPGLSGGRGGGGRNPADAAQPEIKETIHSIQAKLDQLDNRTQAHDKTLVSINNHLVNGKGNDMDRGFSGGVNSLKEEILRELERRVSLSCSSCQAGVEDLRRQQEEDRERIRALEKQLNAMDVRYLQDLGGLRRDVTRLQGCCDTVDDLQYRVTDAERKISSASENIDILQNHVDDLRGGEEGGGGRRKEGGKGGGRKRRKKEGR |
| TRINITY_DN25797_c0_g1::TRINITY_DN25797_c0_g1_i1::g.108128::m.108128 | gi\|808877642\|gb\|KKF26066.1\| | A-kinase anchor protein 13 [Larimichthys crocea] | 20.6 | PDWKEGGVRGGREEEVETTYSRQNCEAADIEETGEEKPAGEERGAKGRKKRRKKKGRRGGGAEHKLSSSSSVESQSQSETLVHREPAATGLGPQSPTEPEAQDTTKREPLESEPACREEAEEEAMHLPSQTETHNRDTHGPVCDVTASESGYAGTTDHGRTDLTEASETKESSEDLSQVLNLDSASDLSQ**TVFMETET**TAGPQGGKETVESSSSPVQQAAGDKETKELAAAGEDAECVDLTGLLESECPEELPVRHSDPTGLAEERSPVEFP |
| TRINITY_DN25047_c0_g1::TRINITY_DN25047_c0_g1_i1::g.29679::m.29679 | gi\|808869080\|gb\|KKF19258.1\| | Ral GTPase-activating protein subunit beta [Larimichthys crocea] | 7.8 | MSISLAALELLAGLAKVKVGVDSADRKRAVSSICGYIVYQCSRPAPLQSRDLHSMIVAAFQFLCVWLTEHPDMLDEKDCLVEVLEIVELGISGSKSRQEQEVRHKGEKEHNPASMRVKDAAEATLSCIMQVLGAFPSPSGPASTCSLLNEDTLIRYARLSATGASNFRYFVLDNSVILAMLEQPLGNEQNPSPSVTVLIRGTAGRHAWTMQLFHQPRGARANQRQVFVPEGRPMPNNDVGIKYNVKQRPFPEEVDKIPLVKADVSIPDLDDIVSKELEVQHDKLRILMHKQIEYENALERHSEEIWKSKPYPDPQTDCKPPPPSQEFQTARLFLSHFGFLSLEALKEPNNSRLPPHLIGLESSLPGFFDDISYLDLLPCRPFDTVFIFYVRAGQKSSHEILRNVESSSSVQPHFLEFLLSLGWPVDVGRHPGWTGHLDTSWSLNSCSDSNDIQQTADEATTPEDTGGSVFNGEKKVLYYADALTEIAFVVPSLTENSEESSVHSDSTVEAD**TNADIMPSL**HKQPNLTLELFPNHSENLESAKKLSPLVKTKRSSTGKSFPALGPETKVFVVWVERFDDIENFPLTDLLAETSTGLEASMSNSTSCRSGLLEKDVPLIFIHPLKTGLFRIRLHGAVGKFGMVIPLVDGMVVSRRALGFLVRQTVINVCRRKRLESDLYNPPHVRRKQKITEIVQRYRNKQLEPEFYTSL |
| RRRRRTRINITY_DN53427_c0_g1::TRINITY_DN53427_c0_g1_i3::g.106537::m.106537 | NA | NA | 7.4 | SQQGGWQSDVPRRLLEEVHRAVDMTDDLDFDGDADLMSDSMSPYIGYTHPHNVPAPSAGHDMYTPNGSAPDPNATAFEPVVQKIQPKVYGDVAKSLPPTYYKSFVEDKPRDPYLFLLHNLDSIRDALSRISFDKTTYPMLNWVMREGAKNPNEAVWAITIGGIESDSFRLLFTGNPKSMLMDQAQQKNVFGLIAGDNWHPKLHKKTLEMVGDFWQWFTFNRGPLSERNFQSWTMTMNRYDDPNNSSISFAKQALFVLNEESLGRNSQVEAKYKMNIADCLQPWLVKDPVLFPVRGPEAFANDWLVTATANNDQSGHVIVVVPLSLTKVQFVLENGGVSFQSEFLITFKEETVSEAGRRDSRKIRKLSMNRFHASLTGTTQHYEMVCNNNLIEGSSDNRTNENKLLAKAQQESIITAKVQPPNMHVNLKGGVLLRVTAAFKTQTKLVQPPQKEIIFTSTVLASIIDTITSNLENLLEEIPGPIPLQQRLHEARRIQQRNQWITEALKECWSQLIDLGGEPPGGNGALQQRRKWQILEDDLIITQQKRLLQLTKQHKEALDLRYKQLTNAERTLWAEVTARKSLLAPERLQRDAPP**LTALSTLQAQ**IRLSEQYQIIFYEQNHQLKRLDNETDQTMLRLEEFAQNIQQYKQSMSDLMGGVPSSSNTAERVLRQETYLIHKICRVLELPCRDYTSKLQNAYHGLKIKLLFGDEGVQHEAKNQLEQILSDLLRKAKFEEQPNELDIVDWLQGEIWQSLYHRVEIPFHQGYLSQMQHLADGQLQQAQIWVAM |
| RRRRRTRINITY_DN50106_c0_g1::TRINITY_DN50106_c0_g1_i2::g.72987::m.72987 | NA | NA | 33.5 | LGCLPGLWMPPPPFETMHPALPAPKMPMRGPGCPFFLLLPPPPAFTPPTPPPALSNFSMFAFLLFFGAPPSPGPTAAPTPPPPLPPPVPPPFAFPTLAAPPSGSLETVMDGTSERSDGCE**YVPSTDPVTD**VVAFEGGRSLLGVVLCISGGTLWMSPWCCLQTPM |
| TRINITY_DN52923_c0_g1::TRINITY_DN52923_c0_g1_i2::g.15490::m.15490 | gi\|499024963\|ref\|XP_004563236.1\| | PREDICTED: translation factor GUF1, mitochondrial isoform X1 [Maylandia zebra] | 8.2 | MSAALVLSPLLRRGLHFKMYRKKILNYSWTCTVLRHRWMKGLPVFSSGFRMVSSSTQTDKQATMDLSKFPADKIRNFCIIAHIDHGKSTLADRLLEMTGAIAKTEKNKQVLDKLQVERERGITVKAQTASLFYSHQGEQYLLNLIDTPGHVDFSYEVSRSISACQGVLLIVDANQGIQAQTVANFYLAFEAQLTIIPVINKIDLKNADPERVESQIEKVFDLPREECIRISAKQGTNVEMVLQAVVDRIPPPVASIEDPFKALVFDSNFDHYRGVVANIAVFGGRVRKGDKIVSAHLGKTYEVNELGLLRPDEHPTQKLFAGQVGYIIAGMKDVKEAQIGDTLYHQEQPVKALPGFKPAKAMVFAGMYPMDQSEYPGLRSAIERLTLNDSSVTVQRDSSLALGAGWRLGFLGLLHMEVFNQRLEQEYNASVIVTAPT**VPYKAVLS**SAKLIKEHGSEEITIVNPAQFPERSVVSQYLEPMVLGTILAPDTYTGKIMTLCLNRRAVQKNMVYIDDQRVMMKYIFPLNEIVVDFYDLLKSMSSGYASFDYENAGYQAADLIKMDILLNGRPVEELTTIVHRDRAYTSGKAMCERLKDSIPRQMFEIAVQAAIGSKVIARETIKAYRKNVLAKCYGGDITRKMKLLKRQAEGKKKMRRIGNIDVPKDAFISVLKRKDK |
| TRINITY_DN20852_c0_g1::TRINITY_DN20852_c0_g1_i1::g.28843::m.28843 | gi\|734596266\|ref\|XP_010736015.1\| | PREDICTED: general transcription factor II-I repeat domain-containing protein 1 [Larimichthys crocea] | 10.4 | FKRPCTYGVPKLKRILDERHGIRFIVKRMFDERIFTAAGKIAREEGKHDLGSTPEDSFPDNLSLPPTAAELVSNPHSSRSTSACVSPLADCEAGPSGDCIPLKRIKTEPPDGEIIQVTVPAEALSPSPASQSIRRSSEAGSLVEDIGEMILQLRRQVESLFSIKYADALGLPEPAKVPYSKFQMYPEDLYVTGLPEGISLRRPNCFGAAKLRKILTASSQIKFFIKRPELLTEQVKQEMPSIPVCDSEPDTKDAAPATEDTAAVSKRPGFSECLESKLSRIDLANTLREQVQDLFNRKYGEALGIKYPVQVPYKRIKNNPDSVIIEGLPPGIPFRKPCTFGSQNLERILAVADKISFTITRPFQGLIPKPAPRRVTLLKKAYASISDDDDINRMGEKVVLREQVKELFNKKYG**EALGLDRSVMV**PYKLIRGSPESVEVSGLPDDIPFRNPNTYDIVCLEKILQAMDKIKFNIKSQLQPFAEICSQPCNTVGTDASTNRRKRKRVQESNRAPASSDLGISTNQIPVMQWPMYMVDYSGVNVQVPGKVNY |
| TRINITY_DN16805_c0_g1::TRINITY_DN16805_c0_g1_i1::g.2913::m.2913 | gi\|657531755\|ref\|XP_008302457.1\| | PREDICTED: centromere protein F-like isoform X2 [Stegastes partitus] | 10.9 | GKESALLSQTEELQKCKQELDALRAKTEVKEREERSAETTALQPNEEKHCMTVAVCDSADQPKTVTPEAETQTPVSSEEDSELFGRPGSVDSDKTESNHDSACVLLEETECGQDGAAAAAADVVAELLALKQENQQLKQKIAGFTVGSSSLASQTVSGNQEDPFKQSQNAGSAVLPCLTEQRPTSEQSDITAEGHESRLQKERRRRDDEGEGGDLKREDKRTAGAEEELDEISQLHVSHLEEQVVALQTKVRLLSEKTQQQAEELLLWRLASQPPPTSDRSPPDRDDQDQSSAVRHTQVVQESSGNITVIREDELLLSTSSNKLQGRMLFSSLQHSNLPEPKSFHLSKKTAALQEHNQDAAKIDKESEKENQE**INVSQRSEVT**CPAHHRDKRDTELSQISSEKTSQQQQQQQHVTKDLQKVSAPNKTKTTECRPGNPEAKPNASRSTNEINTTNDSSDRPARTEMKSVCSQTEETFCSR |
| TRINITY_DN7223_c0_g1::TRINITY_DN7223_c0_g1_i1::g.96790::m.96790 | gi\|657566147\|ref\|XP_008286402.1\| | PREDICTED: lysine--tRNA ligase isoform X1 [Stegastes partitus] | 12.9 | EVFIIGKNFLKDTKVIFQENVSDEKSWKAEAEIDMELFHQNHLIVKVPPYQNQAITSAVCVGIYVVTNAGRSHDVQPFTYTPTDPVKQDVPVKKEMPSPVKTCTFDEQIKVLDGAL**MPSMLPLV**KREDVTPMEVTSNLQSAGVFKTADLCPAQQNPDMSAGHLNKSRPFSNNLSQPAGDPDKSQTPVFTNPEPLSTIQKQDIAATGSFSVPADSLLQQGSQQFLLESREGLRQERPGSGSGAVGRLCGEPPPQQQQ |
| TRINITY_DN52558_c0_g1::TRINITY_DN52558_c0_g1_i1::g.58874::m.58874 | gi\|1012506563\|gb\|JAQ20959.1\| | hypothetical protein [Fundulus heteroclitus] | 25.4 | AQLAVAVLTADIPHHVSSRQHDSVLDLAVLQIHHLVEQESSARGPGEARGDELSSVGQDGVTVGAG**EEASPTDVI**QEDTPHFTDHQVKTTKRLLRSKIFTKKHRRFSPKCFYPSFGLNCRNN |
| TRINITY_DN48857_c0_g1::TRINITY_DN48857_c0_g1_i1::g.72616::m.72616 | NA | NA | 39.5 | MITTRMIIPALSSSLLPSFCLGLVVAALVVLAVFVVEVWLSGGLRGVGVVKSVVGLLVVVPPPFSLVVLGDMLMLVVLILL**VERELLVLG**LALVVILEGIWSLEILSPLPPVTSDTSEY |
| TRINITY_DN130679_c0_g1::TRINITY_DN130679_c0_g1_i1::g.132129::m.132129 | gi\|657565866\|ref\|XP_008286253.1\| | PREDICTED: teneurin-3-like [Stegastes partitus] | 22.3 | LKTLTAQGQELVLFSYHGNSGLLATKSIQIGWTTFYDYDSEGRLTNVTFPTGVVTNLHGDMSSGAVAVDIETSGRDEDVSITTNL**SSVDSFYTLVQ**DQLRNSYQVGNDHSLR |
| TRINITY_DN119168_c0_g1::TRINITY_DN119168_c0_g1_i1::g.135215::m.135215 | gi\|657557352\|ref\|XP_008282872.1\| | PREDICTED: tubulin--tyrosine ligase [Stegastes partitus] | 10.4 | GIIMTAPMYTFVTRDDNSTVYAEVSKILVSTGKWKRLKRDNPRFNLMLGERNRLPFGRLGHEPGLVQLVNYYRGADKLCRKASLVKLIKTSPELSDSSNWFPESYIIYPTNLNTPVAPATNGISHLKNNPKTDEREVFLASYHSKKESGEGTVWIAKSSAGAKGAGILISHDANQLLEYIDNQGQVHVIQKYLEKPLLLEPGHRKFDIRSWVLVDHQYNIYLYREGVLRTSSEPYNSSDLQDMTSHLTNHCIQKEHSQNYGRYEEGNEMFFDEFRLYLLNTHNVTLETTILPQIKQII**KSCLSCIEPAIST**KHLSYQSFQLFGFDFMVDESFKVWLIEINGAPACAQKLYSELCQGIVEVAISSVFTLNSSSDSSSASSSPYSSSPSSTFSTNSCSSPKLRGPLHVGPFTRL |
| TRINITY_DN112730_c0_g1::TRINITY_DN112730_c0_g1_i1::g.133060::m.133060 | gi\|548371092\|ref\|XP_005732622.1\| | PREDICTED: sentrin-specific protease 1-like [Pundamilia nyererei] | 14.4 | SSRPGIQAWSLDTDLSSNRAATLTSAPSPSALQDNSSQDTQSSAHDGDSVIIVNEKKGKKKDSSRVPCFQAELWIKELTSMYDSRARERRRQIEEQEALTAQLLRQRLSEEGQRSPDVEVHVRVPLEKEVPLTPVIEEPKPVEE**KPEFPELT**EEMEADVNRVLRGGSPHEVLSEGFGLSLTRKDLHTLSNLNWLNDEVSAYY |
| RRRRRTRINITY_DN86082_c0_g1::TRINITY_DN86082_c0_g1_i1::g.49114::m.49114 | NA | NA | 11.1 | KILTGDNCHWVIGEVRGDPSEQFWSCLQQFDVPPPNRIQVSGHSVLCHVPQKKSGLGYPNANVNTGILELTQEML**EALPVAAIE**LEDEGGDPRLVLAKGVEYDVVSSHWCYQKNDKEVPVWGPIHGHEDPLPRGNDHKVRHAPIWTEPVTKFDEEVNWQFGKCSRHTHQHKKFRKEAQKNPKRDLRAWL |
| RRRRRTRINITY_DN4314_c0_g2::TRINITY_DN4314_c0_g2_i1::g.743::m.743 | NA | NA | 15.3 | GVGAIVVVIVVVRVCVPFADTASARRDIVSSSVGISWD**ISADGITVIN**IIISSSYVSPPLINGHLPSLSLLPLFLYLPHLHPLLLLLPPLLPHPPLLLLLSFPPPPLLHLEYVRLLLLYLINPRTTEGVGGERVRRRRADGEVGGGGEEE |
| TRINITY_DN98793_c0_g1::TRINITY_DN98793_c0_g1_i1::g.27658::m.27658 | NA | NA | 24.3 | MLRQLGYPISCHPD**ISPCVSADSL**SPHDSPGWALWHPTLWHLGCQRQPEDHQNLGFSCHPWLRSLINHLIRLCLTALLNYSCVGLQWALSARLLGLAIAGFHS |
| TRINITY_DN25823_c0_g1::TRINITY_DN25823_c0_g1_i1::g.67745::m.67745 | gi\|736207514\|ref\|XP_010776255.1\| | PREDICTED: cyclin-dependent kinase-like 2, partial [Notothenia coriiceps] | 30.2 | ATFWGGSSSSINTTMERYESLGLVGEGSYGTVLKCRHRDSGRLVAIKKFMDSDDDKTVKKIAQREIKLLRQLRHGNLVNLLAVWKRRRRW**YLVFEFVE**RTLLDDLE |
| TRINITY_DN136544_c0_g1::TRINITY_DN136544_c0_g1_i1::g.141709::m.141709 | NA | NA | 10.9 | DDRERREECVLVCVEP**AESVLLAPIS**TTRPGHTHTHTHTHTLIHTLIHTYTQIFKLFNERCALDLSHQTDRQTKKLKKNKPQKWTNQKQVQTSQVLLVVFGFFLFFSFLFFSFLFFSFL |
| RRRRRTRINITY_DN58624_c13_g1::TRINITY_DN58624_c13_g1_i1::g.62017::m.62017 | NA | NA | 24.3 | RSVMFRSFLFRCCSIESRVSCIPS**VPLLMWS**SSFDLFCTSVSFVSIWVARSSASPVRSCSSDLIRRTWSSSLWILPSTSLFSASFSSSISCILLFLWLTISEM |
| RRRRRTRINITY_DN56930_c0_g2::TRINITY_DN56930_c0_g2_i1::g.105562::m.105562 | NA | NA | 21.4 | DVNVACRSLFSLPFSSPISVTPSFMYFILCRTLYLHTLSSFSLIFSLQCITFVSIHYLIYFIVNLLIPFCFFSVLSLRSPILLPKKRCQFILASLSIVDHGELSLHVSLSHCTPVSPNLSNFILNM |
| TRINITY_DN15796_c0_g1::TRINITY_DN15796_c0_g1_i1::g.30168::m.30168 | NA | NA | 14.7 | PP**ASDQPVLP**PRRDRGSGRGEREPGLPAAHLRGQGLPAAEGPPRRAVLLQGQAAQVHRDSRSDSDGRRGGRQSGAAQRGGLRPRDEPVEEHGLPALRREQTRAGGVGLHPVFSRRGVSRRLGQQGDVAL |
| RRRRRTRINITY_DN49553_c0_g2::TRINITY_DN49553_c0_g2_i2::g.101002::m.101002 | NA | NA | 15.4 | SVRRASRSSANLAPSATRTGCRTRTSWTRTEQCSWRTAARAGTAMKGPAVSAVCLRAMMERVSRSVPWPS**TASPAASVT**RTGTATRITCSRQILALPADASQVPTPVSSRLASPACM |
| RRRRRTRINITY_DN43735_c0_g2::TRINITY_DN43735_c0_g2_i1::g.107229::m.107229 | NA | NA | 12.3 | KYTCFFIFIVIARSDRMVCSSILFSGSFGISRSFVQGRRSLTKVVLLPRCWLGSSNARPLTGELSSCIYVHISAVRFSSVLRTFSTYGSAL**FFPVCKLK**AMTKGSGLPFVYLNFSMPLVTDPRFPASSEM |
| RRRRRTRINITY_DN105124_c0_g1::TRINITY_DN105124_c0_g1_i1::g.133609::m.133609 | NA | NA | 12.9 | PGTIPPGHIATMAPAFTRYLSHNFVPSRINAMVADLLPQCLYQQKTAVPPPPPTPVAMDNKLKELAIECKQLTRLPCRT**NPDTLINLL**SKMKSLDKKRDENKDIKNIMRRLPEIYKSLQKLKEMYQQDELSTAGAPSMVSSPNGSSL |
| TRINITY_DN6278_c0_g2::TRINITY_DN6278_c0_g2_i1::g.53135::m.53135 | gi\|657583996\|ref\|XP_008296132.1\| | PREDICTED: GTPase IMAP family member 8-like [Stegastes partitus] | 9.1 | MVMVTKEMAPEVGGGSNGADNVFKNTCIKLTGPSAEKLGEAFYGPGIVVLQEIKGVEKGAAYAEDPENSKEKEKELIINRIVRGALMQPPPGCGGEQVTEREGAMAEVKP**APVPPTRIIAG** |
| TRINITY_DN10297_c0_g1::TRINITY_DN10297_c0_g1_i1::g.28240::m.28240 | NA | NA | 7.9 | VGHLKSINKLVSSERGDERCRRCSSNKRAWTSQPRVIPVSRQSVPSRGPFRPVLISCFIHAVAGAQRGLSSEWRAKLS**HQQLAGTTGT**TRLTHRVRAWWWSRTVPAWSPSLIMAVLLASRRAPSPG |
| RRRRRTRINITY_DN57974_c2_g3::TRINITY_DN57974_c2_g3_i1::g.44135::m.44135 | NA | NA | 59 | VSRISIHVIATDSGLENSLKLTYTGADEEKVGTIILTTLDETTEIHFRGGATVEITRGGRSWEIHKADGSFACAVTLVKGPEISIDEPLAEIRPASGIARFSSGEMHSAMETLSSSSMAYSESAMSSMSSMSMTEFKMEAAMSSFSSSEMMSSMMAQQSSSSFSSAEEMTHVEKKMMMSSSMASSAEKHSMMMSGKVALQQERYATVKMRISQFVQGTFSFSSKSVGGESTMECTYEGQDSDALDHILLSCSGESYHMEFHSSQSLRRGNVFWVVERVSSECVCSMRVSGEHAEATVQSLVKPPKPIPSKVTPPSVVRKPSMIPPPSKMRPPSKLGEPEPSKLARPSKIGSPEPSKIGRPTKIGVPEPSKLGRPTKLGAPEPSKLGRPSKVGEPEPSKVRDPSKIPPPSKLRPEPSKFGRPSKIGEPEPSKVGPAPSKLGPAPSTPEPSKVSRPSKIGEPEPSKISPTPSKVSPVPSTVSPEPSKVSPTPSKISPEPSKISPEPSKVSPVPSTISPTPSTVSPEPSKVSPTPSKVSPEPSKVAKVEEKPAARRITLTFHAEQRGASNEVIVTYSGEHSSTVSSIELSSKYQTTTVQVHHSSVLTRSEHVWTVTPAPEGDIDCSFRATEGESVTVSQPKTLIRASLSPKVKHVSVEEAVAAASSKVKTESTAHKGTLSFKDSSYSSGSSLYETSAYKVSSDYREASSYRESSAAYRERTVVERTETLKSKTEEVEFHTQSAYGAKFHTTHYHDIRTVEPVHAKPVDEDRRRSSFTTYGGGSVDLTAYDSAEGKSNSCVCRYTGSDEEQCELITISLVGSLNSFIYKESSESIVTGNHFWQIEAEPKTQINLTFKTDQGLPIRHSRMRVTVRPAHDLSFPTQSVVQYKREAKLSAKRAEYRSMIDFRASREFRSSSRSSREVSRIPSPSRPRTREPTPSRREVIEGEVEVEVSPRARSPRILESVPRMLEIYTPSLSRRRRMFQSVTRKKKTASVEVAEHRVEHRREEHEVARREIRVPSIAPSASFGLELEDELMMKEASARRRSLYDDVPVLLEEDSLDGLSRRRSRFSVESEPSPSRRAPRVTELDDLSLKPIRWRPRYQDTIRPLPMVYFQEWRSLRIRRQRKQVIREMREPIEYQDRLKKYWKMDSLPVFQKITKDEDLVTPDHHLPEPIEYPMFIRREEELKKTESKHVTIDFEGEVNKTSVAPKYMEAAFKIAEQATPNLPVLEETAAIHEAMKNTKEIQKQIHRYKEEETKYERRTYTLRDVKLTAQCSVSGTSNTATVKYVGSDELLTERIHLVHYDVDEQIVVVKPGFQLPQGDKQWKLTPAPIGSVRLEFRVSHGETCDVNALLRKFMPRMTEESVPHPTVTLRAQCSDEGFKNHAMVTYEADDSMDLNHIMLQYLGKDSTFTYKSDDDGPKIREGNKLWAVQPEPHVTITVGFRVDEGIYAARNYLPLTFEPPRQLIKTKDVRRRLKKISRSIYHQRISRVTEVFLEGYASDEGDDSVVKCRYVGDDSEEIDKVYISAFGNSYTIEYKPSPHLQRNGFYWSVQTSKDYNSISCTFRVHGGEEASGHMVPGARLGQYETGIKVKGVALGRQNKFAGGYAIRASSVITDIRKALYQYYRRHRLTRIAKNSVLEIKMRLWPHELAEHATMRLKRDKVLLRDVFDMAELSIDKFAESDFIYECSSINEIMKTISEAAFPNLGSLLVYAMVGVSWMDTATTVLDHRHVEPACYEPATFLMRINEGPVLLRAQGMEIIKIITSKRTTYVINDPRVDFHGYNHSHLFKLSSCVQRLYRVIEQETLEFNSTGLREFIDVGSIFEFILIIEEMSEFYEHLYIINKHRAVNLAEIERLVLERDTGRVKIFKAMFTKKTAIEVCRHVAGFQCRALEESITYKSSLEKVKYFLAEEKTIERTEDVEEDYNRIVSTDEKTTIPETPHSPHSLGFQNEAIVRFQYKTKGFINIITISCDTTQGARIWRDATTPCKEVIYGIIDSGGDNAPPEWTLTISDRGINSVVLAKPVDPIDAVNVEITQKDMGFRNKATIVYYGTDKRQVGKHFVLSTFSRTTIVQHHASNSLIEQGKQWTVAPEPKGSIPIKVTIHDGRAAHVAGMGQLEKPLYIKAPVEVDLNATTSISGTSNTARVQYVTADNENAAAIVLQYYGGKFEQAKIKTGDALIEKGGRYWKVVPKPYGVVKTVFTANAQYKVTMDRIEERFAPARPAQESKPIIPASIESWDSEGGMNECKVRFEYEFGEILGKIEYNTEAIKKTTVSMWKNQKKERKELYYGTIKAGGDSSPPKWCVVCSDNTSSMIFPQQPIGPKEFPSKIILVSPIELPESVGYQNQASVRFYYGASETLNPVRCTNGSVSSSVLHWLEGEKAERKEVIYNTIMSGGDDKPPKWSIIVSSKLLGEVVMPGEPTCPKDQIEVRLSTDVTGFKNSLQVKYRGANTKRQVNRVVLHTYSETNEIIVNESVNLPKKGYFWMIQPIPRGIYVVHLRLSTGAGAFYKDKLPFGPHFQPAAQLRLKGSTEIEGAENIARCTYVGEDEQEETLVSLSHNRGDSSMKYKRSQILEKGYRYWKIEPLPRGIIGCTMTGSEGLKTTVDKIDEKLAPAEGVSLKTKIGMPTRRPRSLGTENEAFIRFEYETAEMLGGMTFQRDKSREKNSKKWASEGAQRFEIWYGLIEKGGDHEPRLWHITICDKTVSIIDIEGPQSPKDFPDKCVVSESAPGPESQGIDNQAVVRFMYEADPILGTVNYMTTTIHTKNHRVWKETSVERREVYYGTVRSGGDDRPRAWSLAVTTKTVEMIEPPNSPPEPPCLPTKPTVAEDSKLSRSVGFQNEASIKFHYQVNEKLGKVVLSTETVRADVTCWAAKPVERREVIYGLIDSGGDDSPSRWSVRVSRAQIDDFELPGEPVDPRGIVKVKIYVAKRGCKNELVLDYTGTDDKHAEKIVLETIDDHTAIMARHSIDRGDKTWKCTPIPKGKIPVSLHIVGGQRVVYGEEYKHDLEYDPYGLMEQVKVVGPIEAPEGAGGANCAIVRFKYEAGQPMHTLTYECMKTPTTNCLTWEVQDVKQMEILYGTIVAGGEEEPPLWALSVSTRTTDTVRPEVPCGPPEIPDMAVTIESSESPKGAGRSNIATIRHEYELGEILGSSRYRTMTVPNKNIKMWRDSHAEKREIIYGAITSGGNDEPPQWTLTMGEHTVDLVEPRSPPGPITKPSKCEVVESQLFGGVGFRNMAAARFVYETGETLRTFKFCPRTVSESITIWGPRHADRQEVVYGGVNAGGDKEPPEWSVTVSVGDTDEIKMPGVPIGPKDLIIVKIVETAMGIANKVELTYDGTDNITTERIVLHTYKGNSDIKIRDLSDKLKVGAFSWSCEPHPKAKIPINLDIPKGRHVHVIKQPINTMDIKPMVKIDQITVPTVTDMPESAGRSNTARLRFLYQDNEMLPQITHQSVSSKVTAVTSWRDTGVKCAEITYGTLRSGGEHLPREWHLTVSSKTVDIVHLNLPVSPVECVLVAEATECPEGIGYINEPLVRFFYHMGEELGTIRFLTKRCETTVTKYSKMNADRKEIIYNSVHAGGDISPVDWTIVVSEKTYDKVKVSGPPGPTDYVKVLITVTKKGSPNEAELVYKGSDYRDVKEVILSTYSDTTEIFGRSQLEVGTKRWAVVPTPRGTVSVFLRLTGGARLCVTKKLDDALELDPMEIRELPEVEASPDSFEGTGNNNIAAVRFRYKKNAALGAAVYENTKLAKTTARYWQESGEELIEVVYGTVDSGGDYMPRTWIMSISHKTTDTIRPMSPPAPTYTPDKCLIYPSTDSPHSDGAANVATVRFQYESGELLGTIKLRTDYITYTRNVRTWRTSSKERKEVIYNKIESGGDSEPKMWEIIVHEKGVATVEPTSPPSPITYANRAIIPDSELAVGPGFKNVGRVRFIYENNKILRSSVCSLTKCETEMIVWNLRSTERREVIYHTVSDGGDELPIKWSVTCKEETVRTIVIKDAPAGPTDLVKVNVAATKTGSANKVTLIYRGTDYRDCSKIVAMAKTATYTLSYKEGLELIKEGKSWHVTPEPKGGVAGDLVLDSGASVIVTERALKSDLEAKPPSYEDKCTVPGTSESPASHVGAANKAIIRYELTEGEGLNTVTFYNETITTYNCKLWRNDDWEDTSYVKKEVIYGVIKSGGDNFPVTWCLSVSSRSIDTVEPKGPPDVPNLAMVPRSAESAKSLGAINMAYVRFQYELGEILSSVKYHCELCPLKNERVWLITNREKKEIWYGTVQSGGDAYPLDWKISIANASVSVVDPRSPPSPPVFPHEIVVAASEISKSVGFKNEACVRFTYEMYKTLHTVKITTRKVSSNVVQWSASGSERKEVIYNSIDTGGDDAPESWSLTCSESSVGSIEVPGTPPTPRGVVVVTVTFTKVGAANDLTLYYKGSDSRKSDKVVLTTREKTTKISVRDTEKLKMEELKWTVKPAPRGSIPIDITFTNSTKCTILQKSISSLDATPEIRPEKIVVSGHTGQPHSIGADNKAKVRFDYETNEILGEVLMKQSKSEGYVVWRDTGKIRFEVIYGRIRSGGDHEPKLWVLSATSSSTDVVDLRKPPAPEETAIVPETLEHPEGQGYQNVAIVRFLYPIGIDLGTVRIMQRSCKTSVEQWALRSAERRDVIYHHIRAGGDNTPADWMMTLAGRTVDKFTIAGPPGPTDLVKVTFTISKRGSNNELSLTYKGAEYRTCGEITLTSFSETTNVDAMIPLEGDARSWTALPAPKGRFAIHLTVMGGNKVIHTQKFNADIDIEPSTLRDLAQVASAMESAEGEGSGNVASIKFKYLEGPVLGKATYRTHICIQSNVRHWELLSAKCMDINYGIIELGGDFVPKTWELTVSSKTSDIVKIMSPASPTCTPERCKFLRSTSSAPGVGAGNEAMVRFEYEYGETLRTARHRLETIPRKSSVKVWRLSKKERRELIYHKIENGGDTRPRTWGLTISDGTIHTADPTGPADPVTYQMQAIIPDSDLPKGVGFKNVASIRFQYENGKILNTANYSCAECKSTVMAWSIRSTERKEVVYHSIAACGDNEPPNWWVTCKEATIGTFRIPGEVPGPTDQVRVNVDARKTGSSNKAEVTYIGRHNRDADRVFVSSTGIGTTIEIDIMTRKDIEKGDKYWVVQPVPRGTISCGLRVNEGARLTAVQDPSWEIEPAISEDKTMIYGSSNSPPSVTGVANRAIVRFEFRDGPSLGTVTFQTECVDTFNCRLWKGRGQRREVSYAVIKSGGDKKPIAWQLTVSDRTVDICVPTGPPDVPSIALAHKSQESVPSLGANNEAYVRFSYELGEMLGIIRYEKNSIITSNVKQWLLSNRDKKEVHYGVIPSGGDFGPAEWKITMGDSTVNFAVPKGPAGPPKYQHQAVVDTSNLSESVGYKNEARVRFQYQTGKVLNPIKFTTRVVSSCVMKWNAQSTERKEVSYCTIEGGGDEKPEDWSIIISQAKIEDFRIPGVPATPPGLTIVNIDFYNKVVRNDLVLTYQGAHEKKAGRVTIATKNDTTRFRVKESEKLPLNDKLWSIVPRPKGKFPLEIHINQGIRVALEGSPYDILNAEPPIIFDMVTIPGIQSFDSRGKENKAFVKFDMVANEKLGTVECSCRKCTAGLTWTPDEKTSKEIIYETIHSGGDHDPRAWQLTVSSKTSDVMVLQKVAGPTETARVPETTECTDGVGYENEASVRFFYAVDEELGSVKYTTNACEATIVQFAKMTSAKKEVIYNTVPSGGDIRPAEWSLTLKGRTVNKIMLKQCTSPSDLVKLSVTIIKPEGVGNEIELLYKGCDDRTVKPILLTTARETSTITFRNDGAINRDGRRWTVNPAPRGKLPIFVEVDRGAKATYQTTMPVDLDLDAEELIDKAQVPQYMKIDEGKGKCNVAYVRFFYYSGPKLHSVVYECTKVQPNITVWEREESNLQDLIYHTIKSGGDYVPKEWGLTISTKTSDVVRLKSPEGPLDIPDYANYLDSAESYNGAGAKNVACVRYQYENNEVLNSVKQRTDRITGKVVKFWQLSKKERKELYYGDIDSGGDLAPREWIVTMTSKTIMTVKPKSPESPPVFANRAIVPASELPDGIGFKNIGRIRFIYENGQILRPVNVICDVLKESVISWMVRSTERKEVIYHSVMAGGEDAPPSWMITVNDATVKKFQIEGAPPGPKDQILLRVVGCASGLSNKVKVEYTGSNLRTTEKLFLSTHEFTHKFSLQSTPELERGDKFWEITPAPKATINFKLEVNSGAKHKVENLYEQPIEVEPAGYTDVCTAPGATMSPNSVSGVANKAMVRFEYTEDQTLGTVTFRTGTVNTLNAKSWRASVGERKEIIYGTVPSGGDFNPKQWALSISTKTIDSIRLNIPPECADIALVADSVKSFASMGAANEAAVKFEYILGANINTVKFHTARIVMKNVRQWLISNRDKMQLHYGFVHSGGDAKPEKWAVTITERTVATIEPTFPAEPITYPLNVSITPSDIHESIGYRNEAKIRFQYELGTTLKMIKMTCRAVASSVDMWNATTTERKQIIYNTIPSGGEYAPPLWKLSVSDETIDYLEVPGVPASPRDLVVINVYSKTTGSSNSATIDYRGSDGAISERLTIKISDPTVESNFRMTSKLLVNDKSLSVKPLPKGTLPIECKLTSGARVYVTNHPFDKMNIEPSELQDKLLVPNLMEKAESFGAHNEARVRFFYEANERLGSVTLHISKTVGCRVWKEQGNERAEVLYSTIRSGGDHEPKAWALSVSEKSVDTVGLKPPPTPKETIKTGEKTEAPVGVGYENEGTVRFYYVEGEQLDTLKYTLRHCTDTLRTWGKKSAERIDVVYNKVPGGGENEPTDWTVTASTKTVDKVEIHTPPGPSDLVRVSLTLSSTGAVNSLTVTYKGSDNRTAKELTISTFTDTTDIVARVRLDVDPKTWSVSPIPKGRFPVTMTFSSGDKVILTKRMENSIDFVPEDEREKAIQRDSEPSPESVGIKNISKVVFVYEAGPTLGVVTFETNKTKQVGVFWEDDLSKRYEVRYGTVPAGGDFLPKNWSLTVTTKTSDIIKPKAPPSPLFQPDEAKTLPCPNSPASLGAANEAFVRYEYECGDRLRSAKVRLDYVPKKNVRCWRLGTKERKEIVYG**AISSGGDSVPR**EWCITMAESTVATVQVNTPAASVTFPDMATAPESELAEGDGYKNVGRVRFIYENGKLLKTIKCTTAMMDDYMLTWSVRSTECKEVIYYMIEAGGTENPPGWSLTCKEASLGSVELPGAPPGPRDLVKITIVKSTTGGTNQLTLTYQGSDKRVSERILLSTHSKTATVEIRGKTGINRGNKLWFVTPNPRGSIDADMKLFDGAKVIVVQRLKDELIIKPPEVDPQVTVPGTTESPASLLDASNKAIVRFDYQEGETLSTVELFTDLLNTFNCKLWCNEPLEKREVIYGTIKAGGDYEPKDWSLSVSSNTINTVTLNCPPDCQDRAAAPDSATSCPGVGAMNEAYVRFEYILGEEVSTVKFQNETVPNRNLKTWLISSQDKCEIHYGIIPSGGDSDPKSWAIVMVSKSAQVVHLNVPPGPSKFPYQAVVPEFEVFQSKGYRNEAAVCFQYETGQTLRSIKISTRAITASVTQWATSGTDRKKVVYNTIESGGTYHPPQWSLSVFDASVEDVSLDCPPGPREFVNVLIEASTIGVSNSLTVEYVGVDVRTAEKIMIQSASDSTQVNVRSTEKLINGDKKWAVTPPPVGKFPVEIKLDNGAKVSFTNSLLNIIPGASTDKVAVPALLSKPESRGKENVASVRFFYEKGRTLGTATVELAKSEACITWTDDGKAQMEVIYRTIKSGGDHDPKEWSLKVTNSTVDSLTIKGPPLPAESVKVPETTEVPLGAGFENVALVRFYYFSGEQLNDIKYSNRVCTSCVSTFAKRAEERKEVIYHSIKTGGDILPTEWSITVSDKKVEKVELNTPASPSDLVRVNIFASKTGNCNEATLVYKGTDNRNVNEILLATYSNTVEIQARESLAGGEKSWKVEPEPRGKINVSLHLTSCARVNVIKRLASDLEIEPAELKEAAKVSGPLDVYEGIGAANMACIRFNYEANEKLRKVAYNTEAVGASPTCVTWEDEDTEKCEVVFGNIAAGGDYIPKSWTLSVTSSSYDTVKPNTPPGPPYTAECVKIYESPESPAGAGAANEAMVRFQYYHGETLGTCRFRLDNLRRKNCKTWRVGEKDRKELIYGDIESGGDSEPREWTLAISDGTVASAMPTSPASPTKFPNQAIIPDSELFEGVGFKNVGAVRFIYEKGPVLKSVKYSVAEVQTDVGTWTVRSTERKEIIYQSVCAGGDNVPPNWNLNCREASVFKVRLPGDPPGPRDLVRVNVPVSTTGGTNSLILVFHGGDARTSDRVTLTTHTLTNKIEMRMTAKDIEKGDKLWTVDPLPKGFYDAEIVFTEGAQVGTVSKFKQDIFATPGEIVDQATIKVSESPASWVGAGNKATVRFEYSEGETLGTVAFQNEIVNTFNAKMWRGDPLERKEVVYGTITSGGDYKPKAWELTINERTIVVAEPKGPPDCPDRAVYCESTKSSPSLGAINEAFVRFEYECGEHLGSAKVRSDYIPKKNVRVWRVGQKERKEIVYGSIRSGGDSAPRKWCITMCDSTASTVEVDTPASPITFPDMARIPESELTEGEGYKNVARVRFLYENGKLL |
| TRINITY_DN58818_c1_g3::TRINITY_DN58818_c1_g3_i4::g.91903::m.91903 | gi\|734595468\|ref\|XP_010731667.1\| | PREDICTED: microprocessor complex subunit DGCR8 [Larimichthys crocea] | 16.9 | MEIDDVLPPLPLEPPDDFGQDEGRAPPPPPLQTSSDAEVMDVSSGGDGYTYTPGDGEAPPPQPLSKGSITFCSHLSDEANPNPPCPRTARHAPPVTTFLPELKLLRDVKISVSFTESSNSKDRKVQYTGEGQEGGSDDGLDGLNGELHDWTKEQEVAESSSAAGNTGRRSEEAELDAENKVEFAVLDELDDFYDNFLDHDDGDNGGFKSEVIVQQEQADEEAGPYPYEEEFDNDVDALLEEGMPVPKKMRPAEDKYGGDSDHQSDGEGGVQPMMTKIKTVLKSRGRPPTEPLPDGWIMTFHNSGIPVYLHRETRVVTWSRPYFLGTGSIRKHDPPTSSIPCLHYKKMKEVE**EKELNGDATHN**TEESPVKPRQEANGEDGAGKSDAAAEDQDGAPPSALTDAAPDGEGAADSSQQLKESQLFEIAQGALGQVKAKVEVCKDESIELEDFRLYLEKCFDFEQVTVKKFRTWAERRQFNRDMKRKQAESERPILPANQKLITLSVQDAPTKKEFVINPNGKSEVCILHEYMQRVLKVRPVYNFFECENPSEPFGASVIIDGVTYGTGTASSKKLAKNKAARATLEILIPDFVKQTSEEKPVEGDELEYFNHISIEDSRVYELTNKAGLLSPYQILHECLKRNHGMGDTSIKFEVIPGKNQKSEYVMTCGKHTVRGWCKNKRVGKQLASQKILQMLHPHVKNWGSLLRMYGRESNKMVKKESSDKSVIELQQFAKKNKPNLHILNKLQEEMRKLATQREETRKKPKMTIMESAQPGSEPLCTVDV |
| TRINITY_DN61040_c0_g1::TRINITY_DN61040_c0_g1_i1::g.64248::m.64248 | gi\|808858429\|gb\|KKF10813.1\| | Polyphosphoinositide phosphatase [Larimichthys crocea] | 16.5 | TKHRVLKIDRTEPKDLVIIDDKHVYNQQEVRELLGRLDLGNRTKIGQKGSSGLSRAVSAFGIVGFVRFLEGYYIVLITKRRKMADIGGHSIYKIEDTSMIYIPNDSVRVTHPDEARYVRIFQNVDLSSNFYFSYSYDLSHSLQYNLTLLQRPYDLWSSTASSTEEEVHTQSKQDSFDIFEDEGLPTQVVYSLQTEPYYKYVWNGKLLERVKDIVHHDWLMYIIHGFCGQSKLLIYGRPVYITLIARRSSKFAGTRFLKRGANCEGDVANEVETEQIVHDASVMSFSAGSYSSYVQVRGSVPLYWSQDISTMMPKPPIRLDQADPYAHVAALHFDQMLQRFGSPIIILNLVKKREKRKHEKILSEE**LYPAVINLN**QFLPPDHWIDYIAWDMARYTKSKLCNVLDRLSMIAENVVKRTGFYINRSNFYCHTLRPDDRWGDVGGHITASGRVQTGVLRTNCVDCLDRTNTAQFMVGKCALAYQLYALGMIDKPKLQFDTDCVRLFEELYEDHGDTLSLQYGGSQLVHRVKTYRKIAPWTQHSKDIMQT |
| TRINITY_DN25699_c0_g1::TRINITY_DN25699_c0_g1_i1::g.56040::m.56040 | gi\|734625846\|ref\|XP_010741037.1\| | PREDICTED: prolyl endopeptidase-like [Larimichthys crocea] | 10.6 | MAFKYPAARRDESKVDDYHGTKISDPYAWLEDPDSAETMVFVEEQNKLTMPYLEGCAVRAQFHQRLTDLYDYPKYSCPYKRGQRYFYFHNKGLQNQDVLYVQDSLDAPATVFFDPNKLSEDGTVALKMGRLSEECEYFAYGLSSSGSDWVTVRFMKADDLSALPDVLERVKFSCLAWTHDAKGIFYNSYPRQEGKTDGTETTCNINQKLYYHVIGTKQSEDILVAEFPDHPKWHSSVTISDDGRYAVLSITEGCEPVNQLWYCDLQQLPNGITGLLPWVKLVDNFEAQYSYITNEGTVFTFRSNLDAPRYCLINIDIQKPDRQHWTTLLPQHDKDVLGFVSCLNQHHLLVNYVHDVKDILQVYELSTGKVVRDLPLDVGTVAGVSCKKKHSEFFYKFTSFTTPGIIYHCDLSEPIPEPKVFREVEVKGIKQEDYQTTQ**VFYPSKDGT**KIPMFLVHAKGLKKDGSHPVFLYGYGGFENSIQPYYNVAYLLFVRHLGGILAVANIRGGGEYGLTWHKGGTLANKQNCFDDFQCAAEYLIQEKYTTASRIAINGASNGGLLVAACVNQRPDLYGCAVAEVGVMDMLKFHKFTIGHAWTTDYGCSEEPEQFKWLSKYSPLHNLPQPPYSGPPYPAVLLLTADHDDRVVPLHTLKYCATLQHGVGSSPLQRQPLMVRVDTRSGHGAGKPTAKAILEDAHIFSFIAETLGLSWKE |
| RRRRRTRINITY_DN58259_c0_g1::TRINITY_DN58259_c0_g1_i1::g.44712::m.44712 | NA | NA | 10.9 | LQGFVEAERHSDLESFSRQNQVRFVLVFQFFAHHQTDTQTEGVQQIFVALLLLLLLVGQEDAVCYLDVLLLGGRYECRHLLLNFVHSDEQLVVILVVLLFVAVVRLLQQPFSSAARRQQGVSGSVLHRRLLLPSSVLLLQGVMQVFLGPASQRLLLSLLLNLLGSFGVHGVLVHRLHRSFFVDPLHLDEDVLLVLGDDHGDKTDGRREVDGGDLVDTHGYFS**VVGVQPDVE**VDGAARQASLFSVDEGVGVGCEDFFDRVSRGVHLSGAVRRHRGGVQLVSEADDLLHLLAVLRSLRLLLLLLADDLLVREDDVEVAAVGFEFVEVDSGVDCLLPHLARQLRVGVTEAERRDASVFHVDEVVVDFVGDELRAVPLHDDYQVEVELVGDHETLPAVRVDLEVRLDALDGRVQAFVLVHVVAVVQVRLLHLALHPVLELRRELVEDLLHLRQRFLLQLEFNGESVEPELAGGVPAGTQQLRRGAGEDVDSFPRGEHHRQVLDLEDVELADLEVQLVEVHDDHVLVVVLLDGHDVGHGDVREVGDLLPEVRTVDLVGLADLRQVPGLDHLRDDVVGLPEELLSAPREEVGLALSTASLFVRLRPLVLSEVRRRVFFLLLLFVAVFVVARLRLVRIVALRQAALLLAAFVPVLQLFLGPFRVSL |
| RRRRRTRINITY_DN596_c0_g1::TRINITY_DN596_c0_g1_i1::g.135::m.135 | NA | NA | 24.3 | MDLLHPHIATHDAKLLRRETAQASEFLTTCKRYHCNYSDKDTDYLREKELRNDFLQQLPERDAWRSIVKGDVFSSFMSTELFRSLFRLHSTPQVSLFSGKDFNFTGERNSLWSDLDLASHIVFDDYRGFMAAFRGAFVERLQINLKAAKLEEDEEE**FERGLTKGGESVK**GGGCRRALAQLRELVALEEGGLNGNRRDEMLDELVLSSMRP |
| RRRRRTRINITY_DN58944_c0_g3::TRINITY_DN58944_c0_g3_i5::g.113937::m.113937 | NA | NA | 20.3 | KNWKIYKKENKLGEKWFAELKPCLWLIHTHNGVHPGCNRWCKNSHTTSFKATIEPTRFFRMVVKWKFERWVNSNTVMHVETCIEEWMDHSINSNIEIEWREKVQATNNLNMSLFIQYCYSIIKKDWKKTQGVKILLEEILTPKTVKDWDKHKLLFDRLQLYRFFDTSPIKF**EESLQTFSK**FNGANHLQHLNTLGYESWKRFGTDLKSPVFDNMYGINTLASITKPLGFTSKIFTIKTWDGM |
| TRINITY_DN54218_c0_g20::TRINITY_DN54218_c0_g20_i1::g.59247::m.59247 | gi\|657588939\|ref\|XP_008298832.1\| | PREDICTED: myozenin-1-like isoform X1 [Stegastes partitus] | 24.4 | AVKEKRRKEYVRTYVSPWERAMKGNEELTATMRAAMPGPVYAHPDMPYYKSFNRMALPYGGYDKASKMLTFEI**PEINAPAEE**PEPVPNLQADIRSRPSFNRTPIGWICSEDNSHIHMDLDTMPFDGETDDL |
| RRRRRTRINITY_DN53303_c0_g4::TRINITY_DN53303_c0_g4_i1::g.16365::m.16365 | NA | NA | 23.9 | INGMGGLLAGKSLRRSIYT**RLEPEAESPL**SSDILTLSFDDHSTTSDARRQNWAVNEGSAESRTGQYLYITYYCNLQVKQSYGTIEESDFDDKEEVDISDQFSSTQSTFM |
| RRRRRTRINITY_DN22592_c0_g1::TRINITY_DN22592_c0_g1_i2::g.4335::m.4335 | NA | NA | 14.8 | PKVGDAVPLPVPLPVVLGARG**AVGDDVVQL**RVRRVLESDAGILVLAAAAPAFRDDVVGVHATFRWLLRELQTVRQGLGHFRVSRVSQERRTWSQVVPSSEGRWEFSGTEGSSSARWERWRLTAMVVSHRQIFSVRNSVGRHVTRGRRGG |
| RRRRRTRINITY_DN41303_c0_g1::TRINITY_DN41303_c0_g1_i1::g.113707::m.113707 | NA | NA | 22.4 | SAATIETSVPEIEKVAEEIVEIVQAMVEACKGSSPKETESETEPQSAAAEEEAAPTEESVTSLKSASAPSEAKASTDEAVPGNSDFEVAAEEEVSEEVVEATQLVVQVPMHIELVKEKNEESQPKVEEAAEKVDPEQVTEEEEKSQEEKVEEPEEGTQSEAKIEVVEEVVISEESVKVEVAKKLEEEPKKEEEVAPADTTEIVEIPVNDTVEMAVHLPRPSKPAPKECIVAVPTETIVPAETTMEIEKETTATAQVPTPTTATPTDPKPPEPKEESVKDMADQIVAQAIVMSQAEIEGAEHVDEKTEEMEENAQEAVASIESEATQVIAAEVMEVVPTDCVADKVEDSIAVQDSEPSLSAVLVVTETIVPAEESPMAVPIAPEIEKTITIKATECITSSLEETVVQVVEKIPTNEETAALTETDLKVEKTEVTPESVEVSQTCIASESTPTIPNVVVAKPSNESVSTAQIVEAEQPVTNIVGAKEIEPEDSETATQVDMVESQSTEVMPELDVTEVQEAQAMTVEDEGPTAEETSAPKEEISVESSLADRVVALCEVVPVVPKELAVKAIEKTGLGTCIAMAEDQAHPQLIANEKIEATETPPEYETNETAVELPVDEVTVSTQIPIESIQAEVAAVVEEATGSQPGQPVPTTVAPTAVAVPKEEATSLRRGMRGSVKPPISTQEEAEDEAVDTDVSKPTAADAEEPIDSLQDHKEEADEVSAPIWSPSRDETSAKVQVAAPEAVVEQEAEVQEDYESMPVVAPTDSDEEGSGVETSAQKEAKKKRRGPFFKRLSFSSEDKPAESDSQVQEGGEEPKEESKAKNKSMVMRKLTDWTSEREPPSSLPEPSSAVEGDSETNQSSVIAAETKGEQEGEAAAAEEDIKTEDDDSDSTKRTRKKPGGMCMLAEWSVSTDMKRKPEETSSVLKETNETKPEEEAPKEVEADEEVSKDALAASESSPLPKAPKEGTAEDESESSRKVLKKPTVLKKFSSWAIIGEKKKEGDSTVEGEAEQNSGNQTVEVAIVHEGSEEPSSPGSDAKPAETSETSSQLQEAAQEGSETLKSEADKKTKQKKTSLKKLGAGTFLKKLPSGQPKVKEQSSLLEAESTVEVPAKEEEAGGEAKEEEKKTEETAAAAPVATEVTVEAEPTAEPKAEEPTAAAEEKPESKDEEPTAAAEEEKTEQEAEAKEEKEDVAATEETKAAEEEAAAEKDEEEKTKKISAKKRLNSFLGGSFLKRFPSLTTEQSPPAAEGDKEAEDEKVAEDTAEATTGEAPADTAEAKEDNEKTVEAEVTAAETEAEKEEATSKEEAAEEEKTPEETGSVEEGEKDKVTLLKVPDKEESKDKKLTFKFGVFRFIKKFGVESADDPKEEAAKEEVASIDNADPSEKEVKEGNVQPAVEDQITGMTEPAEEKQAVSVGDPQGVTLTITGLM |
| TRINITY_DN58368_c2_g1::TRINITY_DN58368_c2_g1_i3::g.45497::m.45497 | gi\|908453958\|ref\|XP_013132059.1\| | PREDICTED: immunoglobulin-like and fibronectin type III domain-containing protein 1 isoform X1 [Oreochromis niloticus] | 26.7 | MWKKSKVTDQTAAGQAGIKKKSKVPGVMITQFKEELPEGQSTPDFTRKPIALTIQEGKFAVFKAIVVGDPTPAVTWSRANGEIVFHPEVCQQNYDEASHEHTISFPKVTPEDADTYKCYATNEHGRAVCTVVLNVIAVGFSKEKELKKTQSGEDITDIRKQLKKRNPDGTREHGKVMEDEEKVWEILLSADKKDYEKICAEYGIKDFRGMLKRLSEMKREREEEIAEFVSHITKLKPIEVTAEDFATIELDMDLKDPNSKVFVYKDGIMVAFSKEEGEGLKHSLKQIGKKFIFTISKLGKDDAGLYSVDVEGVNVFSSDFKVPEVDFAVKIREVKAEEREDALFQCVLSAPMNEIKWFGKNAPLTNGEKHQIIVSEDMLIHKLIVRDCMPLDAGIYAAVAGIKSCNAWLVVEANKDPASKGKKAARKTTVAGGGDDEDLMRIAKEQQEKYQKEMQEKLERAKKEKEEKDAADAAAEAEALAARKEAAKAKARAKAEAKARAKRAASGKSMKSGAAGGA**DGAAGAAGAGGAGGAGGAGGAGGAG**GAGGAGGAGGAGGAGGVDGAAGAGGAGAGGAAGGEGAGVGGEFGDEDFDSLEFSDDDLEGEGEGGEGGEGGKGKRRRARDGPLVPDTVI |
| TRINITY_DN59145_c1_g4::TRINITY_DN59145_c1_g4_i1::g.116184::m.116184 | gi\|657536573\|ref\|XP_008274854.1\| | PREDICTED: protein furry homolog isoform X2 [Stegastes partitus] | 16.3 | MDGAAVCWSKASVISFIKGLASPVGNGYNKPPIPPVCCPQGEKGPPAMMPISIDPESKPGEYVLKSLFANFTTASERKIRIIMAEPLEKPLTKSLQRGEDPQFDQLISAMSSLAEYCLPSILRTLFDWYKRQNGLEEELHEYRPRANTKSKNDEQQRDYLLERRDLAIDFIFSLVLIEVLKQMPLYPVLDSLVNEVINLAFKHFRYKEGYHGPNTGNMHTVADLYAEVIGVLAQSKFPAVKKKFMTELKELRQKEQSPYVVQSTISLIMGVKFFRIKMYPVEDFEASFQFMQECAQYFLEVKDKDIKHALAGLFVEILVPVAAAVKNEVNVPCLRNFVDSLYDTTLDLSSRKKHSLAFYPLVTCLLCVSQKQFFLNRWHVFLNNCLSNLKSRDTKMARVALESLYRLLWVYMIRIKCESNTATQGRLTTIVTTLFPKGSRSVVPRDMPLNIFVKIIQFIAQERLDFAMKEIIFDLLCVGKPAKAFSLNPERMNIGLRAFLVIADKLQQKDGEPPMPNTGCTLPSGNTLRVKKTYLSKTLTDEEAKVIGMSQYYFNVRKAIDNILRHLDKEVGRCMMMTNAQMLNKEPEDMITGERKPKIDLFRTCVAAIPRILPDGMSKPELIDLLSRLTIHMDDELRLIAQNSLQSLLVDFSDWRDDVLFGFTNFLLREVQDTHQGLLDTSLKLLLQLLTQWKLTQAAPGRSYDTAKIHTAELLQTSSSMKIPPERGPHSTVLHAVEGLALVLLCSCQLSTRRLAIAILKEIRSLFMTIGQTEDDDKPMIEIMDQLSPVILESFVNVAVSDTAALPAGHHVDLQWLVEWNALLVNSHYDIRSPSHVWIFAQSVKDPWVLCVYSLLRQDNLPKHCPTALSYAWPYAFTRMQMLMPLVDPNNPVYAKKTSTSGGGDNYVTLWRNYLILCFGVAKPSIMSPGHLRASTPEIASTTPDSGGSYDNKVIGSPSVAWLLKQLVPLMRAESIELTESLVLGFGRTNSLVFRELVEELHPLMKEALERRPENKKRRERRDLLRLQLLRIFELLADAGVISDSTNGALERDTLALGALFLEYVDLTRMVLEAENDKDAEILKDIRAHFSAMVANLIQCVPVHHRRFLFPQQSLRHHLFILFSQWAGPFSIMFTPLDRYSDRNHQITRYQYCALKAMSA**VLCCGPVF**DNVGLSPDGYLYKWLDNILACQDQRVHQLGCEVVILLLELNADQVNLFNWAVDRCYTGSYQLASGCFKAIATVCGSRNYQGDIVTLLNLVLFKASDTNREIYEIAMQLMQILEAKLFVYSKRIAEQKPNNILYGTHGPLPPLYSVSLPQLSSQLARMYPELTLPLFSEVSQRFPTTHPNGRQIMLTYLLPWLGNIELVDSGLLLPVFTPCTSDYDAPSRTTSTNSSHQLRGTGWGSLQATSMVLNNLMFMTAKYGDDLPGPEMENAWNALVSNDKWSNNLRTTLQFLISLCGVSSDTTLLPYIKKVVIYLCRNNTIQTMEELIFELQQTDPVNPVVQHCDSPPFYRS |
| TRINITY_DN59185_c2_g1::TRINITY_DN59185_c2_g1_i1::g.117514::m.117514 | gi\|808874097\|gb\|KKF23281.1\| | hypothetical protein EH28_21636 [Larimichthys crocea] | 17.8 | GGGGGGGGGGGGEEVGEKGRLAKEHSSFSQPEGSVRGVSSAETETCPPTDVAESQLKSQSWREPIATITESICTEQDRLSHPCQEQHGAAISPLPTHPEPSSGNTNAGGVRADLTQNLISEEALSSGHACEESSLTETERNYSQDLDSLIQPQPLTTSQQIAVERQESNNQQVRPESSTTEARTAESAAEIQVQAQGQSNSGTMPGVGVCASRGGTNRVHFADTVKQEGSSTVAVRNKPVSPMDCASLPPLTVHENLHYPVVEASFIFPDFLSGKKPEIPINAAPSKDEAAKQSSADFPKPLKDGQLDKGGSETKSIAVDQSGNNILVTNTVDLQLASKAGTKQLPSPAEKNGTSNKECFDLSQTAGSAVASADHATVEQVSAKALKQEEEKTEKSPIKLDLSKEKELNKFHAESTDSVKSDQPQESPLISAVPEEKEGNQQPLHSRPELPADDVTCKPLNDVTALSAGTDLSLTPEAVNLTCAASLQTDTSDPSYQLPTQSDQTPPCGLVISDPMKASEGAKPTVHSADQTEDESVTSEVKPSDQSMPVIEQCASNPAFVLQPPGPMLTHLEFITDSDIRPPEQTDERRADGDSTKVSGELDGNRSGETTQMSSAQDLKQTNASVTADEADPKPEDRNYAEMSVIELEKSAIKNENILFSQEENVCAPVKLPATLAVTDNVISLSQTEPDSIAIKAVICETSIRDGLINESCPLSSDLPSNDDETNKDNMTEKQKMGDGQDGQTWMQFEEDKKQSGEATMDNEEKTEISSKLQAGKKSTAPQLSDEPDKEVAGELRDPLQPPHEHKVEKSESPVRDKTEEGEGKREIASKSETETSSLSSPTRPAGPSEDTVPTEVESGSEPPTVYDPSLSQTPTATLERSSDSNTAQDLSAALSQSQSTSDPNCFAQQQEQQQQQECLGSRHPTEELSGGCLKGVDETQTLVPGVKGVAEGWDGSIGSLQPSESRDELTCDDSGGSDGVKGLEREDREGAGAVKGADEVYVLSHVGSDLNESGKAAEQNASVDMVITTASSHVGETGQAMDEIKSLGLGFVSSDAELMPGTEFTTDMGGKGQQKSNLSAACQDQHGQPETSTNTAASVEPASQEHETSPSQISISSKANRDSQDIHAEALPSANRSEEIHQENVSVLPDPVGQAETVEKDVSVALAVKDNSSETEECEILDTVSEPLELQSPNKTSATQSSPAVQTAIKGSDVEGITREDKAALDEEKASGQGTAQTEINTVNSGATEKQGVENQILSCKDSESVVSHSSSEQTKTTKNDSGVTDSITPDVVTVPAAVLSKCPEDLASLGKPNDQASLSEPVDVIVVKPIVAASQREGKPLDEAPACVSPAEGPSVNAAAADVSDSPAPKPTAPEPDTNWIKALKEAASQSQGEPVHTAETLRPLPSLESPQLEFHTPTEEIASPLRQEEIPPPEQAAEKTTEIPPPPKKPVDLPEPL**KKLADLPEPT**QSTREELSEPTKEEEEPPRELPEPERESVTSPEIVEGPAELPEAPKSTEDL |
| TRINITY_DN44057_c0_g1::TRINITY_DN44057_c0_g1_i4::g.10034::m.10034 | gi\|1007764938\|ref\|XP_015821192.1\| | PREDICTED: calphotin-like isoform X1 [Nothobranchius furzeri] | 63.5 | MAASLLRIGRLGCVKCLQTESWITLSRAPAASAYSSKSGGNKKSSKKNSSDKNQAQTYFDIEKLVQHKPYELPKKEVSSVAAAAAAAAAQPVAEPVASAAASEAV**AAASVEEATNPVV**KATATADAVSKPDLPPEAPPVVEAPPTADAVSELAAGTEAAAVEATTEAVVEAAAAPAVEAAPAAVAVPVAEVATETVVEAAPVVEAAPAEEATPVAEAATETIVEAEAAPAVEAAPAAEAAPVVEAAPAEEAAPVAEAATETIVEAEATPAVEAAAEVIAEAVAEAAPVVEAAAEVLVEAVAEAAPVVEAAAEVLVEAVAEAAPVVEAAAEVLVEAVAEAA |
| TRINITY_DN32908_c0_g1::TRINITY_DN32908_c0_g1_i1::g.32032::m.32032 | gi\|734627401\|ref\|XP_010741883.1\| | PREDICTED: lon protease homolog 2, peroxisomal [Larimichthys crocea] | 24 | SFDGDSQEVFVCRICFPPKKTVTSENKSTMASGSGIHIPSRLPLLLTHEGVLLPGSTVRFSVDSARNMHLVSQRLLKGTSLKSTIIGVIPNTRDPEHDTDDLPTLHKIGTAGIAVQVVGSNWPKPHYTLLITGLCRFRVSSLLKERPFVLAEVEQLDKLEQYTTSSTEGVATDDAELGELSQKFYQAAVQLLGMLDTSVPVVAKFRRLLDSLPRETLPDVVASMIRTSNKEKLQVLDALSLEERFKKALPMLTRQIEGLKLLQKTRKTNPDNERVLSVRKGGVFPGRQFNLDEEDEDEDGDDMAVLERKVHGANMPEAALRVCLKELKRLKKMPQSMPEYALTRNYLDLMVELPWSKSTKDRLDIRAARTLLDNDHYAMDKLKRRVLEYLAVRQLKTSLKGPILCFVGPPGVGKTSVGRSIARTLGREFHRIALGGVCDQSDIRGHRRTYVGSMPGRIINGLKTVGVNNPVFLLDEVDK**LGKSLQGDPA**AALLEVLDPEQNHSFTDHYLNVAFDLSQVLFIATANTTATIPPALLDRMEVLQVPGYTQEEKVEIAHRHLIPNQLEQHGLTPQQLHIPQDTTQDIISRYTREAGVRSLERKIGAICRAVAVKVAEGHRVTKTDTLIPEGPVEPGDKAAPPEMPIVIDHVALKDILGHPLFEMEVSERFTLPGVALGLAWTPLGGEIMFVEASRMEGEGQLTLTGQLGDVMKESAHLAISWLRANARTYQLTNMVGAPDPLEGTDIHLHFPAGAVTKDGPSAGVTIVTCLASLFSGRLVRSDIAMTGEITLRGLVLPVGGIKDKVLAAYRAGVKCVILPKRNEKDLEELPANIRADLDFVTARNLDEVLNAAFDGGFQGTASTHTPPQLTSKL |
| TRINITY_DN86221_c0_g1::TRINITY_DN86221_c0_g1_i1::g.95108::m.95108 | gi\|657574681\|ref\|XP_008291057.1\| | PREDICTED: titin [Stegastes partitus] | 16.5 | ASISHYIIEKRETSRLSWTMVEPKIQAISYKITKLLPGNEYIFRVTAVNKYGVGEPLESEPVVARNPFTTPSAPSTPEASAITRDSMVLTWERPEDDGGAQIEGYVLEKRDKDGIRWTKCNKKRLNDIRFRCTGLTEGHSFEFRVSAENAAGVGKPSAPTHYFKACDATYPPGPPNNPKVIDHSSTTVSLSWSRPIYDGGAKISGYVVEMKETADDEWIICTPHSGVQATHYTVKKLKENAEYHFRICAVNCEGVGEHVDLPGSVIASEKLEAPEIELDADLRKMVNIRATSTLRLFVTIRGKPEPEVRWSKADGTLNERAQIEVTSSYTMLVIENVDRFDTGKYVLTLENLSGSKSAFINVRVLDSPSAPTNLEVKDVKRSSVSISWEPPLIDGGAKIAHYIVEKREQKRMAFTSVCTNCVRNSYIISDLQEGGRYYFRVLAVNELGVGLPASTEQVKVSEAPLPPGKIVLVDVTRHTVTLSWEKPDHDGGSKITKYIVEMQPKGDDKWTVCSEVKALEATIEGLLTGEEYSFRVTAVNDKGKSDAKPLAVPVVVKDITVEPTINLLFNTYSVKAGDDLKIDVPFKGRPQPEVSWKKDGHALK**QTTRVNVLN**SKTSSKIIIKDATKEDVGKYEITLTNAIGTKTAEISVIILDKPGPPSNIKMDEVSADFICLSWDPPTYDGGCQINNYVVEKRDTTTTTWQIVSATVARTSIKVTRLNQGTEYQFRIAAENRYGKSHVVDSAPVVAQYPFEPPGHPTNVHVAHATKTGMLVEWGRPASDGGSPVIGYHIECKDQSSILWTKVNRGLLAENQFKMTGIEESLLYQFRVYAENIAGIGPCSKACDPVAARDPCESPHNLRVTNITRTSVSLFWEKPEYDGGIKITGYIVERNELPNGRWLKCNFTN |
| RRRRRTRINITY_DN55348_c1_g1::TRINITY_DN55348_c1_g1_i3::g.41807::m.41807 | NA | NA | 14.4 | LKAAMFNIHPKALPFLPLSRKFETMDQVLTPQPPFVCSHAPSASDVSTSQPLSPAPALNVDNQAPFEGVIPCSDMERSLVHVSVKHRKPADVMLRDRYFQMVNEKTLTKLYAVEINDRDFNYQQSIIEGWYKACEASLKKPKDLRRIALAQIHKQFAEDSMEEVAKEMTCLFAEVRSELYHPAKESQMIFRLGQVGNARRPGSFVIYGLQEKTRLTNFCPESIIQCFLELLMNDHTTQMDTQY**YIEIGCN**NHVENRQQYVYWGGDPVQVERYRILQSPLLPKTHAHEILTDEVMQMMGLASEKTINGHLLTEIHLRSLLQPIFAKLRPLTVDDLAERLEDKTWAVETMLLRLYYMAHQHPQEARFNNLSRMYAEKIIDFRREDIEFTAMKEIIKKLLIHQKDNYGKVSLLIANMGSAVGYVLGALRAAYTYEKLDDKLLRLLLYTMNCHLPDAYLYRSFFEFNLCAKPLFFKDDQKFWVKSMATDKILTPVSPSDKELPYIEFNTPIFENKMPLKFKGNLDANGWKKISEESIAEQKYQTGYWEETKDTQGEFSKSVVAVRVHEPRLKDLVMEILDPRFDELLYEAALVEELPYYHLLGAVKSTYGRPREKDKFRFAVKNLDKCEDFVWAQPGETRLKQIYQFMHFIIDEVHLLGEETLDVNIIFFMFGRAGEKQGGVLTNVWGKSKLESLLSGPGEHGILHGLYHGPNSKYYKQLDPIPFTVYLNRIDKIPVVKYFQRLHEEQFPHEPFEPIPVSKNEVEGFLNVVMATLEDLSERGLVCLGMLNSSYYTSHFTLLEQRIDVGDKSPRTELTLKNGTGFKSFPHNPNGTAKELQFLRWADNMLNKEHESDVANVERDKCSEDFLPCLFFQAFRDLAGQLHEHSVDFYYNTHEGSTFANSSGAHESLFQSYENEKPYKKTGLFLMHECFHALGSINGPDSLSGIQVDLAASSKDTTPDSILMAKLGNTFELGRYVRKDEPSRIIDSVVRDVAPDTMRLPLSSVLRFVQNFHFGTSASQIYRTHQAAQNICKFMSQVWPFCVKAKVAGDK |
| TRINITY_DN49670_c0_g4::TRINITY_DN49670_c0_g4_i1::g.57146::m.57146 | gi\|657591042\|ref\|XP_008299984.1\| | PREDICTED: peptidyl-glycine alpha-amidating monooxygenase isoform X2 [Stegastes partitus] | 16.9 | MIPGTMG**VPTLCVLVLA**FICHSHSLEMNDPLYRVKRSQENVLLDPSSCSSRTEQQIFTNRHNFSVALRMPGVVPAGSDTYLCVSFPVPTNRDAYIVDFIPHASMDTVHHMILFGCQTPVSTTNYWDCGSASGTCEDDPSIMYAWARNAPPTKLPKDVGFKVGRNSGITNFVLQIHYGDVSAFRDHHRDCSGLSLRMTTKPQPFVAGIYLLMSMDTVILPGKRVTNADIACDYSSYPIYPFGFRTHTHRLGKVVSGYRIRDGKWTLIGRQSPQLPQAFYPTSKGVDVKYGDTIAARCVFTGEGKTSKTYIGGASDDEMCNFYIMYYMDSRHAVPYMSCVDTGPKELFQHIPAEANVPITVSPDHMNMMHMGHSADQQDKKPLVDANTADQVLDQDNHLEQVSGWPQSSLQLGQVSGLALDSDSNLVIFHRGDHHWGGNSFNNQYRYQQRSLGPIQQSTILVVDPAKGNIVKASGRNMFYLPHGITTDKENNYWVTDVALHQVVKVSSDGRDKTLLALGEAFTPGSDSKHFCQPTDVAVDPETGDIFVSDGYCNARILRFTAQGQYLTEWGAGSSDRRRRIPFQVPHSLVFLPDRREVCVADRENGRIQCFIAETGEFVKEIKKEEFGGEVFAITYSPAGDGLIFAVNGESQYHSVPLKGFVINYSTKDILDTFSPNSKLQEFKMPHDIVETRDGSVFVGDAGSKTVFKFTTEKFHRSVKKAGIEVQELEEMETIIQTKLRPEHNMSKTAAVQEKQTVALQPQPQQKGKEDEEEKKKQEEEEKEEEEKKKSAAKP |
| RRRRRTRINITY_DN58414_c0_g1::TRINITY_DN58414_c0_g1_i3::g.45922::m.45922 | NA | NA | 18.2 | HQLQKMRNLASGAQFWEIQTENFTHNLSISQQSGDSHQIVATLPKGPGLSNLGTISIKDDPRIKDYDAPDAFTLPLLGQKKLNTEHIRAFSKVIIARGGLHRPELAAHERSSGEGYNEDGVVVWNVGKAKYDRAVDPVGGYEGTLQNKVKNVADNEINVAGILLNNSINDLHGRFKLWPGAASIHDTTCKGKVKILVRMNELDKGNWKDFPELLQLRNSSPSVDVKVSGGETPPHQYTDQGPDFDRSPLEDGTPPELKFKDGNPATLFDTEPNFNLTGALAMATVIEPSTVFAHTAPNADNRATFNRNFSTVITNKEGKKVDKRDWQGICPGCANALVVGGVDSLIKAYGDREITARIQESGPTVTFQAKCKLGKDLAQKALSAARGMDEYSSNTCSGILGVKVELPWGSKEAVAGVDAVPHALDPTFPGNIHPKLEDLNLEILEDYECGKDPVLLDSYADALLAIQGRGTKELYTKMRHNYPFVSTTAGIEAGMNCITAMGTCSISDVGPGHYEVIAGTGGKVTLIGAVKLIVDKPSTWGSLSGTLKVGIVKPCKLEWPIGAMVDVADAGGVGICIAGLGGGNPTHSDTGILMVGPYAYNELIIQHIIGSGPKWFGVGYKAGASALFNYVEQNIDKARALDKVGGTQAEILHDCHITSPVAVKPLGSSIFQLMAMQATADQMAVRDPRLRLYTRGRDIDQNHPDDLHGYVIKESLTLPRSLRKRVIDVNSQLKQYNVFSTPEFR**SMSVQAKARYA**ASVHLRRAGHGLALQLRAVTLCYTAMKNVSLSTSSPPSTVCNY |
| RRRRRTRINITY_DN53007_c0_g1::TRINITY_DN53007_c0_g1_i4::g.15702::m.15702 | NA | NA | 21.8 | ESLLLRKPKNRRAASPIPQRNQGSNASDTPDPILTPSQTPGLSPTVAPTSNLTTAPAETKTQLPADVPPREKPKRGRKKGTTAGNRVEAKIGNNELAKSKSSSVVQTAAARFWNQSHTKSIHYKVGSESSFEKQCILCTYKGLEGGGQSCNALHAKLGSVSSYVAECNENTCCIFGKEKVQNKWTELLEPSFNPLGPRTYNLRKSDPVLDDKIRRKTGWDKALEDEAIEQLHFVAVQASRRRVRGSPTREFDEGQSVNQVVKGNDREREKGKERDKERAEREKEKPHGSPAKEQWDTKDPQSQEKKGGSPSEESVVREKGTNNNPDTINDKSNNNTAASTTTTTIIPSHETRVHYDRGAKSRYTKGCHQCLFVPKEDDSLEGGCRKQHYQYGMLSSFHAS |
| RRRRRTRINITY_DN43812_c0_g2::TRINITY_DN43812_c0_g2_i2::g.107305::m.107305 | NA | NA | 19.6 | GHITKNWHTKSNRYLAPFMIPLIKAANDSILSMIYENNWYYLAREAVQFHPSSVCKALQRFLPEQVKVFESPEIVDLIEELENLFMVEKPSHTKPWYKLLAMVVPETLTSDKELFQVVCYALQPHYVSLSKVKHLPLLVKLLFIKHEEKLPLAFGNIISGLIELLEAIGNHHETEYIFRYFINNIQKRIYARLGLFKGYIRHLTTKLFDRERPDESDFLELLQMVFKQDIYKKAINPQFDPSELFRLFFEYVLQLHPWAAELTPEDEEPDFEAGTPNSSPPLTRFMNVAFMHVVEPYIPETIVNRNHTIYEVMESLAARKVEKWKLDSLPDSLFDFLVCCQRLKQIFLKEQEAPAVDRFHMLAVPQFPGNSNPA**DVVMRDGSKNCT**LM |
| TRINITY_DN43602_c0_g1::TRINITY_DN43602_c0_g1_i1::g.104726::m.104726 | gi\|657587916\|ref\|XP_008298272.1\| | PREDICTED: transmembrane protein 41B [Stegastes partitus] | 24.5 | MAKKRRERRETDGLLAQEEVKAAVTDSQQLKEAQHNVGGSARMSLLILFSIFACSASVMYLVYRNFPELPDDEMEKIKIPKDMDDAKALGTVLSKYKDTYYTQVLVAYFATYIFLQTFAIPGSIFLSILSGYLYPFPLALFLVCLCSGLGASFCYMLSYLVGRPMVYKYLTERAQKWSQQVDKHRDHLINYIIFLRITPFLPNWFINITSPVINVPLGVFFIGTFLGVAPPSFVAINAGTTLYKLTTAGEAVSWNSLA**VLGVLAVLS**ILPVCFQKKLQQKLE |
| RRRRRTRINITY_DN56848_c0_g1::TRINITY_DN56848_c0_g1_i1::g.42584::m.42584 | NA | NA | 20 | HQKATEQVEQEATETCVSQSLEEAQRDDKEAVTVVTKGRAHGQELKQFAQPVQTFPFQAEVVPLIKGADVLKRIEDLAPGDPAYFGWRYFVGSSIINQIAKSHLTLGAHITGDLLGMSDTNLLLPTVLTIYKAGSWPKLLGMAWDETDGGINDLILDFKEMMELQDAVDGATYDVVEDAGLGRVLGEANQSCTVTVHAGWAKLLQISFTGVGGSGGTILVRKDSSSD**RCLGGANVLA**SLATSAVYPISAAEAHSVLEPKHSVEYETLTLFEALSGQKWPPVAAWVEDGPAFHTVESGCDMVVGSVDRGLIVPFESDSDTMVSMPDKRLKLLKAGYGGRMSIDLPNLSAAHVKILVESPSSVAPVTVEDTYRLVGNTGYQDIVWASMCSQLRSPSSCVHRRCCVSCGARALAKTSGAVKSGLLCLLRASAM |
| TRINITY_DN45645_c0_g1::TRINITY_DN45645_c0_g1_i1::g.97043::m.97043 | NA | NA | 36.2 | MPNTKTPPLKTPPRSLKTSTTRNQKTSASARLPVSCSSSGRSFCLRQ**VSPRSPIL**HRCCPDLQGRKRRPRPRPGSPLPPPPPAVQLKTLQPRSPRTAPNRTVTRARRPEPPPPQQPPPPPPRQTNRRPPAARSRPRGRRKK |
| RRRRRTRINITY_DN110183_c0_g1::TRINITY_DN110183_c0_g1_i1::g.129779::m.129779 | NA | NA | 47 | TATTTAATAAATTATTA**ATATTTAATAAAT**TATTAATATTTAAAATAATANHTPEEQGSCGAPPKRAPAAGPGPDQLRPDSSQAAAAAGPQLCLTRPGASGHQVHQVHQVHQVHQVH |
| TRINITY_DN50760_c0_g1::TRINITY_DN50760_c0_g1_i1::g.121839::m.121839 | gi\|734594222\|ref\|XP_010735607.1\| | PREDICTED: cation transport regulator-like protein 2 [Larimichthys crocea] | 25 | MWVFGYGSLIWKVDFPYEEKRIGYIKGFSRRFWQGSTDHRGVPGKPGRVVTLVEDPEGCVWGVAYKLPTGREQEVKSYLDYREKGGYQVITVTFHPRPPLVPPPNQTLLYIGSQDNPDYLGPAPLEEIANQIVSSTGPSGQNTEYLFQLADAVRT**ILPEDLDAHL**FSLETLVRERLQSVQKHSHSQTG |
| TRINITY_DN27493_c0_g1::TRINITY_DN27493_c0_g1_i1::g.5557::m.5557 | NA | NA | 25.7 | METFTDTCTCRTCTPREQTTSQAPVCQSLPVTSLAAVLSSVRWRSTNTTTTPCTDM**FAAPAVAPC**QVPGSWTFTFRSGTTLSSLSSPRGRTCTSVWWKAVDRSSGQVNTGKTI |
| RRRRRTRINITY_DN52176_c0_g1::TRINITY_DN52176_c0_g1_i2::g.68765::m.68765 | NA | NA | 27.3 | RSFDCLDSEALMDLRQVGATLQQSCVFSLQHLQPLAGAVGEDVTLFCQLLKVIVDCLCLRPEMFLRLL**FAPAFGAFQL**STFGFLTQNTQKHSVFSCHHHLHVTLGVKDGTILLELLQHLVYREGRQSMLLIFTLVPRSVDLADQQSFVQVFESHTFSSRQCILHSECYTTETLVHRHLPRHRD |
| RRRRRTRINITY_DN14691_c0_g2::TRINITY_DN14691_c0_g2_i1::g.71346::m.71346 | NA | NA | 24.1 | PQFAEDKSLQLHKWRRIAVKYKETGSTEGFVRRITVRMLTVTNDQNESLVLDLTDGVKVTKSKKILKRGNLRLKNDYFADEIKNRSIDLGAKMILDYRFSQVY**KEIDKYDKPQ**NPDEDGEVEYDSDEPDKEDEDEEETQQATRATGKKKSSKFRLHHVSWSRVPDRGHLVLRHCGSAPFASCGWLQRTHISRPCWETQMLGYGAALLHRSNLRGLQRMALAQVVLNQM |
| TRINITY_DN37702_c0_g3::TRINITY_DN37702_c0_g3_i2::g.77697::m.77697 | NA | NA | 31.2 | VCVCVCVCVCVCVCVFTVLEQRKSKTLMFSQRYLSLSPSLPLSPSEKILSSHMKNQDEGTDVLQIVVLFFGLSGPGHVVI**VVFKQTP**SSFWRISFSWRQLEHLEHLEQVQSPPVSPLVCSSSFSLHWQRQQLSSPSSL |
| TRINITY_DN19292_c0_g1::TRINITY_DN19292_c0_g1_i1::g.3547::m.3547 | NA | NA | 35.7 | MWRRRCLHCCVAAQRDAGSRRTTPRTHSPLSLTDPRPLPRLRRLRSSRCSRLLLSSRRLRPRPHRHPLPL**YTPPPPLP**PLLLLLPPLPPLHHPPASPLPLCSPGSRCLCRSL |
| TRINITY_DN16613_c0_g1::TRINITY_DN16613_c0_g1_i1::g.103829::m.103829 | gi\|808887656\|gb\|KKF33984.1\| | hypothetical protein EH28_05835 [Larimichthys crocea] | 26.6 | QMRHTAHELRVTLDHVLQGGEPSVAPPNSQDEALSFLTEPEVEMRAVHNIPEDDLNFLFLAENLRVPKNLYERISEIADYKKYTSALLMILFDRETLATHSLQGRRNTFTGEDCHKPQLPPDILRSIIDHVAAKFGVD**CSQIKTAIRT**KLNNEDKLLKKRLGVGKAENKPSADQSFCQEASLLP |
| RRRRRTRINITY_DN54116_c0_g2::TRINITY_DN54116_c0_g2_i1::g.17246::m.17246 | NA | NA | 16.6 | LVVRDTPRDSSEGSHFCLLSAVVLSVTAIFSPQLWDEGTTQEERTDVSCMEYRRVAHQVHCLLISGRFTASNFPKTCIHSDLGPSRCRGSILKKRVQSSPIPAVIGGRALVPEHWPPQGQPGGGVDAEAEAGSGSAGARESLSSLSISISLSPTISGSVTSGCSMSPNSFASTVSSII**SKALSFFIS**AILARPSPSRCFARSSKESAMLSFGQTAMM |
| RRRRRTRINITY_DN4698_c0_g1::TRINITY_DN4698_c0_g1_i1::g.864::m.864 | NA | NA | 14.7 | DLCVYPLINLRSEDGHPDLSICETWDYWNGKTDYCGNVGSWHQAHLRSLDQDNVERSVGPLDKAPQEAALVHTKKADKSRHDEKKLFETMFFHASPTHAGSSTLVNSQQSLYSQIIESRSLEKWDTQQFPSPPVPPNQKPQESREPEPRHEPQPRVEGDQCSDKLFSTKIQGTVPDFTLRRDKLRVPKTGDDVTQGDLNVVYDKPRYKQRKRKGTSGVAGDDELSATDSAPSATSNKKCLPRSQSDSDASWLGDTHLGQ**VCVNLAQPSP**GLGSTDVSGPRLAPSSLRPPESQAQPATQQ |
| RRRRRTRINITY_DN20669_c0_g1::TRINITY_DN20669_c0_g1_i1::g.83915::m.83915 | NA | NA | 17.5 | WAQTQPQPQMQPPAYSPRMHAYGPAEVLPGTQAWQP**LAQVAPHAQSQLAQH**TEPHYPAPIPHRQQPTWVGTQTPSQTQPYGQPYLQPQAAAQPYPVSIPSSTRVQTWTQLQGQSPHGPQQVLAQAHTSYQTPAGPQVQTHHQPLPQGQSTWVMPQVQTQPHVLPQPHTQVQAMSQVQTQPHAKPQPHTQVHAMSQVQTQPHAKPQPHTQVHA |
| TRINITY_DN97605_c0_g1::TRINITY_DN97605_c0_g1_i1::g.27609::m.27609 | gi\|317419749\|emb\|CBN81785.1\| | DNA-dependent protein kinase catalytic subunit [Dicentrarchus labrax] | 40.2 | LRLRRRFLKDQEKVSINYAQKEIRQQRQKKEDKADERLKKEAQVTLYRNYRVGDFPDIQIPYSSLIAPLQALAQKDPILAKQLFSSLFAGILQEVDKDKPAE |
| TRINITY_DN58331_c1_g1::TRINITY_DN58331_c1_g1_i1::g.46034::m.46034 | NA | NA | 11.9 | MSVGGEDVPHAPGAAVGSEVRAPLRVLVRDSDPELRPVVHLHLPVVTTSDRDQIPQDGHTPAEGAVVEGHGADGQLLVSVEDGESVLGDGQTGMSRVLQQVEERSCCGDGDDITPVAGPADVDRPDGDEVAASGLQLDQTLTGRHGNDGLGDVALSAVGPQLAQLHCVRVHRVGVS |
| TRINITY_DN43949_c0_g2::TRINITY_DN43949_c0_g2_i1::g.101517::m.101517 | NA | NA | 25.2 | MGTKEF**DVDVTAATVT**SSSSSSLFFFFLFFSPPSGVTASSSSVTAATATASSSFFFFFLFFSPPPFSSSSSGGRTTSSSSFLFFFFFFLFSSPSLFCSSSSSSGGRTTSSFSSSSSFLFFFFFFLFFSPPPFSSSSSGG |
| RRRRRTRINITY_DN78144_c0_g1::TRINITY_DN78144_c0_g1_i1::g.94632::m.94632 | NA | NA | 34.3 | TVLCEHSVLVEPCAPLSLGQHHVQVELGQPNGQHCPVVPLCRGQLVLPFALPWLNVPAE**LTAQSPPL**RPGSRVVPGLLVPPCFPCFLIEQSPQALPLSP |
| RRRRRTRINITY_DN62534_c0_g1::TRINITY_DN62534_c0_g1_i1::g.97702::m.97702 | NA | NA | 26.1 | KPLEDETTLRFYGSMGFRFVIDMPKDAQGAKVSQ**KPADSKIPTLTLKVE**KGRSAATIRYAESTFSVDPSKSVESKRVPGTFVVGNCMKNVYLSALHLEPGEPMKIFTHWVLFLSRQQYVQLHIVGFTCTELTCHNDQE |
| RRRRRTRINITY_DN48872_c0_g1::TRINITY_DN48872_c0_g1_i1::g.72600::m.72600 | NA | NA | 13.5 | PNSSASSSSSAASSSVAQVNGYRPECYSNDDASVSLHDLFYLIVCADPNLEFWVVAARWVPYCLDHTRGVLSLVRCFDYGVQQPADLSSPRWSVAPASPTPAPLA**VPVHPGRTAQ**LLEGEGDRGHLGAEDNSFTTLRDLADALLRSGPDLQLSVRWGVGVGVDLHECDSAGRLLDVVGPLQHLLLDPSWDSRHAAWVAGRTWVSAWAWVTRRSRGSGGPSGHTWVVRPRVVLSRWPWM |
| TRINITY_DN86315_c0_g1::TRINITY_DN86315_c0_g1_i1::g.26591::m.26591 | gi\|734637300\|ref\|XP_010747330.1\| | PREDICTED: ubiquitin carboxyl-terminal hydrolase CYLD-like isoform X1 [Larimichthys crocea] | 24.3 | KFVEAPSCLLLLMPRFGKDFKMFDAILPSLS**LDITDLLD**DTLRQCSICQAVAEWECLQCYEDPDITPGRLKQYCATCNTQVHSHRKRSSHGPVKVRVPDGPWTGPLHCTRQ |
| TRINITY_DN80726_c0_g1::TRINITY_DN80726_c0_g1_i1::g.98561::m.98561 | gi\|657532668\|ref\|XP_008305048.1\| | PREDICTED: baculoviral IAP repeat-containing protein 6 isoform X7 [Stegastes partitus] | 22.5 | HEFLSRLQVHLSSTCPQMFSEFLLKLMHILSTERGPFQSGQGPLDAQV**KLLEFTLEQ**NFEVVSVATISAVIESITFLVHHYITCSDKVVSRSGSDSSVGARACFGGLFANFIRPGDAKAV |
| TRINITY_DN78259_c0_g1::TRINITY_DN78259_c0_g1_i1::g.26075::m.26075 | gi\|542199074\|ref\|XP_005473880.1\| | PREDICTED: monoacylglycerol lipase ABHD12 isoform X2 [Oreochromis niloticus] | 18.4 | MKRRAVGQTDPSPGPSEVQRAGAEDSAQGQPETESRWWLKRGLLAASVIFILVP**ISLRILPE**LIQHLVYTHRIRLPFFADLSRPADLSLNHTINMYLTSEEGISLGVWHTVPESQWKEAQGKDLAWYQNTLSDGSPVFIYLHGNTGT |
| TRINITY_DN76008_c0_g1::TRINITY_DN76008_c0_g1_i1::g.94422::m.94422 | gi\|657587270\|ref\|XP_008297918.1\| | PREDICTED: WD repeat and FYVE domain-containing protein 1 [Stegastes partitus] | 9.6 | KKKKKNTKQTMAAEIHSRPQTARPVLLNKIEGHSDAVNAAVLIPKEDGVITVSEDRTIRVWLKRDSGQYWPSIYHTVSSPCSCMSYHHDSRRIF**IGQDNGAVVE**FLISEDFNKMNHVKTYPAHQNRVSDMVFSLESEWVVSTGHDKSVSWMCTQSGSMLGRHYFTAWASCLQYDHETQHAFVGDYSGQITLLKLEKQ |
| TRINITY_DN54250_c0_g1::TRINITY_DN54250_c0_g1_i1::g.39997::m.39997 | NA | NA | 17.1 | MFTGINTYLTRLHQQLQVYVL**ECVLGCVLE**CVSVCVLWSAVSAASVPAACCSANMSVSFSSRTGSLDSAAACSLAAAAPLLLRTTPSDSNTFPVSSNTVDSSTSPRRCSSENLSTNL |
| TRINITY_DN42444_c0_g8::TRINITY_DN42444_c0_g8_i1::g.80898::m.80898 | NA | NA | 15.2 | SAFILKIKLFRVIAFIILYGIHSHRKIQSERSSSASVRVFFFQSFFSPKGKRRSEFSYFPQHLNAASHLNSLPPPPWSVGFGESVDACRDHQLMVPHLPLQEGLQLVDVHLRNNGPRGDEHPEHRVDPLQRHGVEVGQHGLDVGPEQLQLLLPLLRL**GGGVGVSLLVAV**DQHHSSLHLLLGRARLHHVVPP |
| TRINITY_DN36663_c0_g3::TRINITY_DN36663_c0_g3_i3::g.7766::m.7766 | gi\|734635203\|ref\|XP_010746177.1\| | PREDICTED: C-C motif chemokine 24 [Larimichthys crocea] | 13.1 | MRFCLATLVCIATWMSSVQANNGQNCRCLGVSTTRPRVQRIKNYTIQPEGLCPVKAVVFFMLSEKIVCSDPNRDWVKNAMKKVDKDTM**VPPVMEE**EEQVSTTVMQKVDTETTVPPVIEKKEEVSTTVMQKVDTETTVPPVKEEKEEVSTTVMQKVDTETTVPPVTEKKEHVSTTAMQKVDTDLVVPPVIEEKEKESSIV |
| TRINITY_DN28462_c0_g2::TRINITY_DN28462_c0_g2_i1::g.70660::m.70660 | gi\|1012770260\|gb\|JAR44010.1\| | hypothetical protein [Fundulus heteroclitus] | 17.7 | SHHVLGIKHLLSELGDCKGTVLLAAPAGQWSKARHEEVQTRERHHVDSQFA**EISIELT**GEAQASGDSAHGSGHQVVKVSIGGCGQFESAEADVVQSLVVDAVGLVCVLHQLVD |
| TRINITY_DN23496_c0_g1::TRINITY_DN23496_c0_g1_i1::g.4626::m.4626 | gi\|657576750\|ref\|XP_008292170.1\| | PREDICTED: proteasome subunit beta type-5 [Stegastes partitus] | 9.6 | LGALRGPAAMTQINVPGFTRRKRLVHQTVTKMALASVLNSDCADFSFDKCQPFGFGCGAEQSGLGFDATPGDSLSFSVKNPLCAGGDDGVERKIEFLHGTTTLAFKFQHGVIVAVDSRATAGSYIASQTVKKVIEINPYLLGTMAGGAADCSFWERLLARQCRIYELRNKERISVAAASKLLANMVYQYKGMGLSMGTMVCGWDKRGPGLYYVDSEGNRVCGDLFAVGSGSMYAYGVMD**SGLRQDLSVEE**ACELGRRAIYQATYRDAYSGGQVNLYHVHSEGWTRVSQEDVLMLHQQYKDQA |
| RRRRRTRINITY_DN57102_c1_g2::TRINITY_DN57102_c1_g2_i1::g.126058::m.126058 | NA | NA | 26.4 | SGGAGLGSGGGGTTIIGVGGGGTSGTAGTAGLTSTDTPQWVSYSGTKGGMCIMA**WPLPPTGPPGG**VCTLLSSLMSPLRCDSRLLSPTCVTALPTHPAQQLGMTPCTTVAM |
| RRRRRTRINITY_DN52888_c0_g1::TRINITY_DN52888_c0_g1_i1::g.15129::m.15129 | NA | NA | 11.2 | LTNYNLPKWAFRQCY**FLTGCRLAT**QTDRCRYSALTCPDVVAAEQGNCLQILSCIAQPTAYSLVADMVSKSFEGIITECQDRYSSPFIHCIEELLNIVATETREKPLLDELTKVLFLCFSCQTQPSANESTGAAQLAANVFYSLMKDQKGCSGCLGIMKCVQDPKAITSIFNIALPFMKELEDKCLKATAPPGPLHDCVSELGNMIKKQIDANSLLDVLLEIIKTCDQCVDGNPRTAEPVTQLSDAHLTLANCHLGISLFLLFTLLAM |
| RRRRRTRINITY_DN49941_c0_g1::TRINITY_DN49941_c0_g1_i1::g.117740::m.117740 | NA | NA | 17.9 | DLIRDGPRNWRWGELQQHETYQLRRDHFVVRIISKVMKGTIEPLEGIKRNDLMRIEYSQGQNLYTLTEDHLKVAPSTAACLVYQFPLIKNDTDPPLNSEEQKFIPLALVDSMSYAGAGLEQGIGSLSADFDQVLG**SEIVEDTLPL**KLAWAM |
| RRRRRTRINITY_DN102801_c0_g1::TRINITY_DN102801_c0_g1_i1::g.139401::m.139401 | NA | NA | 26.3 | LVHAWDFYHLVWVSGEGLRGHHLASQRLLPPLQLLRSSCRLHVADGSTEVLVPDVGHGVILSPLDSGVHSTDARHGGVDRRPLSVGESPVLHRVALLRLHGDQLLSLAQGDVLQLLLG |
| TRINITY_DN75480_c0_g1::TRINITY_DN75480_c0_g1_i1::g.94369::m.94369 | gi\|958251808\|gb\|JAO40423.1\| | VIGLN, partial [Poeciliopsis prolifica] | 14.7 | VAIPQRYHRAIMGPKGCRIQHITREHEVQIKFPERDDSASGQEPP**PQENGEVSP**EAEFVPRKCDIITISGRAEKCEPAKAALLALVPITEDVEVSYELHRYIIGQKGSGIRKMMEEYEVNIWVPQPEKQLDVIKVTGLVANVDRAKLGLA |
| TRINITY_DN49988_c0_g1::TRINITY_DN49988_c0_g1_i1::g.117669::m.117669 | NA | NA | 13.4 | MLVWWMSRLALTNTFITFPSLKYVLRVSLKLLEYFTNSFFCCSSARLFRYWYFLKTVLTVSSSS**FMALAMSL**MVCRKRTLSLISRGMVLRMCLRARALSFSSWNSGSRLSIMNSGTSSAAMRSSDSL |
| RRRRRTRINITY_DN89119_c0_g1::TRINITY_DN89119_c0_g1_i1::g.95393::m.95393 | NA | NA | 15.6 | IWPRLLILTFSVKVNFQ**EASDVGGGI**KVQFSFSVSICVKGTSSFSMVSTESLPFYVQSTIFLIVPFVDDATCSSVTVRKGSGGVGVGCGSPQEAPQMAPITIPQRTTIA |
| RRRRRTRINITY_DN22990_c1_g1::TRINITY_DN22990_c1_g1_i1::g.52719::m.52719 | NA | NA | 11.3 | EVTIASAPVATYQPIYTGQM**PAAAAAPMYTGT**TGLSLHNMQQTLPAMVSSPHLSLSPEIAAAPTIVAGTPQMVYQQHPMWSTSQVQYTSVPSAAIFPTISTQAIMRNTSISYPSSYFGNQMATPDYTLAMGSEGERTWPRGNQPYKVQTQRKKQGGDAFK |

*ORF code output from TransDecorder is shown.

** The gene ID or the definition (including name of protein and specie) of top-hit gene is shown.

***The color of character indicates the peptide confidence; green, yellow and red means the peptide confidence is ≥95%, <95% ⋏ ≥50% and <50% respectively.
