# Supplementary figures and images for: A Novel Analysis of the Peptide Terminome Characterizes Dynamics of Proteolytic Regulation in Vertebrate Skeletal Muscle Under Severe Stress
Source: Proteomes. 2019 Feb 13;7(1):6. doi: 10.3390/proteomes7010006 (PMC6473766; doi:10.3390/proteomes7010006)

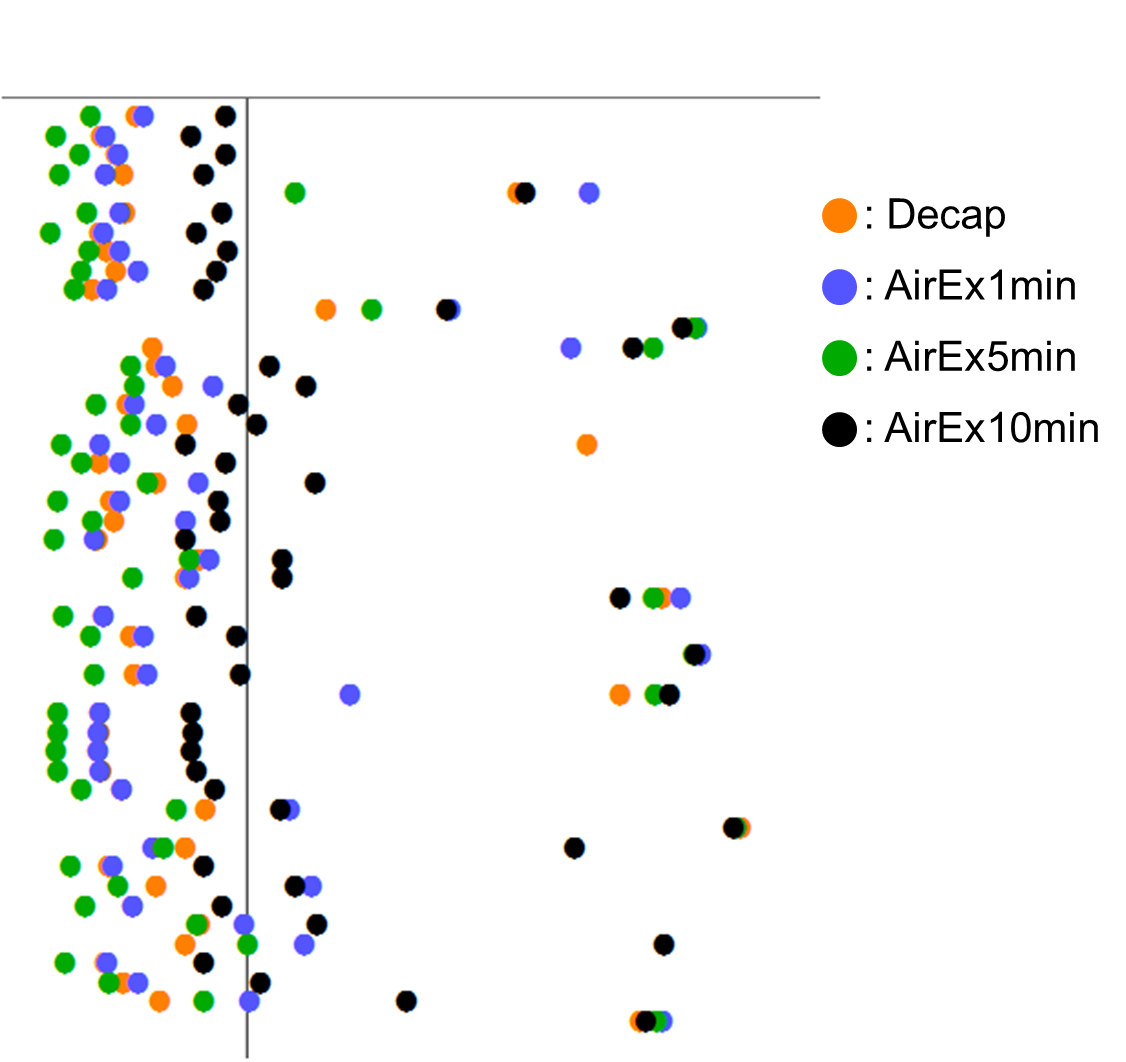

Supplement: Supplementary file 1 [file proteomes-07-00006-s001.zip › supplementary/FigS1.tif]
